# Electrochemical Rearrangement of 3-Hydroxyoxindoles into Benzoxazinones

**DOI:** 10.1021/acs.orglett.1c03569

**Published:** 2021-12-24

**Authors:** Marie Vayer, Miryam Pastor, Christiane Kofink, Nuno Maulide

**Affiliations:** † Christian Doppler Laboratory for Entropy-Oriented Drug Design, Institute of Organic Chemistry, University of Vienna, Währinger Strasse 38, 1090 Vienna, Austria; § Boehringer Ingelheim RCV GmbH & Co KG, Doktor-Boehringer-Gasse 5-11, 1120 Vienna, Austria

## Abstract

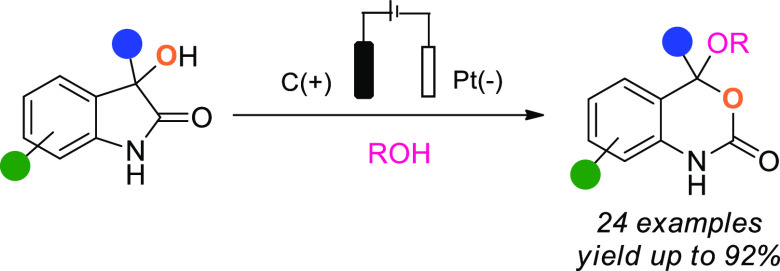

We report an unexpected rearrangement of 3-hydroxyoxindoles into benzoxazinones using electrochemistry. Our reaction employs mild and environmentally friendly conditions, and the benzoxazinone products are obtained in moderate to excellent yields. Mechanistic experiments suggest that a peroxide intermediate is likely involved.

3,1-Benzoxazin-2-ones constitute a privileged scaffold within the carbamate family, present in a large number of pharmaceuticals and biologically active compounds,^1^ such as the well-known antiretroviral efavirenz (**I**)^1a^ and its analogues (**II)** as well as (**III)** that are known to be progesterone receptor antagonists.^1b^ (Scheme 1A).

**Scheme 1:**
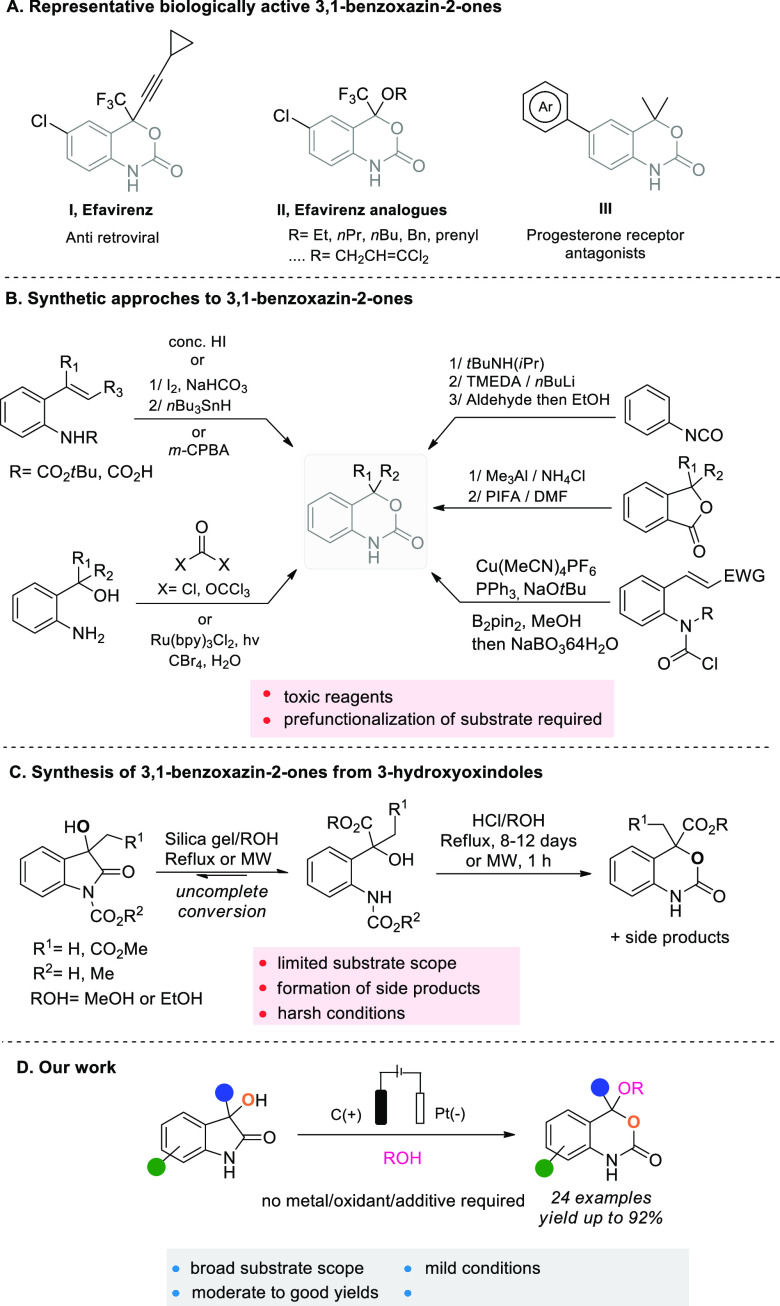
Biologically Active Compounds Containing Benzoxazinone Moieties, Relevant Synthetic Methods to Access These Motifs and Work Presented Herein

Given the prevalence of this motif in medicinal chemistry, there remains a general need for divergent methodologies that facilitate the preparation of a range of benzoxazin-2-one derivatives to support drug discovery. Classical methods to access such structures generally involve either the annulation of *o*-vinylaniline derivatives^2^ or the carbonylation of amino alcohols^3^ (Scheme 1B, left), relying on the use of reagents such as tributyltin hydride or phosgene and its derivatives, respectively. Other approaches form the desired carbamates either by double-lithiation of a transient urea, formed from an isocyanate, followed by reaction with an aldehyde,^4^ or by an aminolysis–Hofmann rearrangement starting from phthalides^5^ (Scheme 1B, right). These methods require the use of strong bases or stoichiometric organometallic reagents and are often step intensive. Recently, the Lautens group reported a novel procedure for the formation of 3,1-benzoxazin-2-ones which, while using considerably milder conditions, still requires a complicated system as well as complex and expensive starting materials (Scheme 1B, bottom right).^6^

While derivatization of 3-hydroxy-2-oxindoles through action of a Brønsted or a Lewis acid in combination with a range of nucleophiles, such as alcohols,^7e,7f^ thiols,^7,7f−7h^ malonates,^7g^ and aryl groups,^7a,7c,7d,7f^ is well-known, the use of 3-hydroxy-2-oxindole to access other heteroaromatic structures is more scarcely reported (Scheme 1D).^8^ The sole example of formation of 3,1-benzoxazin-2-ones from 3-hydroxy-2-oxindole involves ring-opening alkoxylation in the presence of an alcohol, followed by a second step of cyclization. However, the reaction is limited to carbamate-protected 3-hydroxyoxindoles and to methanol or ethanol as the nucleophiles and, moreover, proved comparatively sluggish and unselective.

As part of a research program focused on novel approaches to drug design, we became interested in the reactivity of 3-hydroxyoxindole derivatives, which seemed particularly amenable to electrochemical transformation. Electrochemical synthesis provides a multitude of advantages as an environmentally friendly tool, generally featuring mild conditions, good functional group tolerance, and high chemoselectivity.^9^ In the event, we observed an unexpected rearrangement of 3-hydroxy-2-oxindoles to 3,1-benzoxazin-2-ones under electrochemical conditions (Scheme 1D).

The initial reaction was performed using **1** as the starting material with the commercially available ElectraSyn 2.0 in an undivided cell (Table 1). A graphite (C) anode and a platinum (Pt) cathode were used as electrodes under a constant current of 10 mA, with tetrabutylammonium hexafluorophosphate (*n*Bu_4_PF_6_) as the supporting electrolyte and 10 equiv of MeOH in THF as the solvent. Encouragingly, under these unoptimized conditions, product **2a** was obtained in 62% yield (Table 1, entry 1). Increasing the concentration (entry 2) or the reaction time (entry 3) led to a decrease in the yield of **2a**, and it was noted that prolonged reaction times led to decomposition of the product (see the SI for details). Neither the addition of 4 Å MS (entry 4) nor the use of AgPF_6_ as a sacrificial oxidant (entry 5) proved beneficial for the reaction and the use of TFA (entry 6) or TEMPO (entry 7) as additives gave lower isolated yields due to substantial degradation. Subsequently, various solvents were examined, with DMF and CH_3_CN giving slightly decreased yields, and CH_2_Cl_2_, reported to be reduced at the cathode and act as an electron sink,^10^ also led to no improvement (entries 8–10). Gratifyingly, it was found that using a 1:1 mixture of MeOH and THF allowed us to successfully improve the yield to 91% (entry 11). Finally, a control reaction in the absence of electricity was conducted, and no product was observed (entry 12).

**Table 1:**
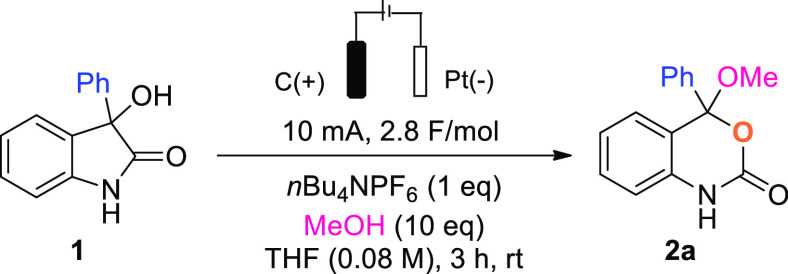
Optimization of Reaction Conditions^a^

entry	variation from initial conditions^a^	yield^b^ (%)
1	none	62
2	0.16 M instead of 0.08 M	47^c^
3	5 h instead of 3 h	20
4	4 Å MS as an additive	53
5	AgPF_6_ (1.5 equiv) as an additive	23^d^
6	TFA (1:4 v/v with THF) as an additive	25^d^
7	TEMPO (10 mol %) as an additive	25^d^
8	MeCN instead of THF	37^e^
9	DMF instead of THF	38
10	CH_2_Cl_2_ instead of THF	55
11	**MeOH/THF**(1:1**v/v, 0.08 M)**	**91**
12	without current	nr

a Initial conditions: undivided cell, Pt cathode, C-SK50 anode, constant current = 10 mA, **1** (0.4 mmol), *n*Bu_4_PF_6_ (1.0 equiv), MeOH (10 equiv), THF (0.08 M), rt, 3 h.

b Isolated yield.

c 50% conversion.

d Partial decomposition was observed.

e 59% conversion.

After identifying suitable reaction conditions, we set out to explore the versatility of this reaction in the presence of a variety of alcohol nucleophiles (Scheme 2). Aliphatic alcohols such as EtOH and *n*PrOH were well tolerated and yielded the corresponding products **2b** and **2c** in 77% and 79% yield, respectively (Scheme 2A). Similarly, when benzyl alcohol, 2-phenylethanol, or allyl alcohol was employed, the desired compounds **2d**–**f** were obtained in moderate to good yields. A secondary alcohol such as 2-propanol was also a competent nucleophile and afforded **2g** in 51% yield. We then examined the scope of this reaction using various 3-substituted 3-hydroxyoxindoles **3a**–**n** (Scheme 3B) and were pleased to observe broad tolerance of different substituents at the C-3 position. Substitution with electron-donating groups (OMe, Me, *t*Bu, and Ph) led to products **4a**–**d** in yields up to 92%, and halogens at the *para* position (**4e**,**f**) or a *meta*-OMe substituent (**4g**) resulted in moderate to good yields. Compound **4h** bearing a pentafluoroaryl substituent was formed in a lower yield of 20%, and a range of aliphatic substituents (**4i**–**4l**) was also tolerated. Additionally, we investigated the substrate scope using various oxindoles substituted on the aromatic (**5a**–**f**, forming **6a**–**f**) (Scheme 2C). All modifications, with the exception of substrate **5c** carrying a nitro group, were well tolerated and provided the desired products in moderate yields. Unambiguous confirmation of the benzoxazinone core was possible through X-ray diffraction of a single crystal obtained from compound **6b**.

**Scheme 2:**
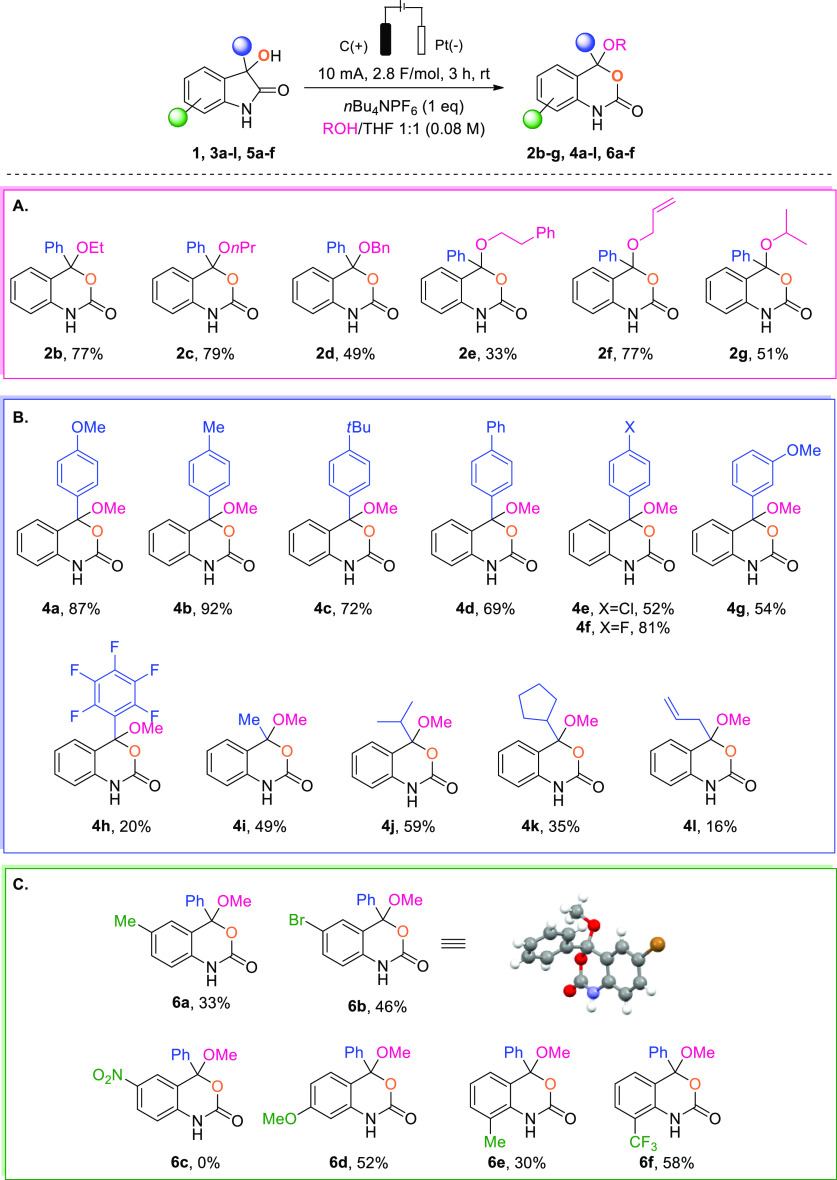
Scope of the Reaction

**Scheme 3:**
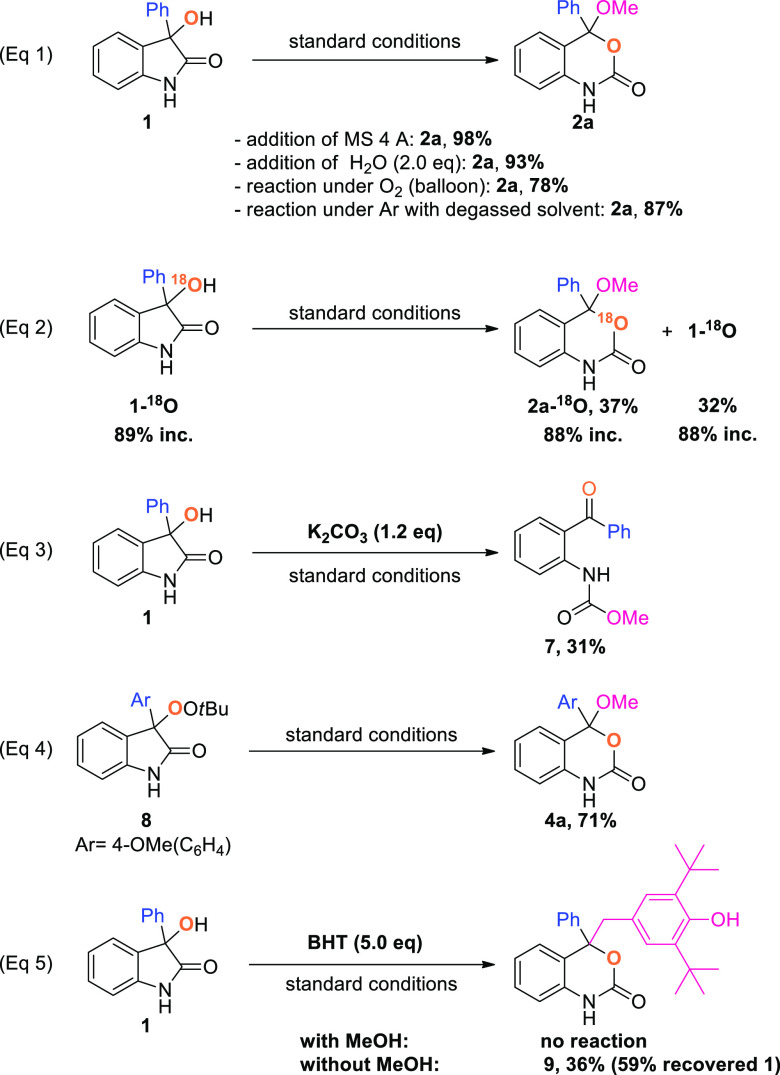
Mechanistic Investigations

The observed formation of 3,1-benzoxazin-2-ones from 3-hydroxy-2-oxindoles raised questions regarding the mechanism of this transformation. In order to shed light on the intricacies of this transformation, several control experiments were conducted (Scheme 3). Initial experiments focused on determining the source of the endocyclic oxygen of the 3,1-benzoxazin-2-one. Water and atmospheric oxygen were ruled out as possible sources after it was found that neither the addition of molecular sieves, water, or molecular oxygen nor conducting the reaction under an inert atmosphere with degassed solvents had a significant impact on the yield (Scheme 3, eq 1). In addition, these results highlight the robustness of our protocol which proved to be tolerant of water as well as oxygen. Suspecting the endocyclic oxygen to stem from the hydroxy group of **1**, the substrate was labeled with ^18^O (Scheme 3, eq 2). Under standard conditions, the corresponding labeled 3,1-benzoxazin-2-one **2a-[**^**18**^**O]** was isolated without loss of the label, suggesting that the oxygen indeed stems from the hydroxy group of **1**. Additional information was obtained when we recovered unreacted starting material with an unchanged degree of incorporation, pointing to the fact that the carbon–oxygen bond is not affected during the reaction. Surprisingly, the reaction of **1** under the standard conditions in the presence of K_2_CO_3_ led to an oxidative fragmentation followed by skeletal rearrangement (Scheme 3, eq 3). A transformation employing similar reaction conditions and starting from the corresponding peroxide was previously reported by Stoltz^12^ and prompted us to investigate the formation of a peroxide as a possible intermediate. A positive control of peroxide involvement was achieved when **8** was subjected to the electrochemical conditions, yielding **4a** in 71% yield (Scheme 3, eq 4). Finally, the reaction was performed in the presence of BHT as a radical scavenger (Scheme 3, eq 5), affording a BHT-benzoxazinone adduct **9** as the exclusive product, suggesting the formation of a benzoxazinone benzylic radical.

On the basis of these results, a possible mechanism is described in Scheme 4A. Initially **1** could be oxidized at the anode to form the peroxide intermediate **I** that could rearrange to give a benzoxazinone benzylic radical **II***via* two possible pathways (Scheme 4B). A Baeyer–Villiger type rearrangement involving a concomitant cleavage of the C–C bond and liberation of an alkoxy radical could directly lead to ring enlargement, or the intramolecular formation on an epoxide could generate intermediate **II** through an oxa-Dowd–Beckwith-type rearrangement. It should be noted that the “Epoxide fragmentation” pathway could also be accessible from an oxygen-centered radical derived from **1**. Radical **II** is then proposed to undergo a second favorable anodic oxidation to form a highly stabilized benzylic carbocation **III**, which can be finally trapped by MeOH leading to product **2a**.

**Scheme 4:**
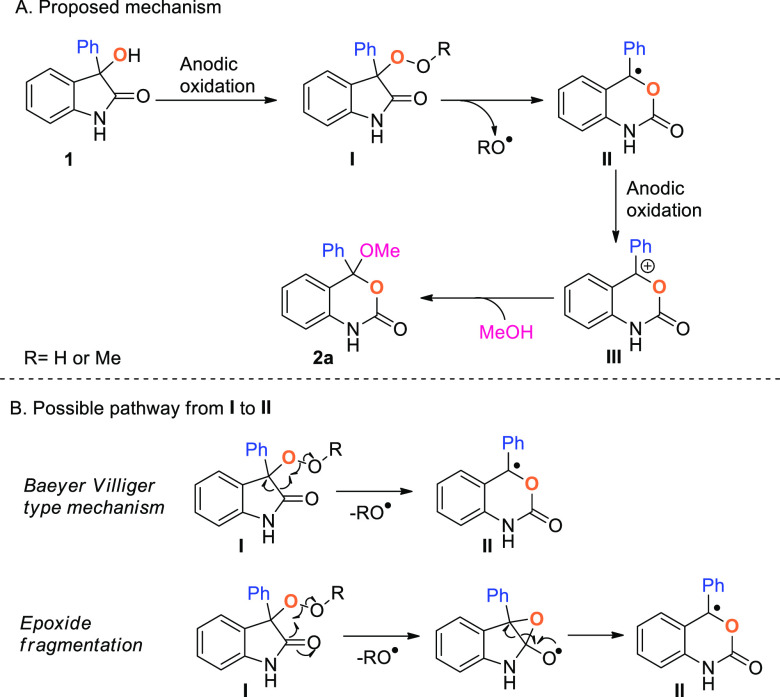
Proposed Mechanism

Under our standard conditions, in the presence of additional methylamine, **1** is converted into a 3,3-disubstituted quinazolinone derivative **10** in 64% yield (Scheme 5). This result, reminiscent of that obtained with K_2_CO_3_ (cf. Scheme 3, eq 3), can similarly be explained by the basic character of methylamine, enabling possible fragmentation of the peroxyoxindole (**IV**) to form an isocyanate intermediate (**V**). Subsequent addition of methylamine to form a urea (**VI**) followed by intramolecular nucleophilic collapse then accounts for the formation of **10**.

**Scheme 5:**
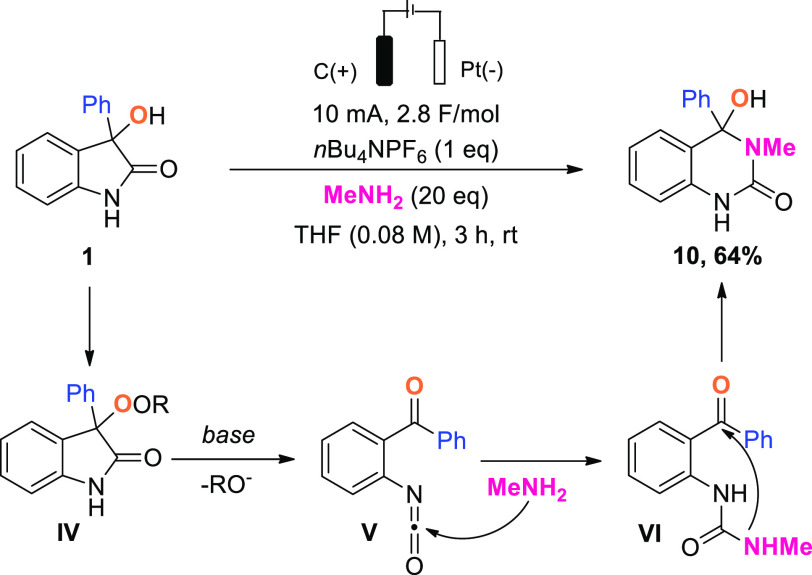
Reaction of 3-Hydroxyoxindole **1** with Methylamine

In conclusion, we have developed a practical strategy to access 3,1-benzoxazin-2-one derivatives by electrochemical skeletal reorganization of 3-hydroxy-2-oxindoles. The reaction boasts broad functional-group tolerance and experimental simplicity, being conducted in a setup open to air with nonanhydrous solvents and is a mechanistically intriguing process.

## Supporting Information Available

Additional optimization tables, experimental procedures, ^1^H and ^13^C NMR spectra, and characterization data of compounds (PDF)

## Accession Codes

CCDC 2102358 contains the supplementary crystallographic data for this paper. These data can be obtained free of charge via www.ccdc.cam.ac.uk/data_request/cif, or by emailing data_request@ccdc.cam.ac.uk, or by contacting The Cambridge Crystallographic Data Centre, 12 Union Road, Cambridge CB2 1EZ, UK; fax: +44 1223 336033.
