# Supplementary material for: Electrochemical Rearrangement of 3-Hydroxyoxindoles into Benzoxazinones
Source: Org Lett. 2021 Dec 24;24(1):27–32. doi: 10.1021/acs.orglett.1c03569 (PMC8762708; doi:10.1021/acs.orglett.1c03569)
Supplement: Supplementary file 1 — ol1c03569_si_001.pdf [file ol1c03569_si_001.pdf]

# Electrochemical Rearrangement of 3-Hydroxyoxindoles into Benzoxazinones

*Supporting Information*


**Marie Vayer,[†, ⁑,‡] Miryam Pastor,[†, ⁑,‡] Christiane Kofink[§] and Nuno Maulide*[,†,⁑]**

[†] *Institute of Organic Chemistry, University of Vienna, Währinger Strasse 38, 1090 Vienna, Austria,* [§] *Doktor-Boehringer-Gasse 5-11, 1120 Vienna, Austria, and* [⁑] *Christian-Doppler Laboratory for Entropy-Oriented Drug Design, Währinger Strasse 38, 1090 Vienna, Austria.*

[‡] These authors contributed equally.

*E-mail: nuno.maulide@univie.ac.at


## Table of Contents


**1 GENERAL REMARKS ..... S2**

**2 GENERAL PROCEDURE FOR ELECTROCHEMICAL FORMATION OF 3,1-BENZOXAZIN-2-ONES ..... S3**

*General procedure (A) to access 3,1-Benzoxazin-2-ones ..... S3*

**3 EXPERIMENTAL PROCEDURES AND CHARACTERIZATION DATA OF THE STARTING MATERIALS 1, 3A-L AND 5A-G ..... S3**

*General procedure (B) to access 3-hydroxysusbtituted oxindoles ..... S3*

**4 CHARACTERIZATION DATA OF 3,1-BENZOXAZIN-2-ONES 2A-G, 4A-L AND 6A-F ..... S13**

**5 MECHANISTIC EXPERIMENTS (SCHEME 3) ..... S24**

5.1 $^{18}$O LABELLED EXPERIMENTS ..... S24

*Synthesis of **1-$^{18}$O** ..... S24*

*Characterization data of **2a-$^{18}$O** ..... S26*

5.2 FORMATION OF METHYL (2-BENZOYLPHENYL)CARBAMATE 7 ..... S26

5.3 REACTION OF PEROXIDE 8 IN THE STANDARD CONDITIONS ..... S27

*Synthesis of 3-(tert-butylperoxy)-3-(4-methoxyphenyl)indolin-2-one **8** ..... S27*

5.4 BHT TRAPPING EXPERIMENT ..... S28

**6 X-RAY DIFFRACTION ANALYSIS OF COMPOUND 6B ..... S29**

**7 NMR SPECTRA ..... S32**

**8 REFERENCES ..... S85**

# 1 General Remarks

All solvents were distilled from appropriate drying agents prior to use or directly taken from commercial sealed bottles under an atmosphere of argon. All reagents were used as received from commercial suppliers (*Alfa Aesar*, *Sigma Aldrich* or *TCI*) unless otherwise stated. Reaction progress was monitored by thin layer chromatography (TLC) performed on aluminum plates coated with silica gel $F_{254}$ with 0.2 mm thickness. Chromatograms were visualized by fluorescence quenching with UV light at 254 nm and/or by staining using vanilin. Flash column chromatography was performed using silica gel 60 (230-400 mesh, Merck and co.). Yields refer to chromatographically and spectroscopically pure compounds. $^{1}$H NMR, $^{13}$C NMR and $^{18}$F NMR spectra were recorded using a Bruker AV-400, AV-600 and AV-700 spectrometer at 300K. $^{1}$H NMR chemical shifts are reported in ppm using residual solvent peak as reference ($CDCl_3$: $\delta$ = 7.26 ppm or DMSO-$d_6$: $\delta$ = 2.50 ppm). Data for $^{1}$H NMR are presented as follows: chemical shift $\delta$ (ppm), multiplicity (s = singlet, d = doublet, t = triplet, m = multiplet, br = broad), coupling constant $J$ (Hz) and integration; $^{13}$C NMR spectra were recorded at 100, 150 or 175 MHz using broadband proton decoupling and chemical shifts are reported in ppm using residual solvent peaks as reference ($CDCl_3$: $\delta$ = 77.16 ppm or DMSO-$d_6$: $\delta$ = 39.52 ppm). Multiplicity was defined by recorded a $^{13}$C NMR spectra using the attached proton test (APT). Neat infra-red spectra were recorded using a Brucker Vertex 70 FT-IR spectrometer. Wavenumbers are reported in $cm^{-1}$. Mass spectra were obtained using a Finnigan MAT 8200 or (70 eV) or an Agilent 5973 (70 eV) spectrometer, using electrospray ionization (ESI) and a maXis UHR-TOF analyzer.

**Electrolysis general information**

Electrochemical reactions were performed with ElectraSyn 2.0 package (IKA) using the constant current mode. The reactions were conducted in a 10 mL vial with a magnetic stir bar and a graphite-SK-50 (5.0 x 0.8 x 0.2 cm) working electrode and a platinum-plated (5.0 x 0.8 x 0.2 cm) counter-electrode with a distance of 0.6 cm between the two electrodes.

# 2 General Procedure for Electrochemical Formation of 3,1-Benzoxazin-2-ones

*General procedure (A) to access 3,1-Benzoxazin-2-ones*

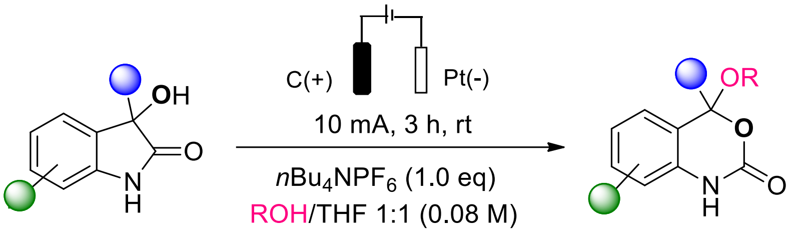


The ElectraSyn vial (10 mL) equipped with a stir bar was charged with 3-hydroxyoxindole derivatives **1**, **3a-l, 5a-f** (0.40 mmol, 1.0 equiv.), *n*$Bu_4NPF_6$ (155 mg, 0.40 mmol, 1.0 equiv.), ROH (2.5 mL) and THF (2.5 mL). The ElectraSyn vial cap equipped with anode (graphite) and cathode (platinum) was inserted into the mixture. The reaction mixture was electrolyzed at a constant current of 10 mA for 3 h. The ElectraSyn vial cap was removed, and electrodes were rinsed with $CH_2Cl_2$ (2.0 mL), which was combined with the crude mixture. Then, the crude mixture was concentrated under reduced pressure and purified by FC over silica gel (20 g $SiO_2$, heptane/EtOAc, 100/0 to 50/50, gradient) to furnish the desired **2a-g**, **4a-l, 6a-f**.

*Due to limitation of this specific electrochemical device, the maximum reaction limit is 0.4 mmol.*

# 3 Experimental Procedures and Characterization Data of the Starting Materials 1, 3a-l and 5a-g

*General procedure (B) to access 3-hydroxysusbtituted oxindoles* (**1**, **3a-l, 5a-g**)

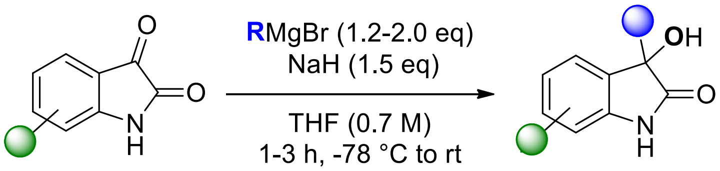


**1, 3a-l, 5a-g**

To a solution of the corresponding isatin derivate (1.0 equiv.) in THF (0.7 M), NaH (1.5 equiv.) was added at -78 °C. The mixture was stirred for 30 min. Then, the Grignard reagent (1.2-2.0 equiv.) was added dropwise at this temperature. The reaction was warmed up to rt and stirred until completion by TLC (1 to 3 hours generally). The mixture was then quenched with a saturated aq. solution $NH_4Cl$, extracted with EtOAc, washed with $H_2O$, dried over $MgSO_4$ and concentrated under reduced pressure. $CH_2Cl_2$ was added to the solids obtained. The insoluble part was filtered and dried in vacuo to afford the desired product. If necessary, crude products were purified by column chromatography over silica gel (40 g $SiO_2$, gradient, heptane/EtOAc, 100/0 to 50/50) to furnish the desired products.

***3-Hydroxy-3-phenylindolin-2-one* 1**

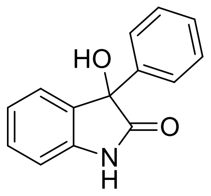

**1**

General procedure **B** was followed with isatin (2.0 g, 13.6 mmol) and phenylmagnesium bromide (3.0 M in $Et_2O$, 9.1 mL, 27.2 mmol, 2.0 equiv.). Purification was performed with flash column chromatography over silica gel (40 g $SiO_2$, heptane/EtOAc, 100/0 to 50/50, gradient) to afford **2a** as a yellow solid (1.2 g, 5.33 mmol, 39%). **$^1$H NMR (400 MHz, DMSO-d$_6$):** $\delta$ 10.40 (s, 1H), 7.33-7.23 (m, 6H), 7.10 (d, $J$ = 6.9 Hz, 1H), 6.96 (td, $J$ = 7.7, 1.1 Hz, 1H), 6.91 (d, $J$ = 7.7 Hz, 1H), 6.63 (s, 1H). **$^{13}$C NMR (100 MHz, DMSO-d$_6$):** $\delta$ 178.5 (C=O), 142.0 (C), 141.6 (C), 133.8 (C), 129.2 (CH), 128.1 (2CH), 127.4 (CH), 125.4 (2CH), 124.8 (CH), 122.1 (CH), 109.9 (CH), 77.3 (C). **FT-IR (neat, cm$^{-1}$):** 3408, 1703, 1615, 1467, 1360, 1338, 1180, 1156, 1119, 1067. **HRMS (ESI$^+$):** *m/z* calcd. for $C_{14}H_{11}NO_2Na$ $[M+Na]^+$ 248.0682, found 248.0670. The data are in agreement with those previously reported in the literature.[1]

***3-Hydroxy-3-(4-methoxyphenyl)indolin-2-one* 3a**

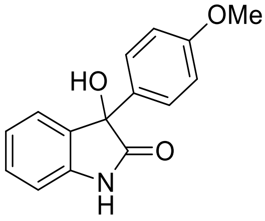

**3a**

General procedure **B** was followed with isatin (0.50 g, 3.40 mmol) and 4-methoxyphenylmagnesium bromide (0.50 M in THF, 9.5 mL, 4.76 mmol, 1.4 equiv.). Purification was performed with flash column chromatography over silica gel (40 g $SiO_2$, heptane/EtOAc, 100/0 to 50/50, gradient) to afford **3a** as a white solid (0.65 mg, 2.55 mmol, 75%). **$^1$H NMR (400 MHz, DMSO-d$_6$):** $\delta$ 10.32 (s, 1H), 7.24 (td, $J$ = 7.6, 1.1 Hz, 1H), 7.18 (d, $J$ = 8.8 Hz, 2H), 7.10 (d, $J$ = 7.2 Hz, 1H), 6.96 (t, $J$ = 7.6 Hz, 1H), 6.89-6.85 (m, 3H), 6.51 (s, 1H), 3.71 (s, 3H). **$^{13}$C NMR (100 MHz, DMSO-d$_6$):** $\delta$ 178.6 (C=O), 158.7 (C), 141.9 (C), 133.7 (C), 133.5 (C), 129.1 (CH), 126.8 (2CH), 124.8 (CH), 121.9 (CH), 113.4 (2CH), 109.8 (CH), 76.9 (C), 55.1 ($CH_3$). **FT-IR (neat, cm$^{-1}$):** 3270, 1718, 1684, 1610, 1509, 1466, 1347, 1297, 1250, 1177, 1123, 1106, 1076, 1033. **HRMS (ESI$^+$):** *m/z* calcd. for $C_{15}H_{13}NO_3Na$ $[M+Na]^+$ 278.0788, found 278.0775. The data are in agreement with those previously reported in the literature.[2]

***3-Hydroxy-3-(p-tolyl)indolin-2-one* 3b**

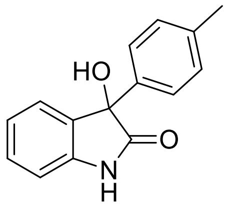

**3b**

General procedure **B** was followed with with isatin (0.50 g, 3.40 mmol) and *p*-tolylmagnesium bromide prepared from 4-bromotoluene (0.75 mL, 6.12 mmol, 1.8 equiv.). Purification was performed with flash column chromatography over silica gel (40 g $SiO_2$, heptane/EtOAc, 100/0 to 50/50, gradient) to afford **3b** as an off white solid (0.20 g, 0.83 mmol, 24%). **$^{1}$H NMR (600 MHz, $CDCl_3$):** $\delta$ 8.87 (brs, 1H), 7.24 (t, $J$ = 7.8 Hz, 1H), 7.16 (d, $J$ = 7.9 Hz, 2H), 7.13-7.10 (m, 3H), 7.02 (t, $J$ = 7.5 Hz, 1H), 6.93 (d, $J$ = 7.8 Hz, 1H), 4.60 (s, 1H), 2.34 (s, 3H). **$^{13}$C NMR (150 MHz, $CDCl_3$):** $\delta$ 179.1 (C=O), 141.8 (C), 137.5 (C), 133.6 (C), 130.0 (C), 129.8 (2CH), 128.5 (2CH), 128.4 (CH), 125.4 (CH), 122.8 (CH), 110.1 (CH), 52.5 (CH), 21.3 ($CH_3$). **HRMS (ESI$^+$):** *m/z* calcd. for $C_{15}H_{13}NO_2Na$ $[M+Na]^+$ 262.0838, found 262.0839. The data are in agreement with those previously reported in the literature.[2]

***3-(4-(Tert-butyl)phenyl)-3-hydroxyindolin-2-one* 3c**

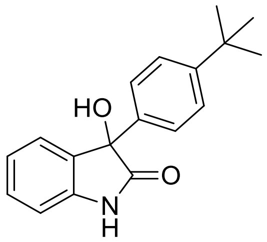

**3c**

General procedure **B** was followed with isatin (0.50 g, 3.40 mmol) and (4-(*tert*-butyl)phenyl)magnesium bromide prepared from 1-bromo-4-tert-butylbenzene (1.1 mL, 6.12 mmol, 1.8 equiv.). Purification was performed with flash column chromatography over silica gel (40 g $SiO_2$, heptane/EtOAc, 100/0 to 50/50, gradient) to afford **3c** as a yellow solid (0.59 g, 2.08 mmol, 61%). **$^{1}$H NMR (600 MHz, DMSO-$d_6$):** $\delta$ 10.36 (s, 1H), 7.32 (d, $J$ = 8.5 Hz, 2H), 7.24 (td, $J$ = 7.7, 0.8 Hz, 1H), 7.19 (d, $J$ = 8.5 Hz, 2H), 7.10 (d, $J$ = 7.4 Hz, 1H), 6.96 (t, $J$ = 7.4 Hz, 1H), 6.88 (d, $J$ = 7.7 Hz, 1H), 6.53 (s, 1H), 1.24 (s, 9H). **$^{13}$C NMR (150 MHz, DMSO-$d_6$):** $\delta$ 178.6 (C=O), 149.8 (C), 141.9 (C), 138.6 (C), 133.8 (C), 129.2 (CH), 125.2 (2CH), 124.9 (2CH), 124.8 (CH), 122.0 (CH), 109.8 (CH), 77.2 (C), 34.2 (C), 31.1 ($3CH_3$). **HRMS (ESI$^+$):** *m/z* calcd. for $C_{18}H_{19}NO_2Na$ $[M+Na]^+$ 304.1308, found 304.1306. The data are in agreement with those previously reported in the literature.[3]

***3-([1,1'-Biphenyl]-4-yl)-3-hydroxyindolin-2-one* 3d**

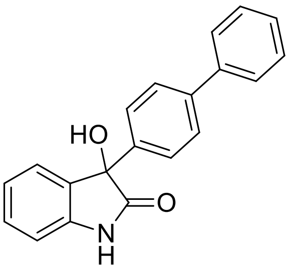


**3d**

General procedure **B** was followed with isatin (1.0 g, 6.80 mmol) and [1,1'-biphenyl]-4-ylmagnesium bromide prepared from 4-bromobiphenyl (3.4 mL, 13.6 mmol, 2.0 equiv.). Purification was performed with flash column chromatography over silica gel (40 g $SiO_2$, heptane/EtOAc, 100/0 to 50/50, gradient) to afford **3d** as a white solid (1.1 g, 3.54 mmol, 52%). **$^{1}$H NMR (600 MHz, DMSO-d$_6$):** *δ* 10.43 (s, 1H), 7.67-7.57 (m, 4H), 7.50-7.40 (m, 2H), 7.40-7.31 (m, 3H), 7.31-7.22 (m, 1H), 7.14 (d, *J* = 6.7 Hz, 1H), 7.03-6.87 (m, 2H), 6.68 (s, 1H). **$^{13}$C NMR (150 MHz, DMSO-d$_6$):** *δ* 178.4 (C=O), 142.0 (C), 140.7 (C), 139.9 (C), 139.4 (C), 133.7 (C), 129.3 (CH), 128.9 (2CH), 127.5 (CH), 126.7 (2CH), 126.5 (2CH), 126.1 (2CH), 124.8 (CH), 122.1 (CH), 109.9 (CH), 77.2 (C). **HRMS (ESI$^+$):** *m/z* calcd. for $C_{20}H_{15}NO_2Na$ $[M+Na]^+$ 324.0995, found 324.0997. The data are in agreement with those previously reported in the literature.[3]

***3-(4-Chlorophenyl)-3-hydroxyindolin-2-one* 3e**

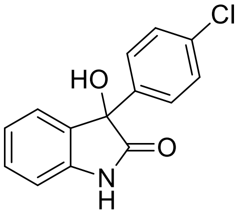


**3e**

General procedure **B** was followed with isatin (0.50 g, 3.40 mmol) and chlorophenylmagnesium bromide (1.0 M in THF, 6.8 mL, 6.80 mmol, 2.0 equiv.). Purification was performed with flash column chromatography over silica gel (40 g $SiO_2$, heptane/EtOAc, 100/0 to 50/50, gradient) to afford **3e** as a beige solid (0.20 g, 0.77 mmol, 23%). **$^{1}$H NMR (600 MHz, DMSO-d$_6$):** *δ* 10.45 (s, 1H), 7.32 (d, *J* = 8.5 Hz, 2H), 7.28-7.24 (m, 3H), 7.10 (d, *J* = 7.3 Hz, 1H), 6.98 (t, *J* = 7.5 Hz, 1H), 6.91 (d, *J* = 7.8 Hz, 1H), 6.74 (s, 1H). **$^{13}$C NMR (150 MHz, DMSO-d$_6$):** *δ* 178.1 (C=O), 142.0 (C), 140.5 (C), 133.2 (C), 132.2 (C), 129.5 (CH), 128.2 (2CH), 127.4 (2CH), 124.8 (CH), 122.2 (CH), 110.0 (CH), 76.9 (C). **FT-IR (neat, cm$^{-1}$):** 3189, 1714, 1620, 1489, 1471, 1396, 1181, 1102. **HRMS (ESI$^+$):** *m/z* calcd. for $C_{14}H_{10}ClNO_2Na$ $[M+Na]^+$ 282.0292, found 282.0293.

*3-(4-Fluorophenyl)-3-hydroxyindolin-2-one* **3f**

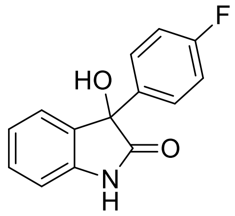


**3f**

General procedure **B** was followed with isatin (0.50 g, 3.40 mmol) and fluorophenylmagnesium bromide (1.0 M in THF, 6.1 mL, 6.12 mmol, 1.8 equiv.). Purification was performed with flash column chromatography over silica gel (40 g $SiO_2$, heptane/EtOAc, 100/0 to 50/50, gradient) to afford **3f** as a light yellow solid (0.45 g, 1.86 mmol, 55%). **$^{1}$H NMR (600 MHz, DMSO-d$_6$):** $\delta$ 10.42 (s, 1H), 7.32-7.27 (m, 2H), 7.26 (td, $J$ = 7.7, 1.2 Hz, 1H), 7.17-7.09 (m, 3H), 6.98 (td, $J$ = 7.5, 0.7 Hz, 1H), 6.90 (d, $J$ = 7.7 Hz, 1H), 6.69 (s, 1H). **$^{13}$C NMR (150 MHz, DMSO-d$_6$):** $\delta$ 178.3 (C=O), 161.6 (d, $J^{CF}$ = 243.5 Hz, C), 141.9 (C), 137.7 (d, $J^{CF}$ = 2.8 Hz, C), 133.4 (C), 129.4 (CH), 127.6 (d, $J^{CF}$ = 8.3 Hz, 2CH), 124.8 (CH), 122.2 (CH), 114.9 (d, $J^{CF}$ = 21.4 Hz, 2CH), 110.0 (CH), 76.8 (C). **$^{19}$F NMR (565 MHz, DMSO-d$_6$):** $\delta$ -115.2. **FT-IR (neat, cm$^{-1}$):** 3189, 1714, 1620, 1489, 1471, 1396, 1181, 1102. **HRMS (ESI$^+$):** *m/z* calcd. for $C_{14}H_{10}FNO_2Na$ [M+Na]$^+$ 266.0588, found 266.0591. All analytical data were in accordance with literature.[3]

*3-Hydroxy-3-(3-methoxyphenyl)indolin-2-one* **3g**

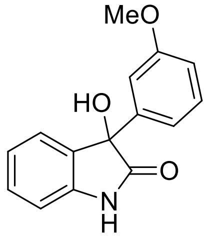


**3g**

General procedure **B** was followed with isatin (0.50 g, 3.40 mmol) and 3-methoxyphenylmagnesium bromide (1.0 M in 2-MeTHF, 4.8 mL, 4.76 mmol, 1.4 equiv.). Purification was performed with flash column chromatography over silica gel (40 g $SiO_2$, heptane/EtOAc, 100/0 to 50/50, gradient) to afford **3g** as a white solid (0.2 g, 0.78 mmol, 23%). **$^{1}$H NMR (400 MHz, DMSO-d$_6$):** $\delta$ 10.39 (s, 1H), 7.24 (td, $J$ = 7.7, 1.1 Hz, 1H), 7.19 (t, $J$ = 8.0 Hz, 1H), 7.09 (d, $J$ = 7.2 Hz, 1H), 6.96 (m, 2H), 6.89 (d, $J$ = 7.7 Hz, 1H), 6.83 (dd, $J$ = 8.0, 2.2 Hz, 1H), 6.69 (d, $J$ = 7.8 Hz, 1H), 6.62 (s, 1H), 3.72 (s, 3H). **$^{13}$C NMR (100 MHz, DMSO-d$_6$):** $\delta$ 178.3 (C=O), 159.1 (C), 143.1 (C), 141.9 (C), 133.7 (C), 129.2 (CH), 129.2 (CH), 124.7 (CH), 122.0 (CH), 117.5 (CH), 112.5 (CH), 111.5 (CH), 109.8 (CH), 77.2 (C), 55.0 ($CH_3$). **FT-IR (neat, cm$^{-1}$):** 3405, 1703, 1615, 1607, 1585, 1485, 1467, 1433, 1338, 1285, 1245, 1179, 1149, 1119, 1098, 1076, 1044. **HRMS (ESI$^+$):** *m/z* calcd. for $C_{15}H_{13}NO_3Na$ [M+Na]$^+$ 278.0788, found 278.0804. The data are in agreement with those previously reported in the literature.[4]

***3-Hydroxy-3-(perfluorophenyl)indolin-2-one* 3h**

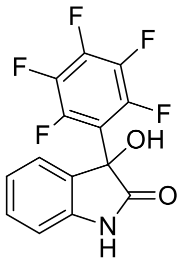

**3h**

General procedure **B** was followed with isatin (0.50 g, 2.04 mmol) and (perfluorophenyl)magnesium bromide prepared from bromopentafluorobenzene (1.2 M in $Et_2O$, 6.0 mL, 6.12 mmol, 1.8 equiv.). Purification was performed with flash column chromatography over silica gel (40 g $SiO_2$, heptane/EtOAc, 100/0 to 50/50, gradient) to afford **3h** as a yellow solid (0.95 g, 3.01 mmol, 89%). **$^1$H NMR (700 MHz, DMSO-d$_6$):** $\delta$ 10.67 (brs, 1H), 7.27 (td, $J$ = 7.7, 1.2 Hz, 1H), 7.24 (d, $J$ = 7.4 Hz, 1H), 7.17 (s, 1H), 6.97 (td, $J$ = 7.6, 0.9 Hz, 1H), 6.90 (d, J = 7.7 Hz, 1H). **$^{13}$C NMR (176 MHz, DMSO-d$_6$):** $\delta$ 175.6 (C=O), 144.5 (dm, $J$ = 251.6 Hz, 2C), 141.8 (C), 139.9 (dm, $J$ = 252.0 Hz, C), 137.3 (dm, $J$ = 248.3 Hz, 2C), 130.4 (CH), 130.4 (C), 124.8 (CH), 122.2 (CH), 115.6 (m, C), 110.2 (CH), 75.2 (C). **$^{19}$F NMR (659 MHz, DMSO-d$_6$):** $\delta$ 139.5 (d, $J$ = 19.9 Hz), -155.2 (t, $J$ = 22.4 Hz), -162.5 (dd, $J$ = 22.4, 19.9 Hz). **HRMS (ESI$^+$):** *m/z* calcd. for $C_{14}H_6F_5NO_2Na$ $[M+Na]^+$ 338.0211, found 338.0208.

***3-Hydroxy-3-methylindolin-2-one 3i***

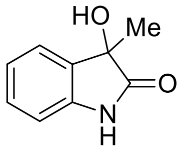

**3i**

General procedure **B** was followed with isatin (0.50 g, 3.40 mmol) and methylmagnesium bromide (3.0 M in $Et_2O$, 2.3 mL, 6.80 mmol, 2.0 equiv.). Purification was performed with flash column chromatography over silica gel (40 g $SiO_2$, heptane/EtOAc, 100/0 to 50/50, gradient) to afford **3i** as a light yellow solid (0.39 g, 2.39 mmol, 70%). **$^1$H NMR (600 MHz, DMSO-d$_6$):** 10.19 (s, 1H), 7.28 (d, $J$ = 7.3 Hz, 1H), 7.18 (t, $J$ = 7.6 Hz, 1H), 6.96 (t, $J$ = 7.4 Hz, 1H), 6.80 (d, $J$ = 7.7 Hz, 1H), 5.84 (s, 1H), 1.35 (s, 3H). **$^{13}$C NMR (150 MHz, DMSO-d$_6$):** $\delta$ 179.7 (C=O), 141.1 (C), 133.6 (C), 128.8 (CH), 123.4 (CH), 121.6 (CH), 109.6 (CH), 72.6 (C), 24.5 ($CH_3$). **HRMS (ESI$^+$):** *m/z* calcd. for $C_9H_9NO_2Na$ $[M+Na]^+$ 186.0525, found 186.0524. The data are in agreement with those previously reported in the literature.[5]

***3-Hydroxy-3-isopropylindolin-2-one* 3j**

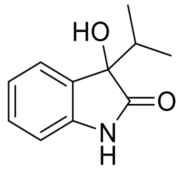

**3j**

General procedure **B** was followed with isatin (0.50 g, 3.40 mmol) and isopropylmagnesium chloride (2.0 M in THF, 2.4 mL, 4.76 mmol, 1.4 equiv.). Purification was performed with flash column chromatography over silica gel (40 g $SiO_2$, heptane/EtOAc, 100/0 to 50/50, gradient) to afford **3j** as a light yellow solid (70 mg, 0.37 mmol, 11%). **$^1$H NMR (400 MHz, DMSO-d$_6$):** $\delta$ 10.19 (s, 1H), 7.23-7.17 (m, 2H), 6.94 (td, $J$ = 7.6, 0.8 Hz, 1H), 6.79 (d, $J$ = 7.6 Hz, 1H), 5.76 (s, 1H), 2.11-2.01 (m, 1H), 0.96 (d, $J$ = 6.9 Hz, 3H), 0.63 (d, $J$ = 6.9 Hz, 3H). **$^{13}$C NMR (100 MHz, DMSO-d$_6$):** $\delta$ 179.5 (C=O), 142.2 (C), 130.6 (C), 128.7 (CH), 124.6 (CH), 121.3 (CH), 109.3 (CH), 78.3 (C), 34.8 (CH), 16.2 ($CH_3$), 15.8 ($CH_3$). **FT-IR (neat, cm$^{-1}$):** 3346, 1699, 1621, 1469, 1357, 1194, 1179, 1123, 1095, 1076. **HRMS (ESI$^+$):** *m/z* calcd. for $C_{11}H_{13}NO_2Na$ $[M+Na]^+$ 214.0838, found 214.0853. The data are in agreement with those previously reported in the literature.[6]

***3-Cyclopentyl-3-hydroxyindolin-2-one*** **3k**

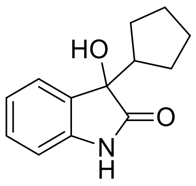


**3k**

General procedure **B** was followed with isatin (0.30 g, 2.04 mmol) and cyclopentylmagnesium bromide (2.0 M in THF, 1.4 mL, 2.85 mmol, 1.4 equiv.). Purification was performed with flash column chromatography over silica gel (40 g $SiO_2$, heptane/EtOAc, 100/0 to 50/50, gradient) to afford **3k** as a light orange solid (0.15 g, 0.69 mmol, 34%). **$^1$H NMR (500 MHz, $CDCl_3$):** $\delta$ 8.01 (brs, 1H), 7.40 (d, $J$ = 7.3 Hz, 1H), 7.25 (td, $J$ = 7.7, 1.2 Hz, 1H), 7.05 (t, $J$ = 7.3 Hz, 1H), 6.87 (d, $J$ = 7.7 Hz, 1H), 2.51-2.41 (m, 1H), 1.82-1.72 (m, 1H), 1.72-1.60 (m, 2H), 1.58-1.45 (m, 4H), 1.32-1.21 (m, 1H). **$^{13}$C NMR (126 MHz, $CDCl_3$):** $\delta$ 180.7 (C=O), 140.7 (C), 130.2 (C), 129.6 (CH), 125.1 (CH), 123.0 (CH), 110.2 (CH), 78.6 (C), 47.7 (CH), 26.6 ($CH_2$), 26.5 ($CH_2$), 25.7 ($CH_2$), 25.5 ($CH_2$). **HRMS (ESI$^+$):** *m/z* calcd. for $C_{13}H_{15}NO_2Na$ $[M+Na]^+$ 240.0995, found 240.0996.

***3-Allyl-3-hydroxyindolin-2-one 3l***

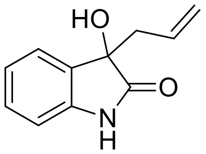


**3l**

General procedure **B** was followed with isatin (0.50 g, 3.40 mmol) and allylmagnesium bromide (1.0 M in $Et_2O$, 6.8 mL, 6.80 mmol, 2.0 equiv.). Purification was performed with flash column chromatography over silica gel (40 g $SiO_2$, heptane/EtOAc, 100/0 to 50/50, gradient) to afford **3k** as a light yellow solid (0.37 g, 1.94 mmol, 57%). **$^1$H NMR (400 MHz, $CDCl_3$):** $\delta$ 8.33 (brs, 1H), 7.37 (d, $J$ = 7.5 Hz, 1H), 7.26 (dt, $J$ = 8.8, 6.5 Hz, 1H), 7.07 (t, $J$ = 7.5 Hz, 1H), 6.88 (d, $J$ = 7.7 Hz, 1H), 5.73–5.61 (m, 1H), 5.15–5.08 (m, 2H), 3.36 (s, 1H), 2.75 (dd, $J$ = 13.4, 6.4 Hz, 1H), 2.62 (dd, $J$ = 13.4, 8.2 Hz, 1H). **$^{13}$C NMR (100 MHz, $CDCl_3$):** $\delta$ 180.3 (C=O), 140.4 (C), 130.4 (CH), 130.3 (C), 129.8 (CH), 124.6 (CH), 123.2 (CH), 120.7 ($CH_2$), 110.5 (CH), 76.4 (C), 43.0 ($CH_2$). **FT-IR (neat, cm$^{-1}$):** 3322, 3221, 3064,

2714, 1681, 1624, 1472, 1355, 1274, 1188, 1110, 1091. **HRMS (ESI$^+$):** *m/z* calcd. for $C_{11}H_{11}NO_2Na$ [M+Na]$^+$ 212.0682, found 212.0675.

***3-Hydroxy-5-methyl-3-phenylindolin-2-one*** **5a**

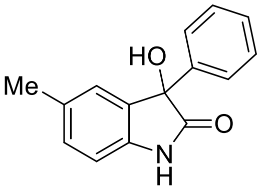


**5a**

General procedure **B** was followed with 5-methylisatin (0.50 g, 3.10 mmol) and phenylmagnesium bromide (3.0 M in $Et_2O$, 2.1 mL, 6.21 mmol, 2.0 equiv.). Purification was performed with flash column chromatography over silica gel (40 g $SiO_2$, heptane/EtOAc, 100/0 to 50/50, gradient) to afford **5a** as a white solid (0.46 g, 1.91 mmol, 61%). **$^1$H NMR (400 MHz, DMSO-d$_6$):** $\delta$ 10.30 (s, 1H), 7.33-7.23 (m, 5H), 7.04 (d, *J* = 7.8 Hz, 1H), 6.90 (s, 1H), 6.79 (d, *J* = 7.8 Hz, 1H), 6.57 (s, 1H), 2.21 (s, 3H). **$^{13}$C NMR (100 MHz, DMSO-d$_6$):** $\delta$ 178.5 (C=O), 141.7 (C), 139.4 (C), 133.9 (C), 130.9 (C), 129.4 (CH), 128.1 (2CH), 127.3 (CH), 125.4 (2CH), 125.3 (CH), 109.6 (CH), 77.4 (C), 20.60 ($CH_3$). **FT-IR (neat, cm$^{-1}$):** 3209, 1697, 1624, 1491, 1202, 1190, 1149, 1139, 1128. **HRMS (ESI$^+$):** *m/z* calcd. for $C_{15}H_{13}NO_2Na$ [M+Na]$^+$ 262.0838, found 262.0853. The data are in agreement with those previously reported in the literature.[2]

***5-Bromo-3-hydroxy-3-phenylindolin-2-one*** **5b**

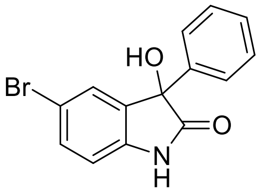


**5b**

General procedure **B** was followed with 5-bromoisatin (0.32 g, 1.39 mmol) and phenylmagnesium bromide (3.0 M in $Et_2O$, 0.93 mL, 2.79 mmol, 2.0 equiv.). Purification was performed with flash column chromatography over silica gel (40 g $SiO_2$, heptane/EtOAc, 100/0 to 50/50, gradient) to afford **5b** as a light yellow solid (0.30 mg, 0.98 mmol, 71%). **$^1$H NMR (600 MHz, DMSO-d$_6$):** $\delta$ 10.57 (brs, 1H), 7.44 (dd, *J* = 8.3, 2.1 Hz, 1H), 7.36-7.31 (m, 2H), 7.30-7.26 (m, 3H), 7.20 (d, *J* = 2.1 Hz, 1H), 6.88 (d, *J* = 8.3 Hz, 1H), 6.79 (s, 1H). **$^{13}$C NMR (150 MHz, DMSO-d$_6$):** $\delta$ 178.0 (C=O), 141.3 (C), 140.8 (C), 136.2 (C), 132.0 (CH), 128.3 (2CH), 127.7 (CH), 127.4 (CH), 125.3 (2CH), 113.7 (C), 112.1 (CH), 77.4 (C). **HRMS (ESI$^+$):** *m/z* calcd. for $C_{14}H_{10}BrNO_2Na$ [M+Na]$^+$ 325.9787, found 325.9786. The data are in agreement with those previously reported in the literature.[3]

***3-Hydroxy-6-methoxy-3-phenylindolin-2-one* 5d**

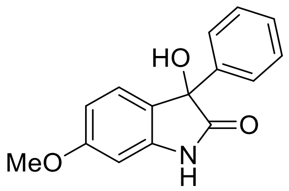


**5d**

General procedure **B** was followed with 6-methoxylisatin (0.30 g, 1.69 mmol) and phenylmagnesium bromide (3.0 M in $Et_2O$, 0.84 mL, 1.49 mmol, 1.5 equiv.). Purification was performed with flash column chromatography over silica gel (40 g $SiO_2$, heptane/EtOAc, 100/0 to 50/50, gradient) to afford **5d** as a light yellow solid (0.13 g, 0.51 mmol, 30%). **$^1$H NMR (600 MHz, DMSO)** $\delta$ 10.34 (brs, 1H), 7.32-7.28 (m, 2H), 7.28-7.22 (m, 3H), 6.99 (d, *J* = 8.2 Hz, 1H), 6.51 (dd, *J* = 8.2, 2.3 Hz, 1H), 6.49 (brs, 1H), 6.45 (d, *J* = 2.3 Hz, 1H), 3.75 (s, 3H). **$^{13}$C NMR (150 MHz, DMSO)** $\delta$ 178.9 (C=O), 160.3 (C), 143.3 (C), 141.9 (C), 128.0 (2CH), 127.3 (CH), 125.7 (CH), 125.7 (C), 125.5 (2CH), 106.8 (CH), 96.6 (CH), 77.0 (C), 55.3 ($CH_3$). **HRMS (ESI$^+$):** *m/z* calcd. for $C_{15}H_{13}NO_3Na$ $[M+Na]^+$ 278.0788, found 278.0790. The data are in agreement with those previously reported in the literature.[7]

***3-Hydroxy-7-methyl-3-phenylindolin-2-one* 5e**

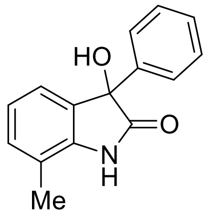


**5e**

General procedure **B** was followed with 7-methylisatin (0.20 g, 1.24 mmol) and phenylmagnesium bromide (3.0 M in $Et_2O$, 0.50 mL, 1.49 mmol, 1.2 equiv.). Purification was performed with flash column chromatography over silica gel (40 g $SiO_2$, heptane/EtOAc, 100/0 to 50/50, gradient) to afford **5e** as a light yellow solid (0.12 g, 0.54 mmol, 44%). **$^1$H NMR (400 MHz, DMSO-d$_6$):** $\delta$ 10.43 (s, 1H), 7.33-7.22 (m 5H), 7.07 (d, *J* = 7.0 Hz, 1H), 6.93-6.84 (m, 2H), 6.57 (s, 1H), 2.25 (s, 3H). **$^{13}$C NMR (100 MHz, DMSO-d$_6$):** $\delta$ 178.9 (C=O), 141.7 (C), 140.4 (C), 133.4 (C), 130.4 (CH), 128.0 (2CH), 127.3 (CH), 125.4 (2CH), 122.1 (CH), 122.0 (CH), 119.2 (C), 77.5 (C), 16.4 ($CH_3$). **FT-IR (neat, cm$^{-1}$):** 3165, 1720, 1626, 1606, 1486, 1448, 1381, 1300, 1209, 1191, 1163, 1118, 1102, 1070. **HRMS (ESI$^+$):** *m/z* calcd. for $C_{15}H_{13}NO_2Na$ $[M+Na]^+$ 262.0838, found 262.0852. The data are in agreement with those previously reported in the literature.[7]

***3-Hydroxy-3-phenyl-7-(trifluoromethyl)indolin-2-one* 5f**

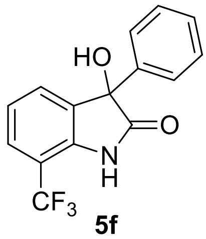


**5f**

General procedure **B** was followed with 7-(trifluoromethyl)isatin (0.30 g, 1.39 mmol) and phenylmagnesium bromide (3.0 M in $Et_2O$, 0.93 mL, 2.79 mmol, 2.0 equiv.). Purification was performed with flash column chromatography over silica gel (40 g $SiO_2$, heptane/EtOAc, 100/0 to 50/50, gradient) to afford **5f** as a light yellow solid (0.20 g, 0.70 mmol, 50%). **$^1$H NMR (600 MHz, DMSO-d$_6$):** $\delta$ 10.92 (brs, 1H), 7.56 (d, $J$ = 8.0 Hz, 1H), 7.38 (d, $J$ = 7.3 Hz, 1H), 7.36-7.31 (m, 2H), 7.31-7.24 (m, 3H), 7.16 (t, $J$ = 7.7 Hz, 1H), 6.85 (s, 1H). **$^{13}$C NMR (150 MHz, CDCl$_3$):** 178.8 (C=O), 140.6 (C), 139.4 (q, $J^{CF}$ = 2.2 Hz, C), 135.6 (C), 128.9 (CH), 128.3 (2CH), 127.8 (CH), 125.7 (q, $J^{CF}$ = 4.6 Hz, CH), 125.4 (2CH), 123.6 (q, $J^{CF}$ = 271.8 Hz, C), 122.4 (CH), 111.0 (q, $J^{CF}$ = 32.8 Hz, C), 76.0 (C). **$^{19}$F NMR (471 MHz, CDCl$_3$):** $\delta$ -59.9. **FT-IR (neat, cm$^{-1}$):** 3219, 2921, 2851, 2360, 2342, 1720, 1614, 1443, 1317, 1198. **HRMS (ESI$^+$):** *m/z* calcd. for $C_{15}H_{10}F_3NO_2Na$ $[M+Na]^+$ 316.0556, found 316.0556.

# 4 Characterization data of 3,1-Benzoxazin-2-ones 2a-g, 4a-l and 6a-f

*4-Methoxy-4-phenyl-1H-benzo[d][1,3]oxazin-2(4H)-one* **2a**

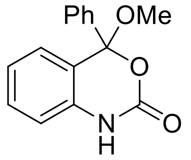


**2a**

General procedure **A** was followed with **1** (90.1 mg, 0.40 mmol) and MeOH (2.5 mL). Purification was performed with flash column chromatography over silica gel (20 g $SiO_2$, heptane/EtOAc, 100/0 to 50/50, gradient) to afford **2a** as a white solid (92.7 mg, 0.36 mmol, 91%). **$^1$H NMR (400 MHz, $CDCl_3$):** $\delta$ 9.01 (brs, 1H), 7.55-7.51 (m, 2H), 7.45-7.38 (m, 3H), 7.34 (ddd, $J$ = 8.4, 7.4, 1.8 Hz, 1H), 7.08-7.01 (m, 2H), 6.95 (d, $J$ = 8.4 Hz, 1H), 3.44 (s, 3H). **$^{13}$C NMR (100 MHz, $CDCl_3$):** $\delta$ 151.6 (C=O), 138.6 (C), 135.1 (C), 130.6 (CH), 129.3 (CH), 128.6 (2CH), 126.9 (3CH), 123.7 (CH), 121.0 (C), 114.8 (CH), 107.5 (C), 52.0 ($CH_3$). **FT-IR (neat, cm$^{-1}$):** 1709, 1603, 1492, 1449, 1434, 1346, 1256, 1215, 1177, 1093, 1076, 1007. **HRMS (ESI$^+$):** *m/z* calcd. for $C_{15}H_{13}NO_3Na$ $[M+Na]^+$ 278.0788, found 278.0789.

*4-Ethoxy-4-phenyl-1H-benzo[d][1,3]oxazin-2(4H)-one* **2b**

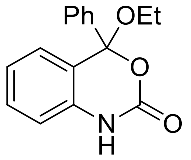


**2b**

General procedure **A** was followed with **1** (90.1 mg, 0.40 mmol) and EtOH (2.5 mL). Purification was performed with flash column chromatography over silica gel (20 g $SiO_2$, heptane/EtOAc, 100/0 to 50/50, gradient) to afford **2b** as a white solid (83.3 mg, 0.31 mmol, 77%). **$^1$H NMR (400 MHz, $CDCl_3$):** $\delta$ 9.73 (brs, 1H), 7.59-7.52 (m, 2H), 7.44-7.37 (m, 3H), 7.32 (ddd, $J$ = 8.4, 7.4, 1.8 Hz, 1H), 7.08-7.02 (m, 2H), 6.99 (d, $J$ = 8.4 Hz, 1H), 3.71 (qd, $J$ = 7.1, 2.2 Hz, 2H), 1.28 (t, $J$ = 7.1 Hz, 3H). **$^{13}$C NMR (100 MHz, $CDCl_3$):** $\delta$ 152.3 (C=O), 139.3 (C), 136.0 (C), 130.4 (CH), 129.2 (CH), 128.5 (2CH), 126.8 (2CH), 126.8 (CH), 123.6 (CH), 121.3 (C), 114.9 (CH), 107.5 (C), 60.5 ($CH_2$), 15.4 ($CH_3$). **FT-IR (neat, cm$^{-1}$):** 1710, 1601, 1490, 1449, 1342, 1255, 1215, 1135, 1095, 1076, 1026, 1002. **HRMS (ESI$^+$):** *m/z* calcd. for $C_{16}H_{15}NO_3Na$ $[M+Na]^+$ 292.0944, found 292.0941.

***4-Phenyl-4-propoxy-1H-benzo[d][1,3]oxazin-2(4H)-one* 2c**

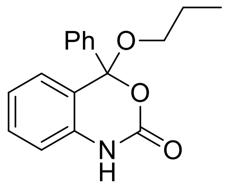

**2c**

General procedure **A** was followed with **1** (90.1 mg, 0.40 mmol) and *n*PrOH (2.5 mL). Purification was performed with flash column chromatography over silica gel (20 g $SiO_2$, heptane/EtOAc, 100/0 to 50/50, gradient) to afford **2c** as a white solid (89.3 mg, 0.32 mmol, 79%). **$^1$H NMR (400 MHz, $CDCl_3$):** $\delta$ 9.72 (brs, 1H), 7.55-7.52 (m, 2H), 7.43-7.36 (m, 3H), 7.30 (ddd, $J$ = 8.4, 7.4, 1.8 Hz, 1H), 7.08-7.00 (m, 2H), 6.99 (d, $J$ = 8.4 Hz, 1H), 3.58 (qt, $J$ = 8.8, 7.4 Hz, 2H), 1.73-1.63 (m, 2H), 0.95 (t, $J$ = 7.4 Hz, 3H). **$^{13}$C NMR (100 MHz, $CDCl_3$):** $\delta$ 152.2 (C=O), 139.6 (C), 136.1 (C), 130.4 (CH), 129.2 (CH), 128.5 (2CH), 126.9 (CH), 126.8 (2CH), 123.6 (CH), 121.2 (C), 114.9 (CH), 107.4 (C), 66.2 ($CH_2$), 23.1 ($CH_2$), 10.7 ($CH_3$). **FT-IR (neat, cm$^{-1}$):** 1708, 1601, 1491, 1449, 1434, 1341, 1254, 1216, 1180, 1133, 1094, 1077, 1036, 1002. **HRMS (ESI$^+$):** *m/z* calcd. for $C_{17}H_{17}NO_3Na$ $[M+Na]^+$ 306.1101, found 306.1102.

***4-(Benzyloxy)-4-phenyl-1H-benzo[d][1,3]oxazin-2(4H)-one* 2d**

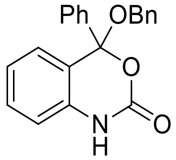

**2d**

The general procedure **A** was followed with **1** (90.1 mg, 0.40 mmol) and BzOH (2.5 mL). Purification was performed with flash column chromatography over silica gel (20 g $SiO_2$, heptane/EtOAc, 100/0 to 50/50, gradient) to afford **2d** as a white solid (64.6 mg, 0.12 mmol, 49%). **$^1$H NMR (400 MHz, $CDCl_3$):** $\delta$ 9.77 (brs, 1H), 7.54-7.47 (m, 2H), 7.36-7.29 (m, 3H), 7.28-7.10 (m, 6H), 7.04 (d, $J$ = 7.5 Hz, 1H), 6.98-6.87 (m, 2H), 4.62 (d, $J$ = 2.7 Hz, 2H). **$^{13}$C NMR (100 MHz, $CDCl_3$):** $\delta$ 152.1 (C=O), 139.1 (C), 137.1 (C), 135.1 (C), 130.6 (CH), 129.4 (CH), 128.6 (2CH), 128.5 (2CH), 127.9 (3CH), 126.9 (3CH), 123.7 (CH), 120.9 (C), 115.0 (CH), 107.5 (C), 66.6 ($CH_2$). **HRMS (ESI$^+$):** *m/z* calcd. for $C_{21}H_{17}NO_3Na$ $[M+Na]^+$ 354.1101, found 354.1101.

***4-Phenethoxy-4-phenyl-1H-benzo[d][1,3]oxazin-2(4H)-one* 2e**

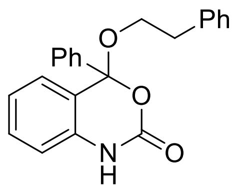

**2e**

General procedure **A** was followed with **1** (90.1 mg, 0.40 mmol) and 2-phenylethanol (2.5 mL). Purification was performed with flash column chromatography over silica gel (20 g $SiO_2$, heptane/EtOAc, 100/0 to 50/50, gradient) to afford **2e** as a white solid (46.2 mg, 0.14 mmol, 33%). **$^1$H NMR (400 MHz, $CDCl_3$):** *δ* 9.24 (brs, 1H), 7.39-7.34 (m, 2H), 7.31-7.27 (m, 3H), 7.24-7.17 (m, 3H), 7.14-7.09 (m, 3H), 6.91 (ddd, *J* = 8.4, 7.4, 1.8 Hz, 1H), 6.88-6.84 (m, 2H), 3.82-3.72 (m, 2H), 2.90 (t, *J* = 7.1 Hz, 2H). **$^{13}$C NMR (100 MHz, $CDCl_3$):** *δ* 151.8 (C=O), 139.3 (C), 138.5 (C), 136.0 (C), 130.4 (CH), 129.2 (2CH), 129.2 (CH), 128.6 (2CH), 128.5 (2CH), 126.8 (CH), 126.8 (2CH), 126.5 (CH), 123.6 (CH), 121.0 (C), 114.8 (CH), 107.4 (C), 66.5 ($CH_2$), 36.4 ($CH_2$). **HRMS (ESI$^+$):** *m/z* calcd. for $C_{22}H_{19}NO_3Na$ $[M+Na]^+$ 368.1257, found 368.1256.

***4-(Allyloxy)-4-phenyl-1H-benzo[d][1,3]oxazin-2(4H)-one* 2f**

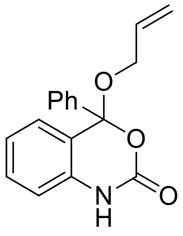


**2f**

General procedure **A** was followed with **1** (90.1 mg, 0.40 mmol) and allyl alcohol (2.5 mL). Purification was performed with flash column chromatography over silica gel (20 g $SiO_2$, heptane/EtOAc, 100/0 to 50/50, gradient) to afford **2f** as a white solid (86.6 mg, 0.31 mmol, 77%). **$^1$H NMR (400 MHz, $CDCl_3$):** *δ* 9.31 (brs, 1H), 7.57-7.53 (m, 2H), 7.44-7.38 (m, 3H), 7.31 (ddd, *J* = 8.4, 7.4, 1.8 Hz, 1H), 7.08-7.00 (m, 2H), 6.96 (d, *J* = 8.4 Hz, 1H), 5.96 (ddt, *J* = 17.2, 10.5, 5.5 Hz, 1H), 5.35-5.29 (m, 1H), 5.21-5.16 (m, 1H), 4.18 (ddd, *J* = 5.5, 1.7, 1.6 Hz, 2H). **$^{13}$C NMR (100 MHz, $CDCl_3$):** *δ* 151.8 (C=O), 138.9 (C), 136.0 (C), 133.7 (CH), 130.5 (CH), 129.3 (CH), 128.6 (2CH), 126.9 (3CH), 123.7 (CH), 121.2 (C), 117.5 ($CH_2$), 114.9 (CH), 107.4 (C), 66.9 ($CH_2$). **FT-IR (neat, cm$^{-1}$):** 1709, 1602, 1491, 1449, 1347, 1256, 1213, 1133, 1105, 1090, 1074, 1036, 1005. **HRMS (ESI$^+$):** *m/z* calcd. for $C_{17}H_{15}NO_3Na$ $[M+Na]^+$ 304.0944, found 304.0942.

***4-Isopropoxy-4-phenyl-1H-benzo[d][1,3]oxazin-2(4H)-one* 2g**

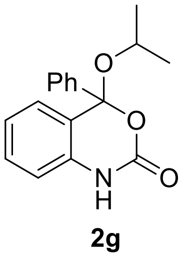


**2g**

General procedure **A** was followed with **1** (90.1 mg, 0.40 mmol) and *i*PrOH (2.5 mL). Purification was performed with flash column chromatography over silica gel (20 g $SiO_2$, heptane/EtOAc, 100/0 to 50/50, gradient) to afford **2g** as a brown solid (57.8 mg, 0.20 mmol, 51%). **$^1$H NMR (400 MHz, $CDCl_3$):** *δ* 9.79 (brs, 1H), 7.57-7.50 (m, 2H), 7.42-7.35 (m, 3H), 7.29 (ddd, *J* = 7.6, 7.6, 1.4 Hz, 1H), 7.15 (dd, *J* = 7.6, 1.4 Hz, 1H), 7.02 (ddd, *J* = 7.6, 7.6, 1.1

Hz, 1H), 6.98 (dd, $J$ = 7.6, 1.1 Hz, 1H) 4.12-4.02 (m, 1H), 1.30 (d, $J$ = 6.1 Hz, 3H), 1.23 (d, $J$ = 6.1 Hz, 3H). **$^{13}$C NMR (100 MHz, $CDCl_3$):** $\delta$ 152.3 (C=O), 140.2 (C), 135.0 (C), 130.4 (CH), 129.1 (CH), 128.4 (2CH), 127.1 (CH), 126.9 (2CH), 123.4 (CH), 121.4 (C), 114.9 (CH), 108.3 (C), 69.2 (CH), 24.2 ($2CH_3$). **FT-IR (neat, cm$^{-1}$):** 1707, 1599, 1494, 1361, 1258, 1202, 1068, 1012. **HRMS (ESI$^+$):** *m/z* calcd. for $C_{17}H_{17}NO_3Na$ $[M+Na]^+$ 306.1101, found 306.1105.

***4-Methoxy-4-(4-methoxyphenyl)-1H-benzo[d][1,3]oxazin-2(4H)-one* 4a**

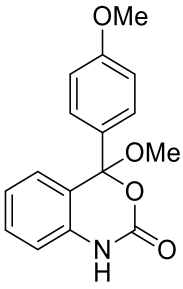


**4a**

The general procedure **A** was followed with **3a** (102 mg, 0.40 mmol) and MeOH (2.5 mL). Purification was performed with flash column chromatography over silica gel (20 g $SiO_2$, heptane/EtOAc, 100/0 to 50/50, gradient) to afford **4a** as a light brown solid (98.7 mg, 0.35 mmol, 87%). **$^1$H NMR (400 MHz, $CDCl_3$):** $\delta$ 8.79 (brs, 1H), 7.43 (d, $J$ = 8.9 Hz, 2H), 7.31 (ddd, $J$ = 8.0, 7.0, 1.9 Hz, 1H), 7.10-7.00 (m, 2H), 6.94-6.90 (m, 3H), 3.82 (s, 3H), 3.43 (s, 3H). **$^{13}$C NMR (100 MHz, $CDCl_3$):** $\delta$ 160.3 (C=O), 151.6 (C), 136.1 (C), 130.9 (C), 130.5 (CH), 128.4 (2CH), 126.9 (CH), 123.6 (CH), 121.3 (C), 114.7 (CH), 113.9 (2CH), 107.6 (C), 55.5 ($CH_3$), 52.0 ($CH_3$). **FT-IR (neat, cm$^{-1}$):** 1700, 1604, 1513, 1505, 1494, 1432, 1353, 1319, 1305, 1251, 1209, 1185, 1169, 1153, 1095, 1032, 1009. **HRMS (ESI$^+$):** *m/z* calcd. for $C_{16}H_{15}NO_4Na$ $[M+Na]^+$ 308.0893, found 308.0887.

***4-Methoxy-4-(p-tolyl)-1H-benzo[d][1,3]oxazin-2(4H)-one* 4b**

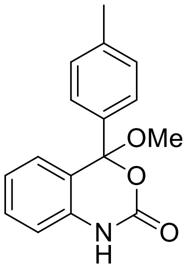


**4b**

General procedure **A** was followed with **3b** (95.7 mg, 0.40 mmol) and MeOH (2.5 mL) Purification was performed with flash column chromatography over silica gel (20 g $SiO_2$, heptane/EtOAc, 100/0 to 50/50, gradient) to afford **4b** as a light yellow solid (99.0 mg, 0.37 mmol, 92%). **$^1$H NMR (400 MHz, $CDCl_3$):** $\delta$ 9.66 (brs, 1H), 7.40 (d, $J$ = 8.3 Hz, 2H), 7.30 (td, $J$ = 8.0, 1.5 Hz, 1H), 7.22 (d, $J$ = 8.0 Hz, 2H), 7.06 (dd, $J$ = 7.7, 1.5 Hz, 1H), 7.02 (td, $J$ = 7.7, 1.0 Hz, 1H), 6.99 (dd, $J$ = 8.0, 1.0 Hz, 1H), 3.43 (s, 3H), 2.37 (s, 3H). **$^{13}$C NMR**

**(150 MHz, CDCl$_3$):** $\delta$ 152.3 (C=O), 139.3 (C), 135.9 (C), 135.2 (C), 130.5 (CH), 129.3 (2CH), 126.9 (2CH), 126.8 (CH), 123.7 (CH), 121.1 (C), 115.0 (CH), 107.7 (C), 52.0 ($CH_3$), 21.4 ($CH_3$). **FT-IR (neat, cm$^{-1}$):** 3096, 3004, 2924, 2360, 2342, 1714, 1603, 1493, 1345, 1255, 1182. **HRMS (ESI$^+$):** *m/z* calcd. for $C_{16}H_{15}NO_3Na$ $[M+Na]^+$ 292.0944, found 292.0943.

***4-(4-(Tert-butyl)phenyl)-4-methoxy-1H-benzo[d][1,3]oxazin-2(4H)-one* 4c**

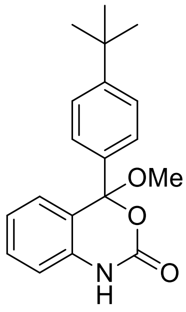


**4c**

The general procedure **A** was followed with **3c** (113 mg, 0.40 mmol) and MeOH (2.5 mL). Purification was performed with flash column chromatography over silica gel (20 g $SiO_2$, heptane/EtOAc, 100/0 to 50/50, gradient) to afford **4c** as a light yellow solid (89.9 mg, 0.29 mmol, 72%). **$^1$H NMR (600 MHz, DMSO-d$_6$):** $\delta$ 10.62 (brs, 1H), 7.46 (d, $J$ = 8.5 Hz, 2H), 7.36-7.29 (m, 3H), 7.02 (t, $J$ = 7.5 Hz, 1H), 7.00-6.95 (m, 2H), 3.25 (s, 3H), 1.27 (s, 9H). **$^{13}$C NMR (150 MHz, DMSO-d$_6$):** $\delta$ 151.5 (C=O), 149.3 (C), 136.7 (C), 135.8 (C), 130.3 (CH), 126.3 (CH), 125.9 (2CH), 125.3 (2CH), 122.7 (CH), 120.1 (C), 114.4 (CH), 106.0 (C), 51.0 ($CH_3$), 34.4 (C), 31.0 ($3CH_3$). **FT-IR (neat, cm$^{-1}$):** 3100, 2950, 2359, 2342, 1720, 1602, 1494, 1347, 1275, 1263, 1258. **HRMS (ESI$^+$):** *m/z* calcd. for $C_{19}H_{22}NO_3$ $[M+H]^+$ 312.1594, found 312.1595.

***4-([1,1'-Biphenyl]-4-yl)-4-methoxy-1H-benzo[d][1,3]oxazin-2(4H)-one* 4d**

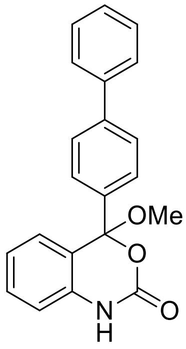


**4d**

The general procedure **A** was followed was followed with **3d** (121 mg, 0.40 mmol) and MeOH (2.5 mL). Purification was performed with flash column chromatography over silica gel (20 g $SiO_2$, heptane/EtOAc, 100/0 to 50/50, gradient) to afford **4d** as a light yellow solid (91.1 mg, 0.28 mmol, 69%).**$^1$H NMR (600 MHz, DMSO-d$_6$):** $\delta$ 10.69 (brs, 1H), 7.74 (d, $J$ = 8.4 Hz, 2H), 7.68 (d, $J$ = 7.3 Hz, 2H), 7.50 (d, $J$ = 8.4 Hz, 2H), 7.48 (t, $J$ = 7.7 Hz, 2H), 7.39 (t, $J$ = 7.3 Hz, 1H), 7.35 (ddd, $J$ = 8.6, 6.1, 2.8 Hz, 1H), 7.08-7.02 (m, 2H), 7.00 (d, $J$ = 8.1

Hz, 1H), 3.29 (s, 3H). **$^{13}$C NMR (150 MHz, DMSO-d$_6$):** $\delta$ 149.2 (C=O), 140.8 (C), 139.3 (C), 138.6 (C), 135.8 (C), 130.4 (CH), 129.0 (2CH), 127.8 (CH), 126.8 (2CH), 126.8 (2CH), 126.8 (2CH), 126.3 (CH), 122.8 (CH), 119.9 (C), 114.5 (CH), 105.9 (C), 51.0 ($CH_3$). **FT-IR (neat, cm$^{-1}$):** 3006, 2989, 2360, 2342, 1716, 1603, 1492, 1343, 1275, 1258, 1216, 1186. **HRMS (ESI$^+$):** *m/z* calcd. for $C_{21}H_{17}NO_3Na$ [M+Na]$^+$ 354.1101, found 354.1097.

***4-(4-Chlorophenyl)-4-methoxy-1H-benzo[d][1,3]oxazin-2(4H)-one* 4e**

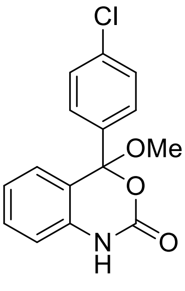


**4e**

General procedure **A** was followed with **3e** (104 mg, 0.40 mmol) and MeOH (2.5 mL). Purification was performed with flash column chromatography over silica gel (20 g $SiO_2$, heptane/EtOAc, 100/0 to 50/50, gradient) to afford **4e** as a light yellow solid (60.0 mg, 0.21 mmol, 52%). **$^1$H NMR (600 MHz, CDCl$_3$):** $\delta$ 9.66 (s, 1H), 7.46 (d, *J* = 8.6 Hz, 2H), 7.38 (d, *J* = 8.6 Hz, 2H), 7.33 (td, *J* = 7.6, 1.5 Hz, 1H), 7.07–7.03 (m, 1H), 7.03-6.97 (m, 2H), 3.42 (s, 3H). **$^{13}$C NMR (150 MHz, CDCl$_3$):** $\delta$ 151.8 (C=O), 137.5 (C), 135.4 (C), 135.1 (C), 130.8 (CH), 128.8 (2CH), 128.4 (2CH), 126.8 (CH), 123.8 (CH), 120.1 (C), 115.0 (CH), 107.1 (C), 52.0 ($CH_3$). **FT-IR (neat, cm$^{-1}$):** 3093, 2989, 2925, 2359, 2342, 1703, 1601, 1491, 1343, 1267, 1253, 1219. **HRMS (ESI$^+$):** *m/z* calcd. for $C_{15}H_{12}ClNO_3Na$ [M+Na]$^+$ 312.0398, found 312.0398.

***4-(4-Fluoro-phenyl)-4-methoxy-1,4-dihydro-benzo[d][1,3]oxazin-2-one* 4f**

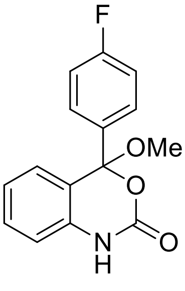


**4f**

General procedure **A** was followed with **3f** (97.3 mg, 0.40 mmol) and MeOH (2.5 mL). Purification was performed with flash column chromatography over silica gel (20 g $SiO_2$, heptane/EtOAc, 100/0 to 50/50, gradient) to afford **4f** as a light yellow solid (88.5 mg, 0.32 mmol, 81%). **$^1$H NMR (700 MHz, DMSO-d$_6$):** $\delta$ 10.70 (brs, 1H), 7.48-7.45 (m, 2H), 7.35 (dd, *J* = 7.8, 1.5 Hz, 1H), 7.29-7.25 (m, 2H), 7.02 (dd, *J* = 8.0, 1.1 Hz, 1H), 6.99 (d, *J* = 8.0 Hz, 1H), 6.94 (dd, *J* = 7.8, 1.1 Hz, 1H), 3.25 (s, 3H). **$^{13}$C NMR (176 MHz, DMSO):** $\delta$ 162.7 (d, $J^{CF}$ = 245.8 Hz, C), 149.1 (C=O), 135.8 (d, $J^{CF}$ = 4.0 Hz, C), 135.8 (C), 130.5 (CH), 128.6 (d, $J^{CF}$ = 8.6 Hz, 2CH), 126.3 (CH), 122.8 (CH), 119.7 (C), 115.4 (d, $J^{CF}$ = 21.7 Hz, 2CH),

114.5 (CH), 105.5 (C), 51.0 ($CH_3$). **$^{19}$F NMR (659 MHz, DMSO-d$_6$):** $\delta$ -113.0. **FT-IR (neat, cm$^{-1}$):** 3094, 2990, 2930, 2360, 2332, 1716, 1708, 1602, 1507, 1494, 1345, 1276, 1228, 1217. **HRMS (ESI$^+$):** *m/z* calcd. for $C_{15}H_{12}FNO_3Na$ [M+Na]$^+$ 296.0693, found 296.0692.

***4-Methoxy-4-(3-methoxyphenyl)-1H-benzo[d][1,3]oxazin-2(4H)-one* 4g**

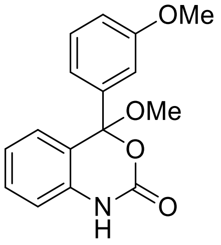


**4g**

The general procedure **A** was followed with **3g** (102 mg, 0.40 mmol) and MeOH (2.5 mL) Purification was performed with flash column chromatography over silica gel (20 g $SiO_2$, heptane/EtOAc, 100/0 to 50/50, gradient) to afford **4g** as a light brown solid (61.3 mg, 0.22 mmol, 54%). **$^1$H NMR (600 MHz, $CDCl_3$):** $\delta$ 9.38 (brs, 1H), 7.35-7.29 (m, 2H), 7.10-7.07 (m, 3H), 7.03 (ddd, $J$ = 8.0, 7.0, 1.9 Hz, 1H), 6.97 (dd, $J$ = 8.0, 0.9 Hz, 1H), 6.93 (ddd, $J$ = 8.1, 2.5, 0.9, 1H), 3.82 (s, 3H), 3.45 (s, 3H). **$^{13}$C NMR (150 MHz, $CDCl_3$):** $\delta$ 159.9 (C=O), 151.8 (C), 140.0 (C), 135.0 (C), 130.6 (CH), 129.6 (CH), 126.8 (CH), 123.7 (CH), 120.8 (C), 119.3 (CH), 115.0 (CH), 114.9 (CH), 112.6 (CH), 107.3 (C), 55.5 ($CH_3$), 52.0 ($CH_3$). **FT-IR (neat, cm$^{-1}$):** 1702, 1604, 1589, 1486, 1433, 1355, 1313, 1293, 1273, 1263, 1200, 1171, 1086, 1052, 1041, 1015. **HRMS (ESI$^+$):** *m/z* calcd. for $C_{16}H_{15}NO_4Na$ [M+Na]$^+$ 308.0893, found 308.0898.

***4-Methoxy-4-(perfluorophenyl)-1H-benzo[d][1,3]oxazin-2(4H)-one* 4h**

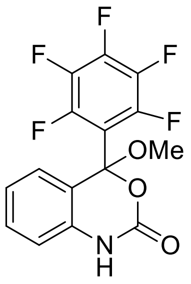


**4h**

General procedure **A** was followed with **3h** (126 mg, 0.40 mmol) and MeOH (2.5 mL). Purification was performed with flash column chromatography over silica gel (20 g $SiO_2$, heptane/EtOAc, 100/0 to 50/50, gradient) to afford **4h** as a light yellow solid (27.0 mg, 0.078 mmol, 20%). **$^1$H NMR (700 MHz, $CDCl_3$):** $\delta$ 9.07 (brs, 1H), 7.38-7.35 (m, 1H), 7.13-7.04 (m, 2H), 6.94 (d, $J$ = 8.0 Hz, 1H), 3.45 (s, 3H). **$^{13}$C NMR (176 MHz, $CDCl_3$):** $\delta$ 149.9 (C=O), 144.9 (dm, $J$ = 257.1 Hz, 2C), 141.8 (dm, $J$ = 257.2 Hz, C), 138.2 (dm, $J$ = 251.7 Hz, 2C), 134.5 (C), 131.5 (CH), 126.1 (CH), 124.3 (CH), 118.0 (C), 114.9 (CH), 114.8 (m, C), 105.7 (C), 51.8 ($CH_3$). **$^{19}$F NMR (659 MHz, $CDCl_3$):** $\delta$ -139.1 (m), -151.7 (m), -160.7 (m). **FT-IR (neat, cm$^{-1}$):** 3240, 3111, 2931, 1716, 1606, 1521, 1489, 1353, 1309, 1273, 1252, 1148. **HRMS (ESI$^+$):** *m/z* calcd. for $C_{15}H_8F_5NO_3Na$ [M+Na]$^+$ 368.0317, found 368.0317.

*4-Methoxy-4-methyl-1H-benzo[d][1,3]oxazin-2(4H)-one* **4i**

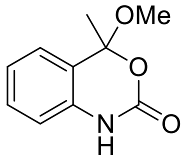

**4i**

General procedure **A** was followed with **3i** (65.3 mg, 0.40 mmol) and MeOH (2.5 mL). Purification was performed with flash column chromatography over silica gel (20 g $SiO_2$, heptane/EtOAc, 100/0 to 50/50, gradient) to afford **4i** as a light yellow solid (37.8 mg, 0.20 mmol, 49%). **$^{1}$H NMR (400 MHz, $CDCl_3$):** $\delta$ 9.81 (s, 1H), 7.31 (td, *J* = 7.7, 1.4 Hz, 1H), 7.27 (dd, *J* = 7.6, 1.6 Hz, 1H), 7.12 (td, *J* = 7.6, 1.0 Hz, 1H), 6.93 (dt, *J* = 7.9 Hz, 1H), 3.31 (s, 3H), 1.87 (s, 3H).**$^{13}$C NMR (100 MHz, $CDCl_3$):** $\delta$ 152.3 (C=O), 134.8 (C), 130.5 (CH), 125.2 (CH), 123.8 (CH), 120.4 (C), 114.9 (CH), 107.0 (C), 51.1 ($CH_3$), 27.1 ($CH_3$). **FT-IR (neat, cm$^{-1}$ ):** 3094, 2923, 2359, 2332, 1704, 1601, 1493, 1349, 1274, 1258. **HRMS (ESI$^+$):** *m/z* calcd. for $C_{10}H_{11}NO_3Na$ $[M+Na]^+$ 216.0631, found 216.0629.

*4-Isopropyl-4-methoxy-1H-benzo[d][1,3]oxazin-2(4H)-one* **4j**

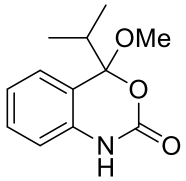

**4j**

General procedure **A** was followed with **3j** (76.5 mg, 0.40 mmol) and MeOH (2.5 mL). Purification was performed with flash column chromatography over silica gel (20 g $SiO_2$, heptane/EtOAc, 100/0 to 50/50, gradient) to afford **4j** as a light orange solid (52.0 mg, 0.25 mmol, 59%). **$^{1}$H NMR (600 MHz, $CDCl_3$):** $\delta$ 9.32 (brs, 1H), 7.31 (td, *J* = 7.8, 1.4 Hz, 1H), 7.20 (dd, *J* = 7.0, 0.7 Hz, 1H), 7.10 (td, *J* = 7.6, 1.0 Hz, 1H), 6.91 (dd, *J* = 8.0, 0.7 Hz, 1H), 3.21 (s, 3H), 2.27 (m, 1H), 1.08 (d, *J* = 6.8 Hz, 3H), 0.87 (d, *J* = 6.8 Hz, 3H). **$^{13}$C NMR (150 MHz, $CDCl_3$):** $\delta$ 151.3 (C=O), 138.1 (C), 129.4 (CH), 125.1 (CH), 122.5 (CH), 116.9 (C), 113.5 (CH), 111.0 (C), 50.6 ($CH_3$), 38.9 (CH), 15.7 ($CH_3$), 14.5 ($CH_3$). **FT-IR (neat, cm$^{-1}$):** 1703, 1601, 1494, 1461, 1434, 1373, 1332, 1271, 1252, 1248, 1143, 1132, 1096, 1042, 1012. **HRMS (ESI$^+$):** *m/z* calcd. for $C_{12}H_{15}NO_3Na$ $[M+Na]^+$ 244.0944, found 244.0947.

*4-Cyclopentyl-4-methoxy-1H-benzo[d][1,3]oxazin-2(4H)-one* **4k**

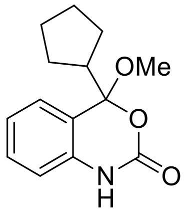

**4k**

General procedure **A** was followed with **3k** (86.9 mg, 0.40 mmol) and MeOH (2.5 mL). Purification was performed with flash column chromatography over silica gel (20 g $SiO_2$, heptane/EtOAc, 100/0 to 50/50, gradient) to afford **4k** as a light orange solid (35.1 mg, 0.14 mmol, 35%). **$^1$H NMR (700 MHz, $CDCl_3$):** $\delta$ 9.57 (brs, 1H), 7.29 (td, *J* = 7.9, 1.4 Hz, 1H), 7.23 (d, *J* = 7.6 Hz, 1H), 7.09 (td, *J* = 7.6, 1.0 Hz, 1H), 6.93 (dd, *J* = 8.0, 0.6 Hz, 1H), 3.21 (s, 3H), 2.61-2.56 (m, 1H), 1.83 (dd, *J* = 12.9, 8.4 Hz, 1H), 1.74-1.70 (m, 1H), 1.66-1.63 (m, 1H), 1.62-1.57 (m, 1H), 1.55-1.44 (m, 3H), 1.42-1.36 (m, 1H). **$^{13}$C NMR (176 MHz, $CDCl_3$)** $\delta$ 152.5 (C=O), 135.7 (C), 130.3 (CH), 126.0 (CH), 123.6 (CH), 118.8 (C), 114.7 (CH), 111.4 (C), 51.5 ($CH_3$), 51.1 ($CH_3$), 27.2 ($CH_2$), 26.1 ($CH_2$), 25.9 ($CH_2$), 25.6 ($CH_2$). **FT-IR (neat, $cm^{-1}$):** 2922, 2359, 2333, 1703, 1602, 1493, 1346, 1247. **HRMS (ESI$^+$):** *m/z* calcd. for $C_{14}H_{17}NO_3Na$ $[M+Na]^+$ 270.1101, found 270.1100.

***4-Allyl-4-methoxy-1H-benzo[d][1,3]oxazin-2(4H)-one* 4l**

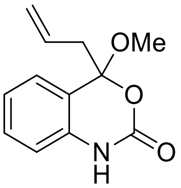


**4l**

The general procedure **A** was followed with **3l** (75.7 mg, 0.40 mmol) and MeOH (2.5 mL). Purification was performed with flash column chromatography over silica gel (20 g $SiO_2$, heptane/EtOAc, 100/0 to 50/50, gradient) to afford **4l** as a light yellow solid (14.0 mg, 0.064 mmol, 16%). **$^1$H NMR (700 MHz, $CDCl_3$):** $\delta$ 9.47 (brs, 1H), 7.31 (ddd, *J* = 7.9, 6.3, 1.3 Hz, 1H), 7.24 (d, *J* = 7.7 Hz, 1H), 7.13-7.08 (m, 1H), 6.92 (d, *J* = 7.9 Hz, 1H), 5.66 (ddt, *J* = 17.3, 10.2, 7.2 Hz, 1H), 5.12-5.09 (m, 1H), 5.09-5.07 (m, 1H), 3.28 (s, 3H), 2.87 (dd, *J* = 14.3, 7.5 Hz, 1H), 2.81 (dd, *J* = 14.3, 6.9 Hz, 1H). **$^{13}$C NMR (176 MHz, $CDCl_3$):** $\delta$ 151.7 (C=O), 135.4 (C), 130.4 (CH), 130.2 (CH), 125.7 (CH), 123.5 (CH), 120.4 (C), 118.1 (C), 114.5 (CH), 108.5 ($CH_2$), 51.2 (CH), 45.9 ($CH_2$). **FT-IR (neat, $cm^{-1}$):** 3246, 3103, 2982, 2938, 1704, 1602, 1494, 1358, 1256, 1148. **HRMS (ESI$^+$):** *m/z* calcd. for $C_{12}H_{13}NO_3Na$ $[M+Na]^+$ 242.0788, found 242.0789.

***4-Methoxy-6-methyl-4-phenyl-1H-benzo[d][1,3]oxazin-2(4H)-one* 6a**

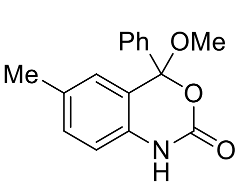


**6a**

The general procedure **A** was followed with **5a** (95.7 mg, 0.40 mmol) and MeOH (2.5 mL). Purification was performed with flash column chromatography over silica gel (20 g $SiO_2$, heptane/EtOAc, 100/0 to 50/50, gradient) to afford **6a** as a white solid (35.7 mg, 0.13 mmol, 33%). **$^1$H NMR (400 MHz, $CDCl_3$):** $\delta$ 9.08 (brs, 1H), 7.52 (dd, *J* = 7.7, 1.6 Hz, 2H), 7.45-7.38 (m, 3H), 7.11 (d, *J* = 8.2 Hz, 1H), 6.84 (d, *J* = 7.8 Hz, 2H), 3.43 (s, 3H), 2.24 (s, 3H). **$^{13}$C NMR (100 MHz, $CDCl_3$):** $\delta$ 151.7 (C=O), 138.8 (C), 133.4 (C), 132.7 (C), 131.2 (CH), 129.3

(CH), 128.6 (2CH), 127.0 (CH), 126.9 (2CH), 120.6 (C), 114.7 (CH), 107.5 (C), 51.9 ($CH_3$), 21.0 ($CH_3$). **FT-IR (neat, $cm^{-1}$):** 1708, 1607, 1512, 1450, 1347, 1251, 1226, 1195, 1164, 1095, 1075, 1008. **HRMS ($ESI^+$):** *m/z* calcd. for $C_{16}H_{15}NO_3Na$ $[M+Na]^+$ 292.0944, found 292.0941.

***6-Bromo-4-methoxy-4-phenyl-1H-benzo[d][1,3]oxazin-2(4H)-one* 6b**

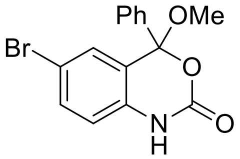


**6b**

The general procedure **A** was followed with **5b** (122 mg, 0.40 mmol) and MeOH (2.5 mL). Purification was performed with flash column chromatography over silica gel (20 g $SiO_2$, heptane/EtOAc, 100/0 to 50/50, gradient) to afford **6b** as a light yellow solid (60.9 mg, 0.18 mmol, 46%). **$^1$H NMR (500 MHz, DMSO-$d_6$):** $\delta$ 10.85 (brs, 1H), 7.54 (dd, *J* = 8.6, 2.2 Hz, 1H), 7.52-7.42 (m, 5H), 7.03 (d, *J* = 8.6 Hz, 1H), 6.95 (d, *J* = 2.2 Hz, 1H), 3.27 (s, *J* = 6.6 Hz, 3H). **$^{13}$C NMR (126 MHz, DMSO-$d_6$):** $\delta$ 148.8 (C=O), 138.5 (C), 135.2 (C), 133.3 (CH), 129.3 (CH), 128.7 (2CH), 128.5 (CH), 126.2 (2CH), 122.4 (C), 116.8 (CH), 114.0 (C), 105.1 (C), 51.1 ($CH_3$). **HRMS ($ESI^+$):** *m/z* calcd. for $C_{15}H_{12}NO_3BrNa$ $[M+Na]^+$ 355.9893, found 355.9886. The structure was analysed by X-Ray diffraction.

***4,7-Dimethoxy-4-phenyl-1H-benzo[d][1,3]oxazin-2(4H)-one* 6d**

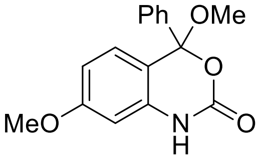


**6d**

The general procedure **A** was followed with **5d** (102 mg, 0.40 mmol) and MeOH (2.5 mL). Purification was performed with flash column chromatography over silica gel (20 g $SiO_2$, heptane/EtOAc, 100/0 to 50/50, gradient) to afford **6d** as a light yellow solid (59.0 mg, 0.21 mmol, 52%). **$^1$H NMR (400 MHz, $CDCl_3$):** $\delta$ 9.85 (brs, 1H), 7.51 (dd, *J* = 7.9, 1.6 Hz, 2H), 7.43-7.35 (m, 3H), 6.92 (d, *J* = 8.6 Hz, 1H), 6.57 (dd, *J* = 8.6, 2.4 Hz, 1H), 6.49 (d, *J* = 2.3 Hz, 1H), 3.78 (s, 3H), 3.42 (s, 3H). **$^{13}$C NMR (100 MHz, $CDCl_3$):** $\delta$ 161.4 (C), 152.4 (C=O), 139.3 (C), 136.5 (C), 129.2 (CH), 128.5 (2CH), 128.2 (CH), 126.8 (2CH), 112.7 (C), 110.4 (CH), 107.8 (C), 99.5 (CH), 55.7 ($CH_3$), 51.8 ($CH_3$). **FT-IR (neat, $cm^{-1}$):** 3220, 3080, 2937, 2836, 1711, 1624, 1598, 1517, 1449, 1339, 1290, 1260. **HRMS ($ESI^+$):** *m/z* calcd. for $C_{16}H_{15}NO_4Na$ $[M+Na]^+$ 308.0893, found 308.0891

***4-Methoxy-8-methyl-4-phenyl-1H-benzo[d][1,3]oxazin-2(4H)-one* 6e**

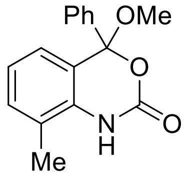

**6e**

The general procedure **E** was followed with **5e** (95.7 mg, 0.40 mmol) and MeOH (2.5 mL). Purification was performed with flash column chromatography over silica gel (20 g $SiO_2$, heptane/EtOAc, 100/0 to 50/50, gradient) to afford **6e** as a light yellow solid (32.3 mg, 0.12 mmol, 30%). **$^{1}$H NMR (600 MHz, $CDCl_3$):** $\delta$ 8.32 (brs, 1H), 7.52 (dd, *J* = 8.0, 1.2 Hz, 2H), 7.43-7.37 (m, 3H), 7.16 (d, *J* = 7.3 Hz, 1H), 6.94 (t, *J* = 7.6 Hz, 1H), 6.89 (d, *J* = 7.6 Hz, 1H), 3.44 (s, 3H), 2.34 (s, 3H). **$^{13}$C NMR (150 MHz, $CDCl_3$):** $\delta$ 151.1 (C=O), 138.6 (C), 133.4 (C), 131.8 (CH), 129.2 (CH), 128.5 (2CH), 127.0 (2CH), 124.7 (CH), 123.1 (CH), 122.8 (C), 121.0 (C), 107.2 (C), 52.0 ($CH_3$), 16.8 ($CH_3$). **FT-IR (neat, cm$^{-1}$):** 1706, 1600, 1493, 1476, 1250, 1348, 1256, 1221, 1082, 1067, 1010. **HRMS (ESI$^+$):** *m/z* calcd. for $C_{16}H_{15}NO_3Na$ $[M+Na]^+$ 292.0944, found 292.0941.

***4-Methoxy-4-phenyl-8-(trifluoromethyl)-1H-benzo[d][1,3]oxazin-2(4H)-one* 6f**

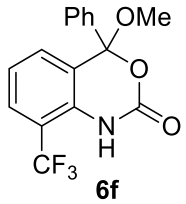

**6f**

The general procedure **E** was followed with **5f** (117 mg, 0.40 mmol) and MeOH (2.5 mL). Purification was performed with flash column chromatography over silica gel (20 g $SiO_2$, heptane/EtOAc, 100/0 to 50/50, gradient) to afford **6f** as a light yellow solid (74.7 mg, 0.23 mmol, 58%). **$^{1}$H NMR (700 MHz, $CDCl_3$):** $\delta$ 7.64 (brs, 1H), 7.60 (d, *J* = 7.8 Hz, 1H), 7.54-7.50 (m, 2H), 7.47-7.42 (m, 3H), 7.24 (d, *J* = 7.8 Hz, 1H), 7.11 (t, *J* = 7.8 Hz, 1H), 3.44 (s, 3H).**$^{13}$C NMR (176 MHz, $CDCl_3$):** $\delta$ 149.1 (C=O), 137.0 (C), 132.9 (q, $J^{CF}$ = 1.3 Hz, C), 131.0 (CH), 129.7 (CH), 128.9 (2CH), 127.7 (q, $J^{CF}$ = 4.8 Hz, CH), 127.1 (2CH), 123.3 (q, $J^{CF}$ = 272.9 Hz, C), 123.7 (C), 123.0 (CH), 115.1 (q, $J^{CF}$ = 32.8 Hz, C), 106.3 (C), 52.1 ($CH_3$). **$^{19}$F NMR (376 MHz, $CDCl_3$):** $\delta$ -60.3. **FT-IR (neat, cm$^{-1}$):** 3264, 3218, 3157, 2359, 2332, 1732, 1602, 1452, 1324, 1275, 1266, 1167, 1117. **HRMS (ESI$^+$):** *m/z* calcd. for $C_{16}H_{12}F_3NO_3Na$ $[M+Na]^+$ 346.0661, found 346.0663.

***4-Hydroxy-3-methyl-4-phenyl-3,4-dihydroquinazolin-2(1H)-one* 10**

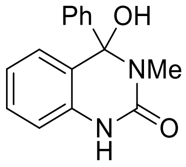


**10**

The general procedure **A** was followed with **1** (90.1 mg, 0.40 mmol) and $MeNH_2$ (1.0 M in THF, 4.0 mL, 20 equiv.) in 1.0 mL of THF. Purification was performed with flash column chromatography over silica gel (20 g $SiO_2$, heptane/EtOAc, 100/0 to 50/50, gradient) to afford **10** as a yellow solid (64.7 mg, 0.25 mmol, 64%). **$^1$H NMR (400 MHz, DMSO-d$_6$):** $\delta$ 9.73 (brs, 1H), 7.42-7.38 (m, 2H), 7.37-7.32 (m, 2H), 7.25 (dd, $J$ = 7.3, 6.5 Hz, 1H), 7.11 (ddd, $J$ = 7.7, 7.7, 1.4 Hz, 1H), 7.00 (s, 1H), 6.88 (dd, $J$ = 7.7, 1.5 Hz, 1H), 6.82 (dd, $J$ = 7.7, 1.4 Hz, 1H), 6.77 (ddd, $J$ = 7.4, 7.3, 1.4 Hz, 1H), 2.60 (s, 3H). **$^{13}$C NMR (100 MHz, DMSO-d$_6$):** $\delta$ 151.6 (C=O), 145.3 (C), 134.8 (C), 128.5 (CH), 128.2 (2CH), 128.0 (CH), 127.4 (CH), 125.7 (2CH), 124.4 (C), 120.9 (CH), 113.3 (CH), 86.8 (C), 28.0 ($CH_3$). **FT-IR (neat, cm$^{-1}$):** 1651, 1605, 1513, 1499, 1469, 1445, 1431, 1398, 1347, 1327, 1278, 1186, 1168, 1023. **HRMS (ESI$^+$):** *m/z* calcd. for $C_{15}H_{14}N_2O_2$ $[M-OH]^+$ 237.1022, found 237.1024.

# 5 Mechanistic Experiments (Scheme 3)

## 5.1 $^{18}$O labelled experiments

*Synthesis of* ***1-$^{18}$O***

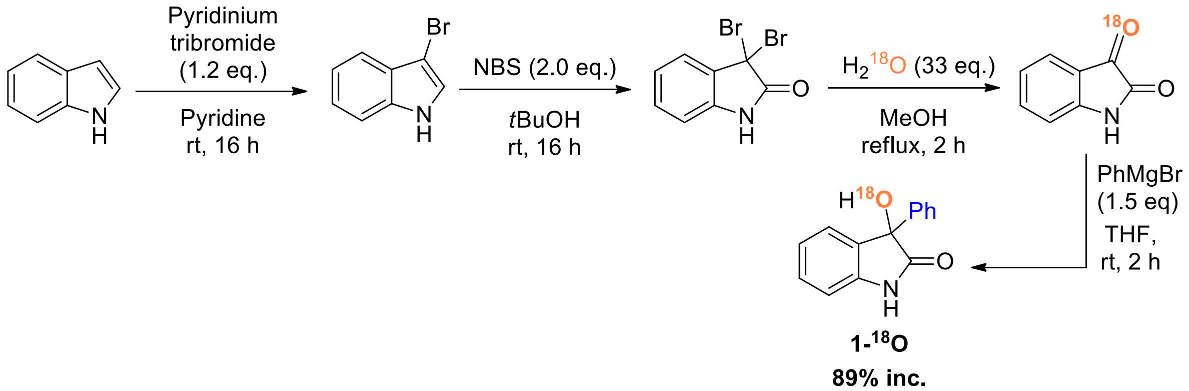


***3-Bromo-1H-indole***

Following a reported procedure,[8] a 100 mL round bottom flask was charged with indole (2.0 g, 17.1 mmol, 1.0 equiv.) in pyridine (20 mL). Then, pyridinium tribromide (6.6 g, 20.5 mmol, 1.2 equiv) in pyridine (20 mL) was added dropwise over 10 min after which the reaction was left to stir 16 h at rt. Next, ice water was added, and the reaction mixture was extracted with $Et_2O$ (20 mL). The organic layers were washed with 6N HCl (20 mL) and saturated aq. $NaHCO_3$ (20 mL), dried with $MgSO_4$, filtered and concentrated in vacuo. The

desired product (1.99 g, 10.2 mmol, 60%) is used directly in the next step without further purification.

### *3,3-Dibromoindolin-2-one*

Following a reported procedure,[9] a 50 mL round bottom flask was charged with 3-bromo-1*H*-indole (1.0 g, 5.1 mmol, 1.0 equiv.) in *t*BuOH (10 mL). Then, *N*-bromosuccinimide (1.8 g, 10.2 mmol, 2.0 equiv) was added portion wise and reaction was stirred at rt for 16 h. Next, a saturated solution of $Na_2S_2O_3$ was added, and the reaction mixture was extracted with $Et_2O$ (20 mL). The organic layers were washed with brine (20 mL), dried with $MgSO_4$, filtered and concentrated in vacuo. The desired product (1.6 g, 5.5 mmol, quant.) is used directly in the next step without further purification.

### *$^{18}$O-Labelled Isatin*

In a sealed tube, 3,3-dibromoindolin-2-one (1.6 g, 5.5 mmol, 1.0 equiv.) was introduced followed by MeOH (10 ml) and $H_2{}^{18}O$ (3.3 ml, 181 mmol, 33 equiv.). The mixture was heated at reflux for 2 h and then concentrated in vacuo. Purification by FC (20 g $SiO_2$, heptane/EA: 100/0 to 60/40) afforded the desired product as an orange solid (150 mg, 1.02 mmol, 19%, 89% incorporation). **$^1$H NMR (400 MHz, $CDCl_3$):** $\delta$ 7.88 (brs, 1H), 7.63 (d, $J$ = 7.6 Hz, 1H), 7.57 (t, $J$ = 7.9 Hz, 1H), 7.13 (t, $J$ = 7.6 Hz, 1H), 6.91 (d, $J$ = 7.9 Hz, 1H). **HRMS (ESI$^+$):** *m/z* calcd. for $C_8H_5NO^{18}ONa$ $[M+Na]^+$ 172.0255, found 172.0254.

### *3-Hydroxy-3-phenylindolin-2-one* **1-$^{18}$O**

General procedure **B** was followed with $^{18}$O-Labelled Isatin (150 mg, 1.02 mmol) and phenylmagnesium bromide (3.0 M in $Et_2O$, 0.68 mL, 2.04 mmol, 2.0 equiv.) to afford **1-$^{18}$O** as a yellow solid (90.4 mg, 0.40 mmol, 39%). **$^1$H NMR (400 MHz, DMSO-$d_6$):** $\delta$ 10.40 (s, 1H), 7.33-7.23 (m, 6H), 7.10 (d, $J$ = 6.9 Hz, 1H), 6.96 (td, $J$ = 7.7, 1.1 Hz, 1H), 6.91 (d, $J$ = 7.7 Hz, 1H), 6.63 (s, 1H). **HRMS (ESI$^+$):** *m/z* calcd. for $C_{14}H_{11}NO^{18}ONa$ $[M+Na]^+$ 250.0724, found 250.0725.

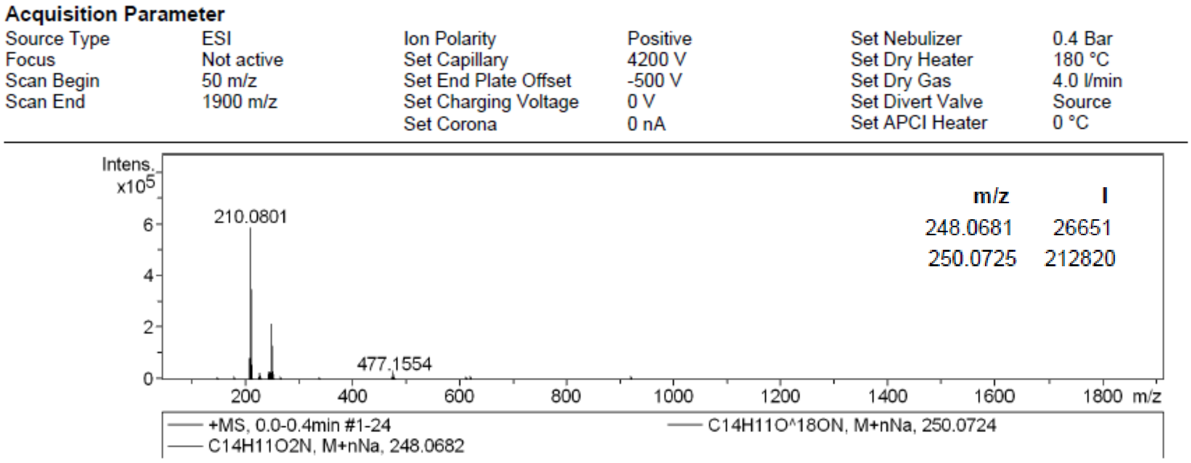

*Characterization data of **2a-18O***

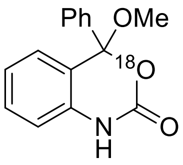


**2a-18O**

General procedure **A** was followed with **1-18O** (55.0 mg, 0.38 mmol) and MeOH (2.5 mL) Purification was performed with flash column chromatography over silica gel (20 g $SiO_2$, heptane/EtOAc, 100/0 to 50/50, gradient) to afford **2a-18O** as a white solid (35.0 mg, 0.14 mmol, 37%, 88% incorporation). **$^{1}$H NMR (400 MHz, $CDCl_3$):** $\delta$ 9.62 (brs, 1H), 7.53 (dd, $J$ = 7.9, 1.7 Hz, 2H), 7.45-7.35 (m, 3H), 7.34-7.28 (m, 1H), 7.08-7.00 (m, 2H), 6.99 (d, $J$ = 8.0 Hz, 1H), 3.44 (s, 3H). **$^{13}$C NMR (100 MHz, $CDCl_3$):** $\delta$ 153.0 (C=O), 138.7 (C), 135.1 (C), 130.6 (CH), 129.3 (CH), 128.6 (2CH), 126.9 (3CH), 123.7 (CH), 120.8 (C), 114.9 (CH), 107.5 (C), 52.0 ($CH_3$). **HRMS (ESI$^+$):** $m/z$ calcd. for $C_{15}H_{13}NO^{18}ONa$ $[M+Na]^+$ 280.0830, found 280.0829.

**Acquisition Parameter**

| | | | | | |
|---|---|---|---|---|---|
| Source Type | ESI | Ion Polarity | Positive | Set Nebulizer | 0.4 Bar |
| Focus | Not active | Set Capillary | 4200 V | Set Dry Heater | 180 °C |
| Scan Begin | 50 m/z | Set End Plate Offset | -500 V | Set Dry Gas | 4.0 l/min |
| Scan End | 1900 m/z | Set Charging Voltage | 0 V | Set Divert Valve | Source |
| | | Set Corona | 0 nA | Set APCI Heater | 0 °C |

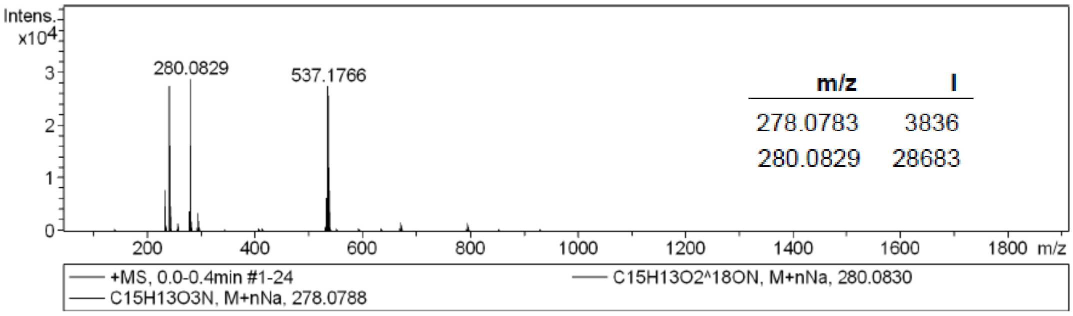


## 5.2 Formation of methyl (2-benzoylphenyl)carbamate 7

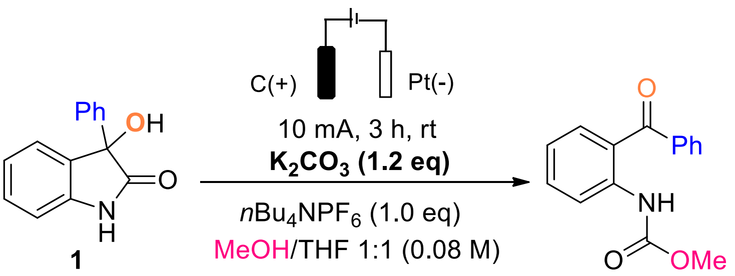


**7, 31%**

General procedure **A** was followed with **1** (90.1 mg, 0.40 mmol) and MeOH (2.5 mL) in presence of $K_2CO_3$ (1.2 equiv.). Purification was performed with flash column chromatography over silica gel (20 g $SiO_2$, heptane/EtOAc, 100/0 to 50/50, gradient) to afford

**7** as a white solid (31.4 mg, 0.12 mmol, 31%). **$^{1}$H NMR (400 MHz, $CDCl_3$):** $\delta$ 10.29 (brs, 1H), 8.42 (d, $J$ = 8.4 Hz, 1H), 7.69 (d, $J$ = 7.1 Hz, 2H), 7.61-7.50 (m, 3H), 7.47 (t, $J$ = 7.6 Hz, 2H), 7.03 (t, $J$ = 7.6 Hz, 1H), 3.79 (s, 3H). **$^{13}$C NMR (100 MHz, $CDCl_3$):** $\delta$ 199.4 (C=O), 154.4 (C=O), 141.1 (C), 138.9 (C), 134.4 (CH), 133.7 (CH), 132.4 (CH), 130.0 (2CH), 128.4 (2CH), 123.0 (C), 121.3 (CH), 120.0 (CH), 52.5 ($CH_3$). **HRMS (ESI$^+$):** *m/z* calcd. for $C_{15}H_{13}NO_3Na$ $[M+Na]^+$ 278.0788, found 278.0789.

## 5.3 Reaction of peroxide 8 in the standard conditions

*Synthesis of 3-(tert-butylperoxy)-3-(4-methoxyphenyl)indolin-2-one* **8**

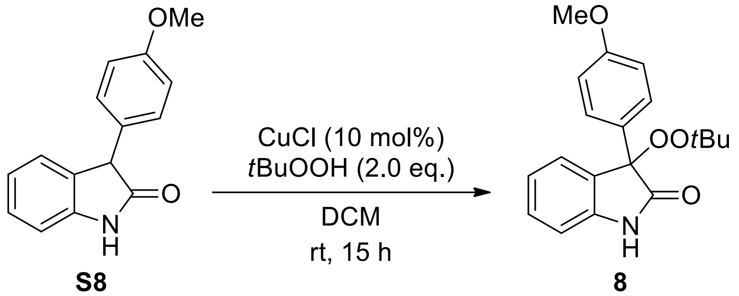


Following a reported procedure,[10] a flamed-dried schlenk tube was charged with 3-(4-methoxyphenyl)indolin-2-one **S8** (300 mg, 1.25 mmol, 1 equiv) and copper(I) chloride (12.4 mg, 0.13 mmol, 0.1 equiv). The tube is sealed, then evacuated and backfilled with argon (3 times). Then, $CH_2Cl_2$ (12 mL) is added, followed by dropwise addition of tert-butyl hydroperoxide (5.5 M in decane, 2.5 mL, 2 equiv) and the reaction is stirred at rt for 15 h. The reaction mixture is dry-loaded onto silica gel and purified directly by FC (40 g $SiO_2$, heptane/EtOAc, 100/0 to 50/50, gradient) to afford **8** (210 mg, 0.64 mmol, 51%). **$^{1}$H NMR (700 MHz, $CDCl_3$):** $\delta$ 8.36 (brs, 1H), 7.40 (d, $J$ = 9.0 Hz, 2H), 7.34 (d, $J$ = 7.4 Hz, 1H), 7.30 (td, $J$ = 7.7, 1.2 Hz, 1H), 7.09 (td, $J$ = 7.6, 0.9 Hz, 1H), 6.89 (d, $J$ = 7.8 Hz, 1H), 6.86 (d, $J$ = 9.0 Hz, 2H), 3.78 (s, 3H), 1.18 (s, 9H). **$^{13}$C NMR (176 MHz, $CDCl_3$):** $\delta$ 176.6 (C=O), 160.3 (C), 141.8 (C), 129.8 (CH), 128.9 (C), 128.9 (2CH), 127.9 (C), 126.6 (CH), 122.6 (CH), 114.0 (2CH), 110.3 (CH), 86.2 (C), 80.9 (C), 55.4 ($CH_3$), 26.7 ($3CH_3$). **HRMS (ESI$^+$):** *m/z* calcd. for $C_{19}H_{21}NO_4Na$ $[M+Na]^+$ 350.1363, found 350.1364.

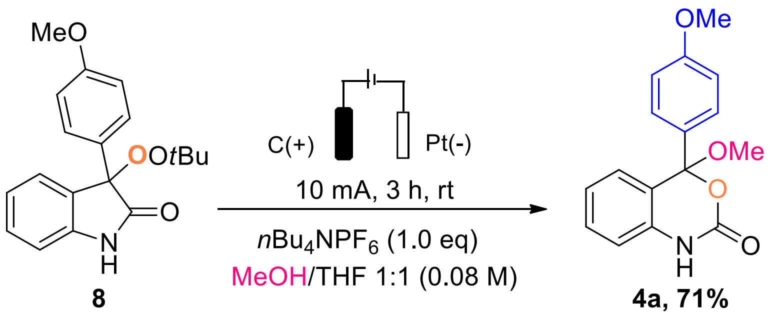


General procedure **A** was followed with **8** (100 mg, 0.31 mmol) and MeOH (2.5 mL). Purification was performed with flash column chromatography over silica gel (20 g $SiO_2$, heptane/EtOAc, 100/0 to 50/50, gradient) to afford **4a** as a white solid (62.0 mg, 0.22 mmol,

71%). **$^{1}$H NMR (400 MHz, $CDCl_3$):** $\delta$ 8.79 (brs, 1H), 7.43 (d, $J$ = 8.9 Hz, 2H), 7.31 (ddd, $J$ = 8.0, 7.0, 1.9 Hz, 1H), 7.10-7.00 (m, 2H), 6.94-6.90 (m, 3H), 3.82 (s, 3H), 3.43 (s, 3H). **$^{13}$C NMR (100 MHz, $CDCl_3$):** $\delta$ 160.3 (C=O), 151.6 (C), 136.1 (C), 130.9 (C), 130.5 (CH), 128.4 (2CH), 126.9 (CH), 123.6 (CH), 121.3 (C), 114.7 (CH), 113.9 (2CH), 107.6 (C), 55.5 ($CH_3$), 52.0 ($CH_3$). **FT-IR (neat, $cm^{-1}$):** 1700, 1604, 1513, 1505, 1494, 1432, 1353, 1319, 1305, 1251, 1209, 1185, 1169, 1153, 1095, 1032, 1009. **HRMS (ESI$^+$):** *m/z* calcd. for $C_{16}H_{15}NO_4Na$ $[M+Na]^+$ 308.0893, found 308.0887.

## 5.4 BHT trapping experiment

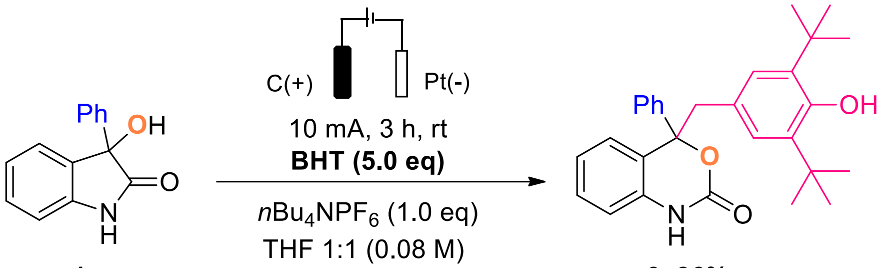


**1** **9, 36%**

General procedure **A** was followed with **1** (90.1 mg, 0.40 mmol) and MeOH (2.5 mL) in the presence of BHT (441 mg, 2.0 mmol, 5.0 equiv.). Purification was performed with flash column chromatography over silica gel (20 g $SiO_2$, heptane/EtOAc, 100/0 to 50/50, gradient) to afford **4a** as a white solid (66.2 mg, 0.15 mmol, 37%). **1a** was also recovered (52.8 mg, 0.23 mmol, 59%). **$^{1}$H NMR (400 MHz, $CDCl_3$):** $\delta$ 7.46-7.35 (m, 2H), 7.33-7.23 (m, 5H), 7.11 (s, 2H), 7.03 (t, $J$ = 7.5 Hz, 1H), 6.86 (d, $J$ = 7.8 Hz, 1H), 5.17 (s, 1H), 5.12 (d, $J$ = 15.4 Hz, 1H), 4.55 (d, $J$ = 15.4 Hz, 1H), 3.60 (s, 1H), 1.39 (s, 18H). **$^{13}$C NMR (100 MHz, $CDCl_3$):** $\delta$ 177.8 (C=O), 153.5 (C), 143.1 (C), 140.6 (C), 136.4 (2C), 131.9 (C), 129.9 (CH), 128.8 (2CH), 128.4 (CH), 126.3 (C), 125.3 (2CH), 125.0 (CH), 124.2 (2CH), 123.5 (CH), 109.9 (CH), 78.1 (C), 44.3 ($CH_2$), 34.4 (2C), 30.4 ($6CH_3$). **HRMS (ESI$^+$):** *m/z* calcd. for $C_{29}H_{33}NO_3Na$ $[M+Na]^+$ 466.2353, found 466.2355.

# 6 X-Ray Diffraction Analysis of compound 6b

The X-ray intensity data was measured on Bruker D8 Venture diffractometer equipped with multilayer monochromator, Mo K/α INCOATEC micro focus sealed tube and Oxford cooling system. The structure was solved by *Charge Flipping*. Non-hydrogen atoms were refined with *anisotropic displacement parameters*. Hydrogen atoms were inserted at calculated positions and refined with riding model. The following software was used: *Bruker SAINT software package*[11] using a narrow-frame algorithm for frame integration, *SADABS*[12] for absorption correction, *OLEX2*[13] for structure solution, refinement, molecular diagrams and graphical user-interface, *Shelxle*[14] for refinement and graphical user-interface *SHELXS-2015*[15] for structure solution, *SHELXL-2015*[15] for refinement, *Platon*[16] for symmetry check. Experimental data and CCDC-Codes Experimental data (Available online: http://www.ccdc.cam.ac.uk/conts/retrieving.html) can be found in **Table S1**. Crystal data, data collection parameters, and structure refinement details are given in **Table S2**. Asymmetric Units visualized in **Figure S1**.

The sample was prepared by slow liquid-liquid diffusion using a mixture of $CH_2Cl_2$/MeOH/AcOEt (1/1/1).

**Table S1.** Experimental parameter and CCDC-Code.

| Sample | Machine | Source | Temp. | Detector Distance | Time/ Frame | #Frames | Frame width | CCDC |
|---|---|---|---|---|---|---|---|---|
| | Bruker | | [K] | [mm] | [s] | | [°] | |
| **6b** | D8 | Mo | 100 | 30 | 3 | 2299 | 0.360 | 2102358 |

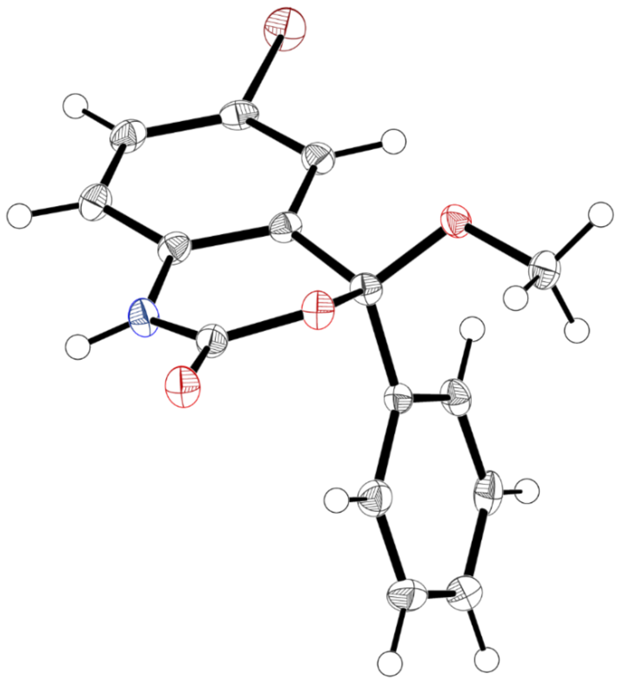

**Figure S1.** Crystal structure of **6b** drawn with 50% displacement ellipsoid. Only one part visualized. The bond precision for C-C single bonds is 0.0057Å.

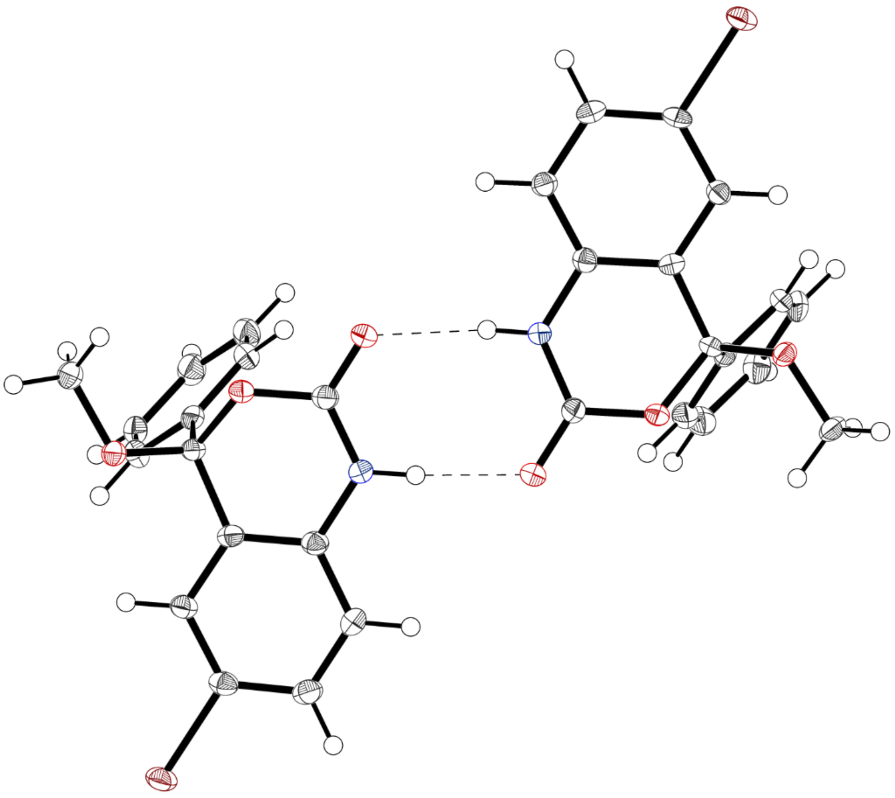

**Figure S2.** Crystal structure of **6b** drawn with 50% displacement ellipsoid. The bond precision for C-C single bonds is 0.0057Å.

**Table S2.** Sample and crystal data, data collection and structure refinement. More detailed information can be found in the Cif Code of CCDC: **2102358.**

| | |
|---|---|
| Identification code | mipa361_P-1 |
| Empirical formula | $C_{15}H_{12}BrNO_3$ |
| Formula weight | 334.17 |
| Temperature/K | 100.0 |
| Crystal system | triclinic |
| Space group | P-1 |
| a/Å | 9.9912(6) |
| b/Å | 11.1573(6) |
| c/Å | 12.8256(7) |
| α/° | 99.968(2) |
| β/° | 105.709(2) |
| γ/° | 99.578(2) |

| | |
|---|---|
| Volume/Å$^3$ | 1320.82(13) |
| Z | 4 |
| $\rho_{calc}$g/cm$^3$ | 1.680 |
| $\mu$/mm$^{-1}$ | 3.118 |
| F(000) | 672.0 |
| Crystal size/mm$^3$ | 0.358 × 0.313 × 0.124 |
| Radiation | MoKα (λ = 0.71073) |
| 2Θ range for data collection/° | 3.804 to 56.564 |
| Index ranges | -13 ≤ h ≤ 12, -14 ≤ k ≤ 14, 0 ≤ l ≤ 17 |
| Reflections collected | 9148 |
| Independent reflections | 6510 [$R_{int}$ = 0.1797, $R_{sigma}$ = 0.0928] |
| Data/restraints/parameters | 6510/0/363 |
| Goodness-of-fit on F$^2$ | 1.067 |
| Final R indexes [I>=2σ (I)] | $R_1$ = 0.0763, w$R_2$ = 0.1391 |
| Final R indexes [all data] | $R_1$ = 0.1015, w$R_2$ = 0.1492 |
| Largest diff. peak/hole / e Å$^{-3}$ | 2.29/-1.28 |

# 7 NMR Spectra

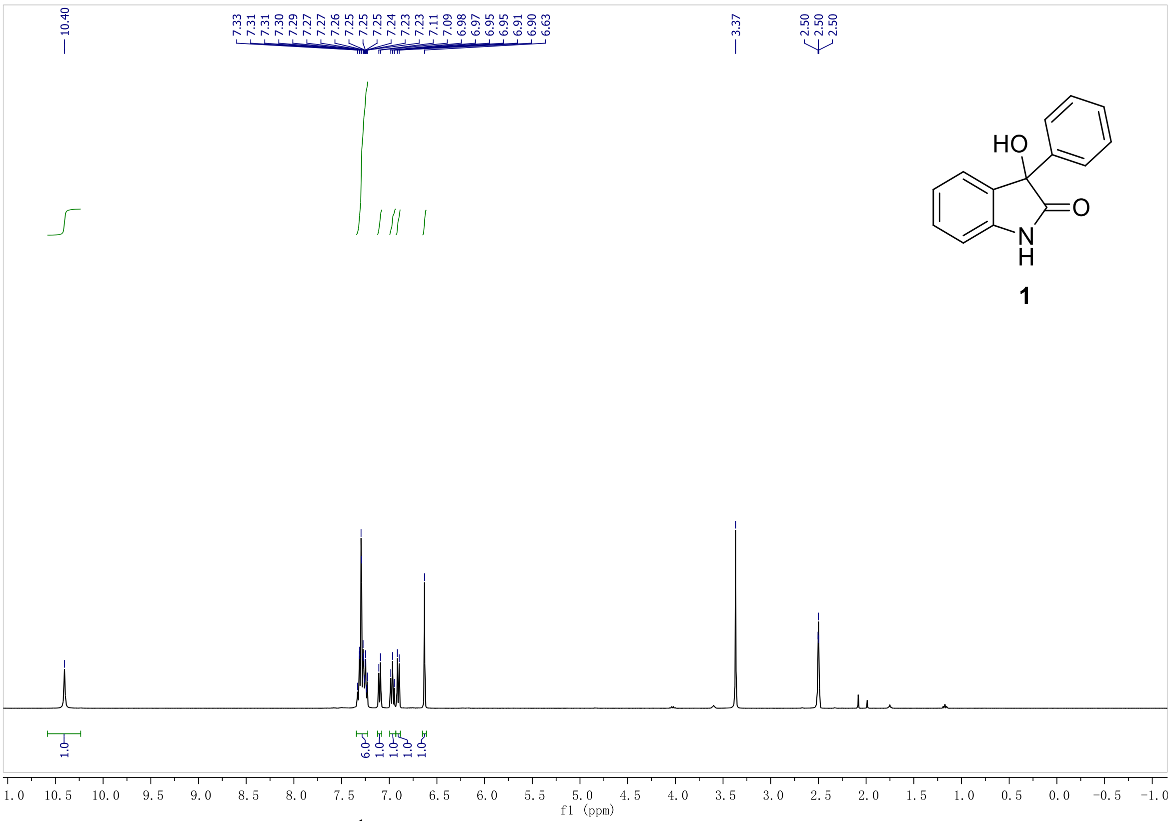


$^{1}$H NMR in DMSO-$d_6$ at 400 MHz

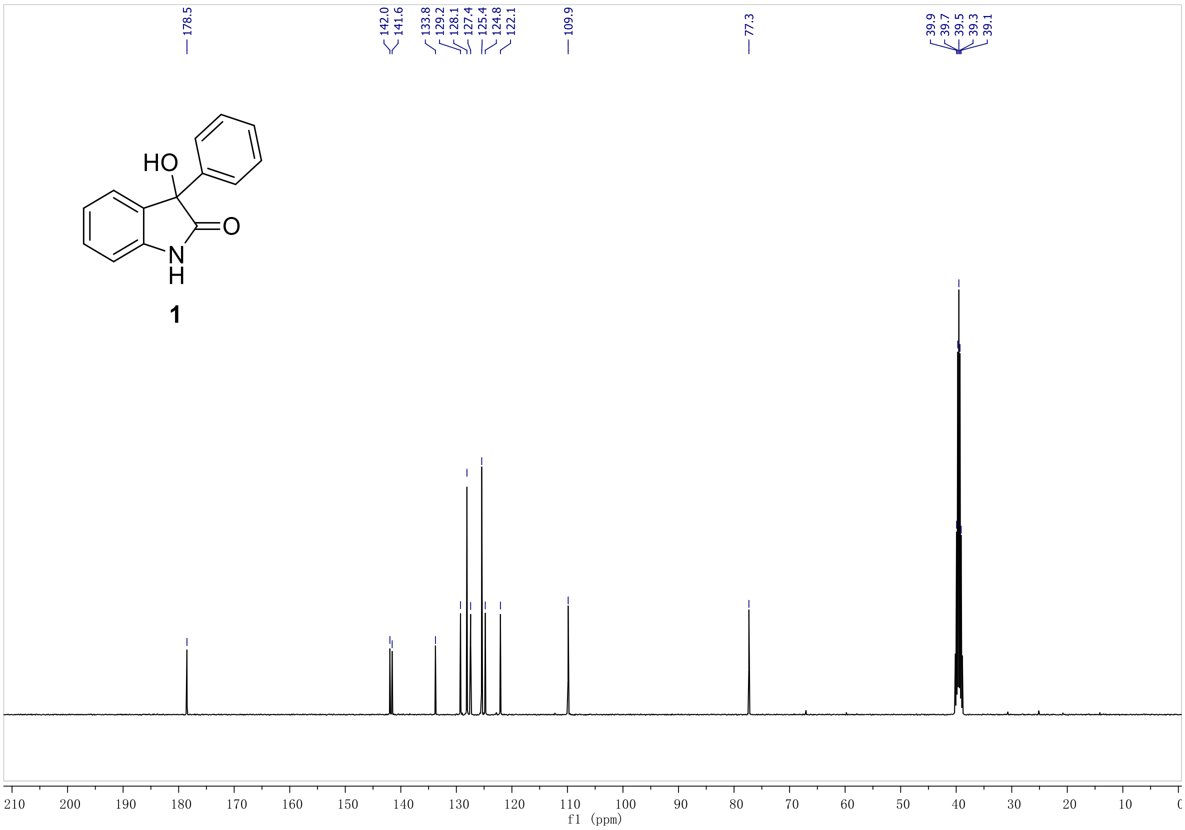


$^{13}$C{$^{1}$H} NMR in DMSO-$d_6$ at 100 MHz

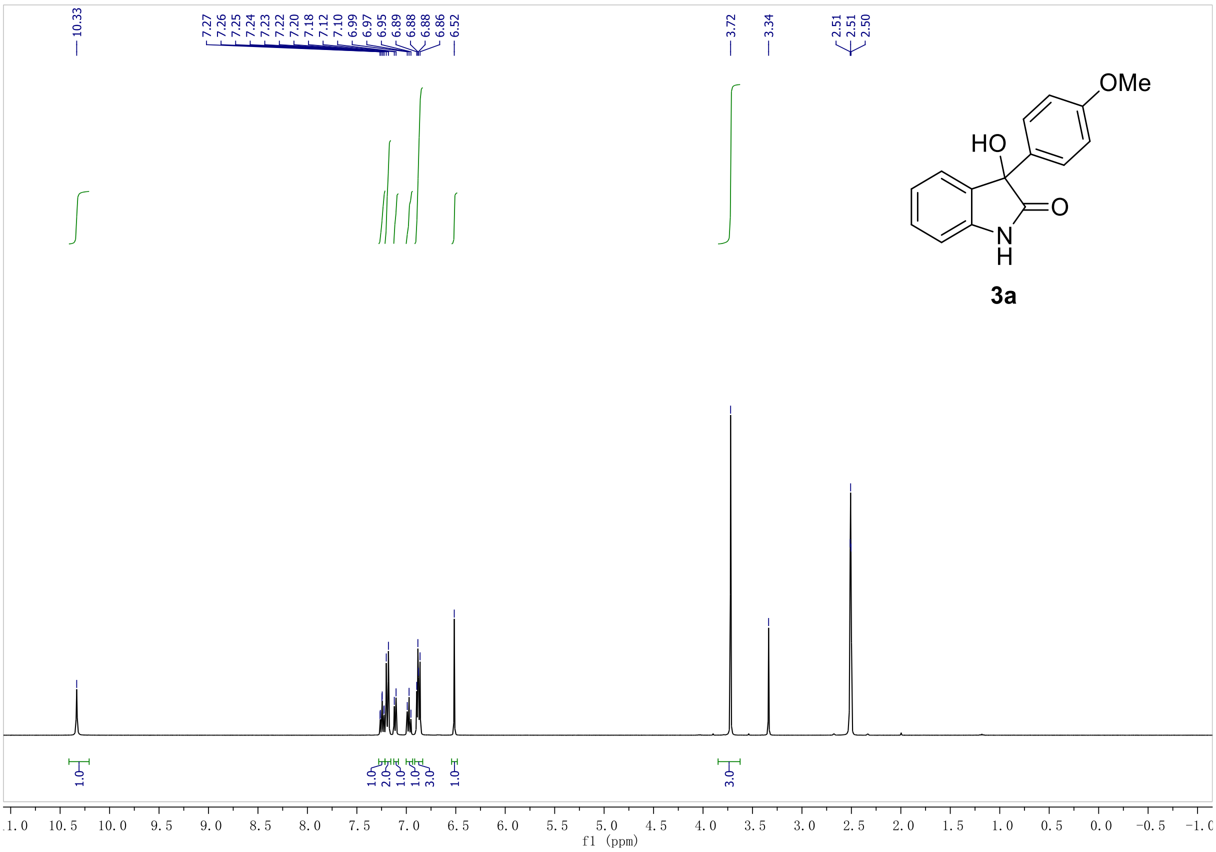


$^{1}$H NMR in DMSO-$d_6$ at 400 MHz

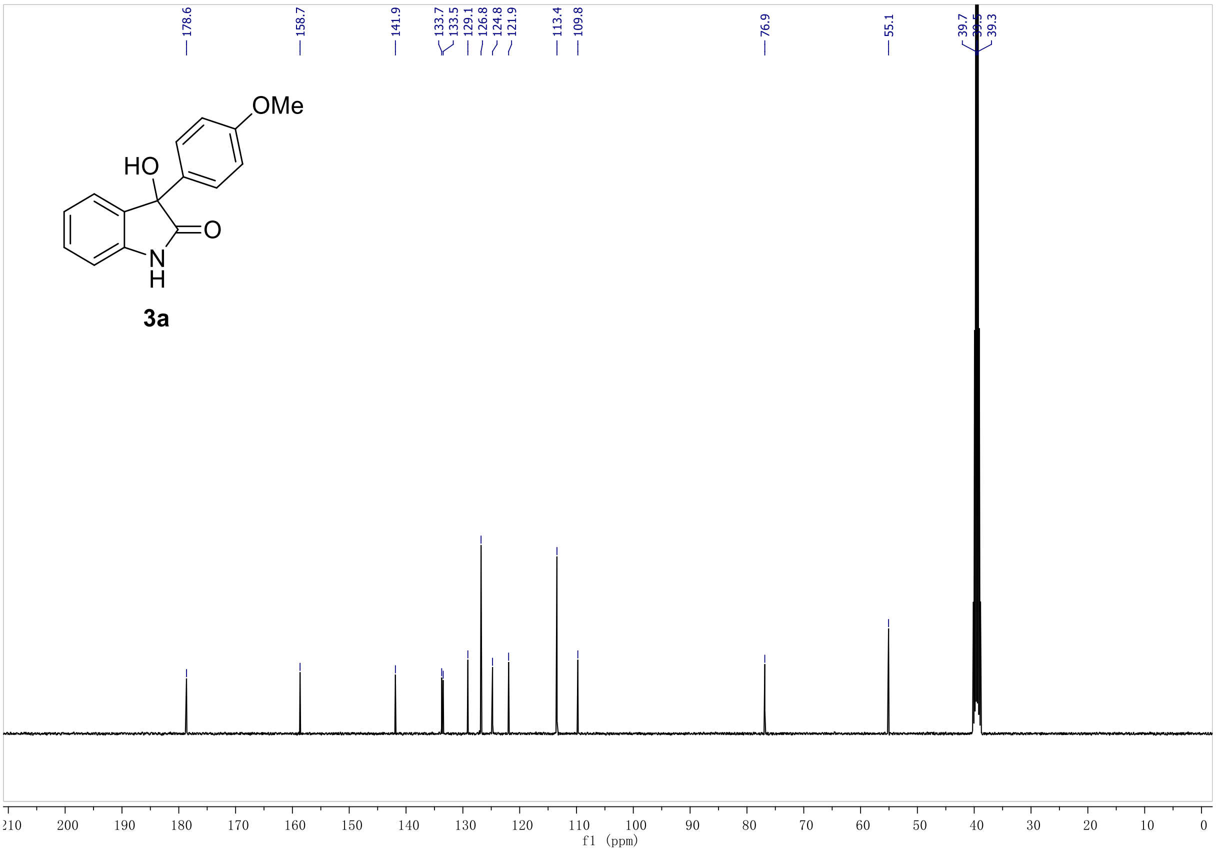


$^{13}$C{$^{1}$H} NMR in DMSO-$d_6$ at 100 MHz

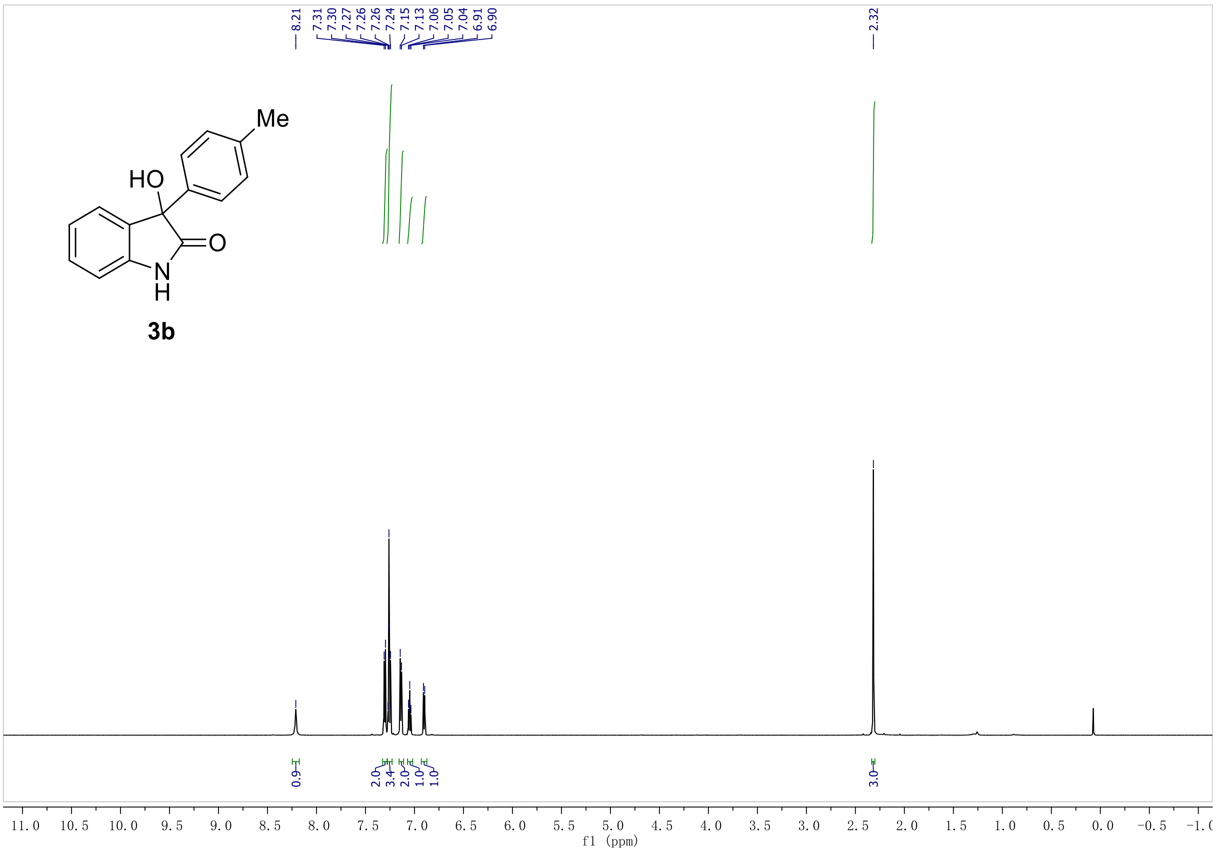


$^{1}$H NMR in $CDCl_3$ at 600 MHz

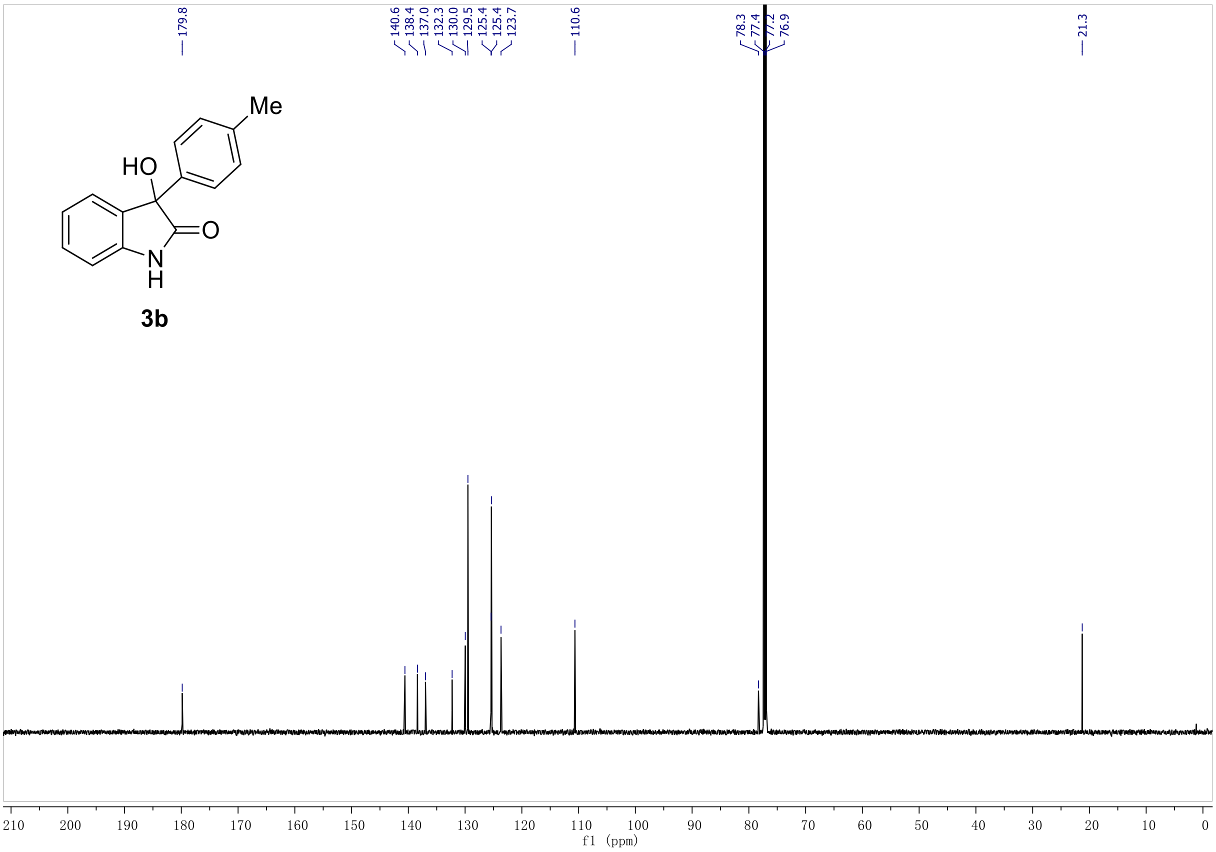


$^{13}C\{^{1}H\}$ NMR in $CDCl_3$ at 150 MHz

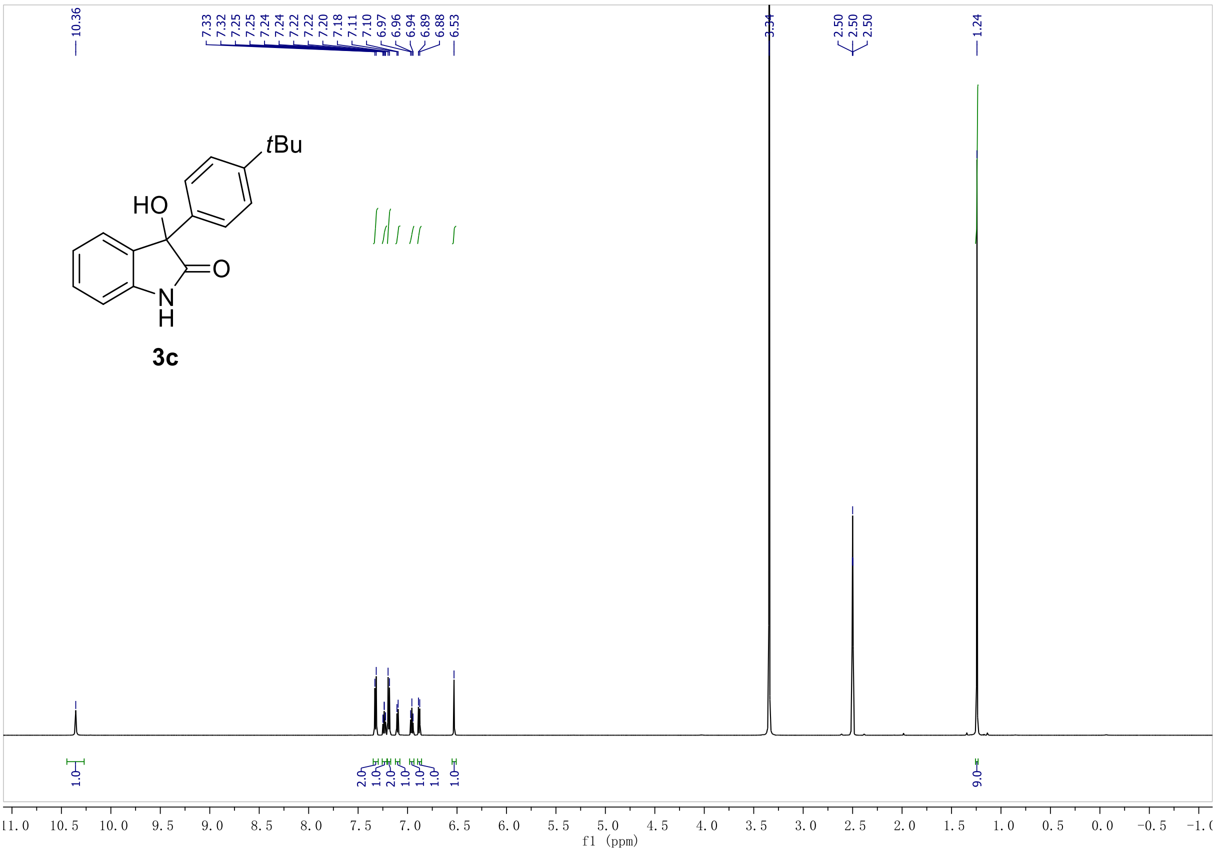


$^1$H NMR in DMSO-$d_6$ at 600 MHz

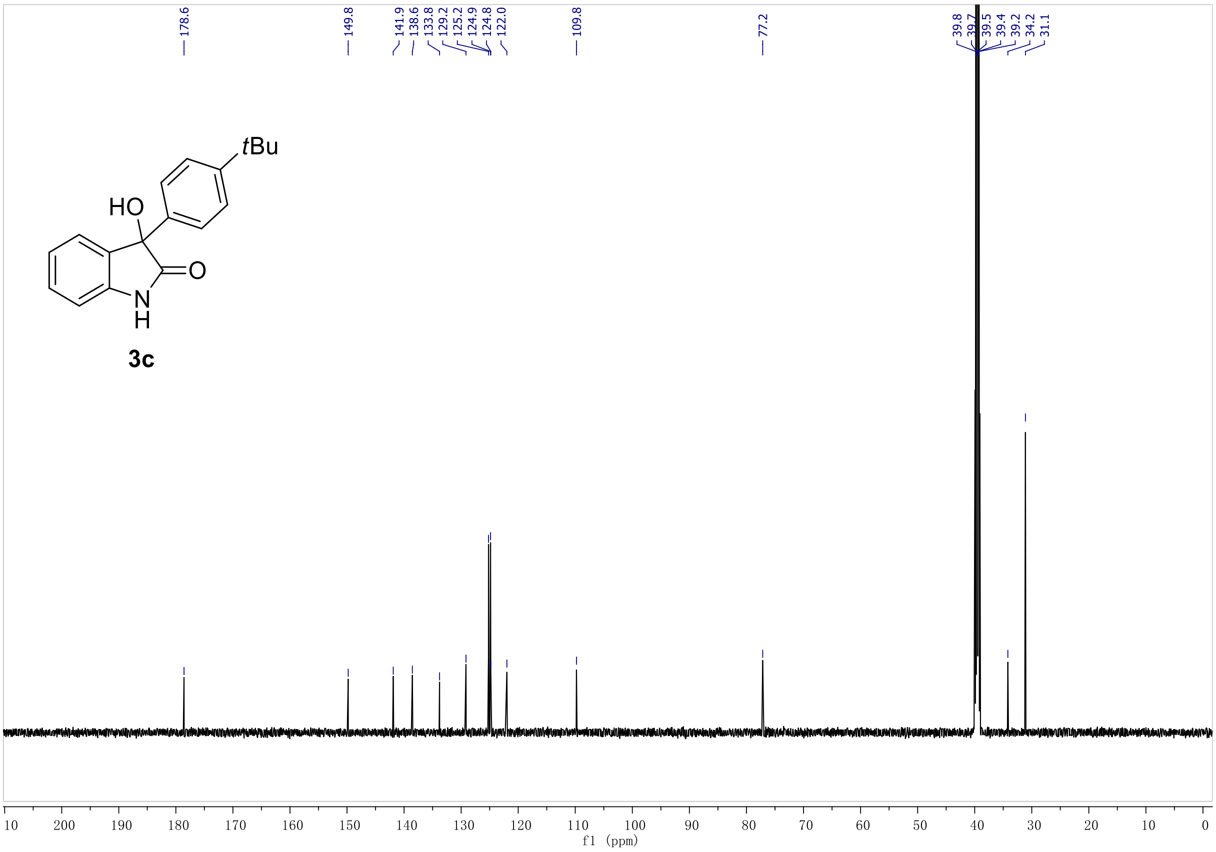


$^{13}$C{$^1$H} NMR in DMSO-$d_6$ at 150 MHz

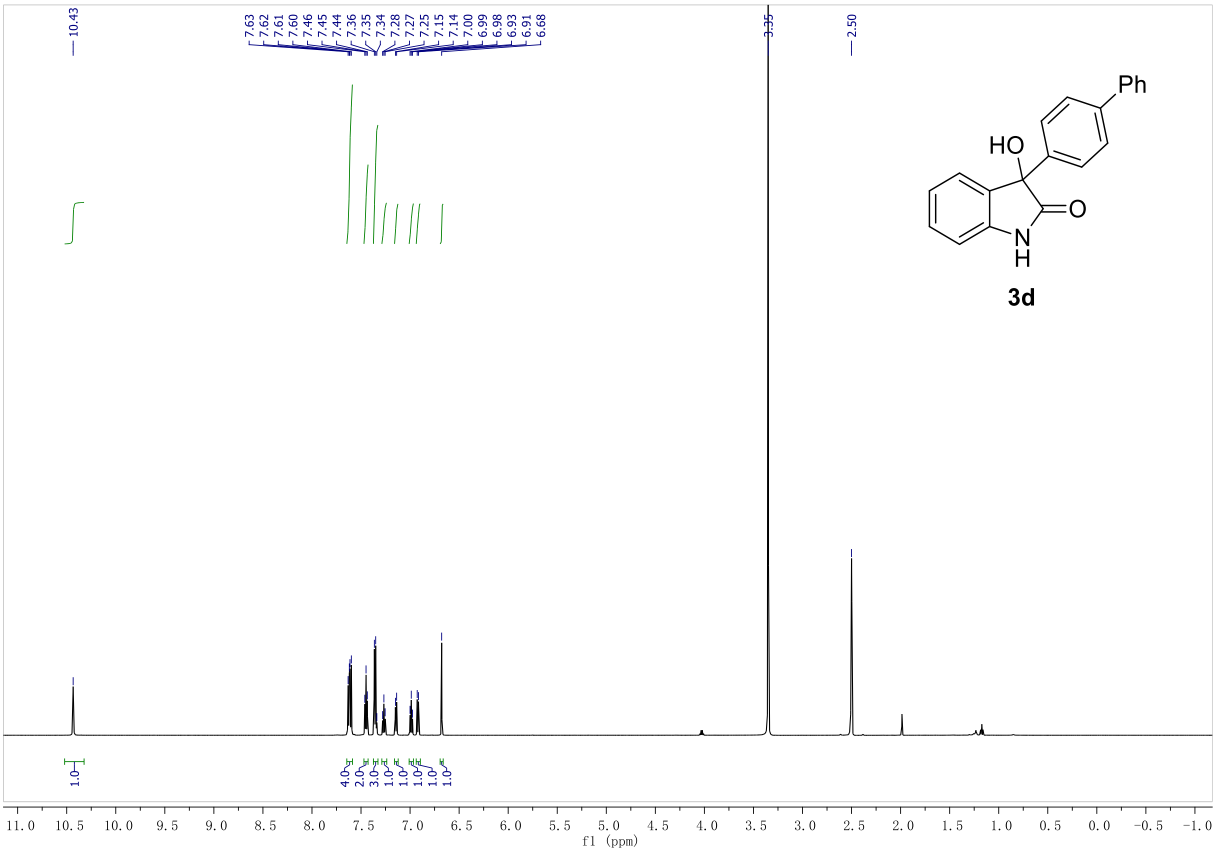


$^{1}$H NMR in DMSO-$d_6$ at 600 MHz

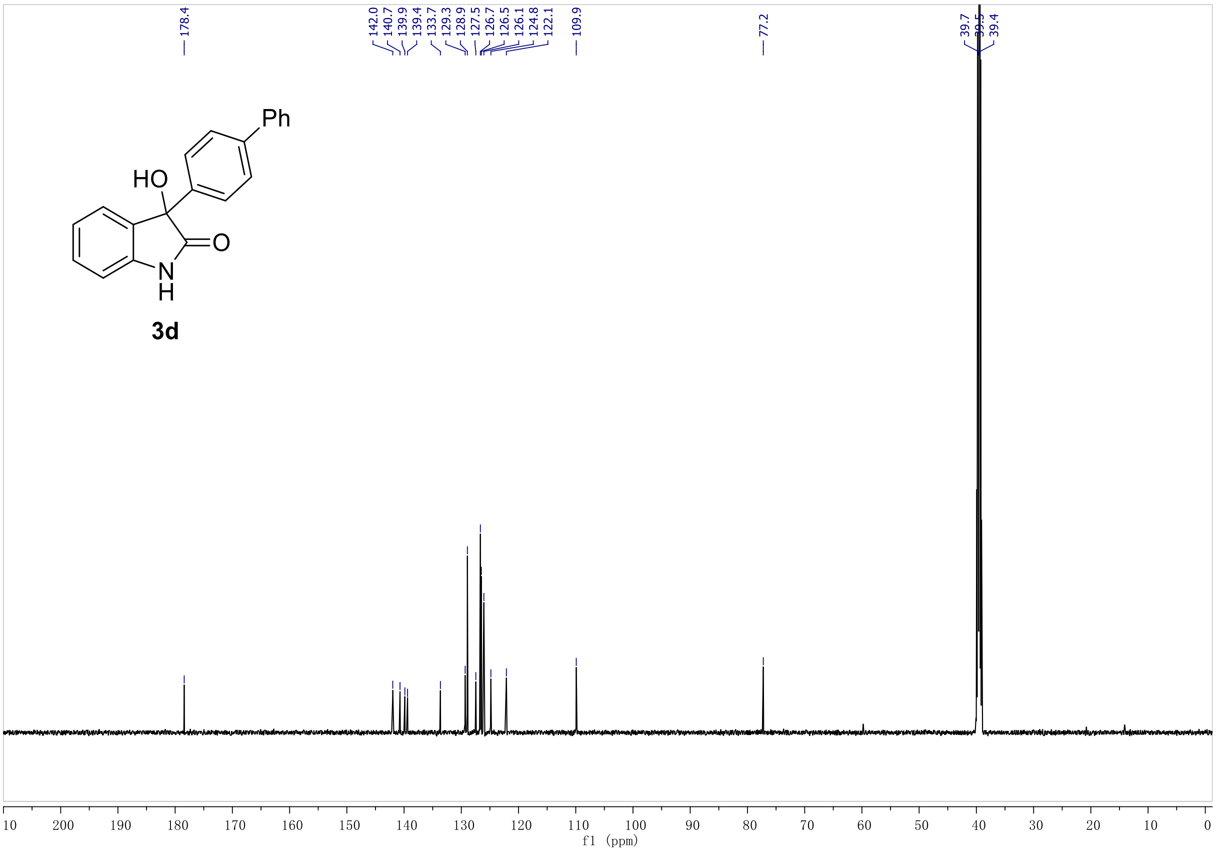


$^{13}$C{$^{1}$H} NMR in DMSO-$d_6$ at 150 MHz

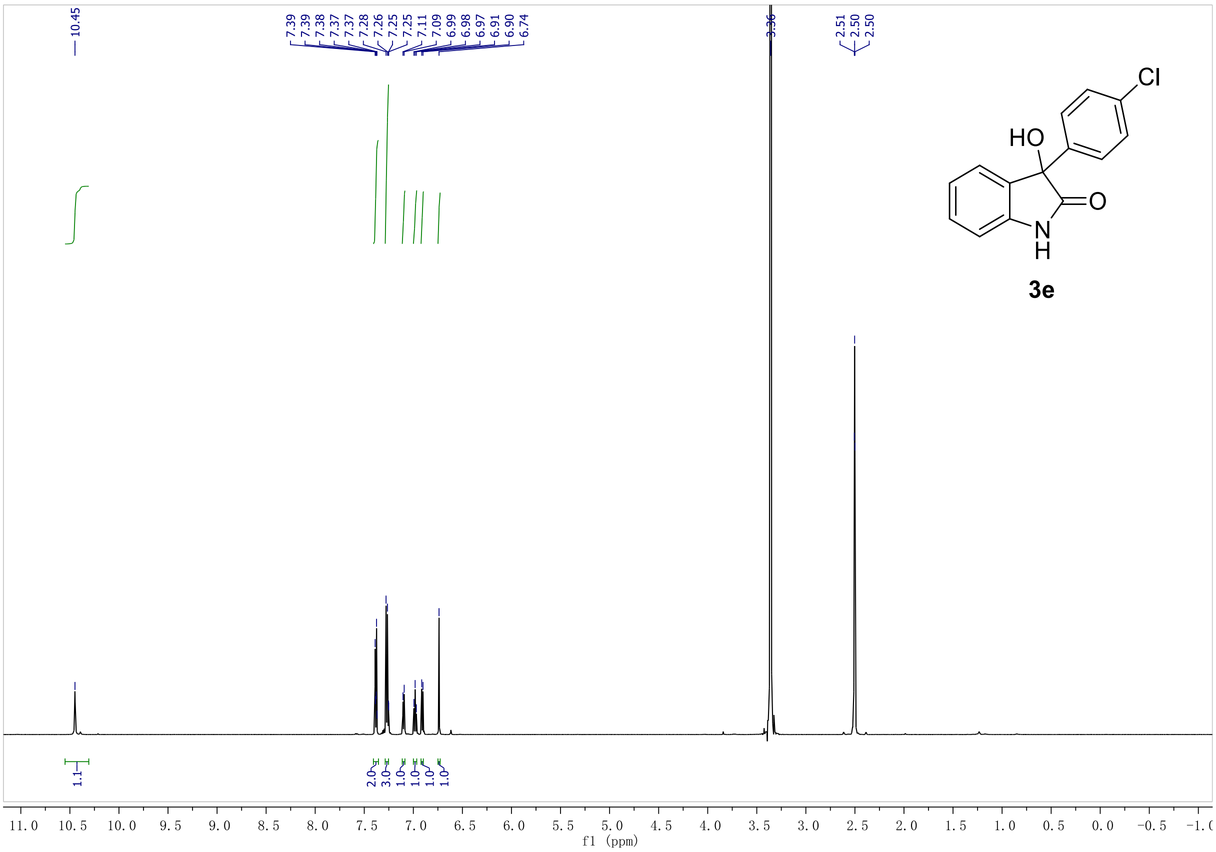


$^1$H NMR in DMSO-$d_6$ at 600 MHz

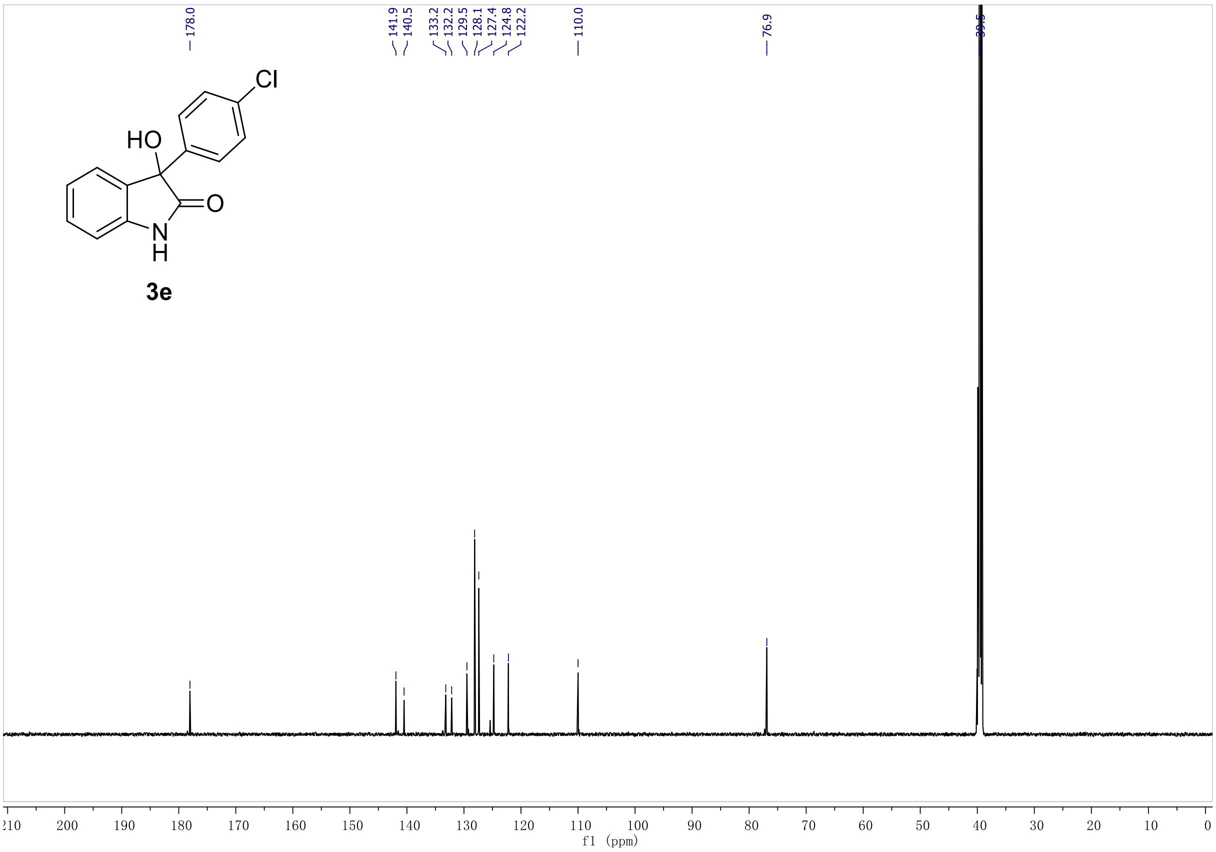


$^{13}$C{$^1$H} NMR in DMSO-$d_6$ at 150 MHz

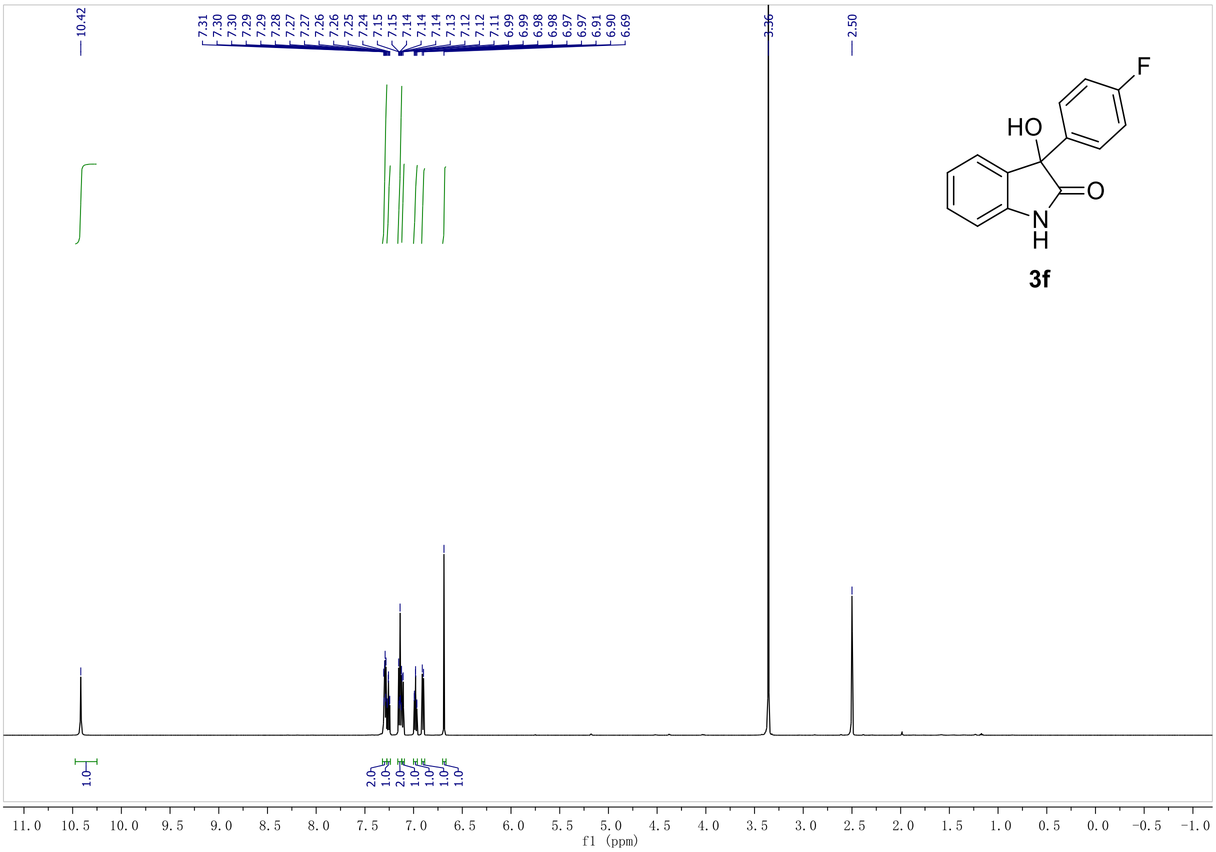

$^{1}H$ NMR in DMSO-$d_6$ at 600 MHz

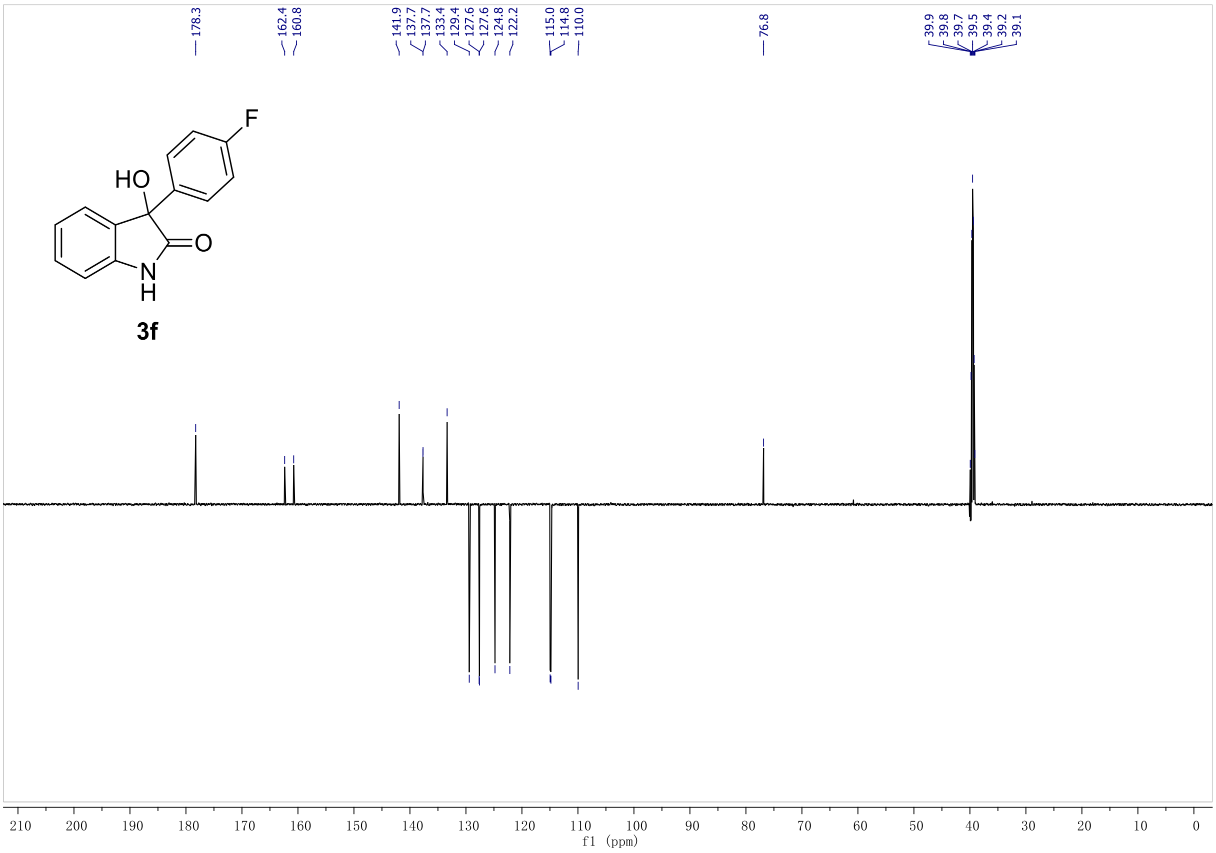

$^{13}C\{^{1}H\}$ NMR in DMSO-$d_6$ at 150 MHz

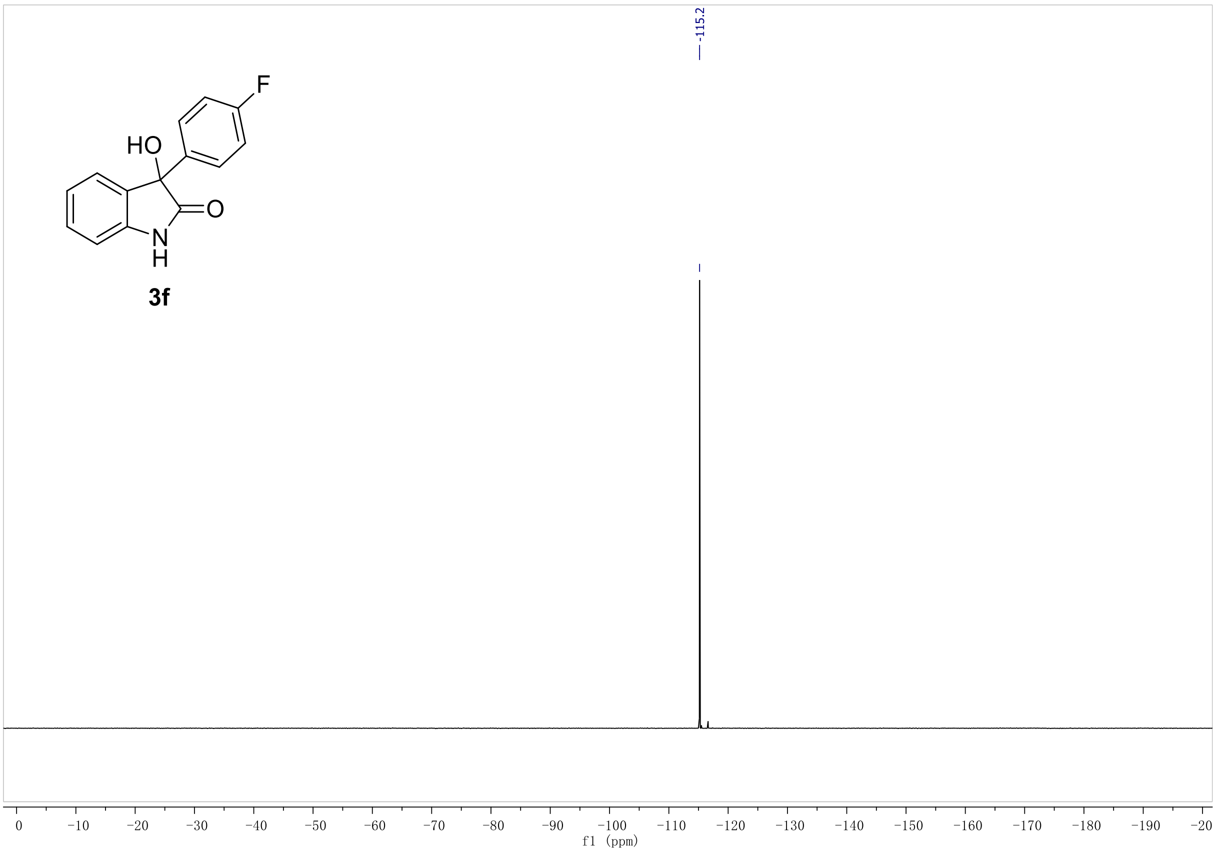


$^{19}$F NMR in DMSO-$d_6$ at 565 MHz

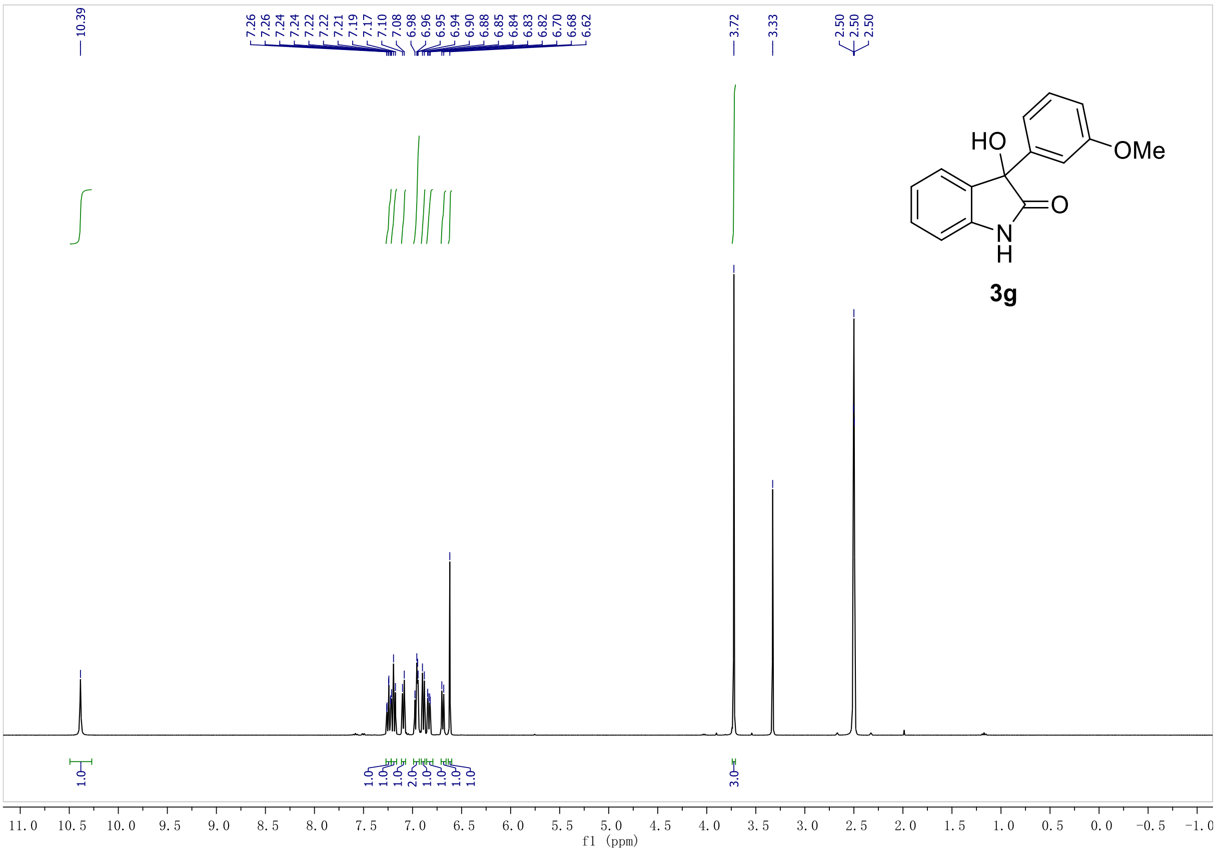

$^{1}$H NMR in DMSO-$d_6$ at 400 MHz

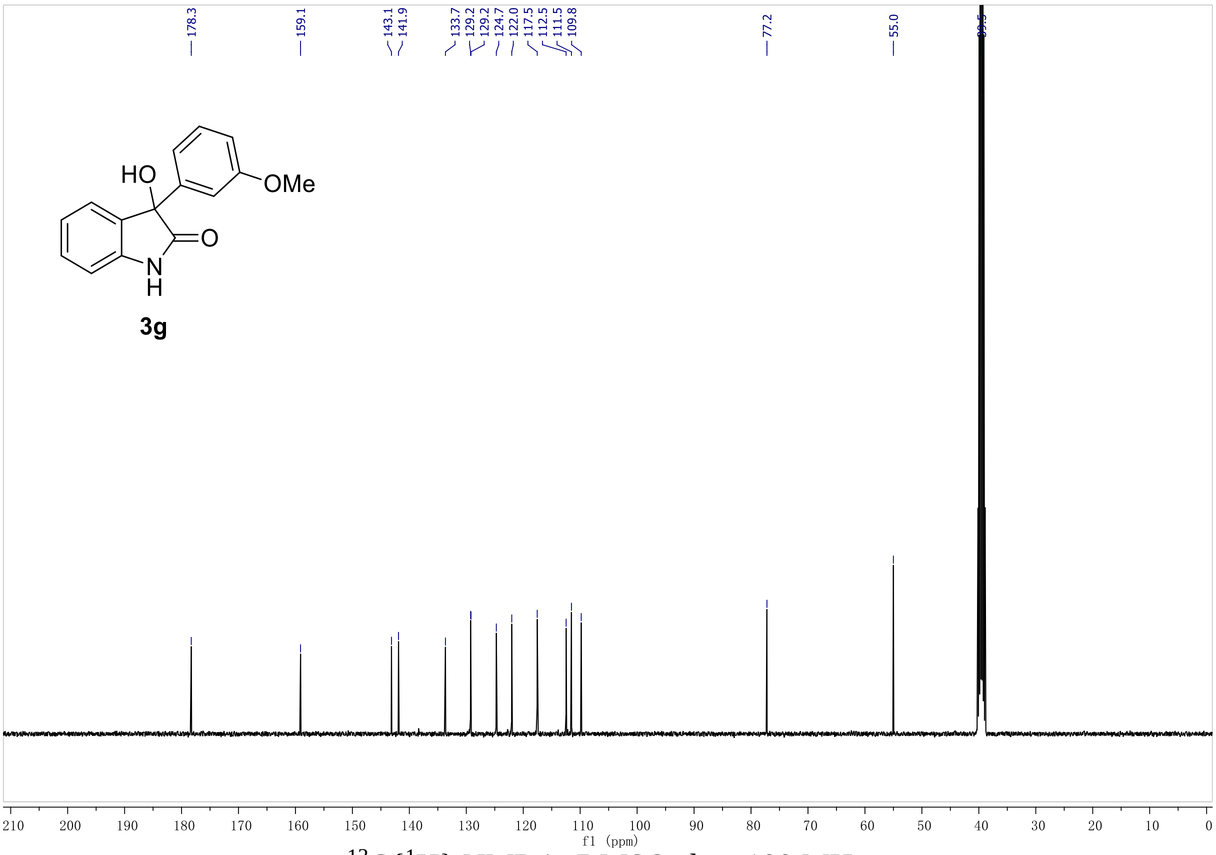

$^{13}$C{$^{1}$H} NMR in DMSO-$d_6$ at 100 MHz

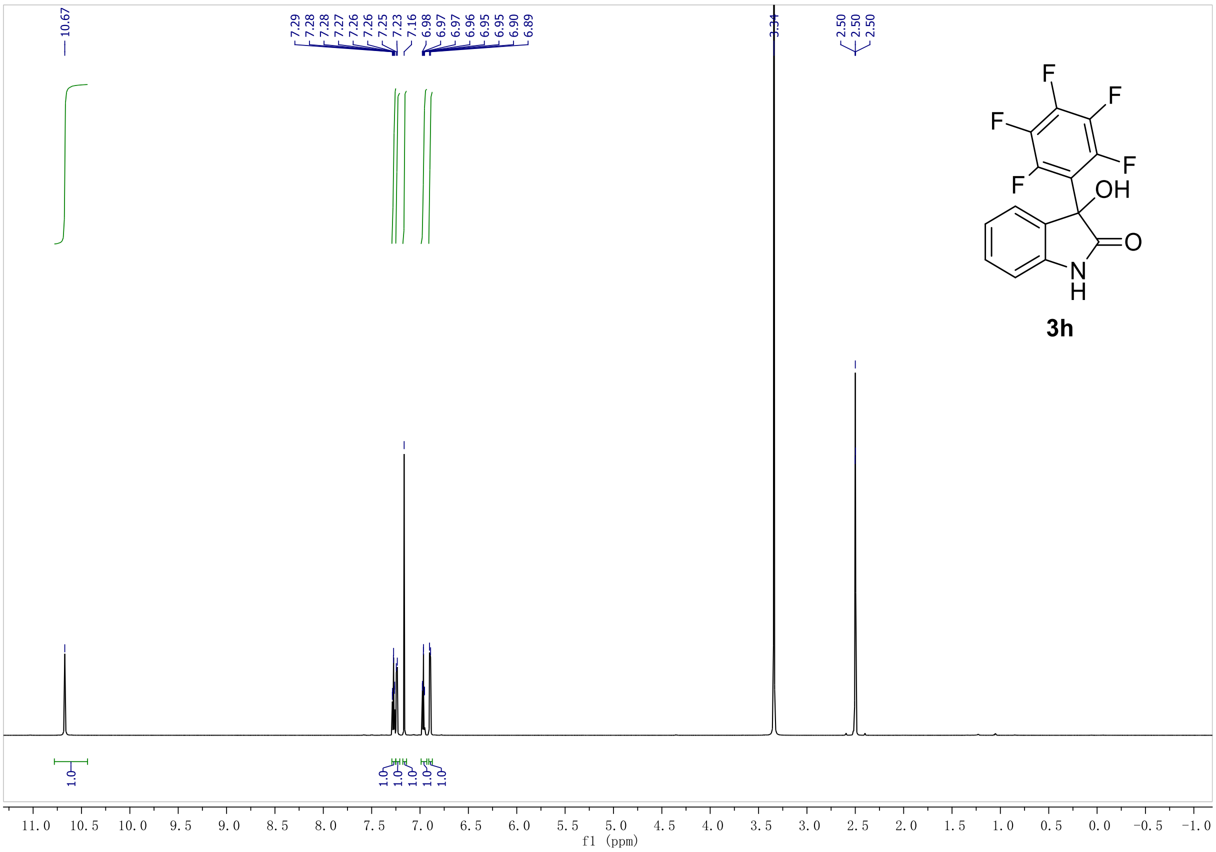


$^{1}H$ NMR in DMSO-$d_6$ at 700 MHz

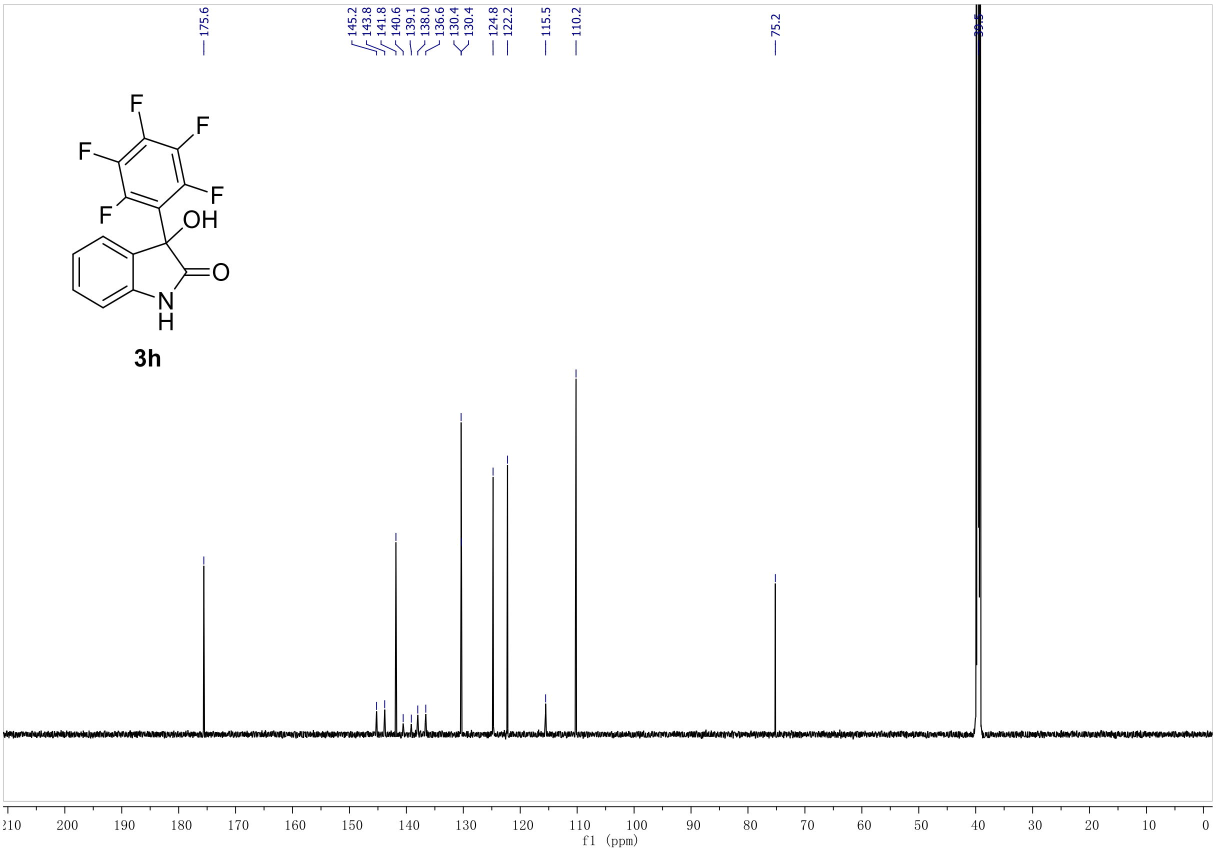


$^{13}C\{^{1}H\}$ NMR in DMSO-$d_6$ at 175 MHz

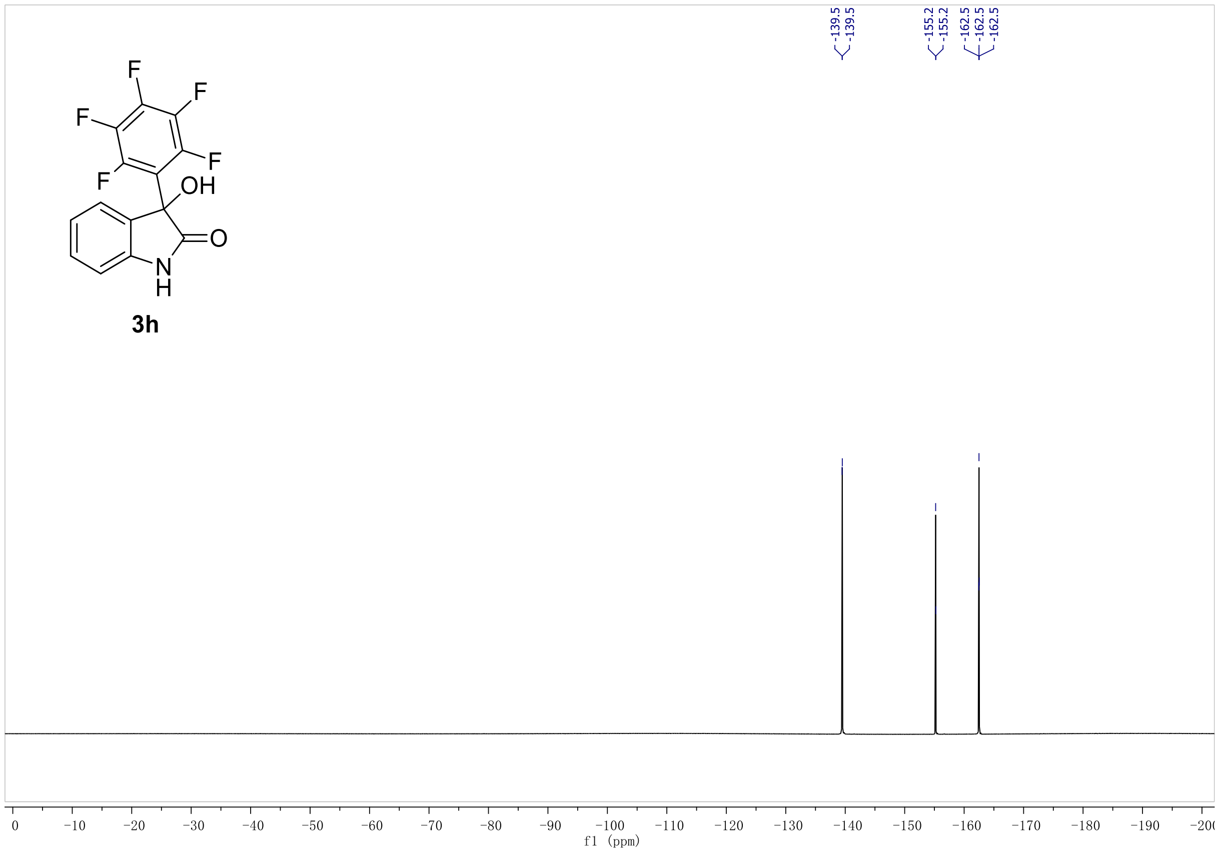


$^{19}$F NMR in DMSO-$d_6$ at 659 MHz

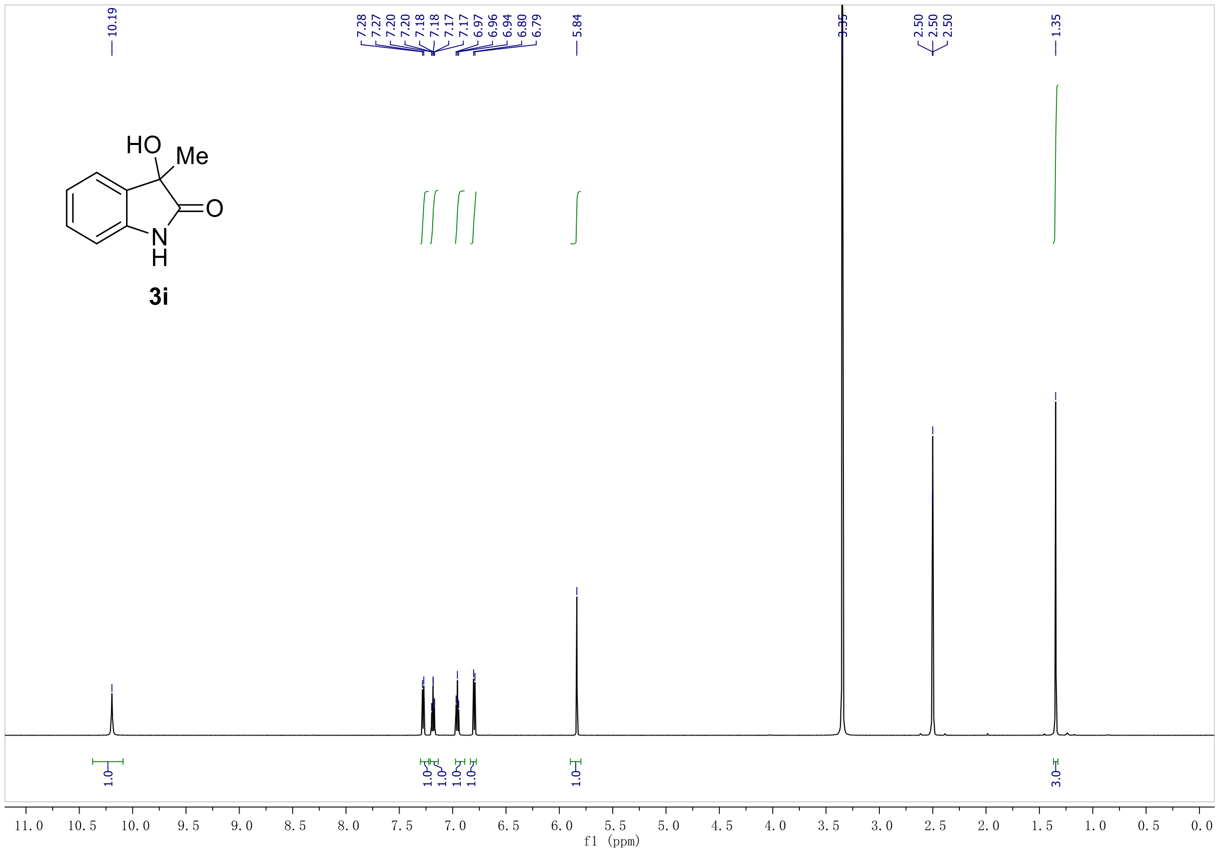

$^{1}$H NMR in DMSO-$d_6$ at 600 MHz

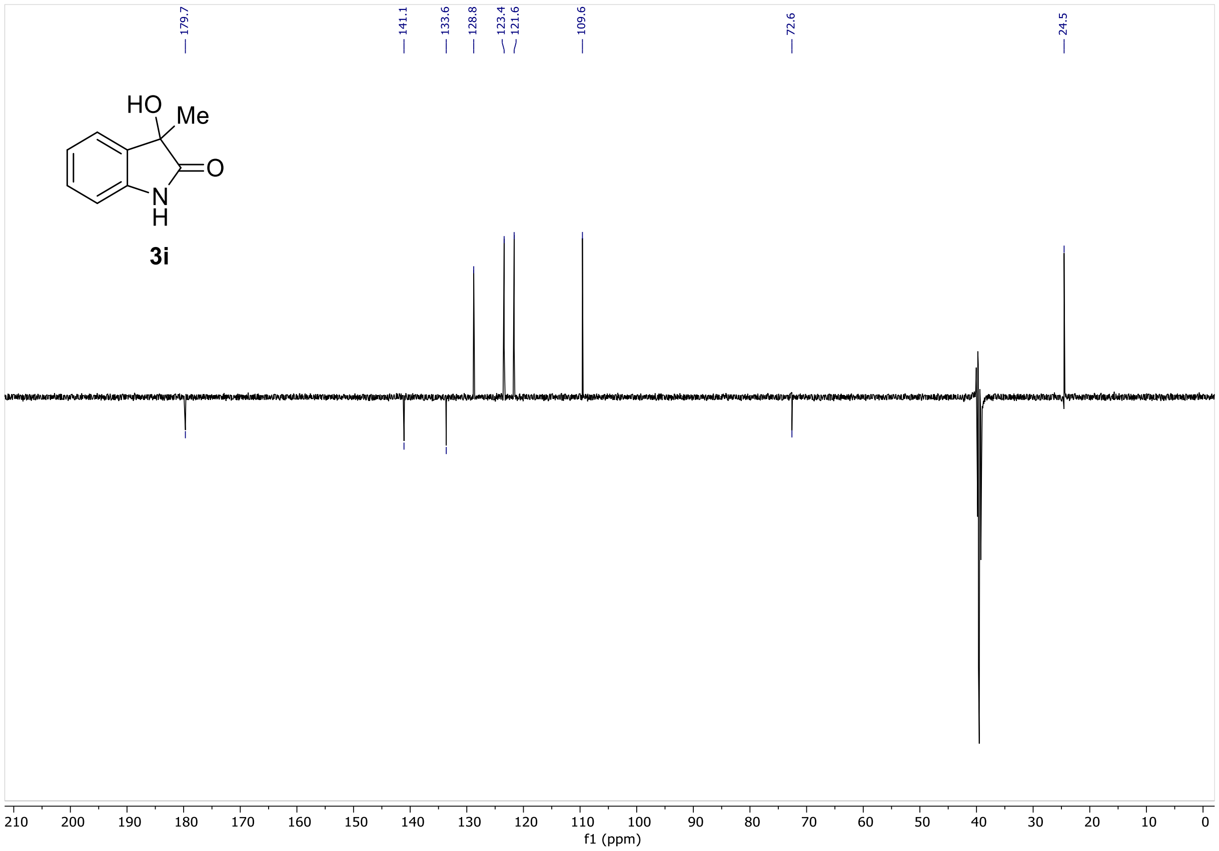

$^{13}$C{$^{1}$H} NMR in DMSO-$d_6$ at 150 MHz

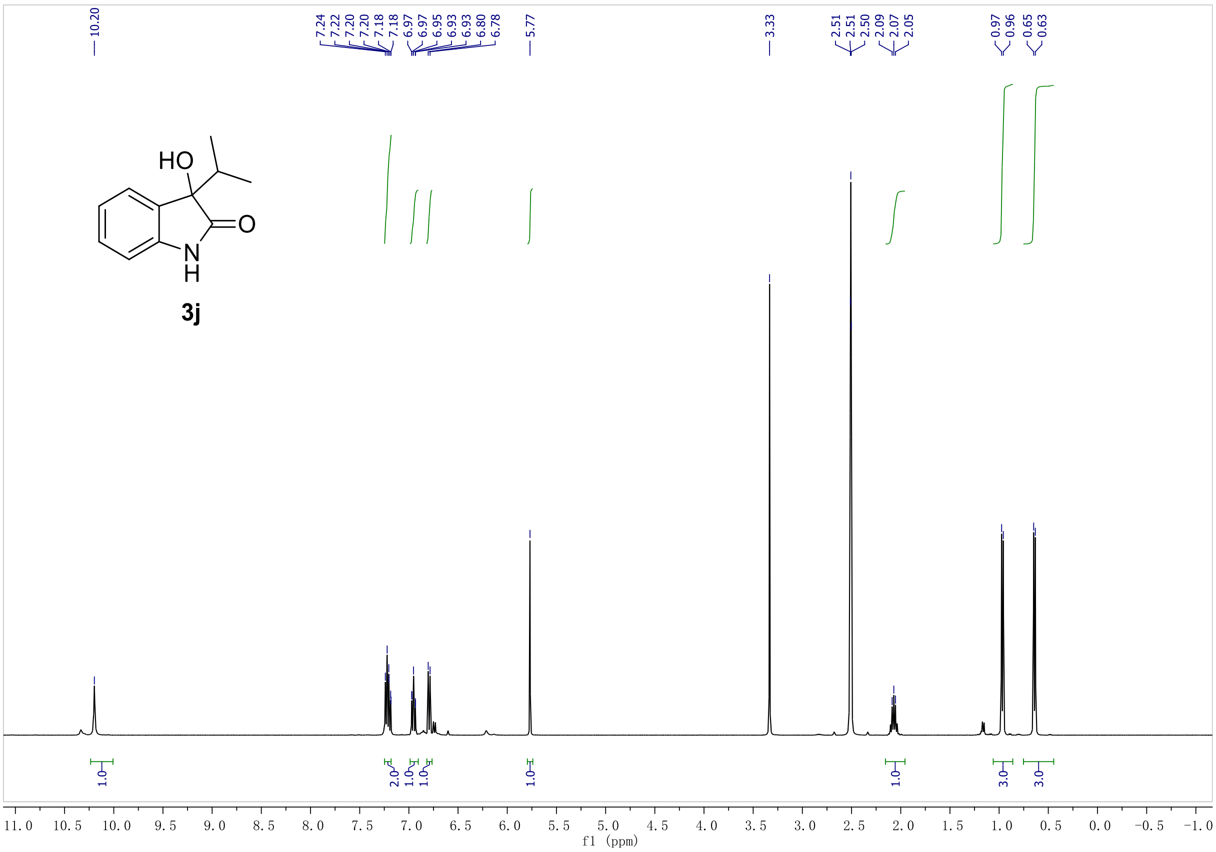


$^{1}$H NMR in DMSO-$d_6$ at 400 MHz

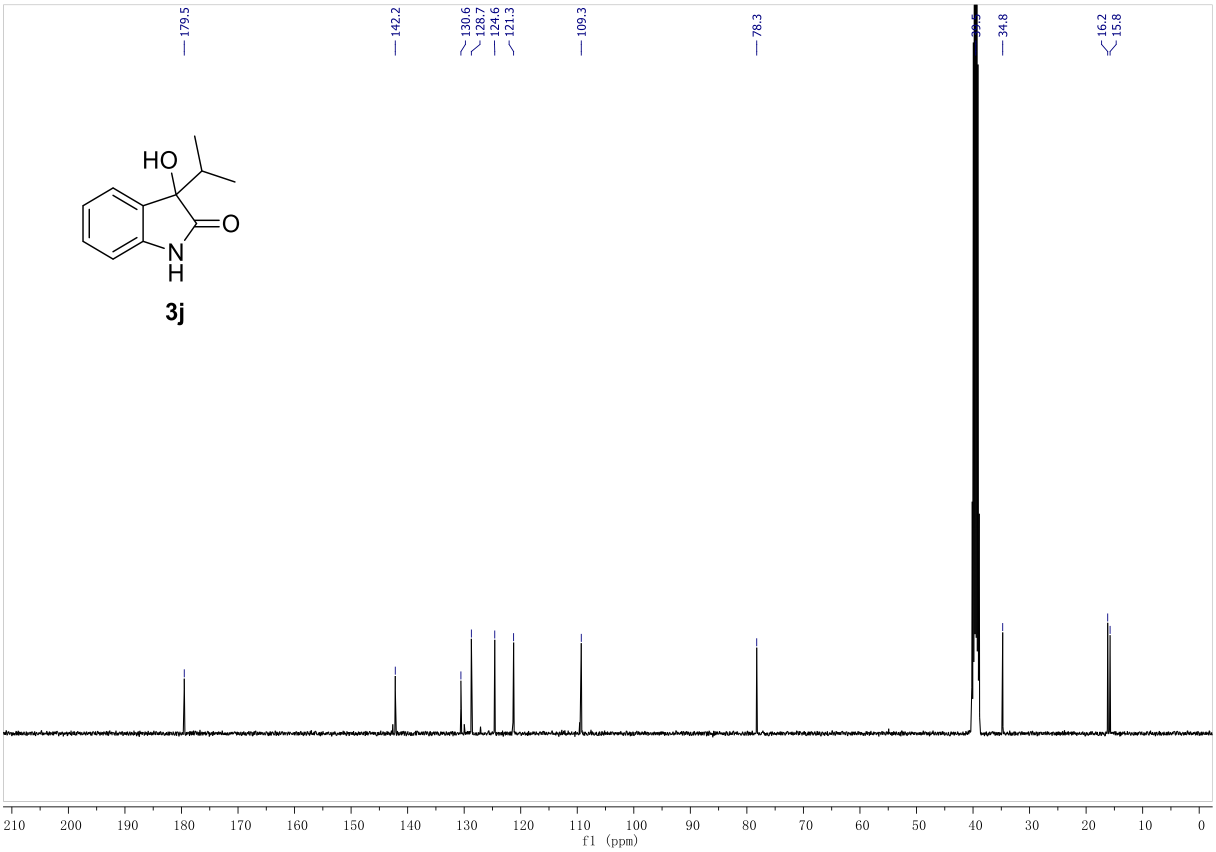


$^{13}$C{$^{1}$H} NMR in DMSO-$d_6$ at 100 MHz

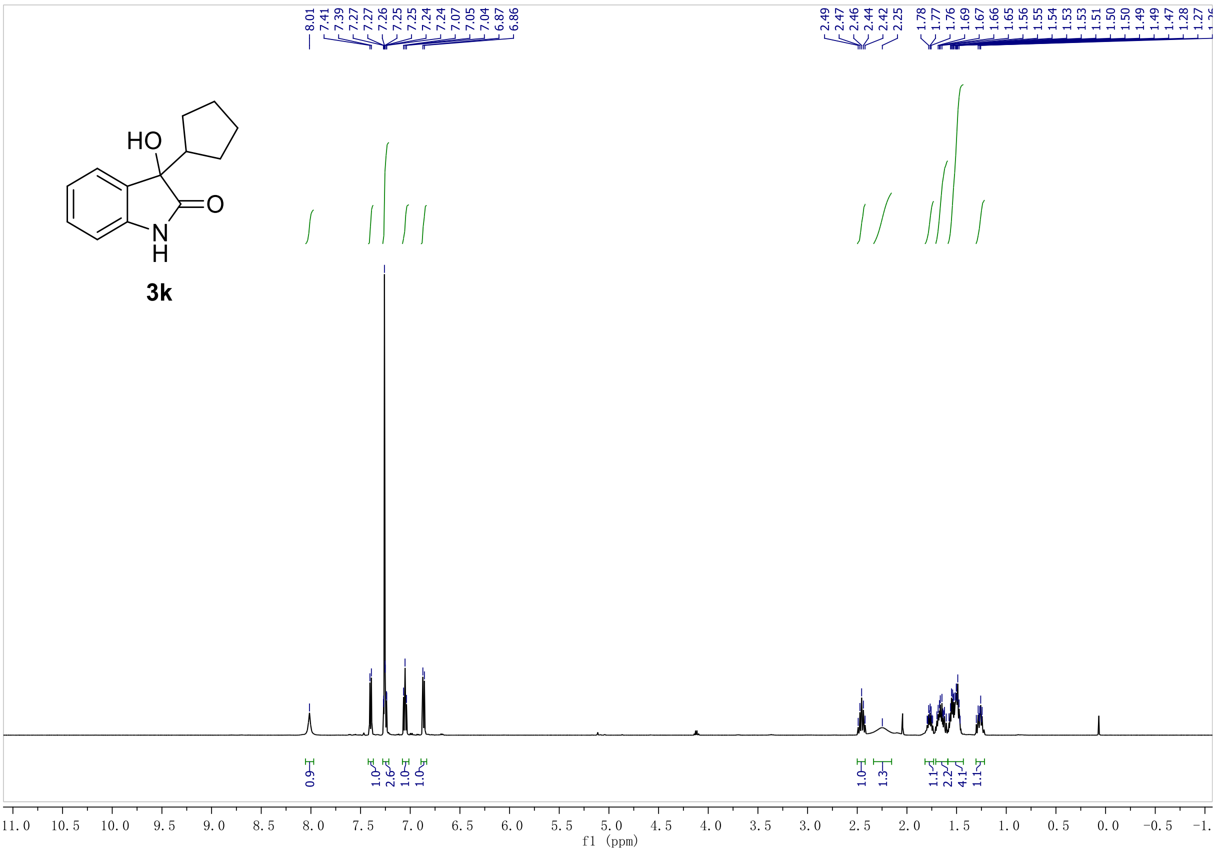


$^{1}H$ NMR in $CDCl_3$ at 400 MHz

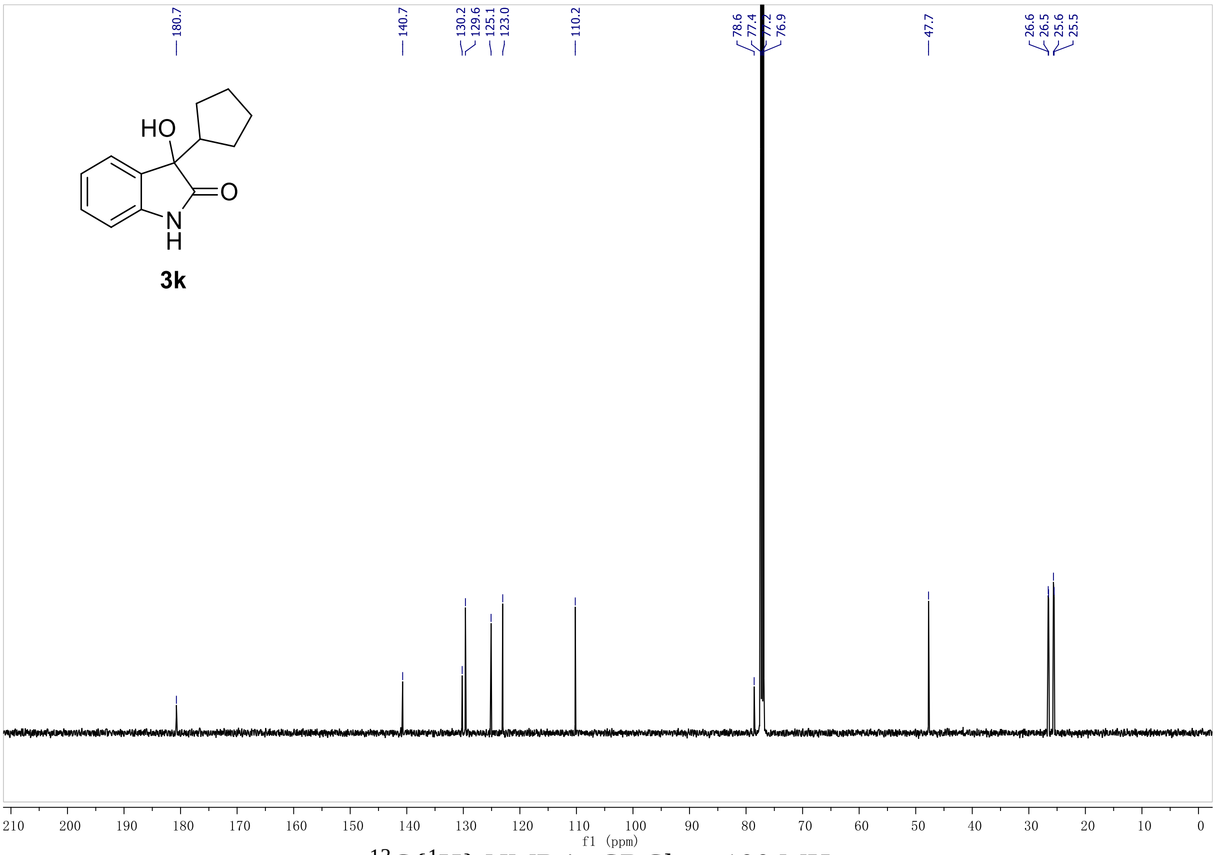


$^{13}C\{^{1}H\}$ NMR in $CDCl_3$ at 100 MHz

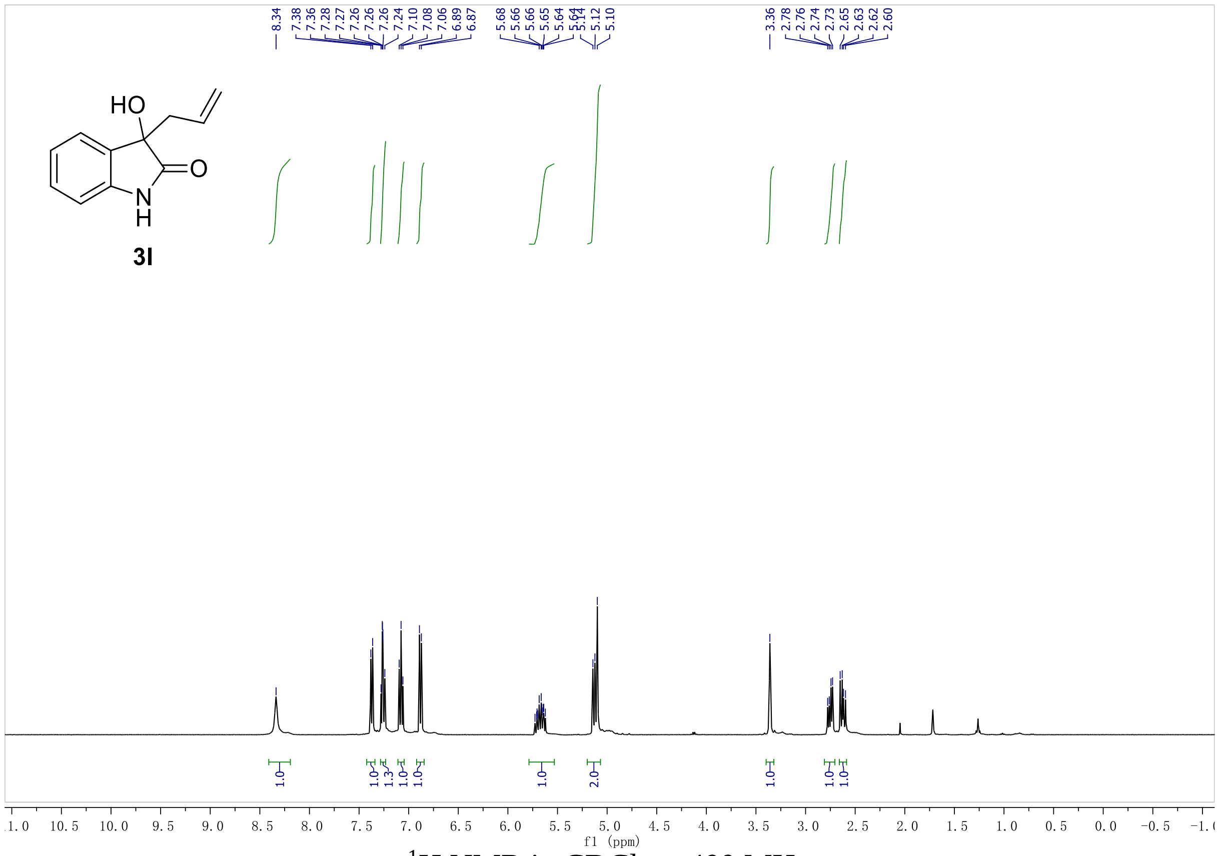

$^{1}H$ NMR in $CDCl_3$ at 400 MHz

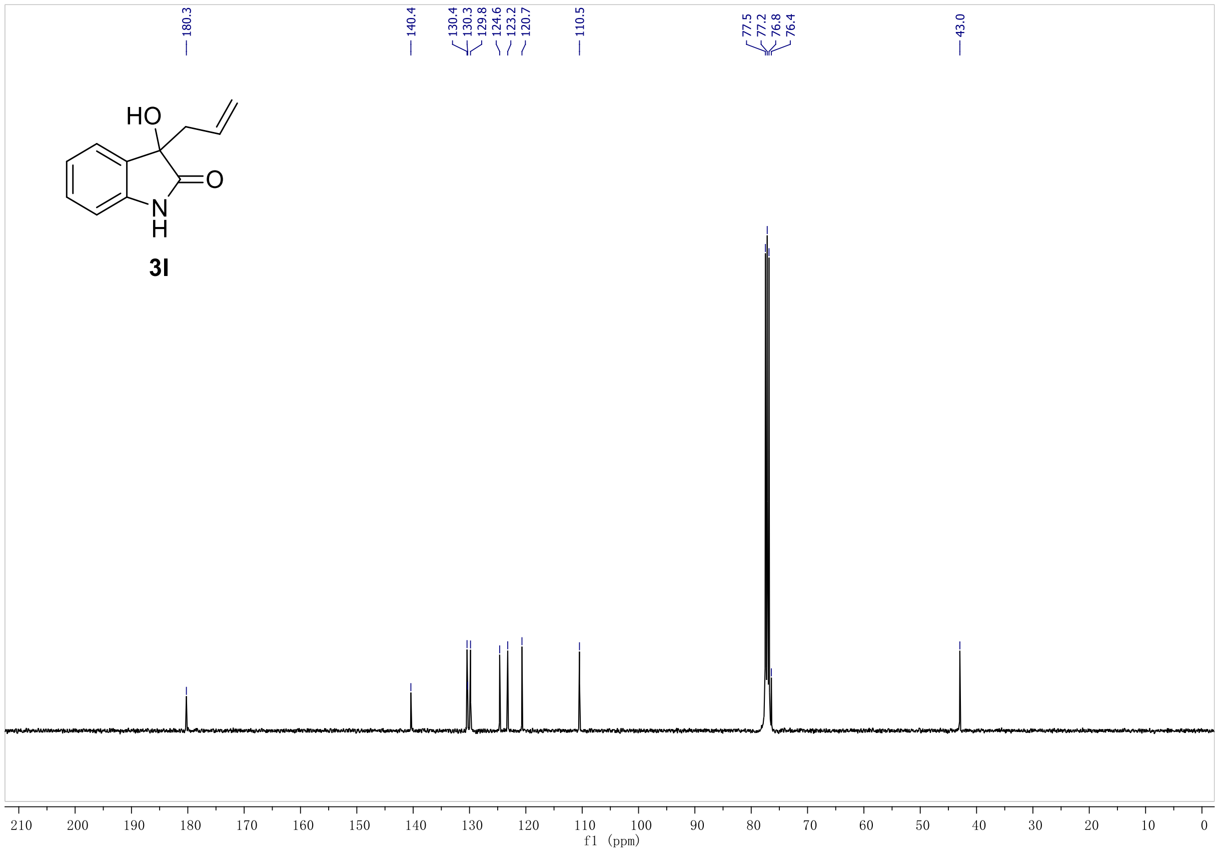

$^{13}C\{^{1}H\}$ NMR in $CDCl_3$ at 400 MHz

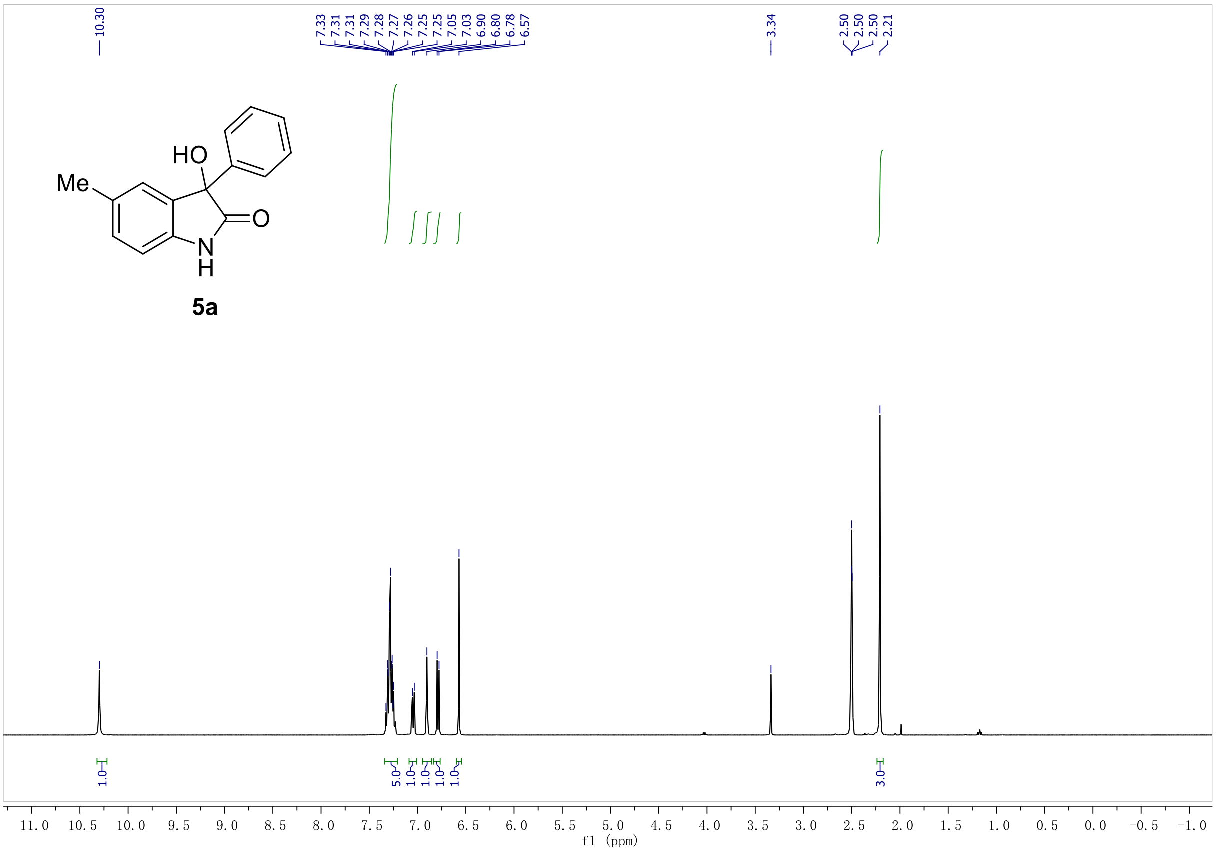


$^{1}H$ NMR in DMSO-$d_6$ at 400 MHz

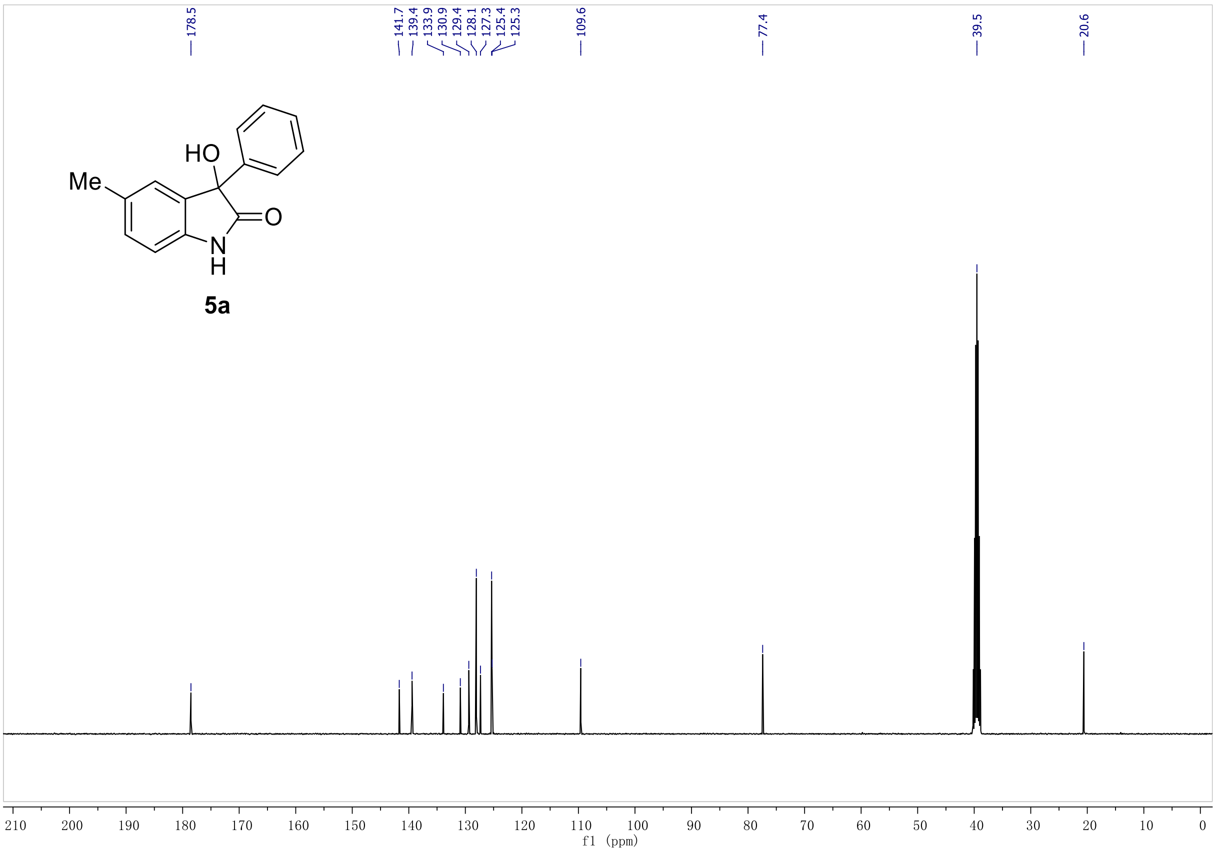


$^{13}C\{^{1}H\}$ NMR in DMSO-$d_6$ at 100 MHz

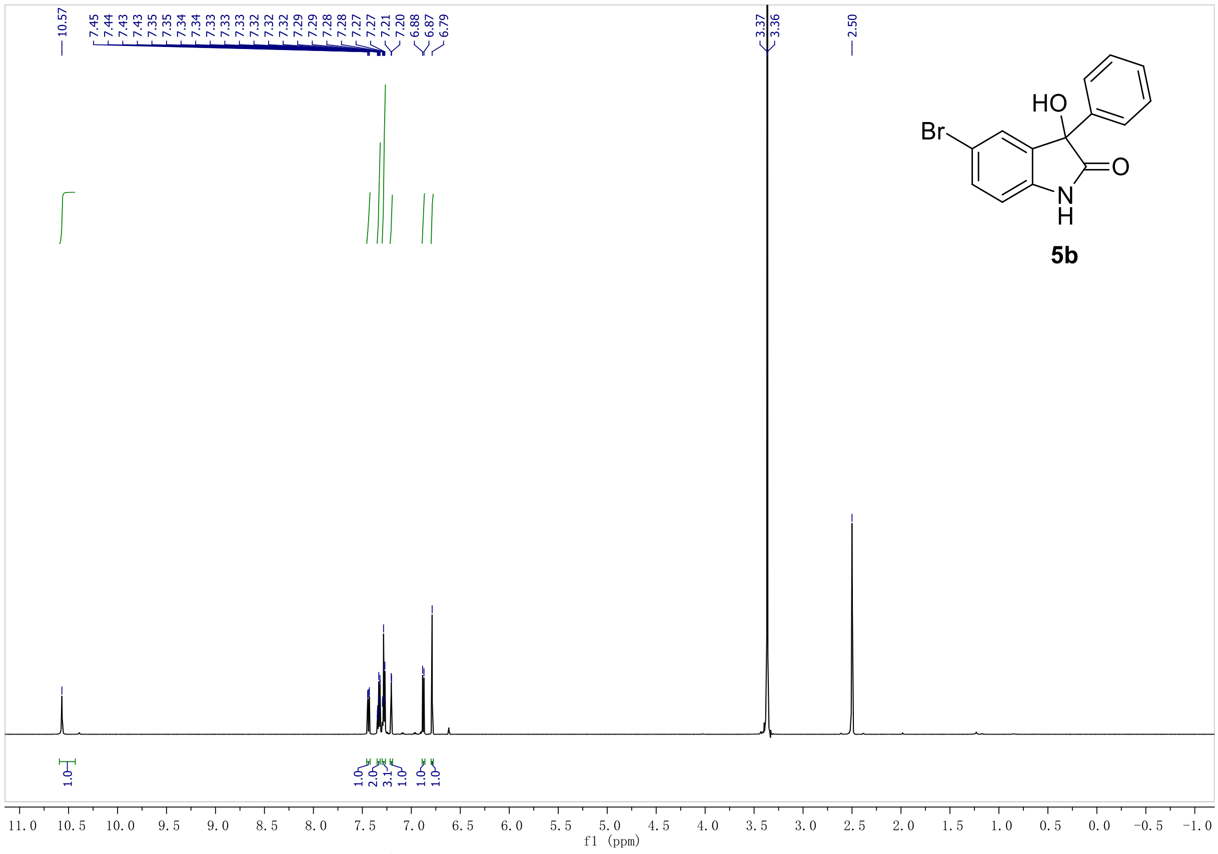


$^{1}H$ NMR in DMSO-$d_6$ at 600 MHz

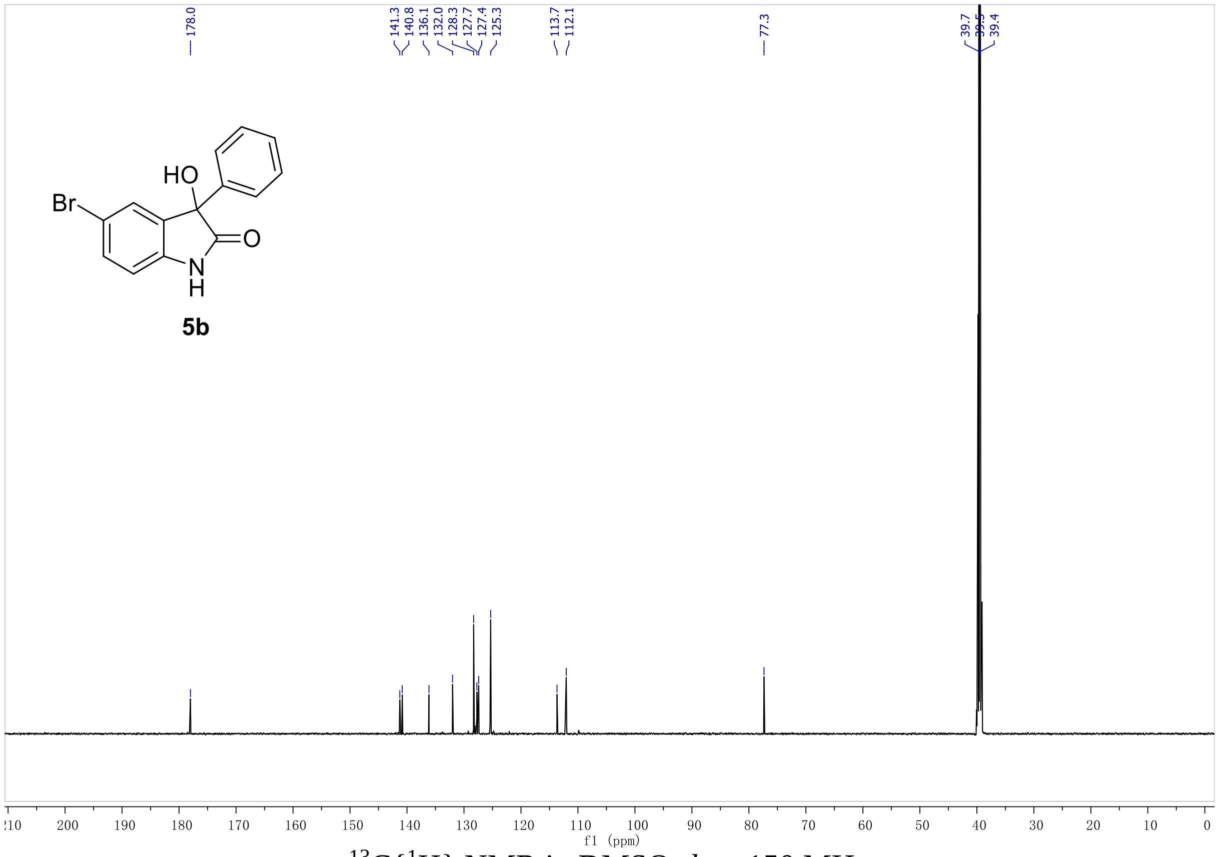


$^{13}C\{^{1}H\}$ NMR in DMSO-$d_6$ at 150 MHz

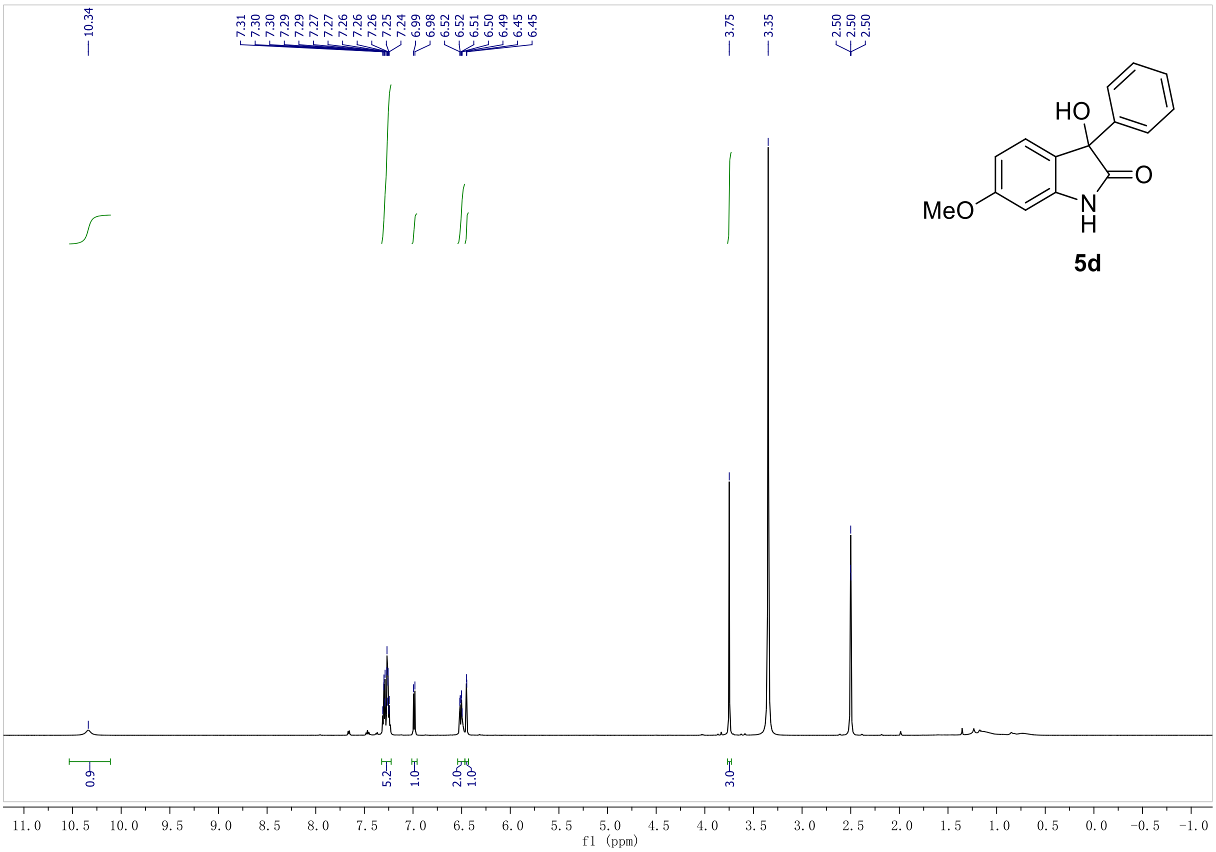


$^{1}H$ NMR in DMSO-$d_6$ at 600 MHz

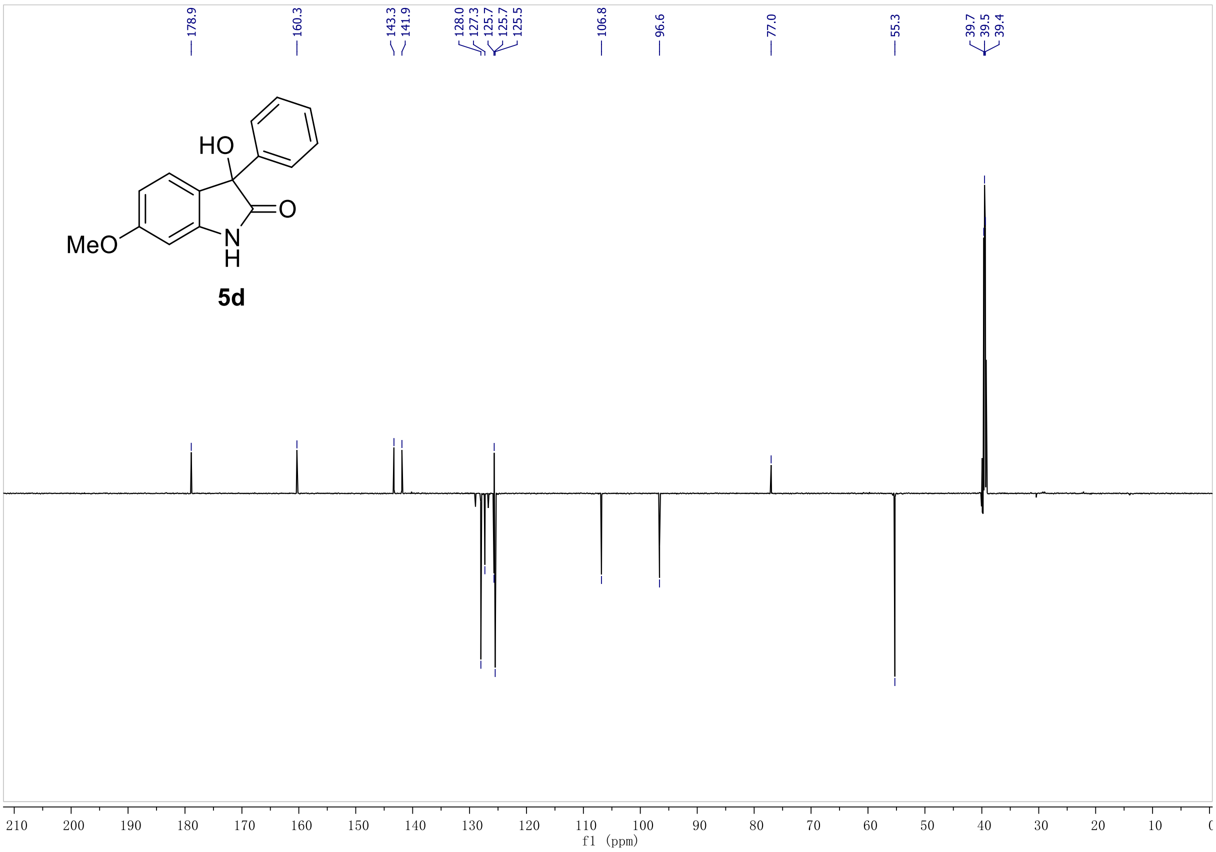


$^{13}C\{^{1}H\}$ NMR in DMSO-$d_6$ at 150 MHz

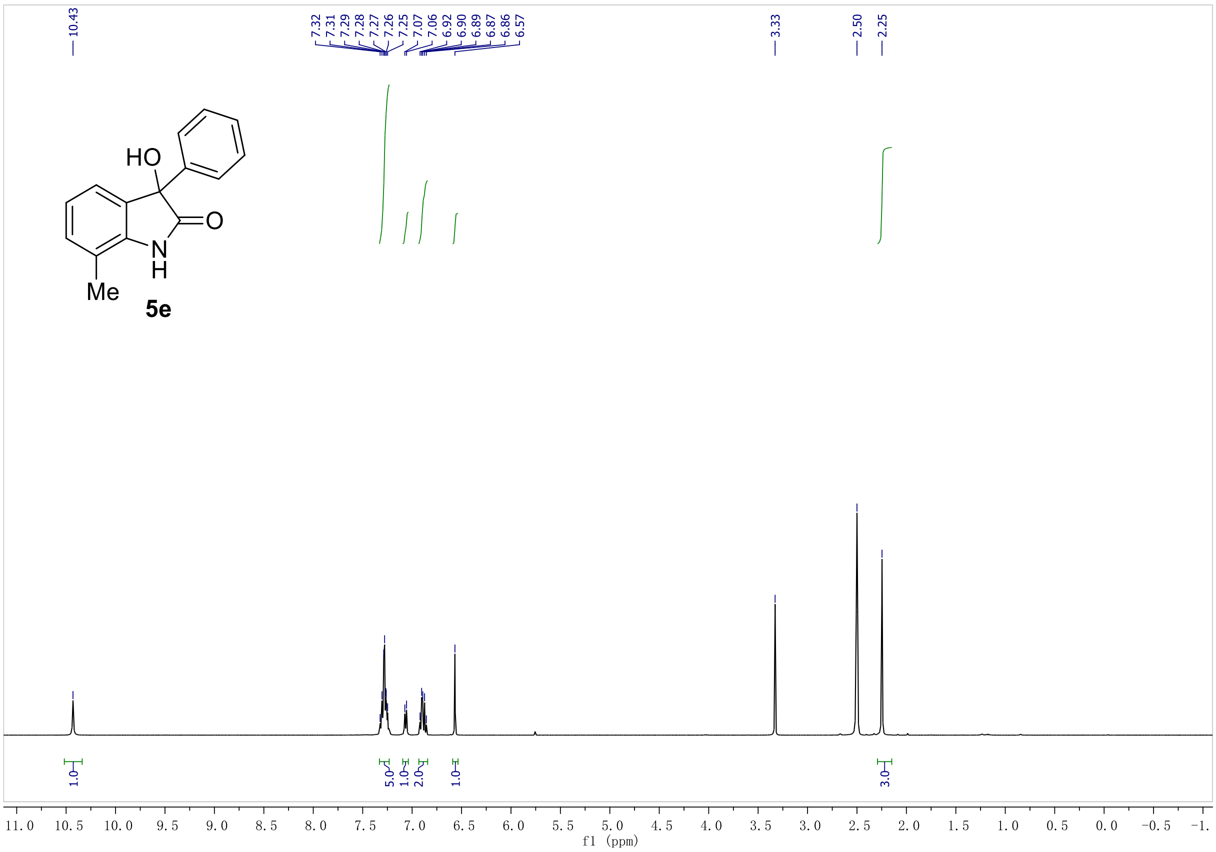


$^{1}H$ NMR in DMSO-$d_6$ at 400 MHz

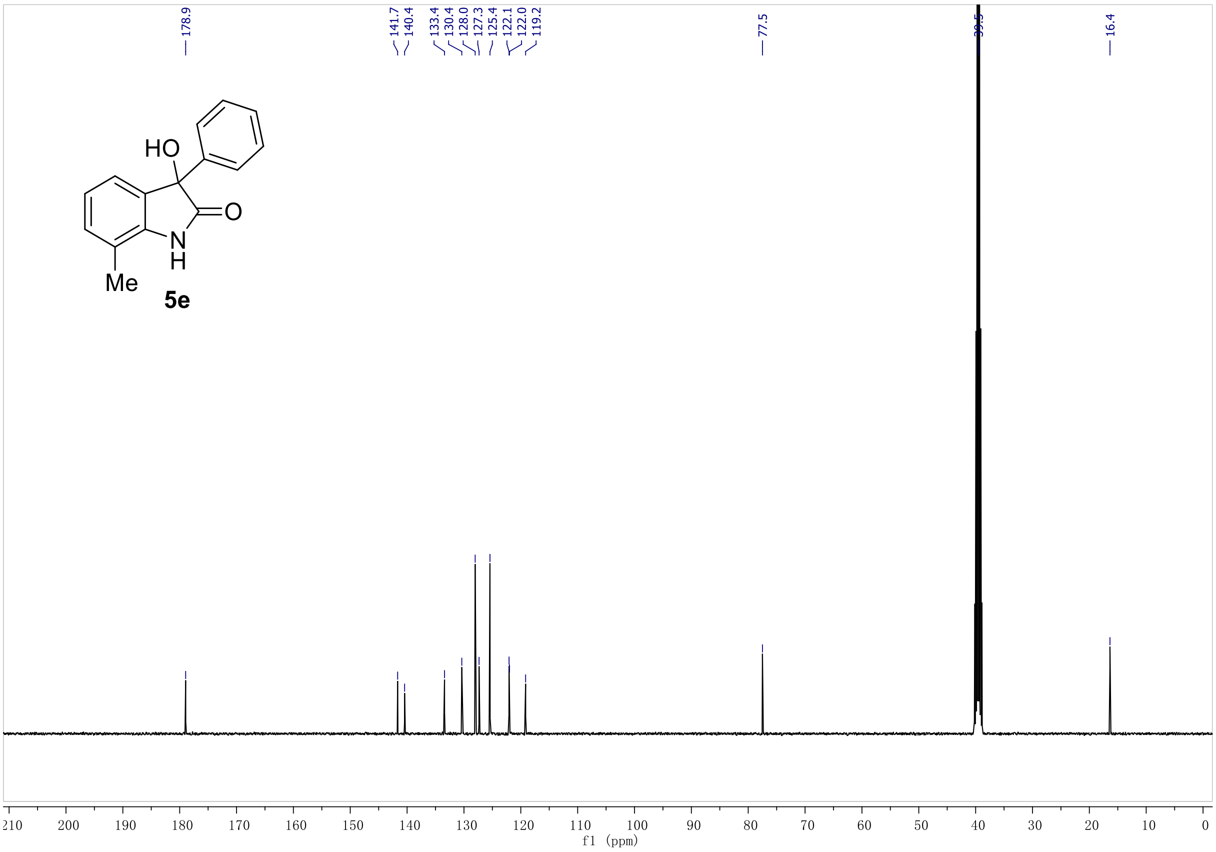


$^{13}C\{^{1}H\}$ NMR in DMSO-$d_6$ at 100 MHz

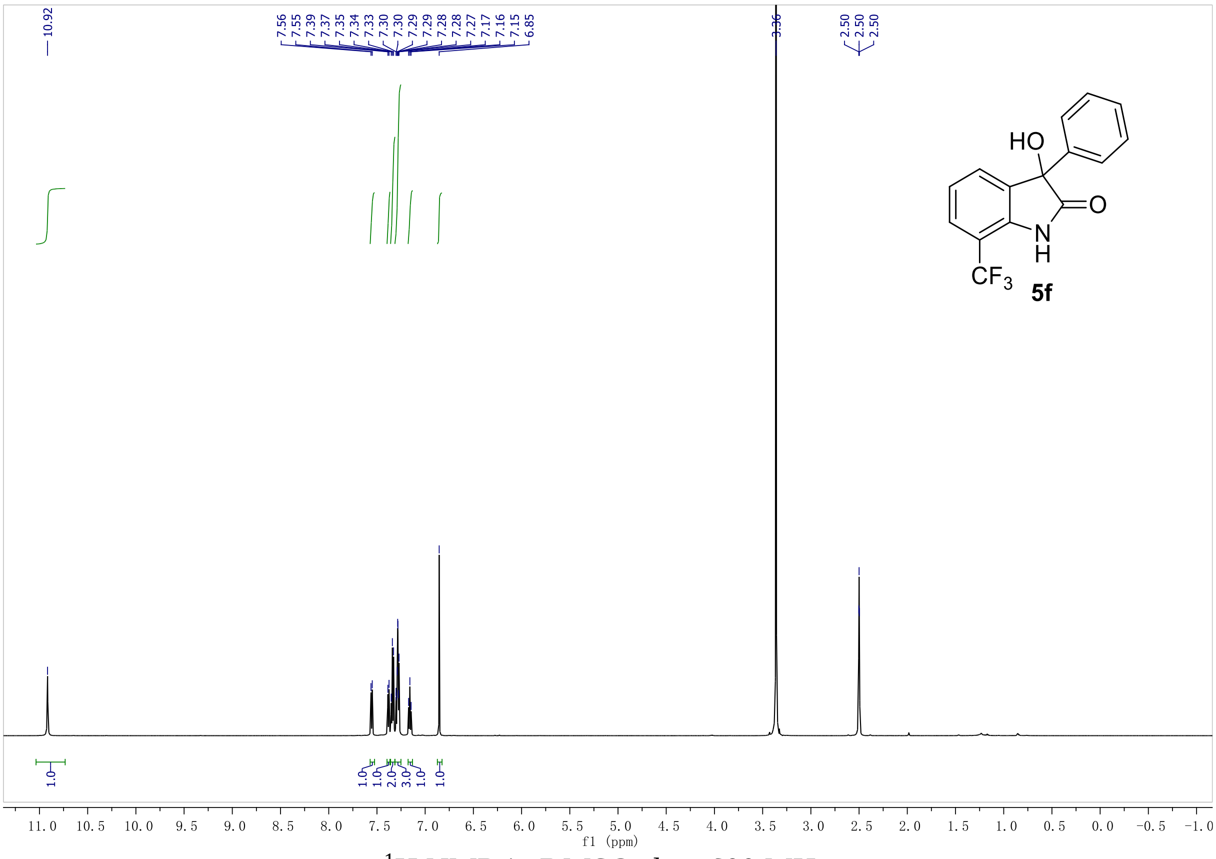


$^{1}$H NMR in DMSO-$d_6$ at 600 MHz

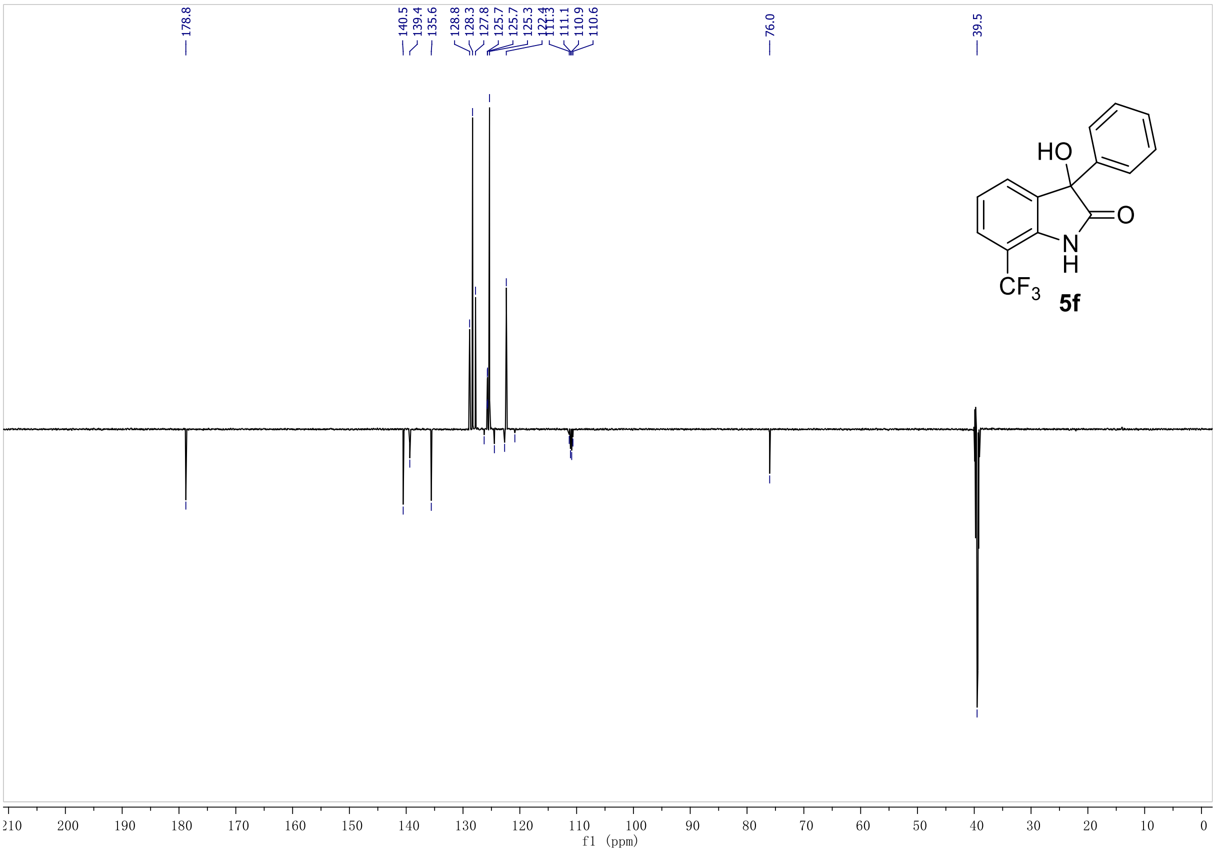


$^{13}$C{$^{1}$H} NMR in DMSO-$d_6$ at 150 MHz

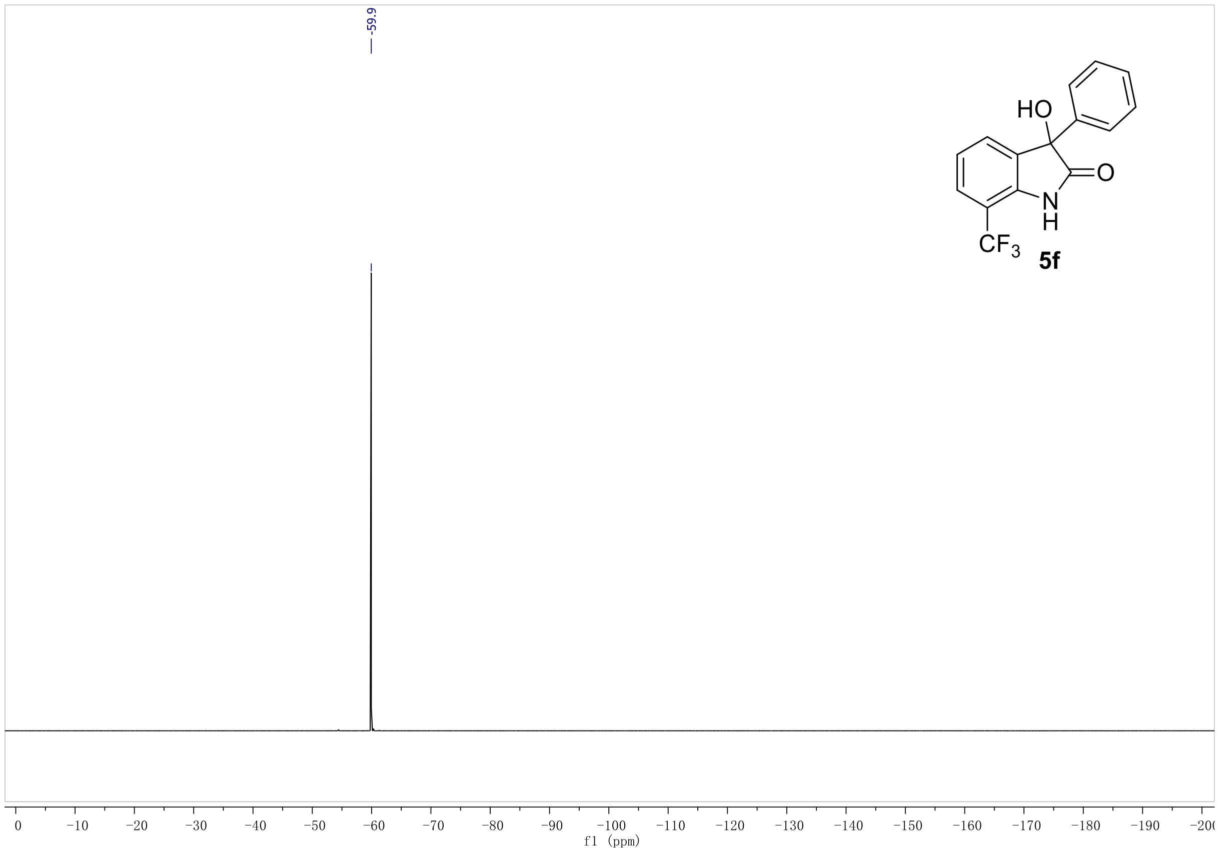


$^{19}$F NMR in DMSO-$d_6$ at 565 MHz

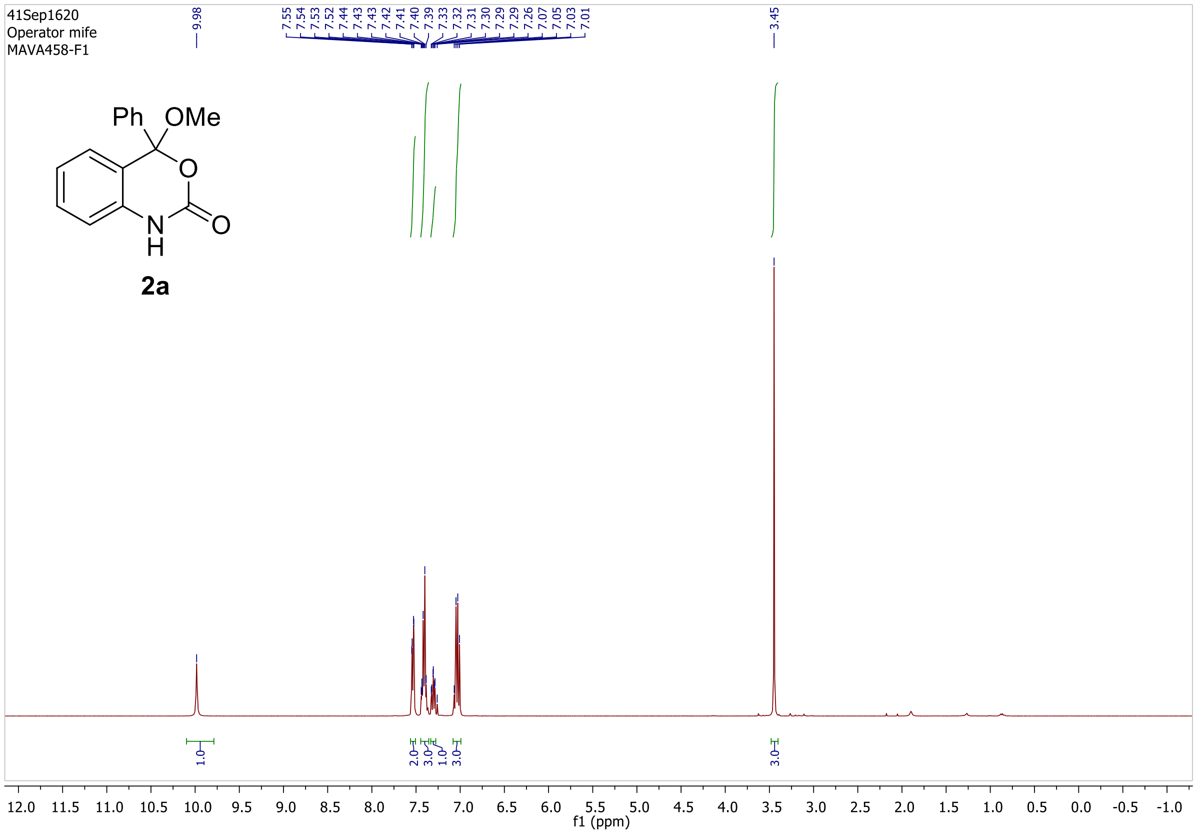


$^{1}$H NMR in $CDCl_3$ at 400 MHz

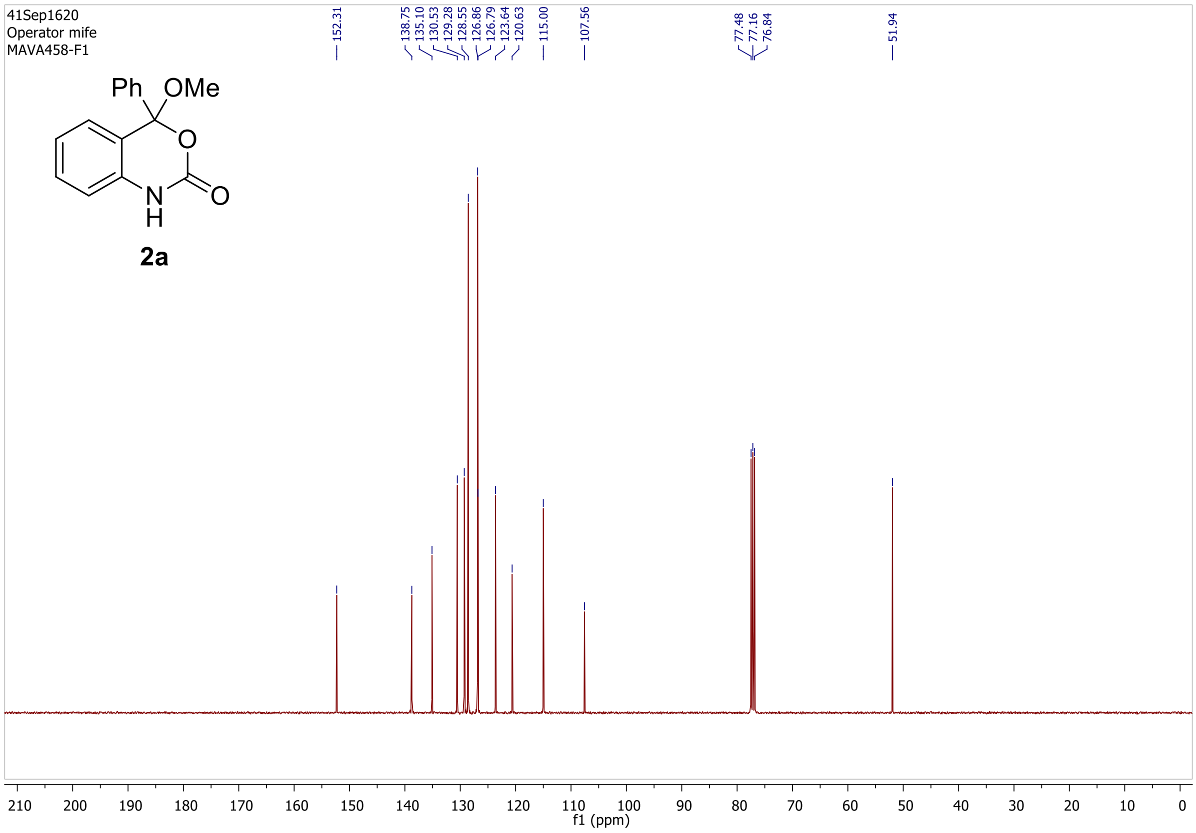


$^{13}$C{$^{1}$H} NMR in $CDCl_3$ at 400 MHz

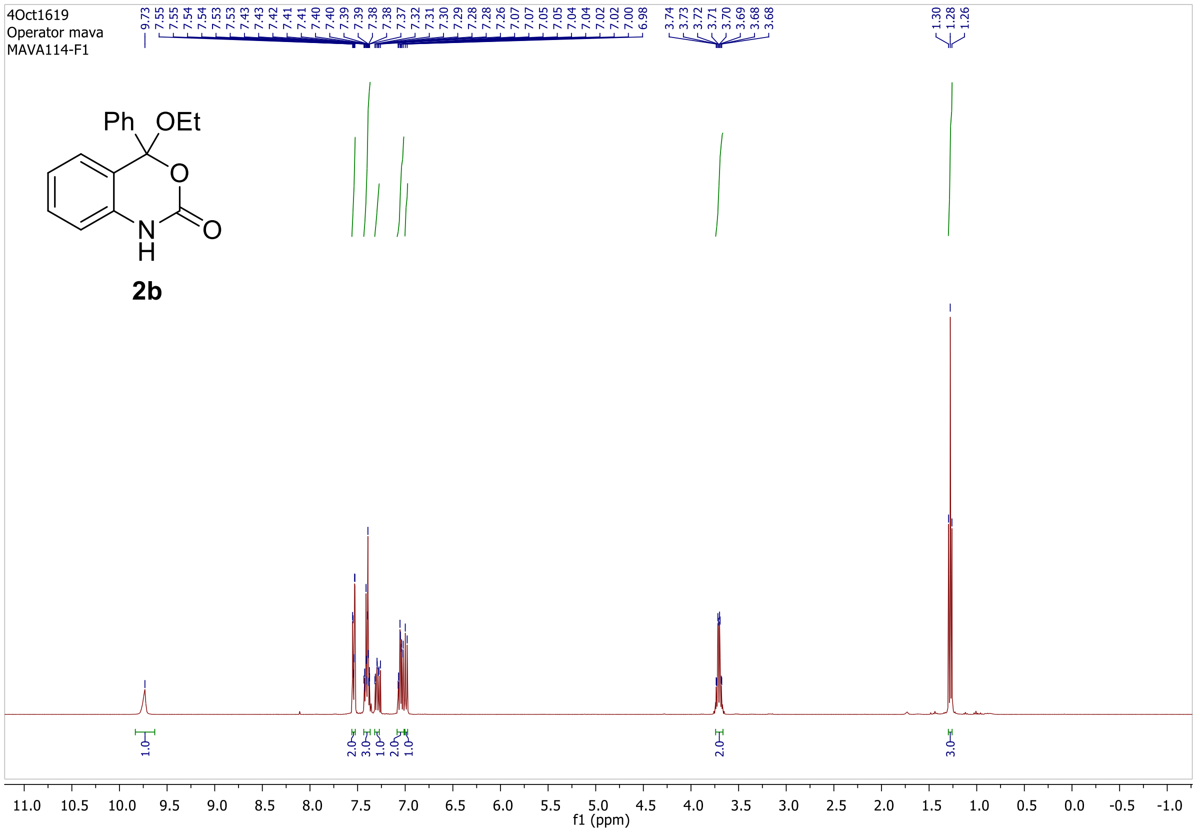


$^{1}$H NMR in $CDCl_3$ at 400 MHz

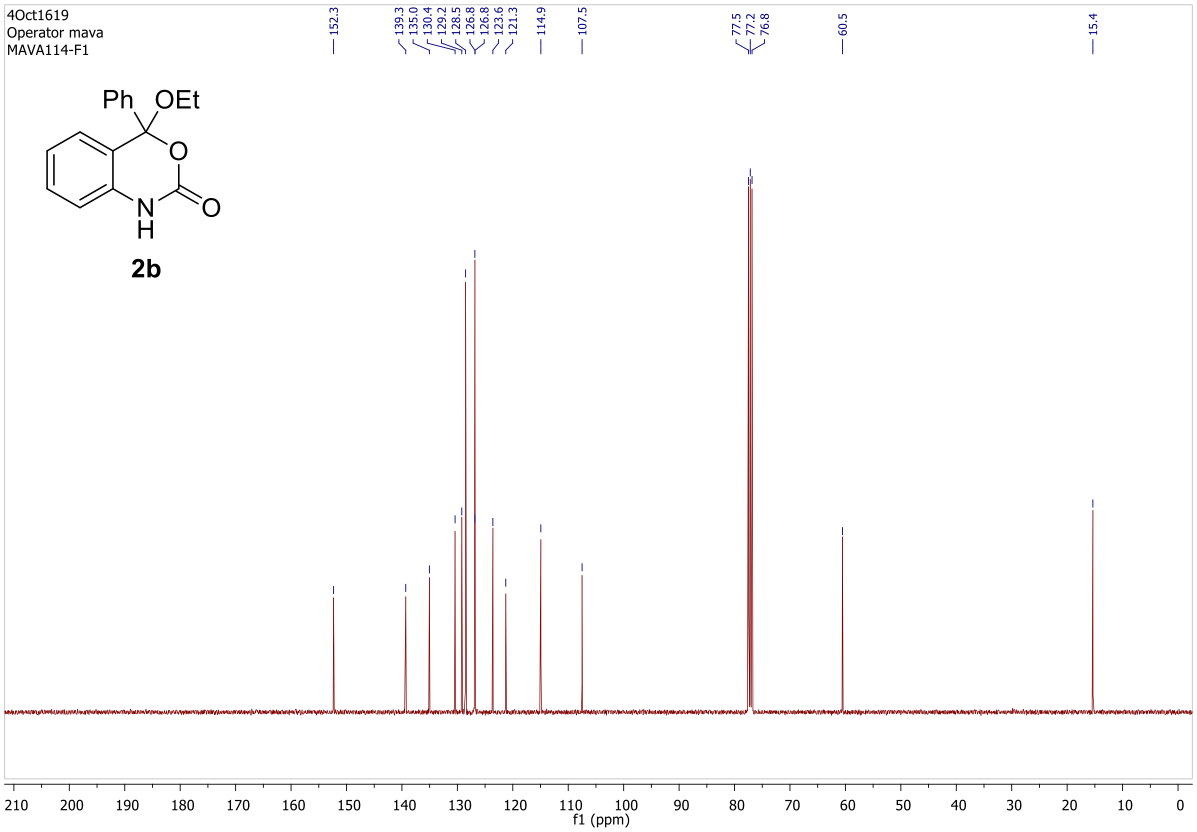


$^{13}C\{^{1}H\}$ NMR in $CDCl_3$ at 100 MHz

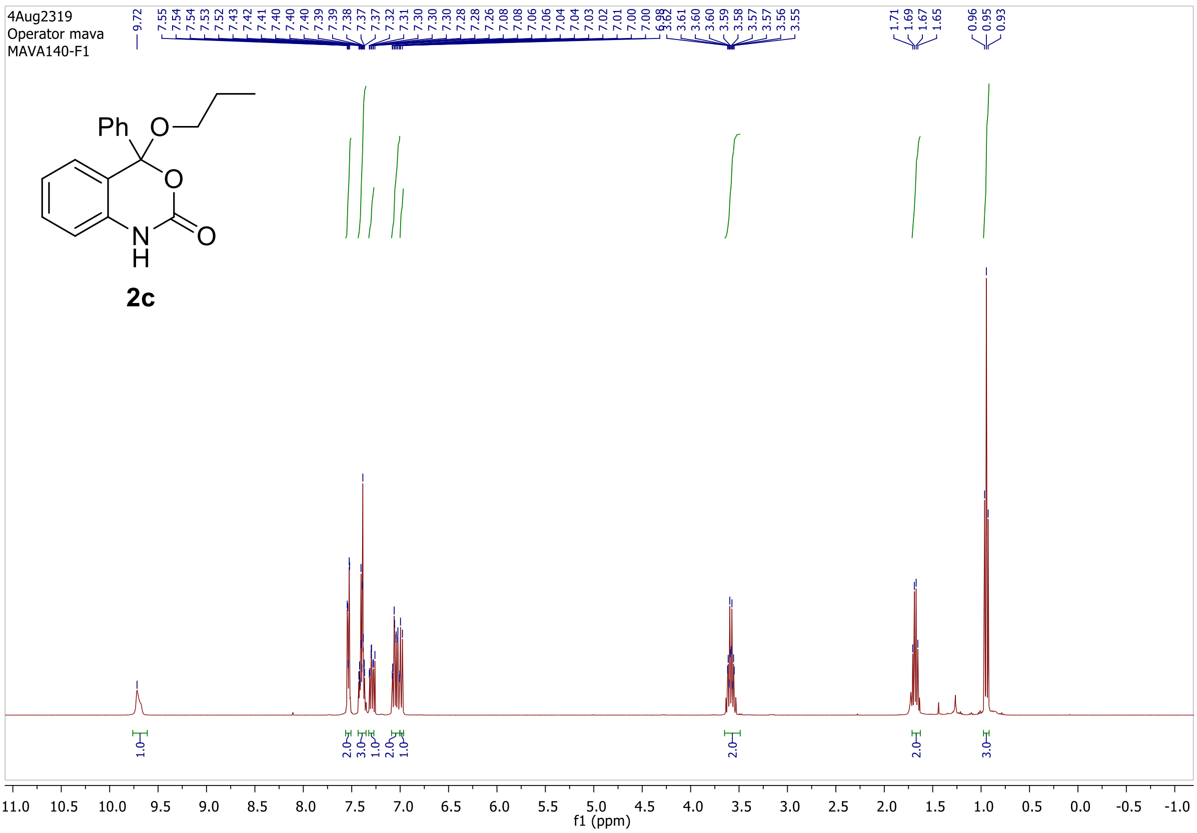

$^{1}H$ NMR in $CDCl_3$ at 400 MHz

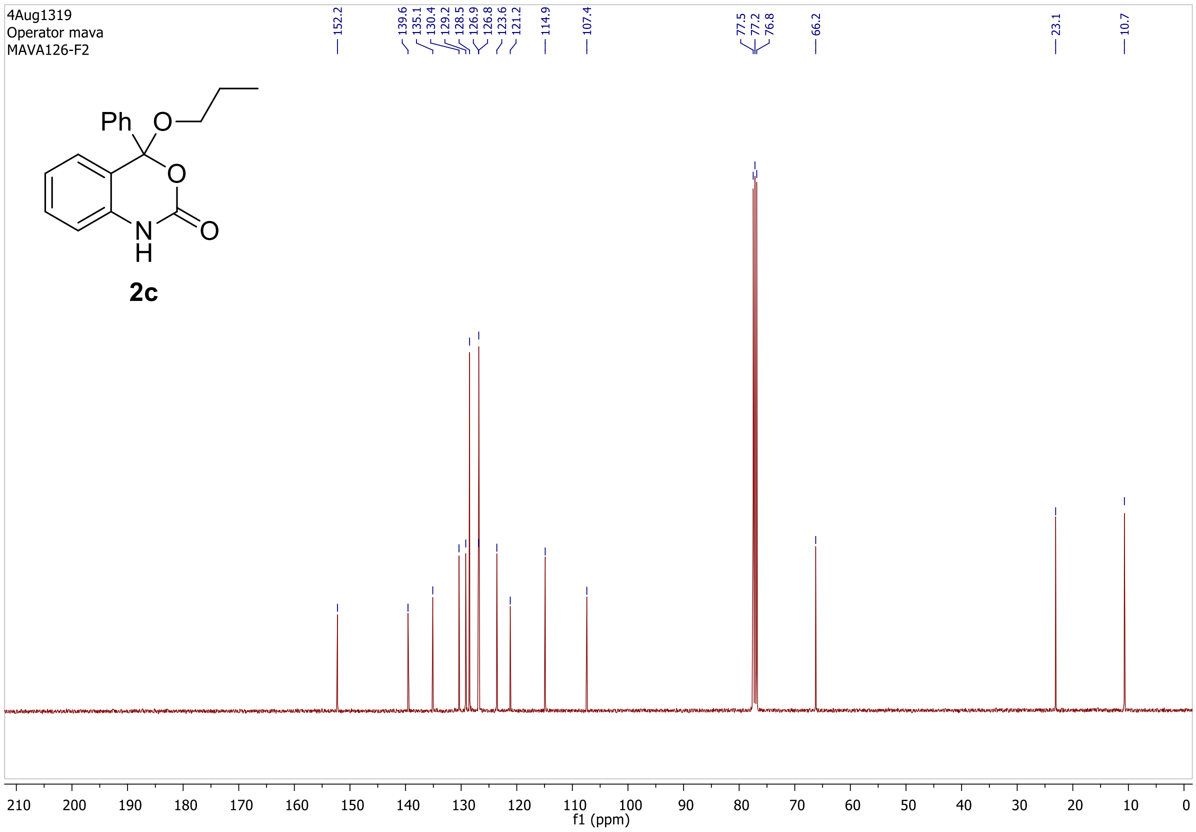

$^{13}C\{^{1}H\}$ NMR in $CDCl_3$ at 100 MHz

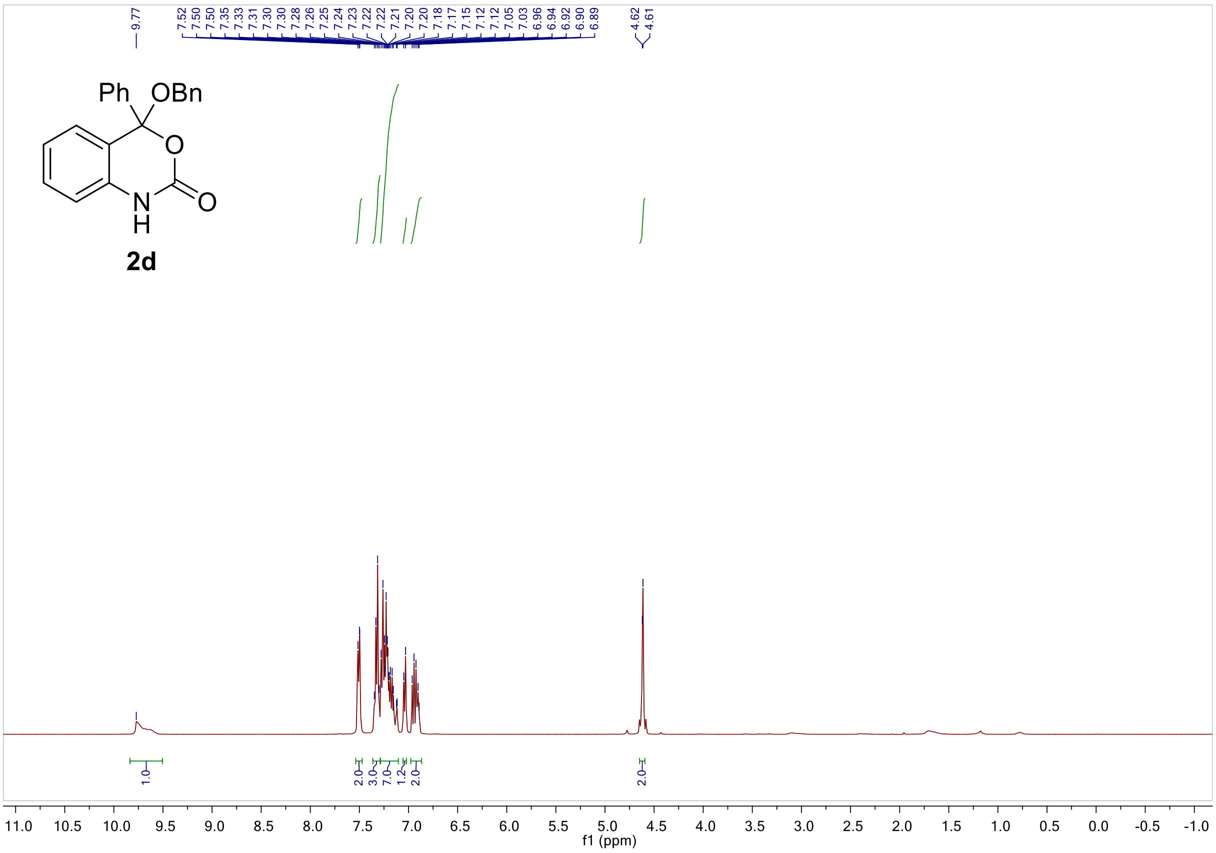

$^{1}H$ NMR in $CDCl_3$ at 400 MHz

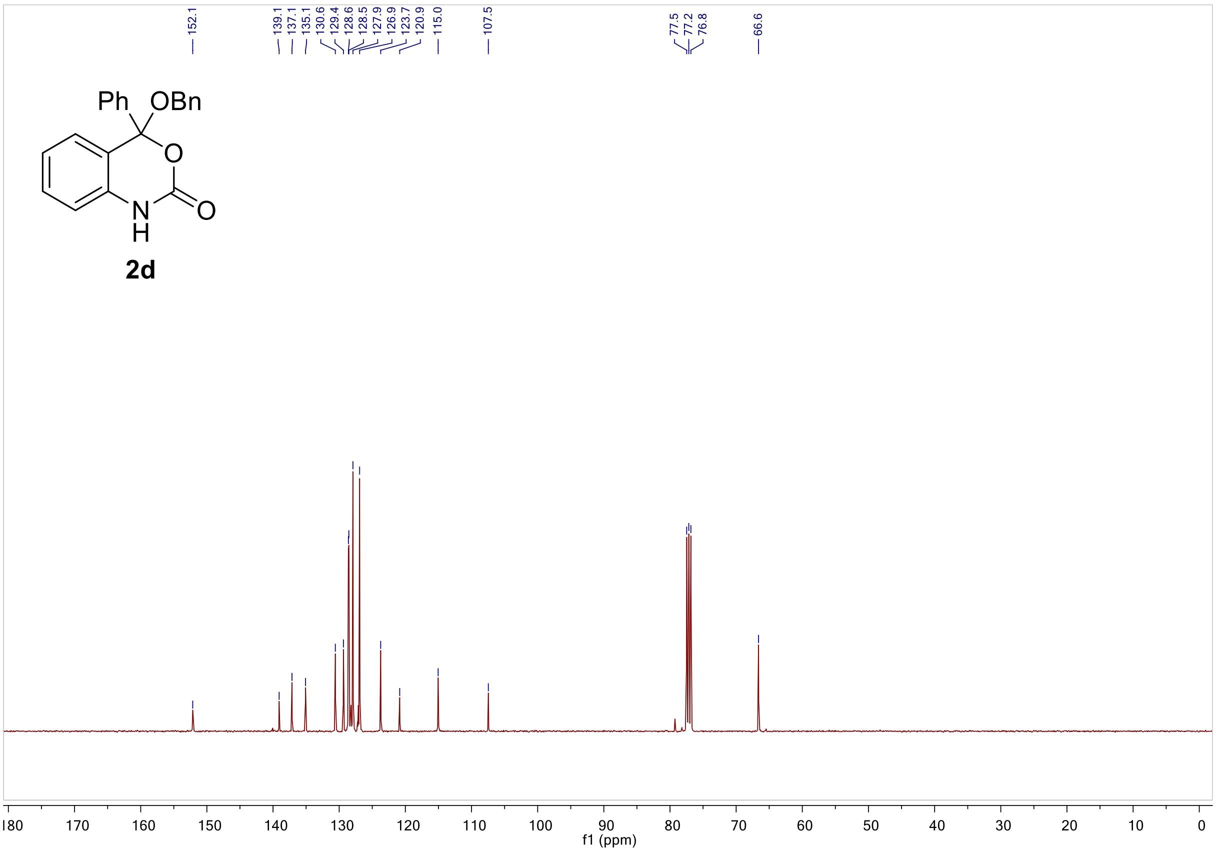

$^{13}C\{^{1}H\}$ NMR in $CDCl_3$ at 100 MHz

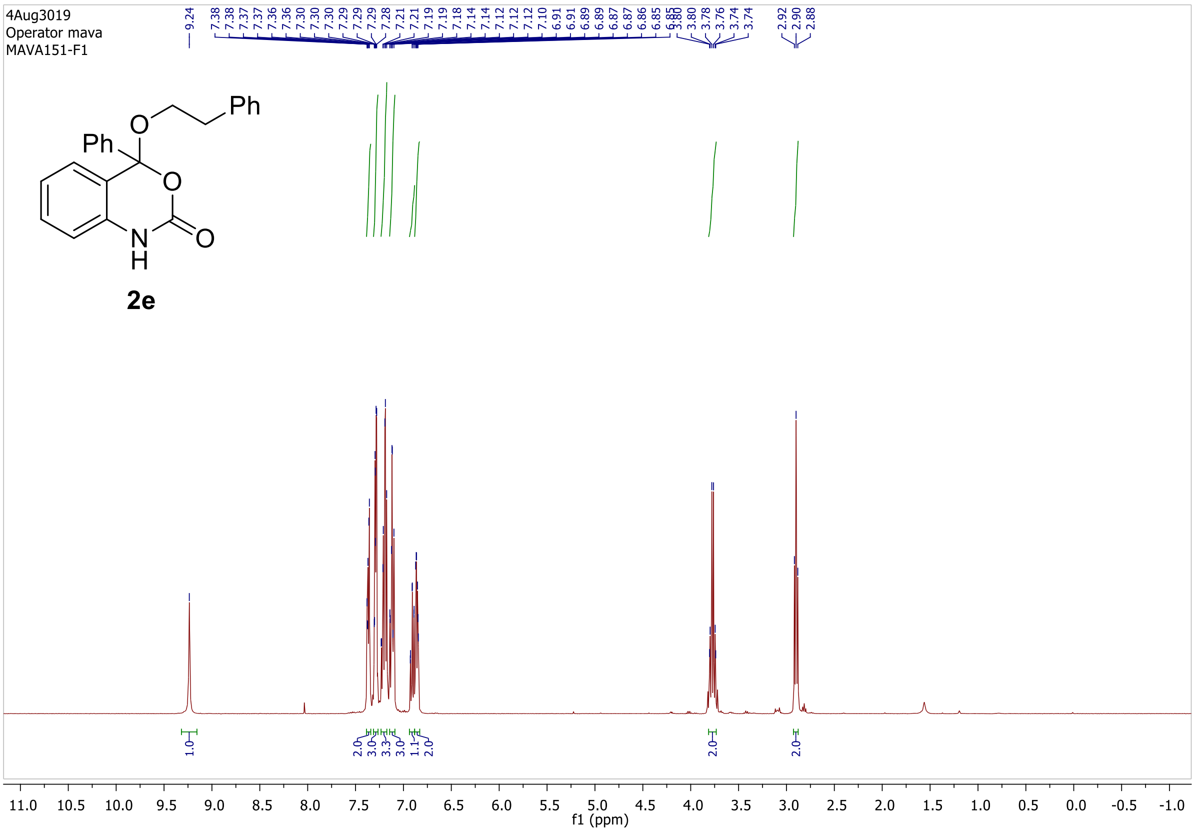


$^{1}H$ NMR in $CDCl_3$ at 400 MHz

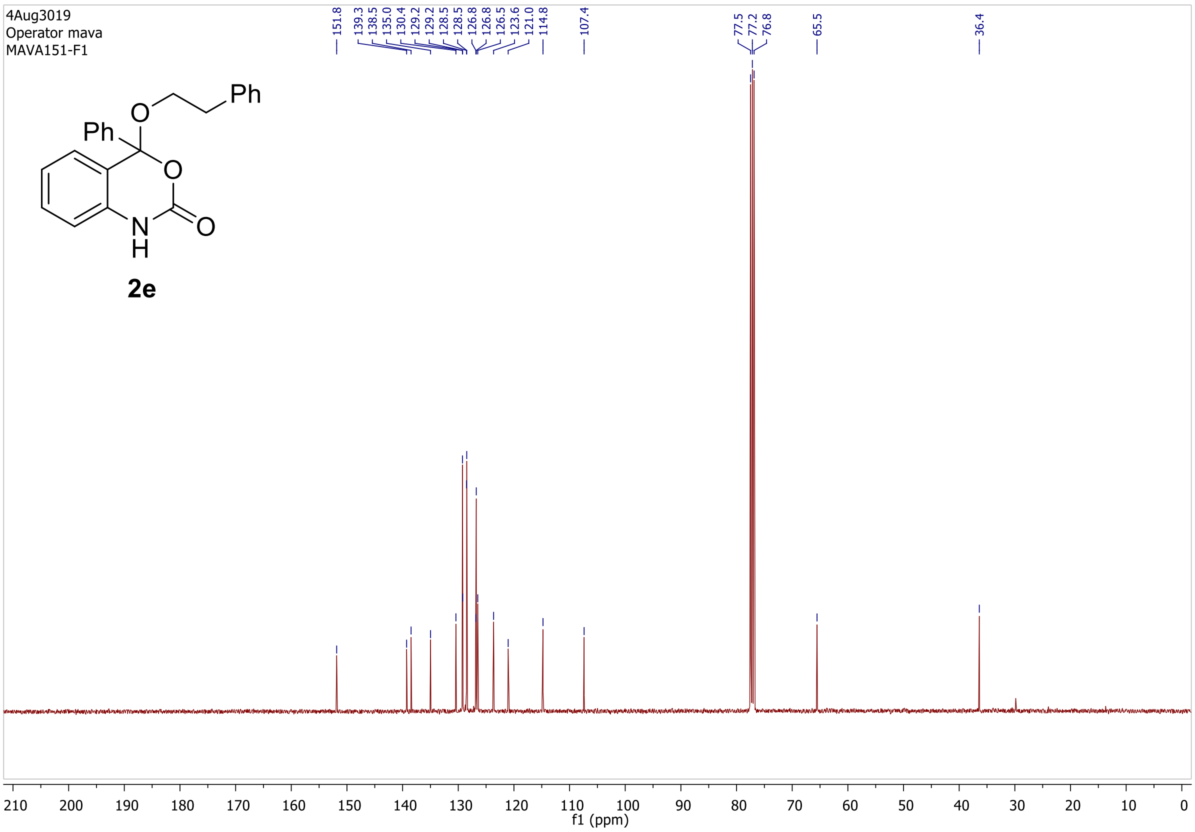


$^{13}C\{^{1}H\}$ NMR in $CDCl_3$ at 100 MHz

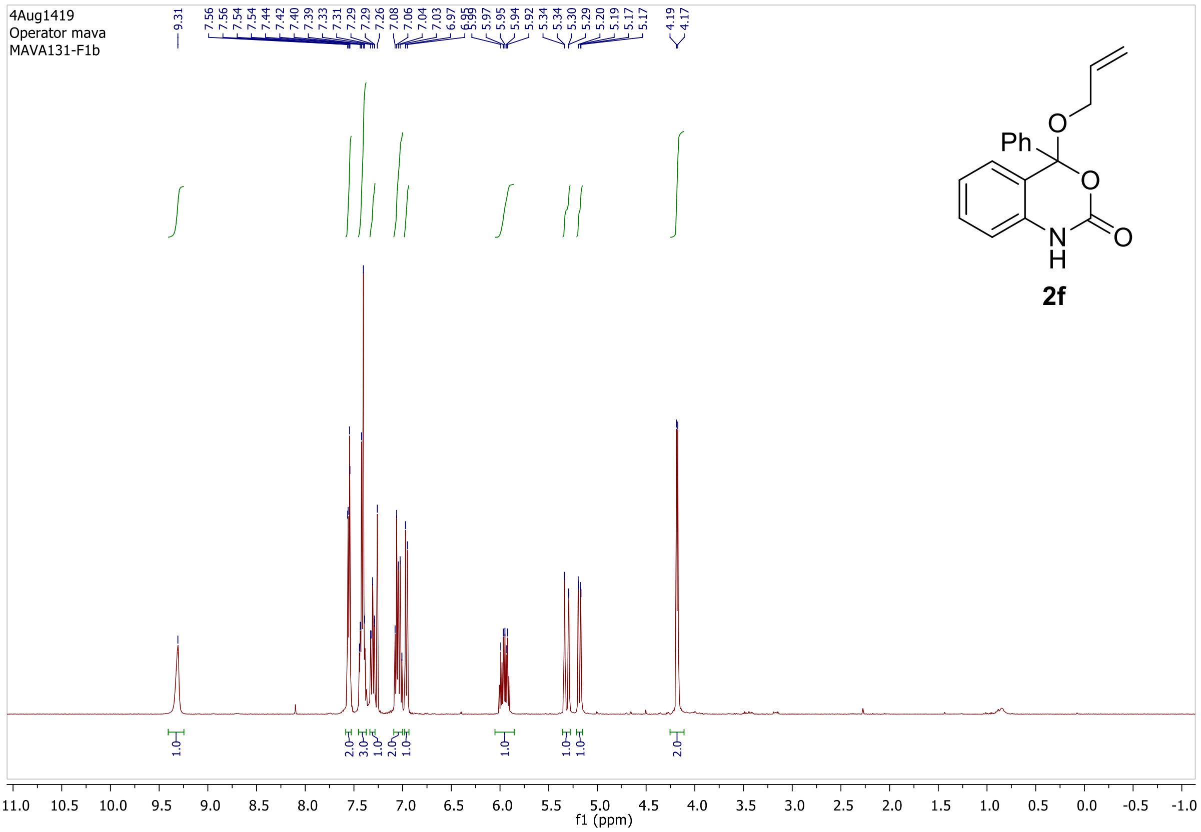


$^{1}H$ NMR in $CDCl_3$ at 400 MHz

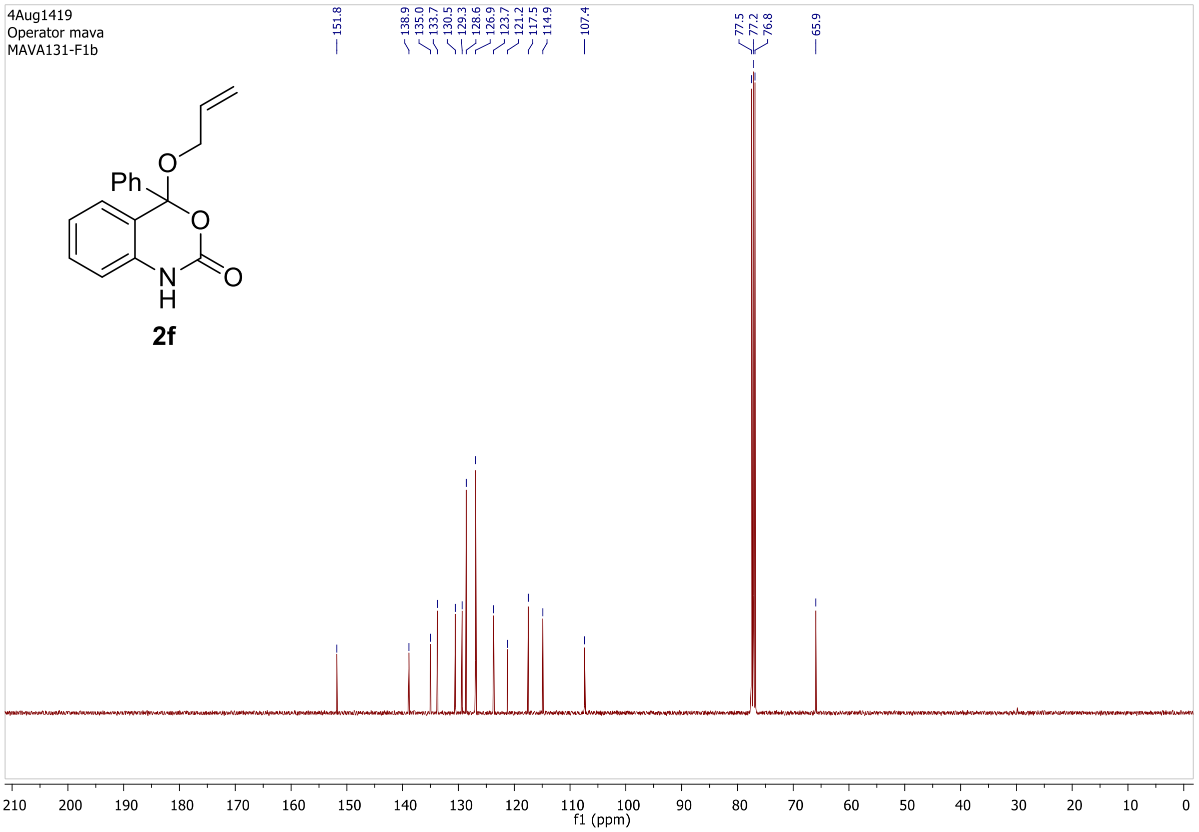


$^{13}C\{^{1}H\}$ NMR in $CDCl_3$ at 100 MHz

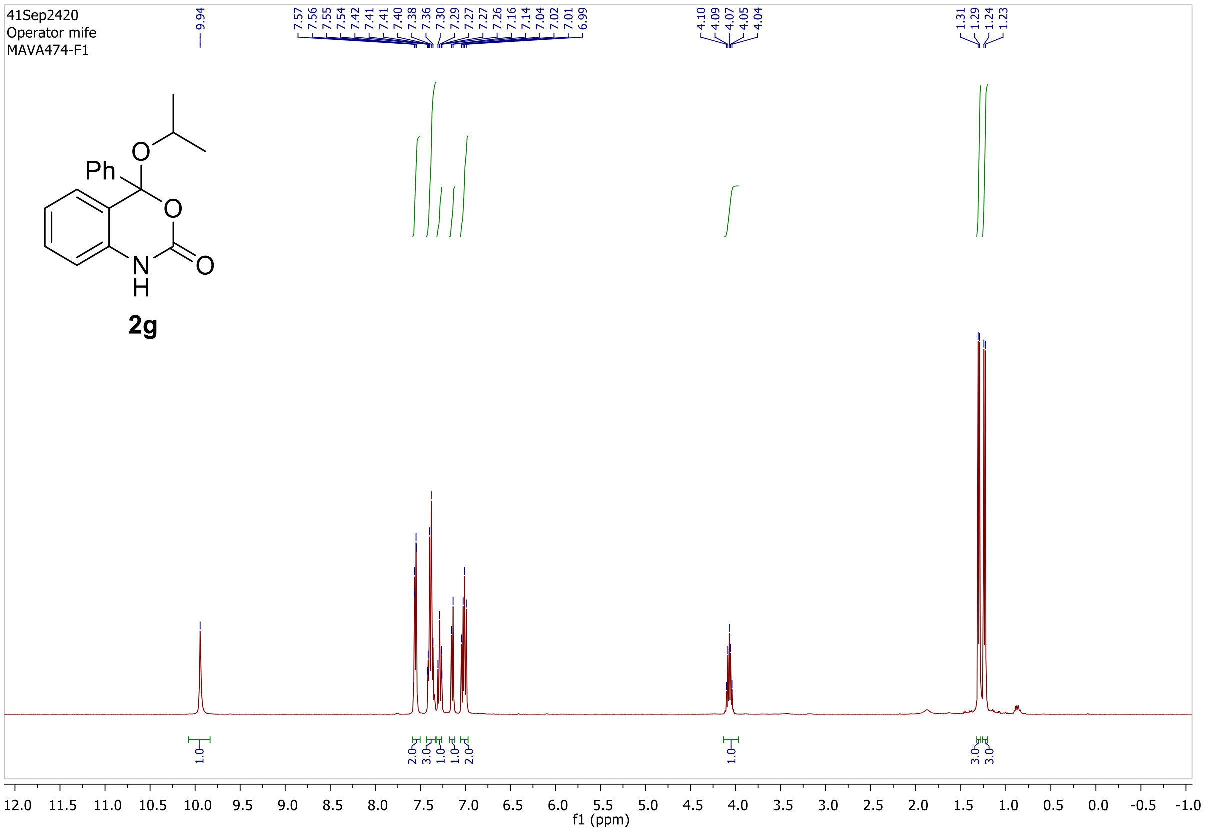


$^{1}$H NMR in $CDCl_3$ at 400 MHz

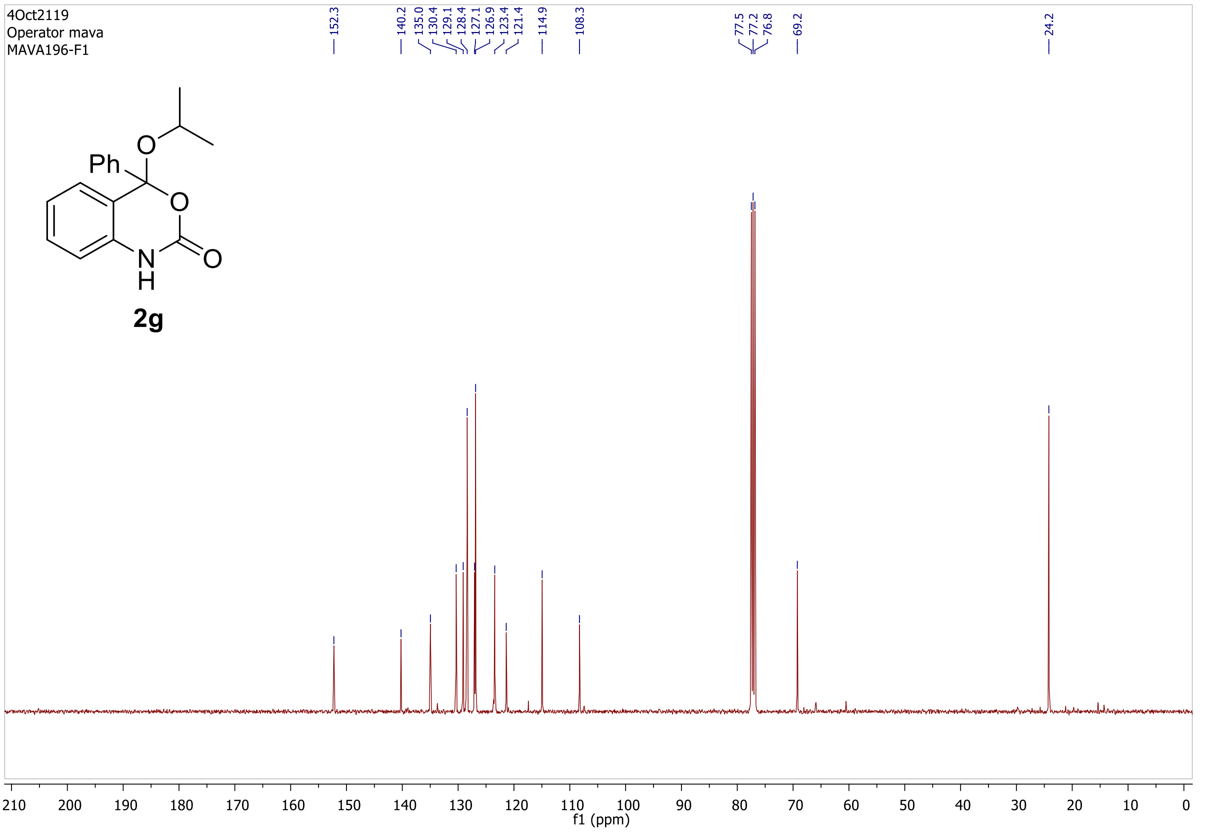


$^{13}C\{^{1}H\}$ NMR in $CDCl_3$ at 100 MHz

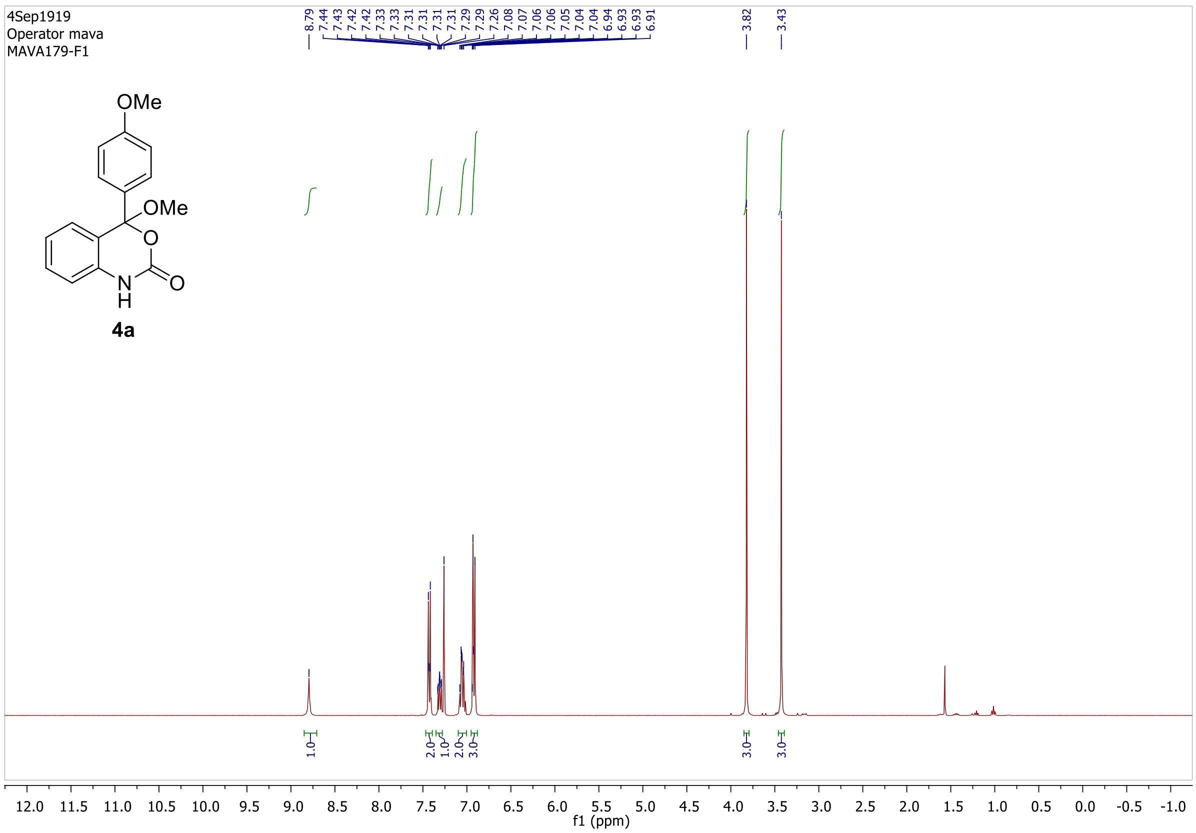


$^{1}H$ NMR in $CDCl_3$ at 400 MHz

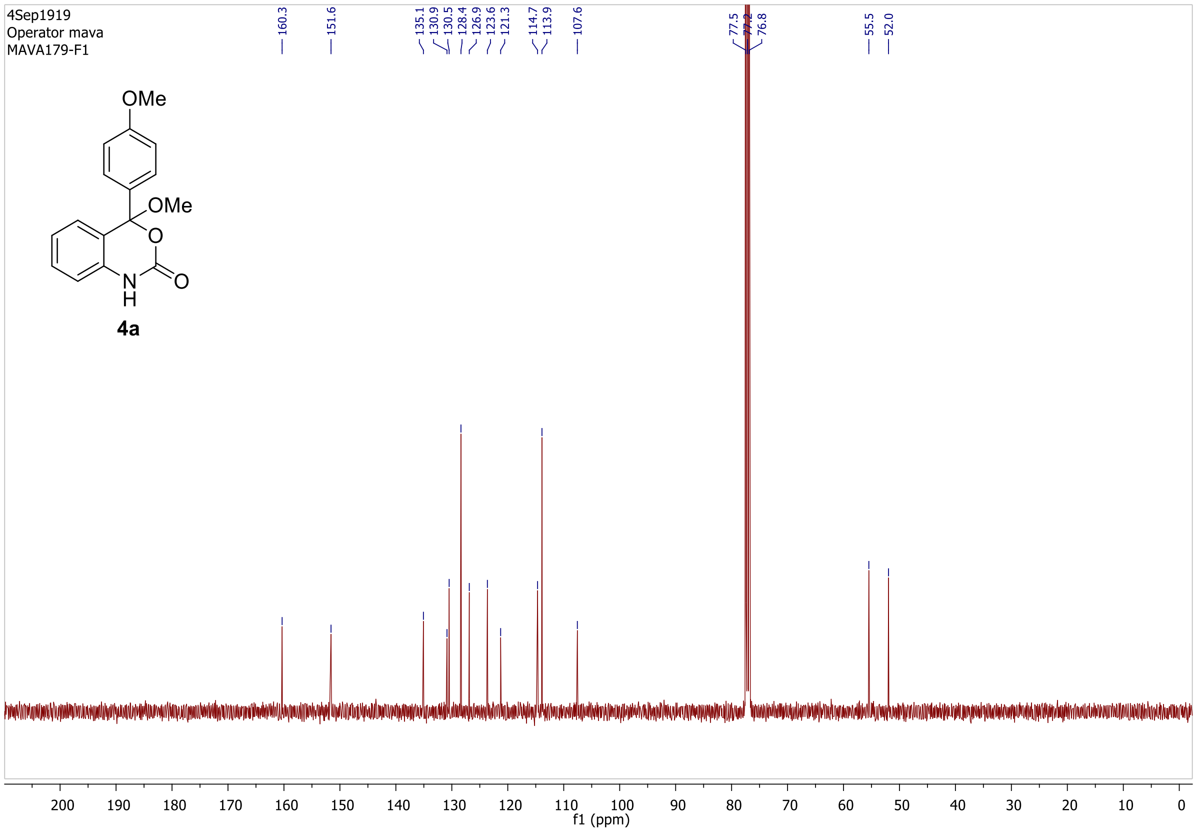


$^{13}C\{^{1}H\}$ NMR in $CDCl_3$ at 100 MHz

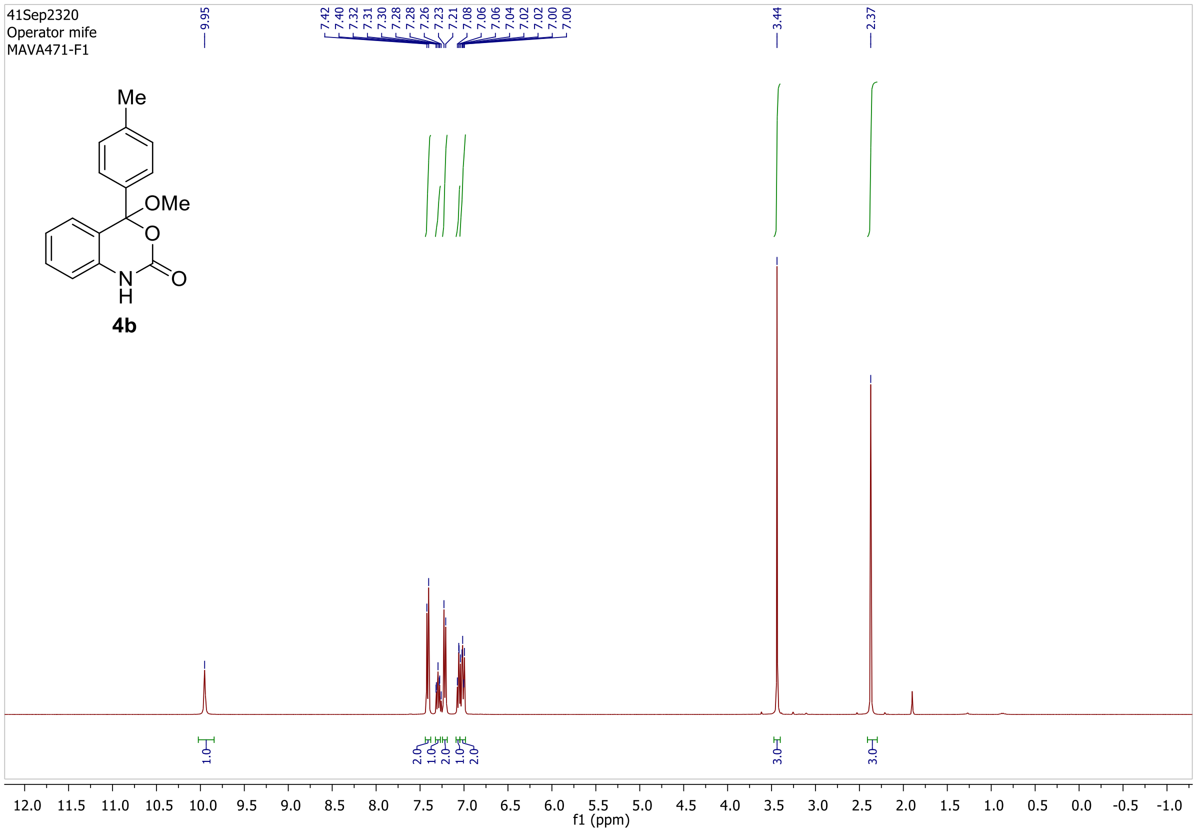


$^1$H NMR in $CDCl_3$ at 400 MHz

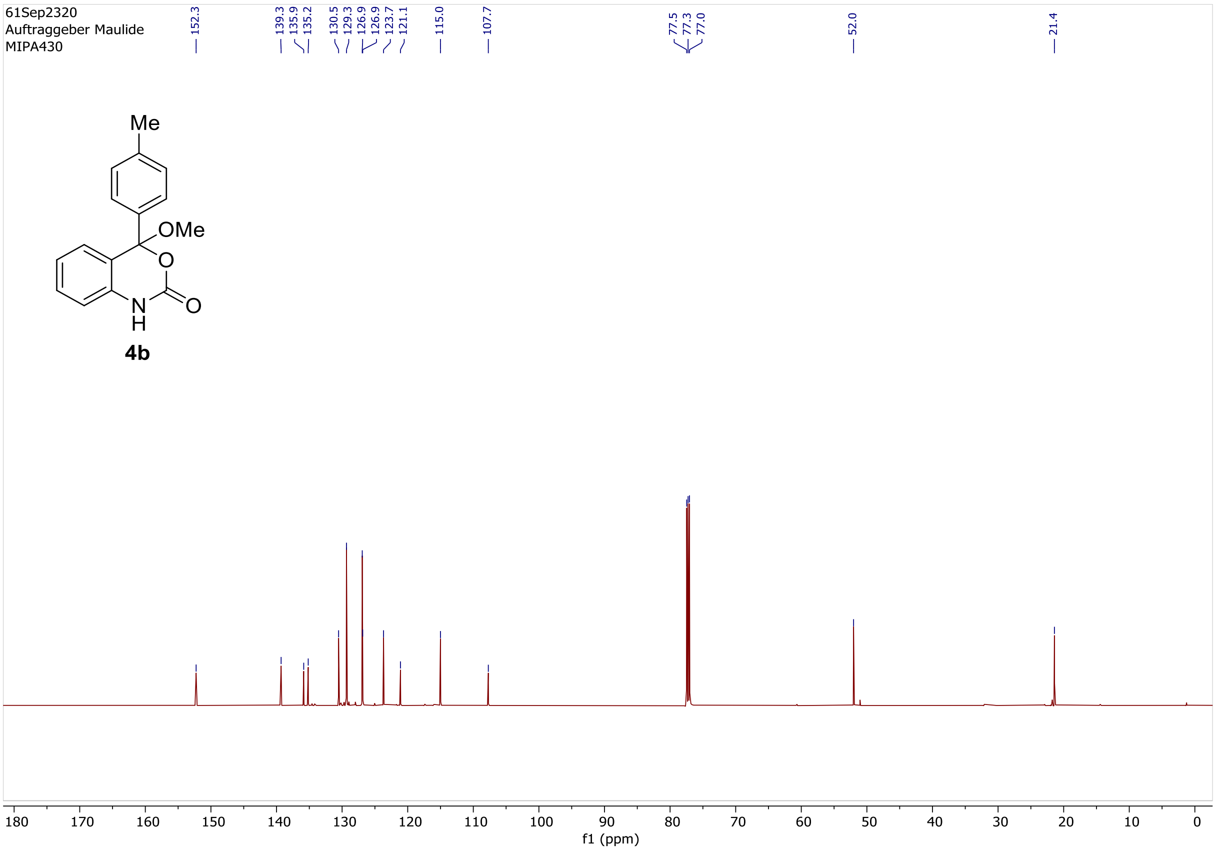


$^{13}C\{^1H\}$ NMR in $CDCl_3$ at 150 MHz

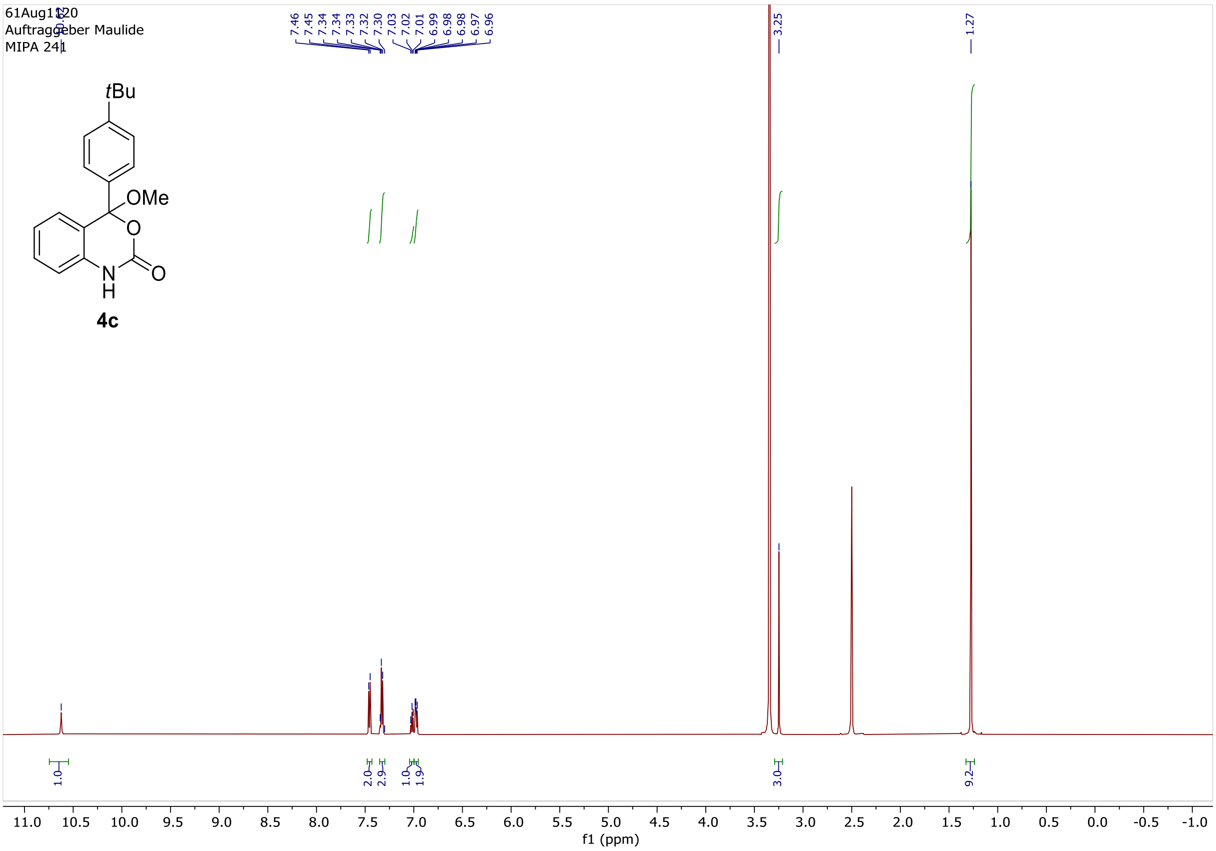

$^{1}H$ NMR in $CDCl_3$ at 600 MHz

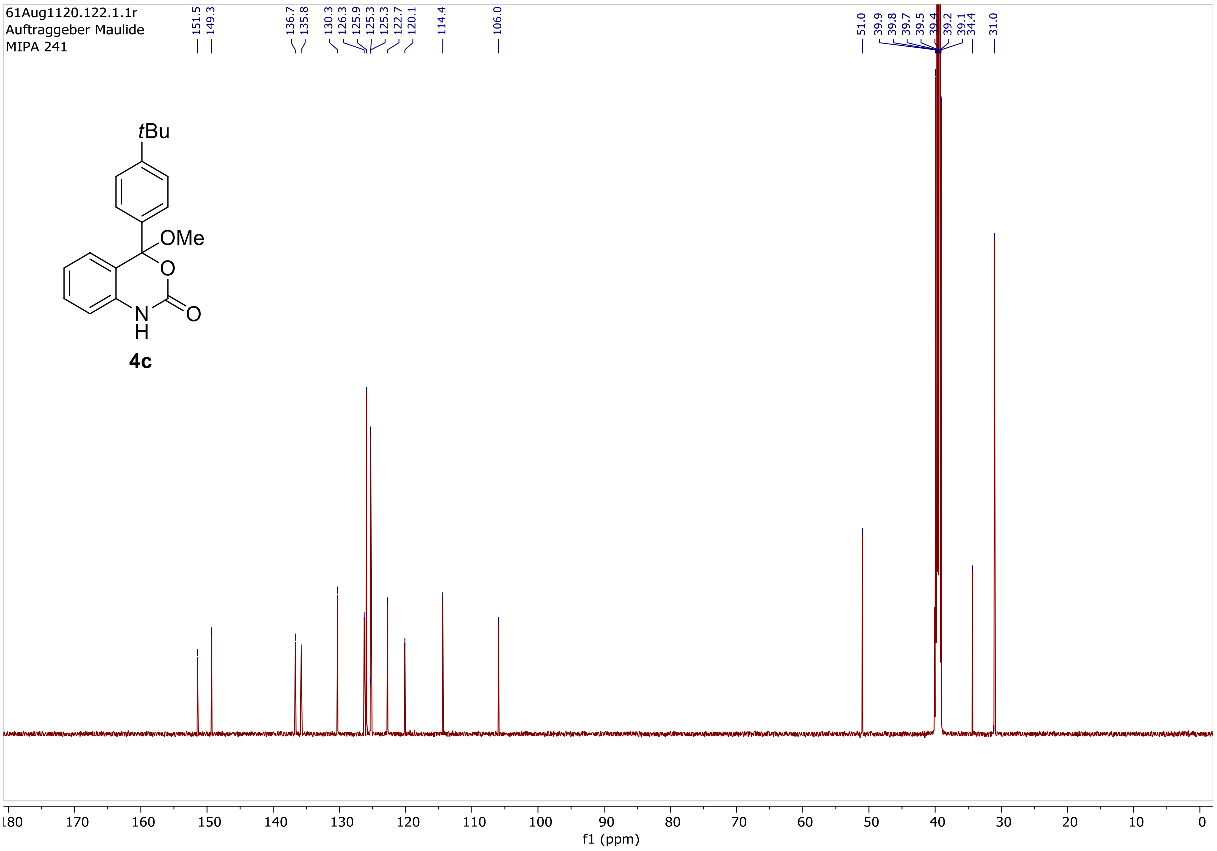

$^{13}C\{^{1}H\}$ NMR in $CDCl_3$ at 150 MHz

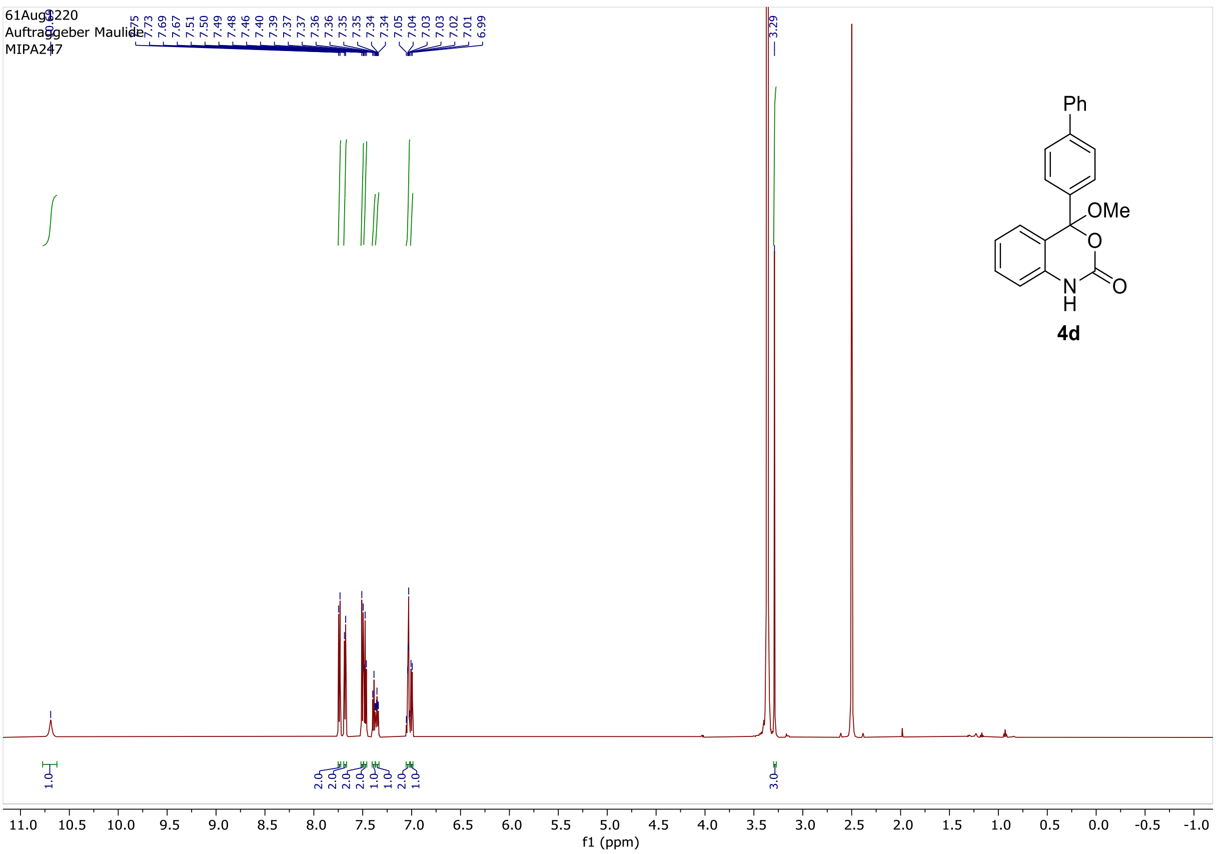

$^{1}H$ NMR in $CDCl_3$ at 600 MHz

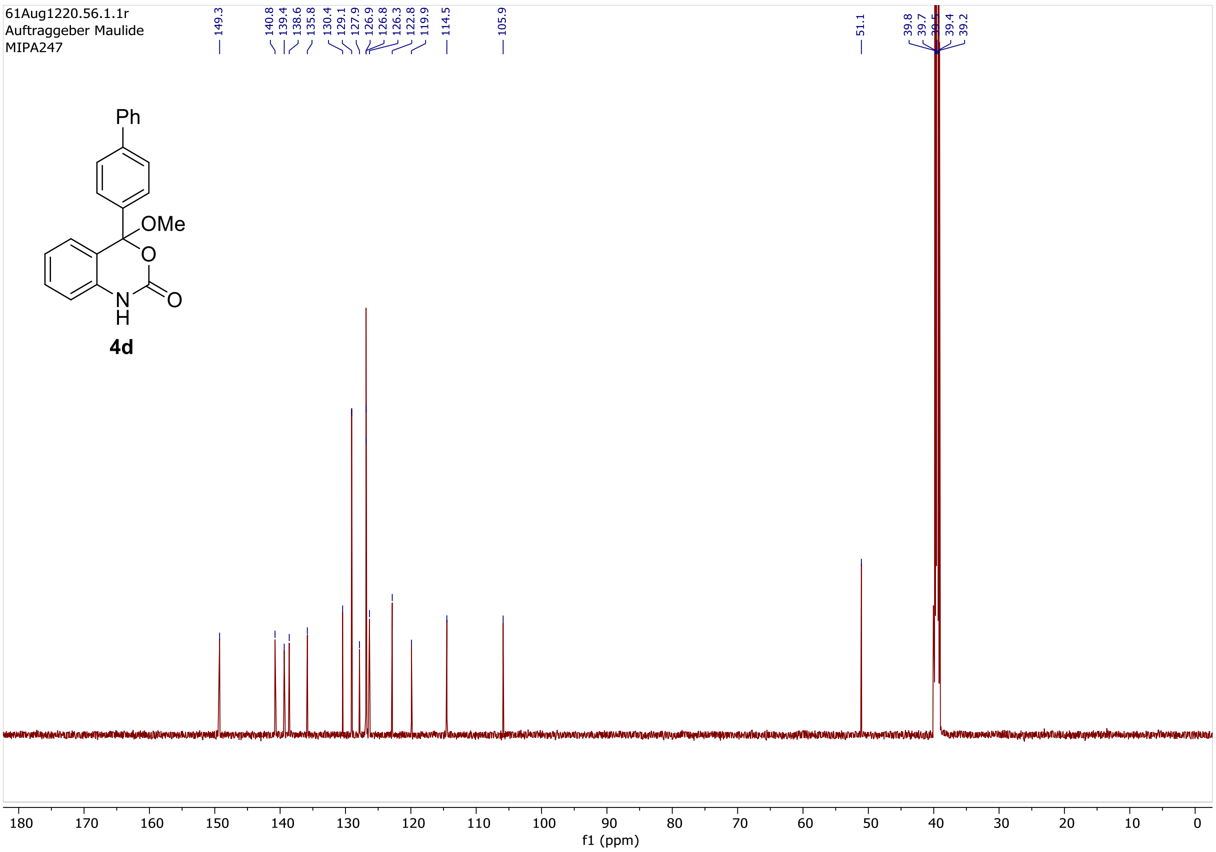

$^{13}C\{^{1}H\}$ NMR in $CDCl_3$ at 150 MHz

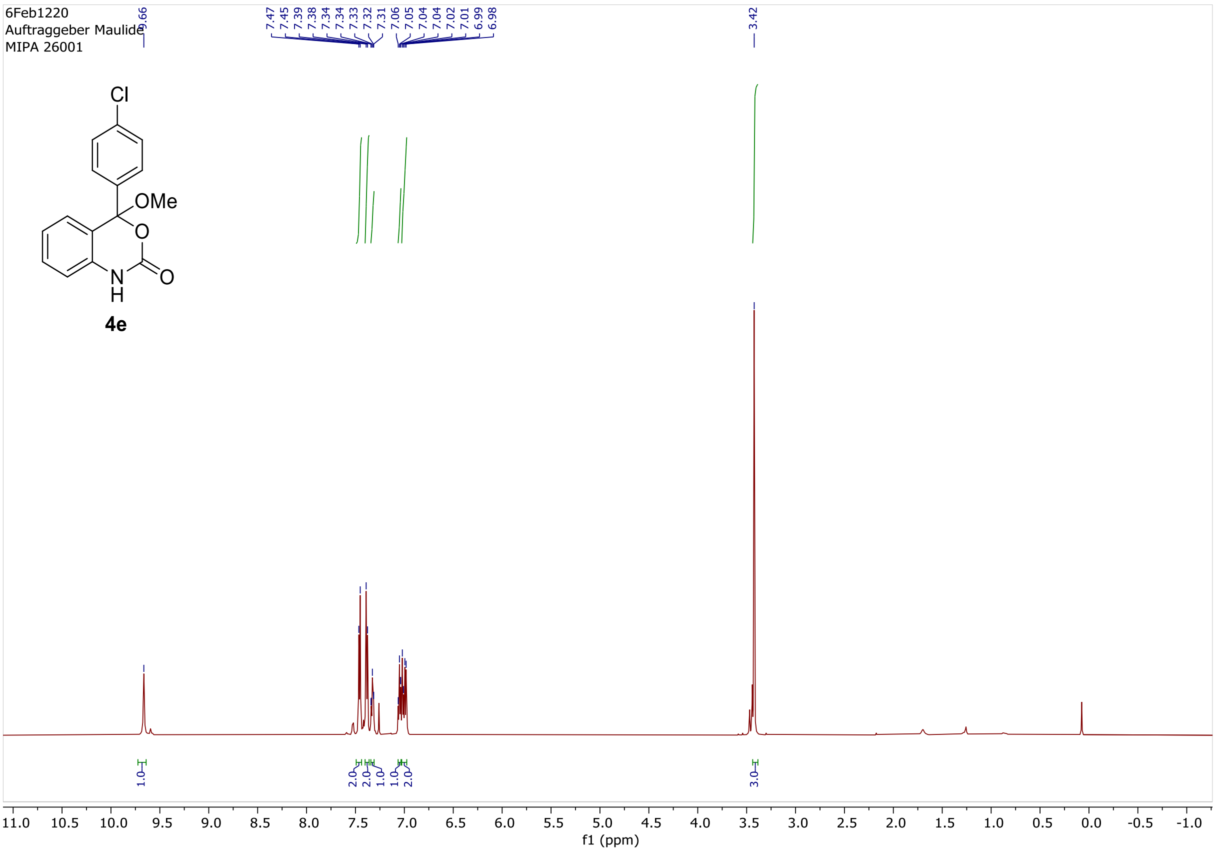


$^{1}H$ NMR in $CDCl_3$ at 600 MHz

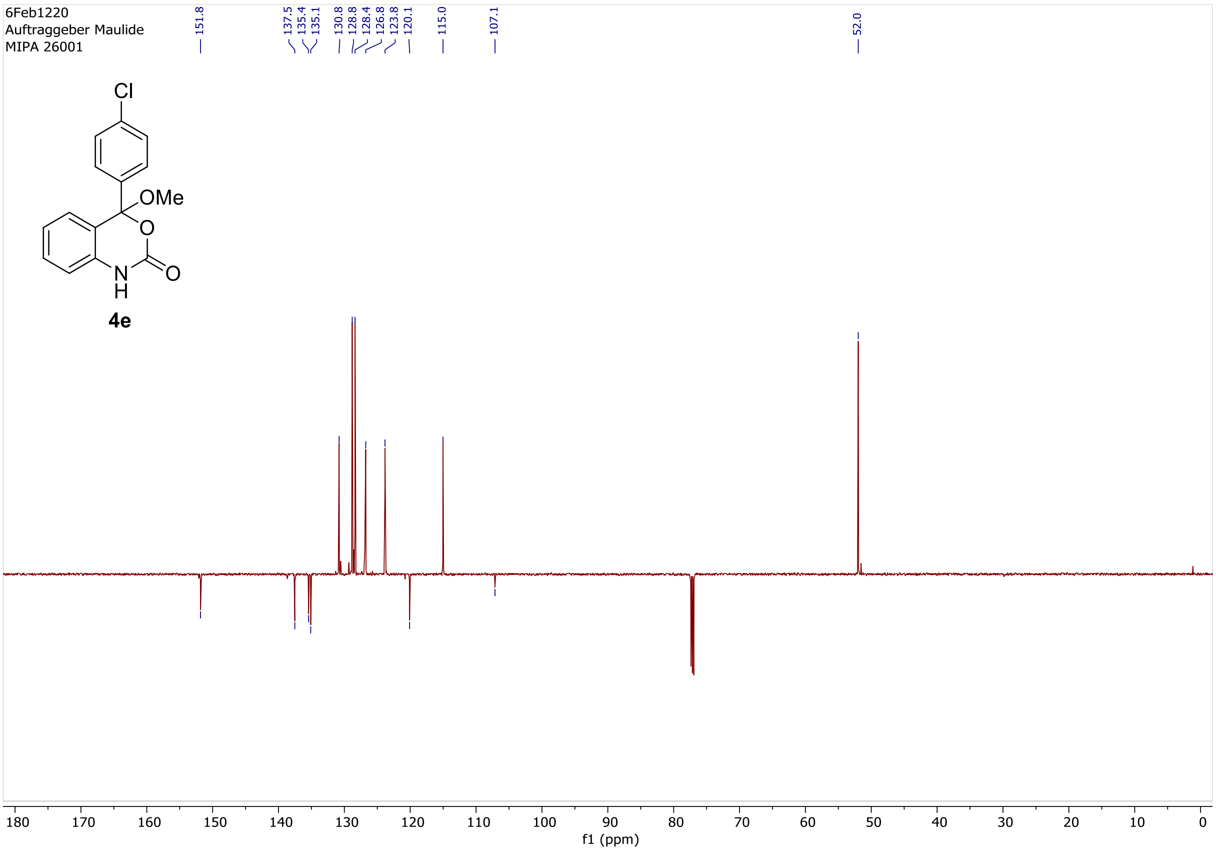


$^{13}C\{^{1}H\}$ NMR in $CDCl_3$ at 150 MHz

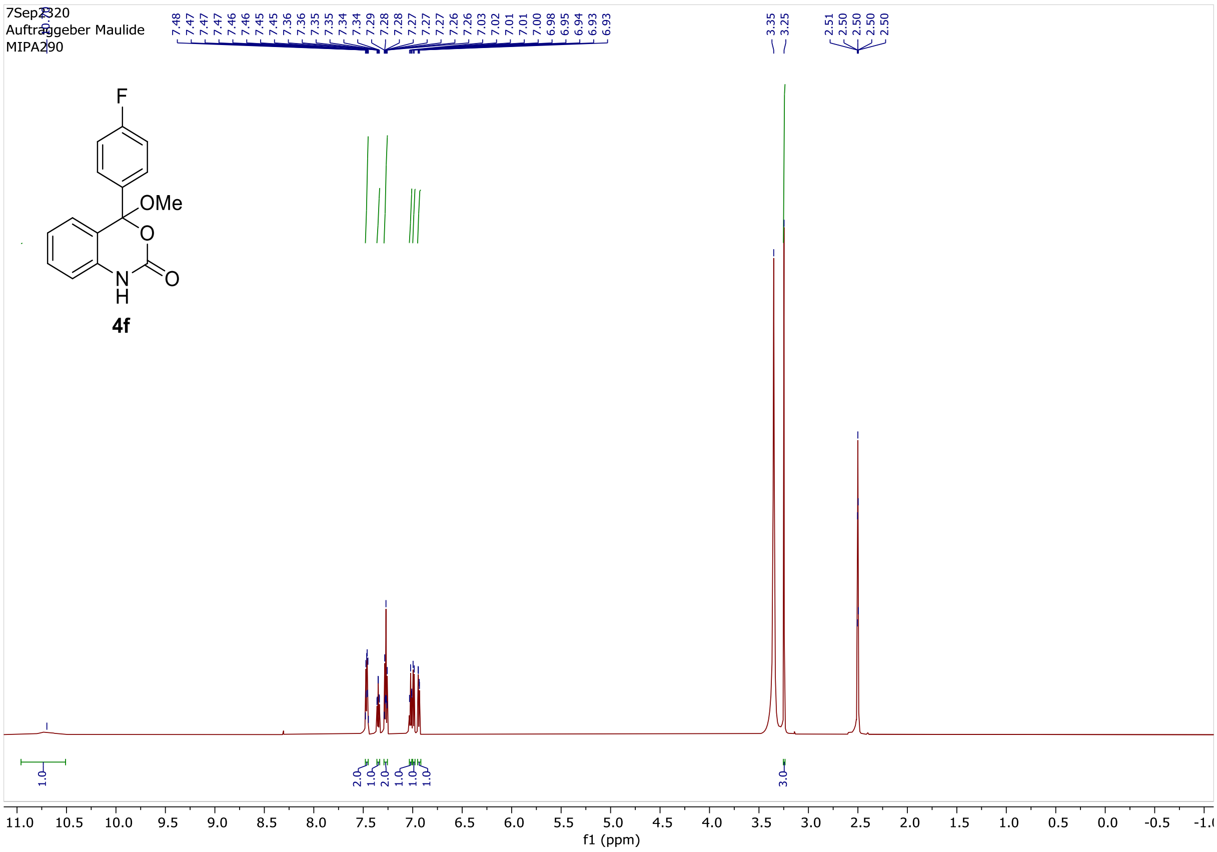

$^{1}H$ NMR in $CDCl_3$ at 700 MHz

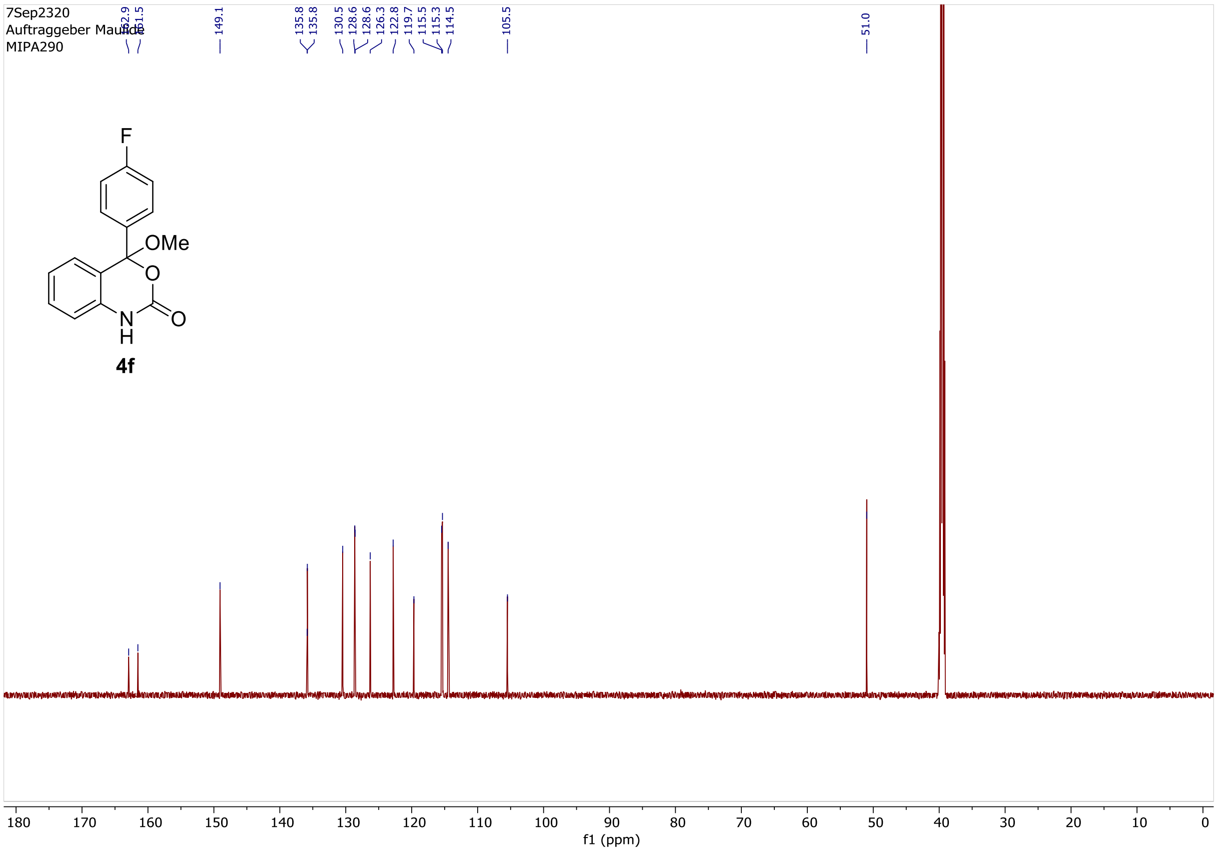

$^{13}C\{^{1}H\}$ NMR in $CDCl_3$ at 176 MHz

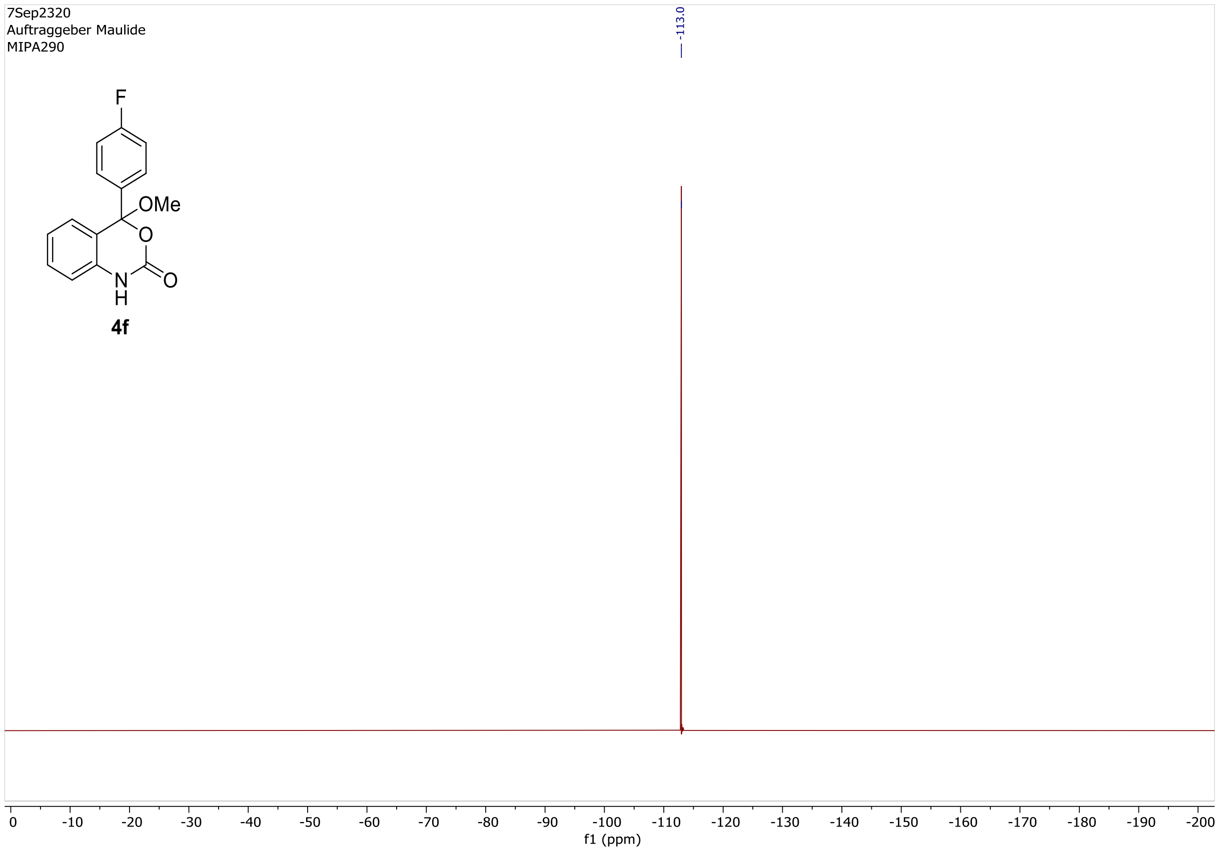


$^{19}$F NMR in DMSO-$d_6$ at 659 MHz

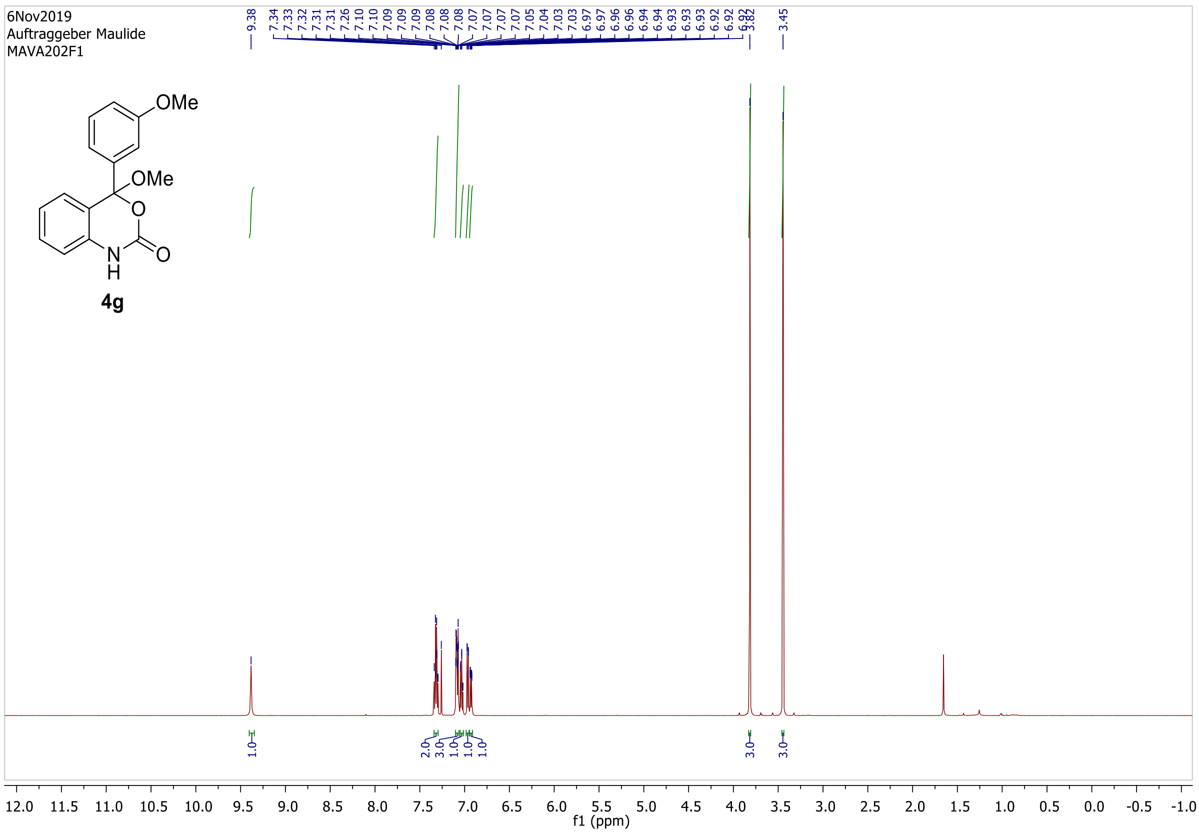


$^{1}H$ NMR in $CDCl_3$ at 600 MHz

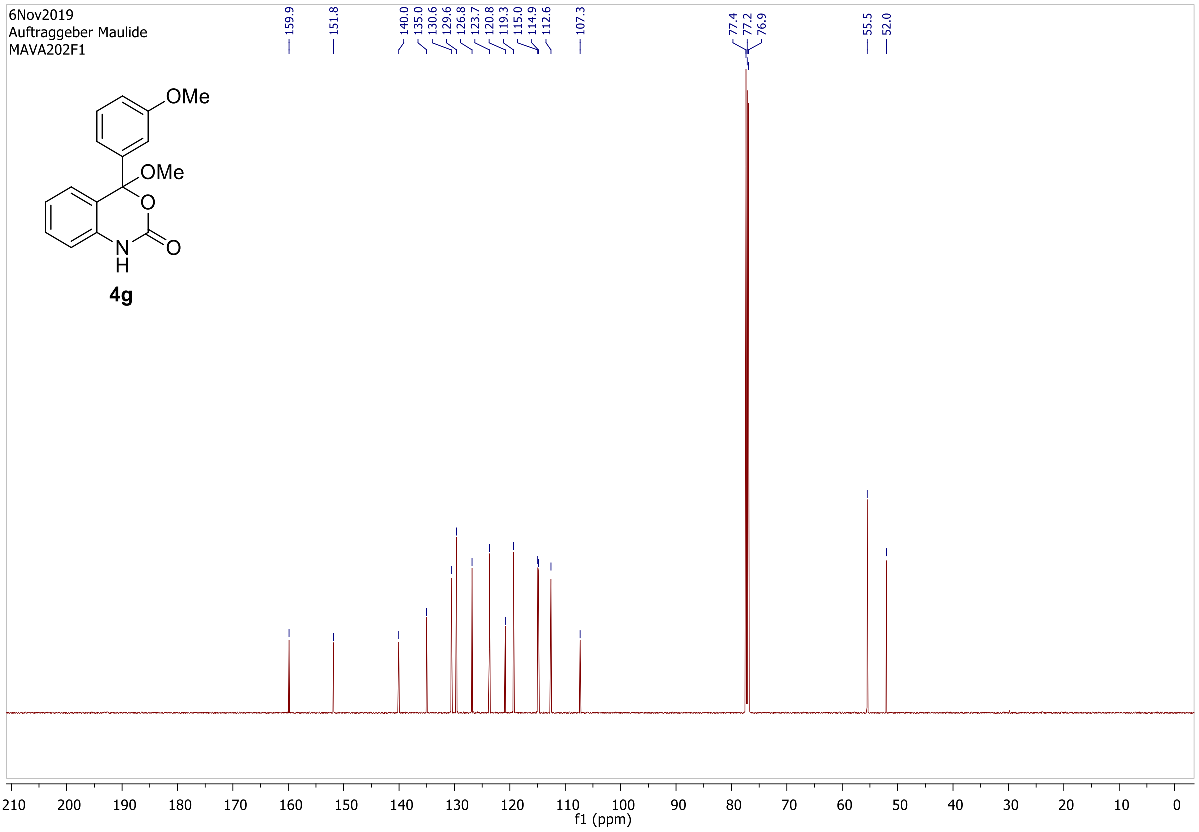


$^{13}C\{^{1}H\}$ NMR in $CDCl_3$ at 150 MHz

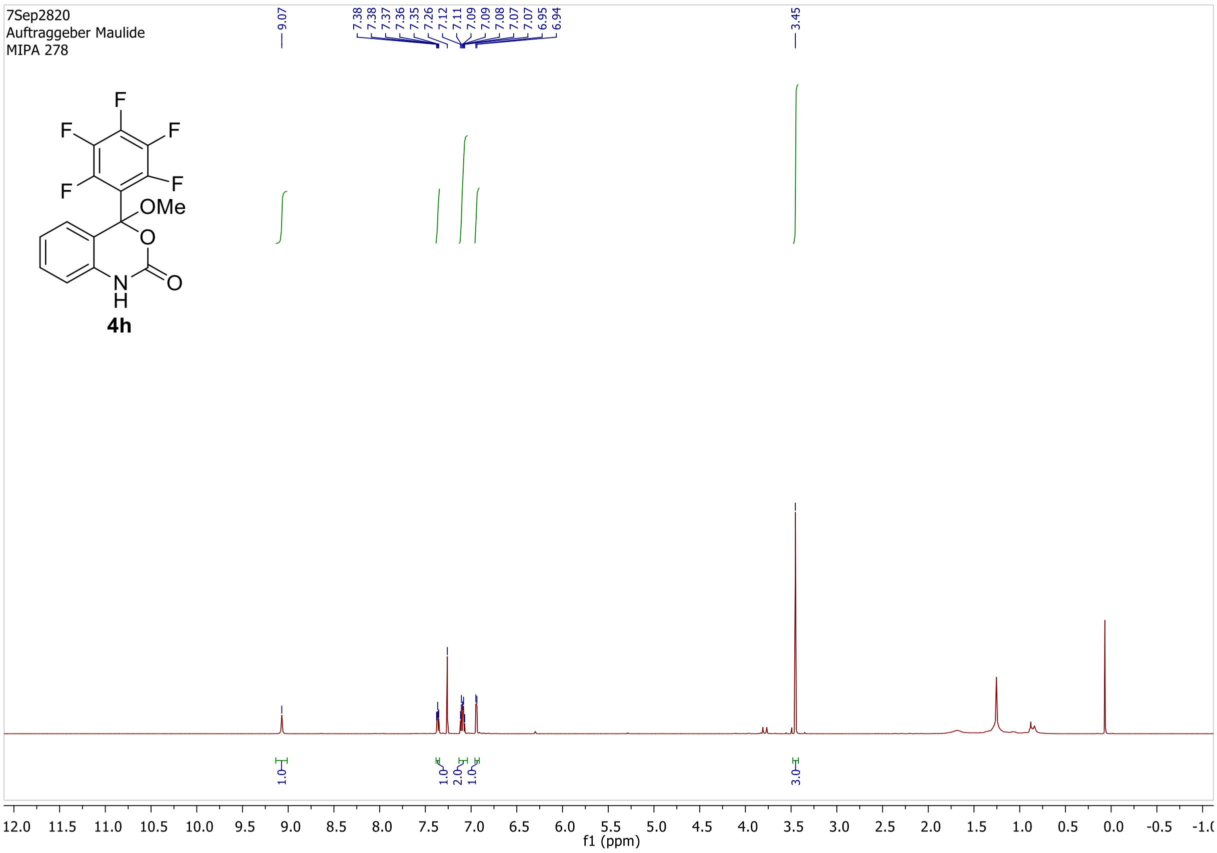


$^{1}$H NMR in $CDCl_3$ at 700 MHz

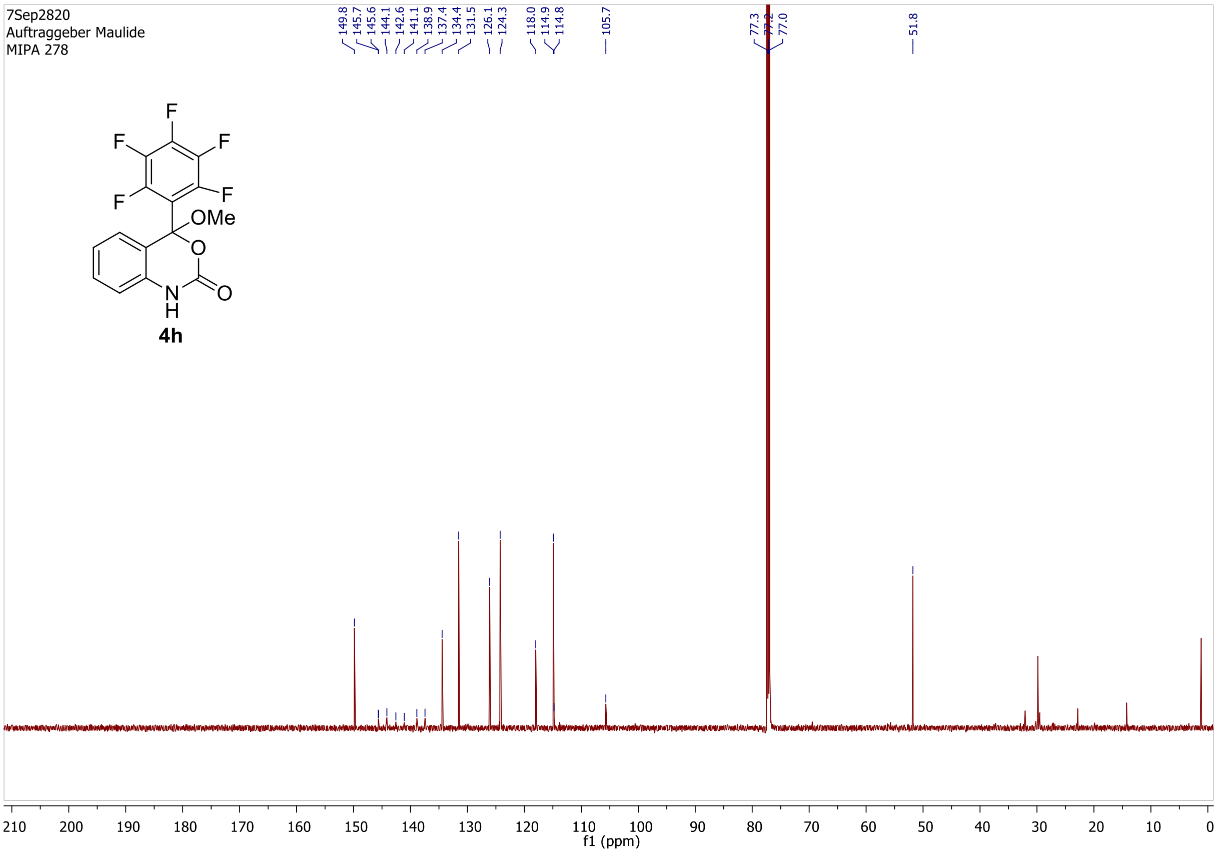


$^{13}$C{$^{1}$H} NMR in $CDCl_3$ at 176 MHz

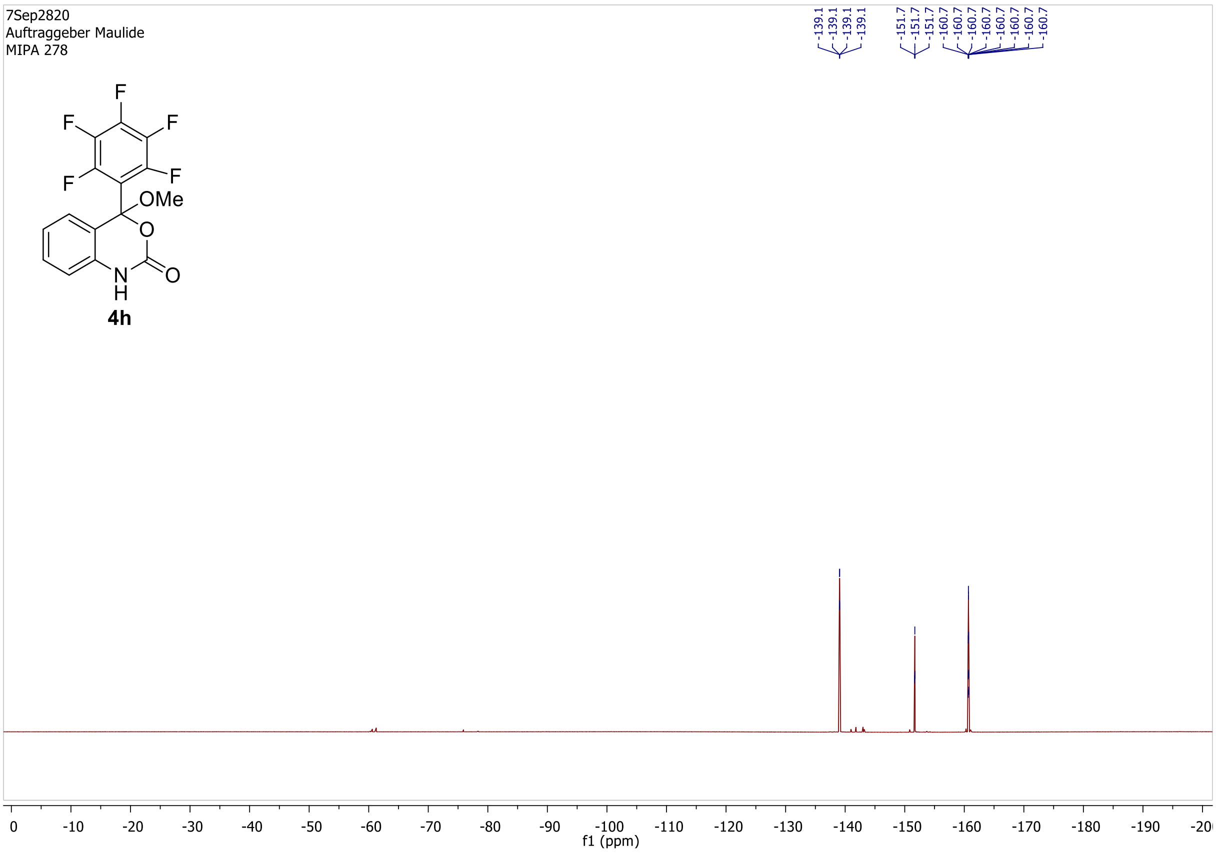


$^{19}$F NMR in DMSO-$d_6$ at 659 MHz

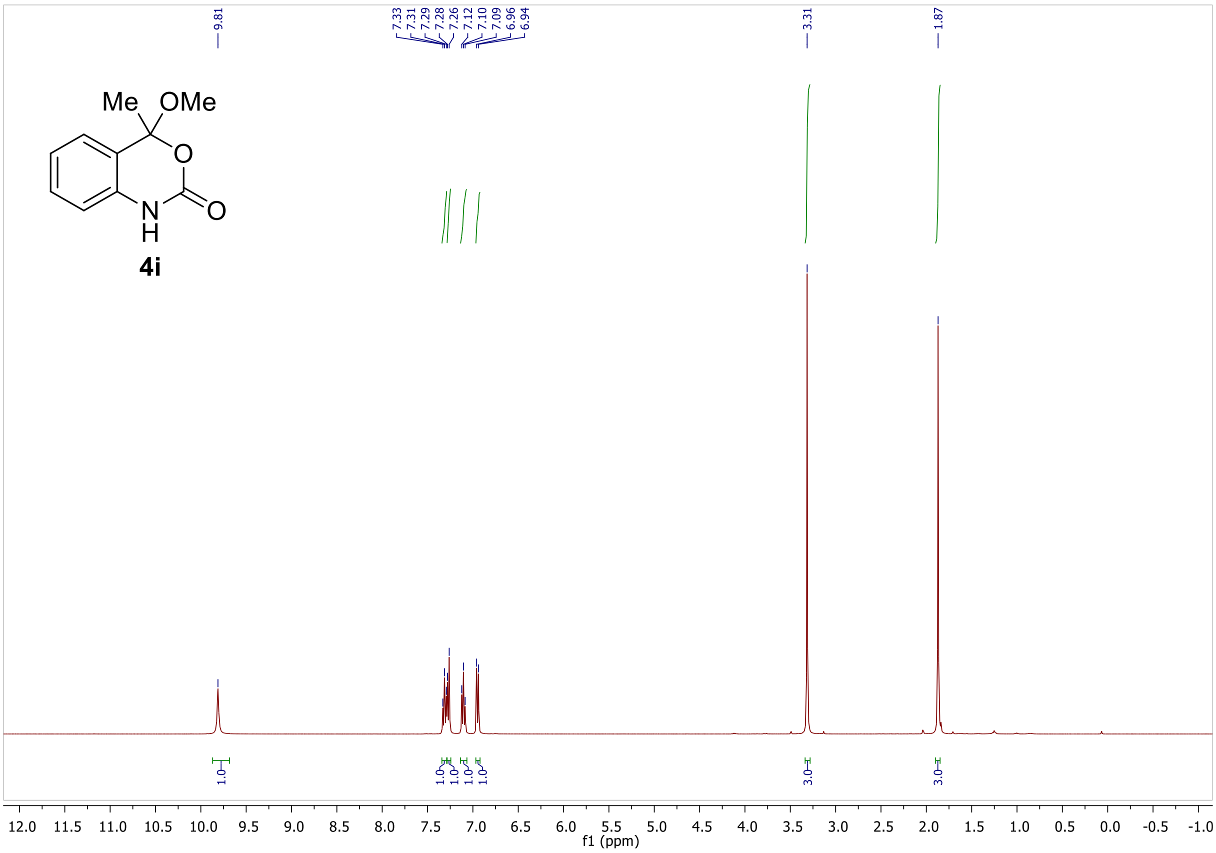


$^{1}H$ NMR in $CDCl_3$ at 400 MHz

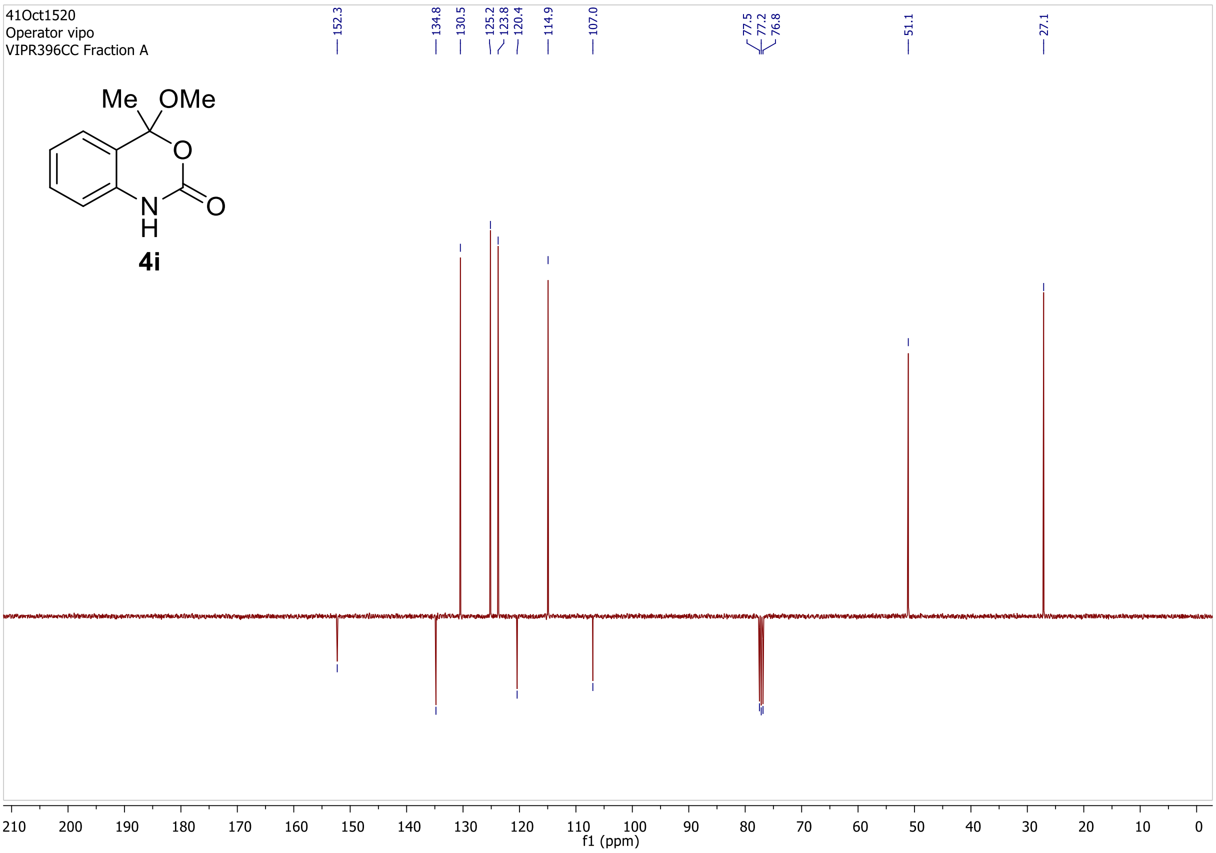


$^{13}C\{^{1}H\}$ NMR in $CDCl_3$ at 100 MHz

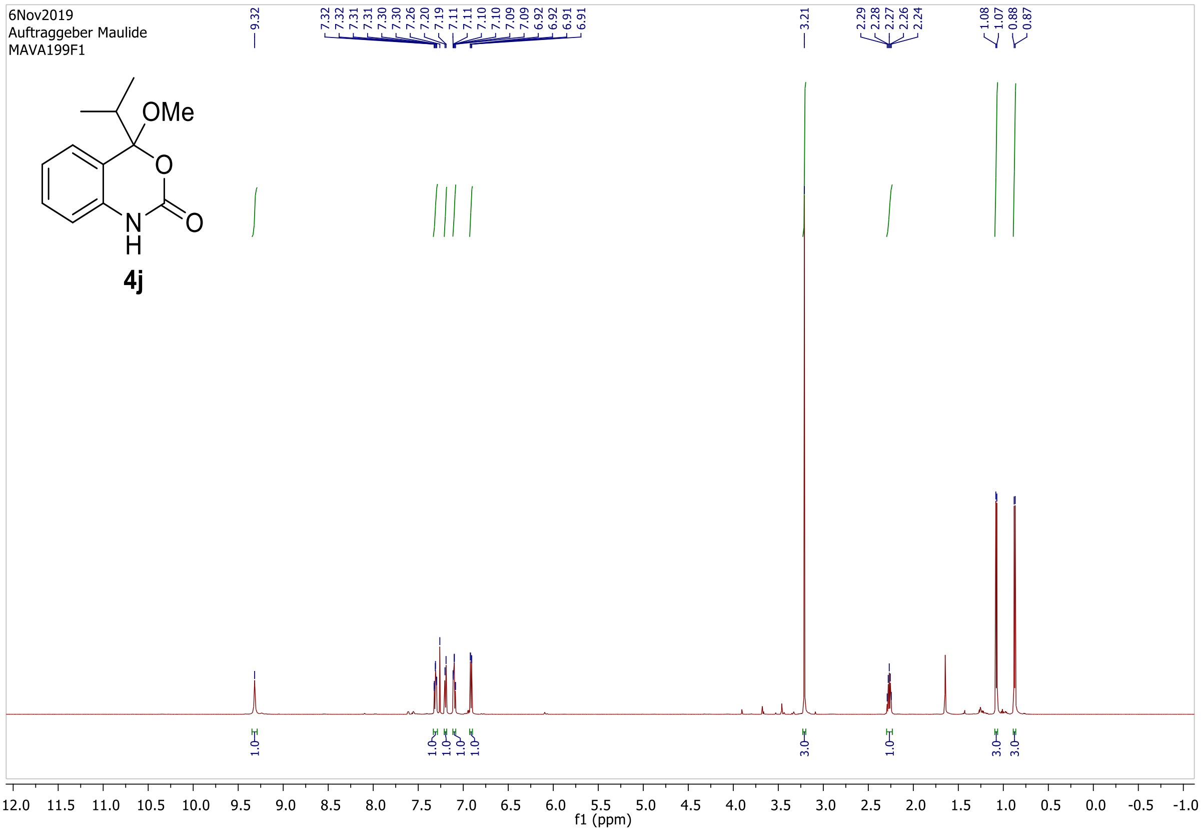


$^{1}$H NMR in $CDCl_3$ at 600 MHz

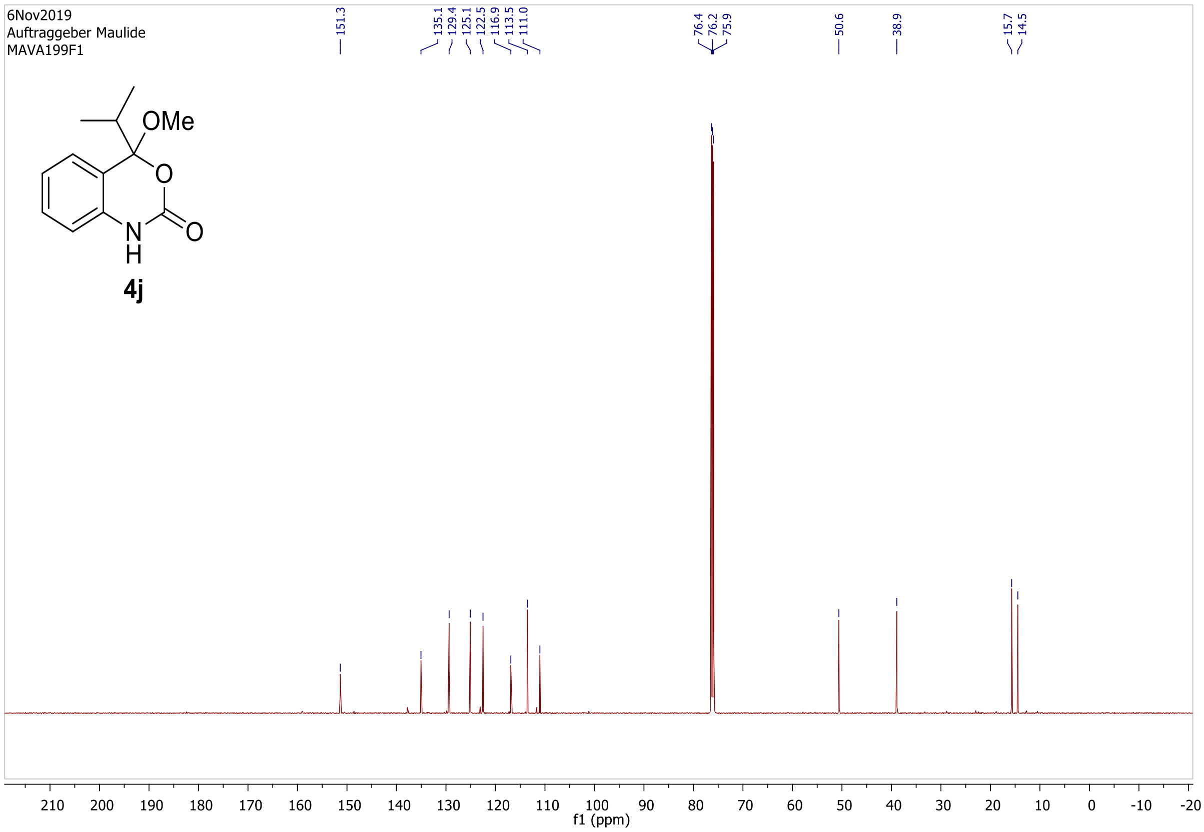


$^{13}$C{$^{1}$H} NMR in $CDCl_3$ at 150 MHz

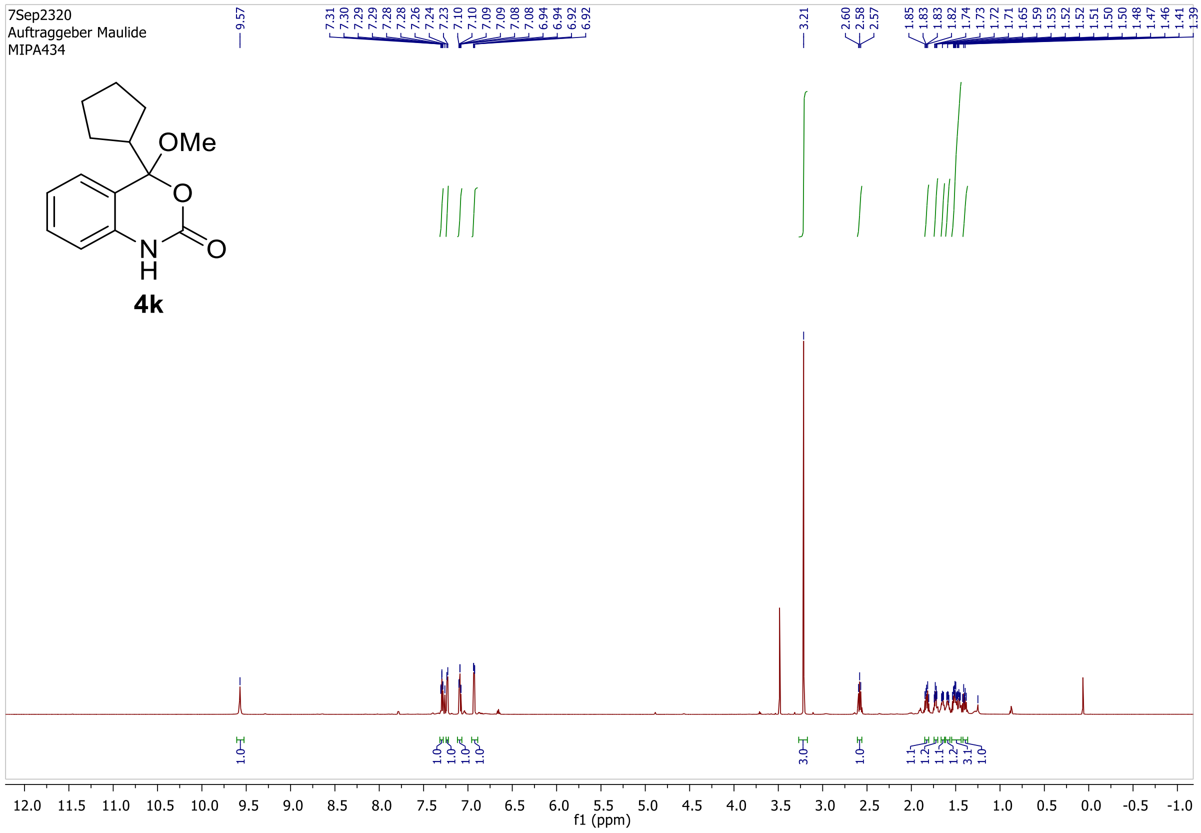


$^{1}H$ NMR in $CDCl_3$ at 700 MHz

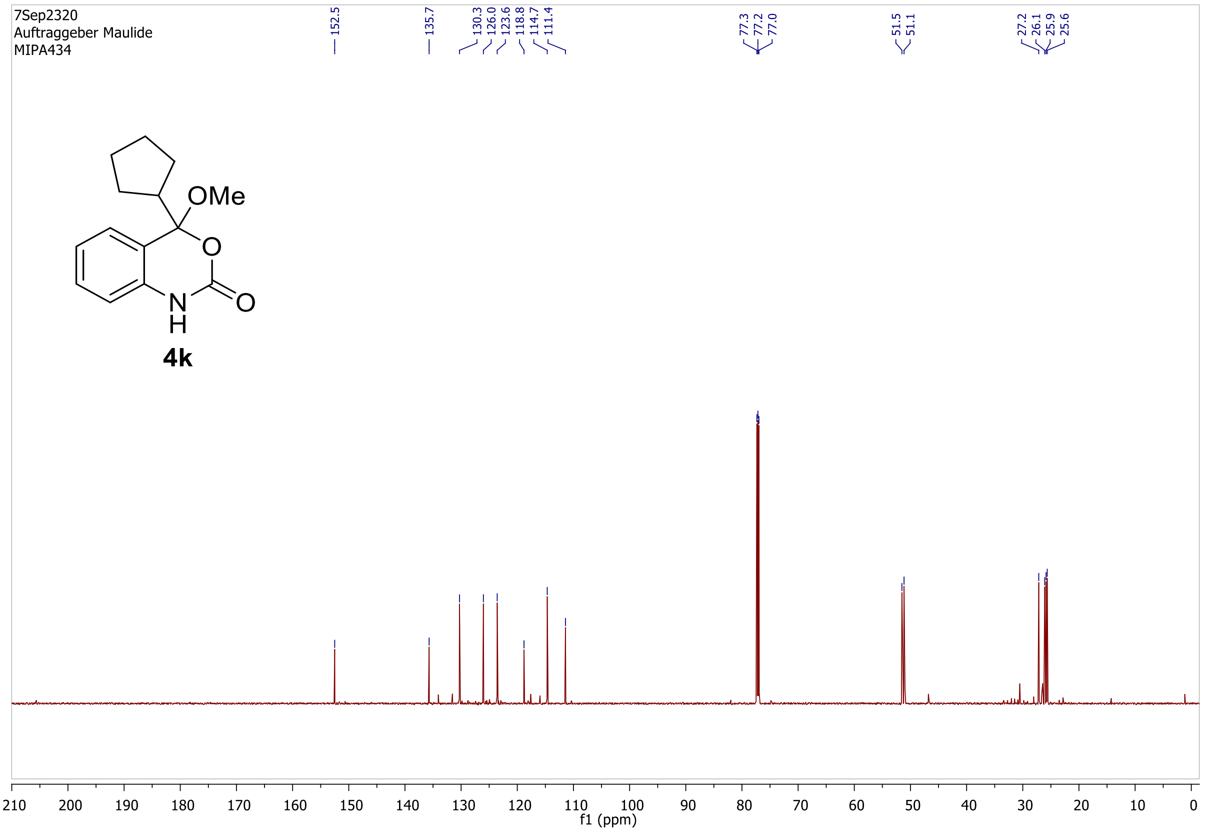


$^{13}C\{^{1}H\}$ NMR in $CDCl_3$ at 176 MHz

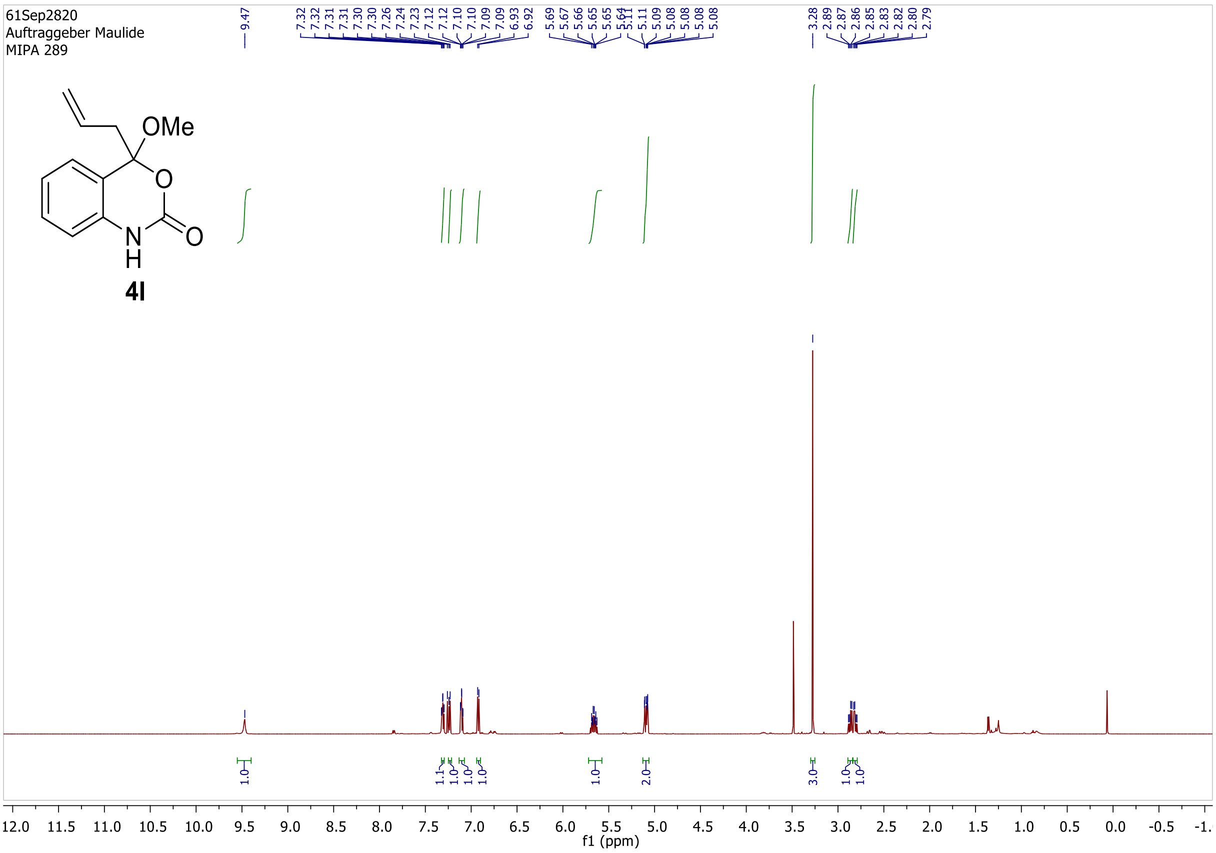


$^{1}$H NMR in $CDCl_3$ at 700 MHz

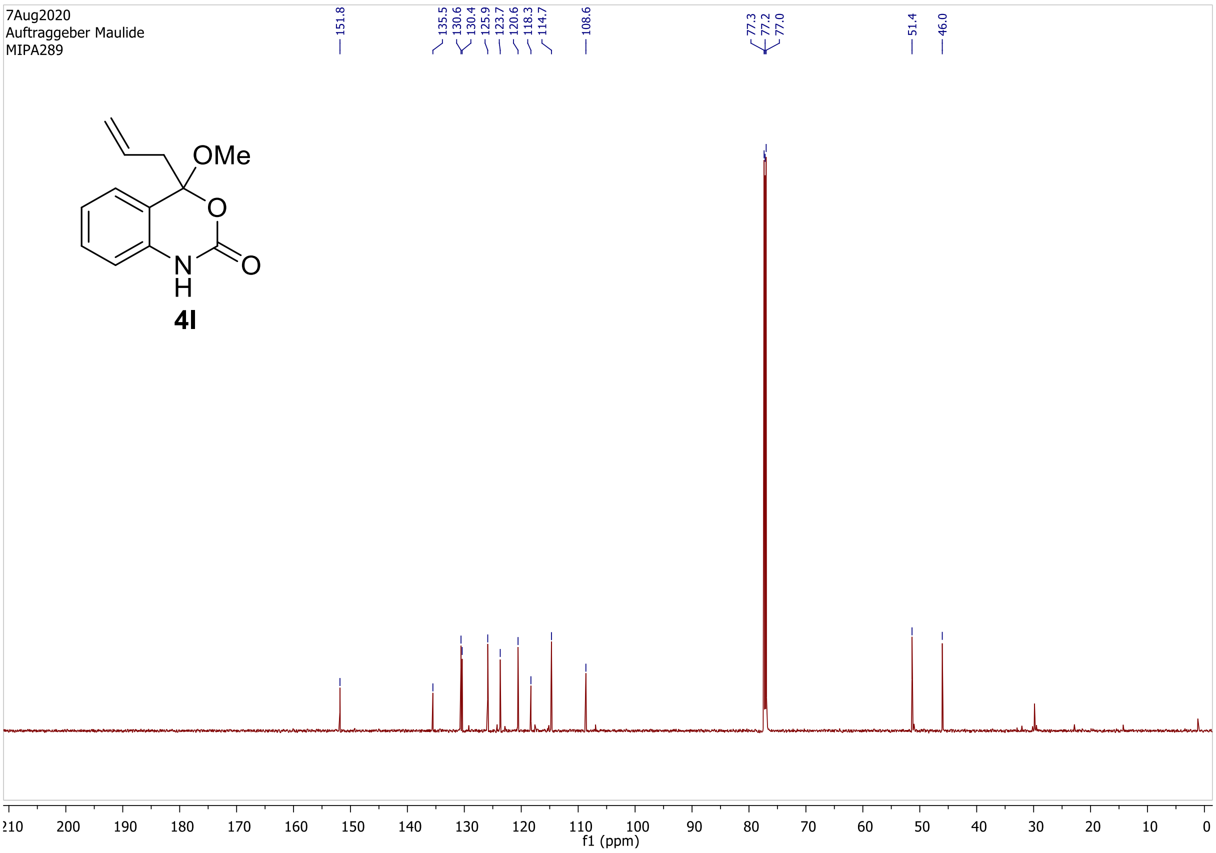


$^{13}C\{^{1}H\}$ NMR in $CDCl_3$ at 176 MHz

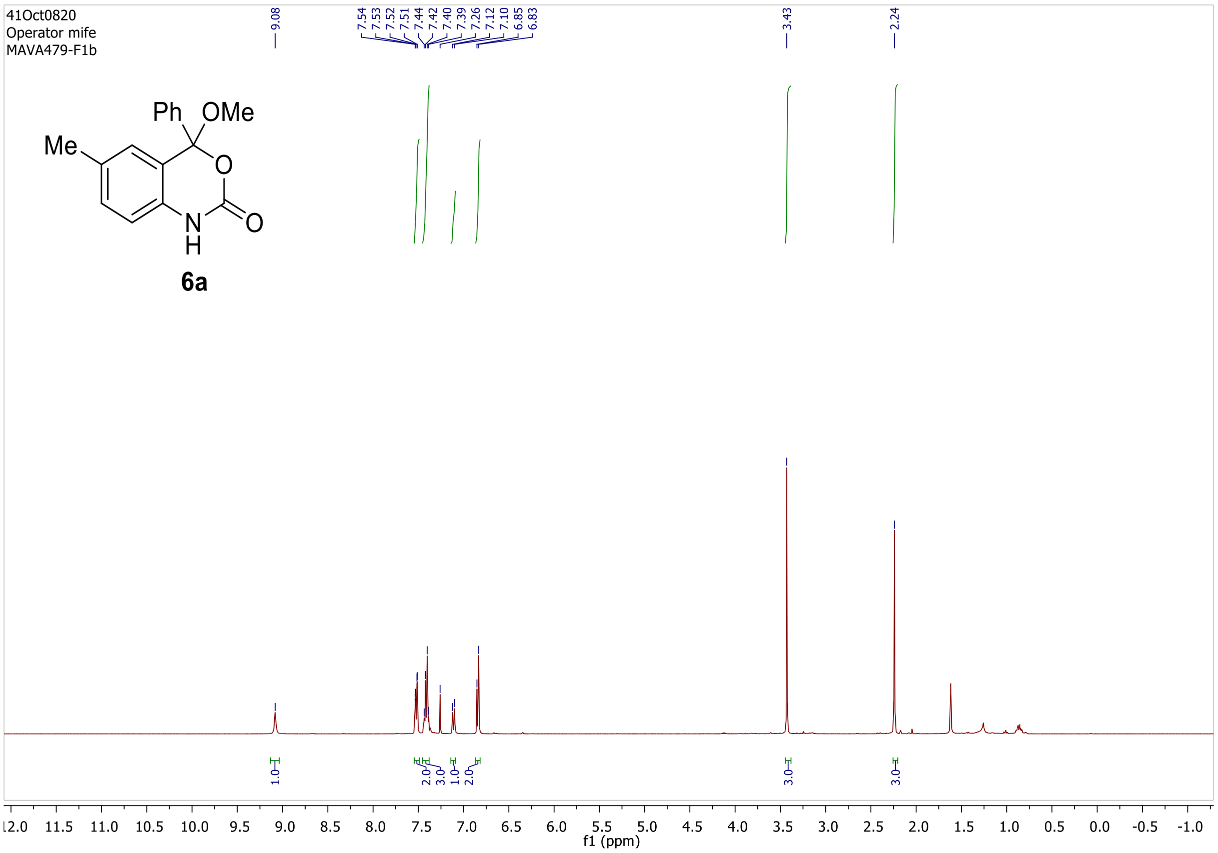


$^{1}$H NMR in $CDCl_3$ at 400 MHz

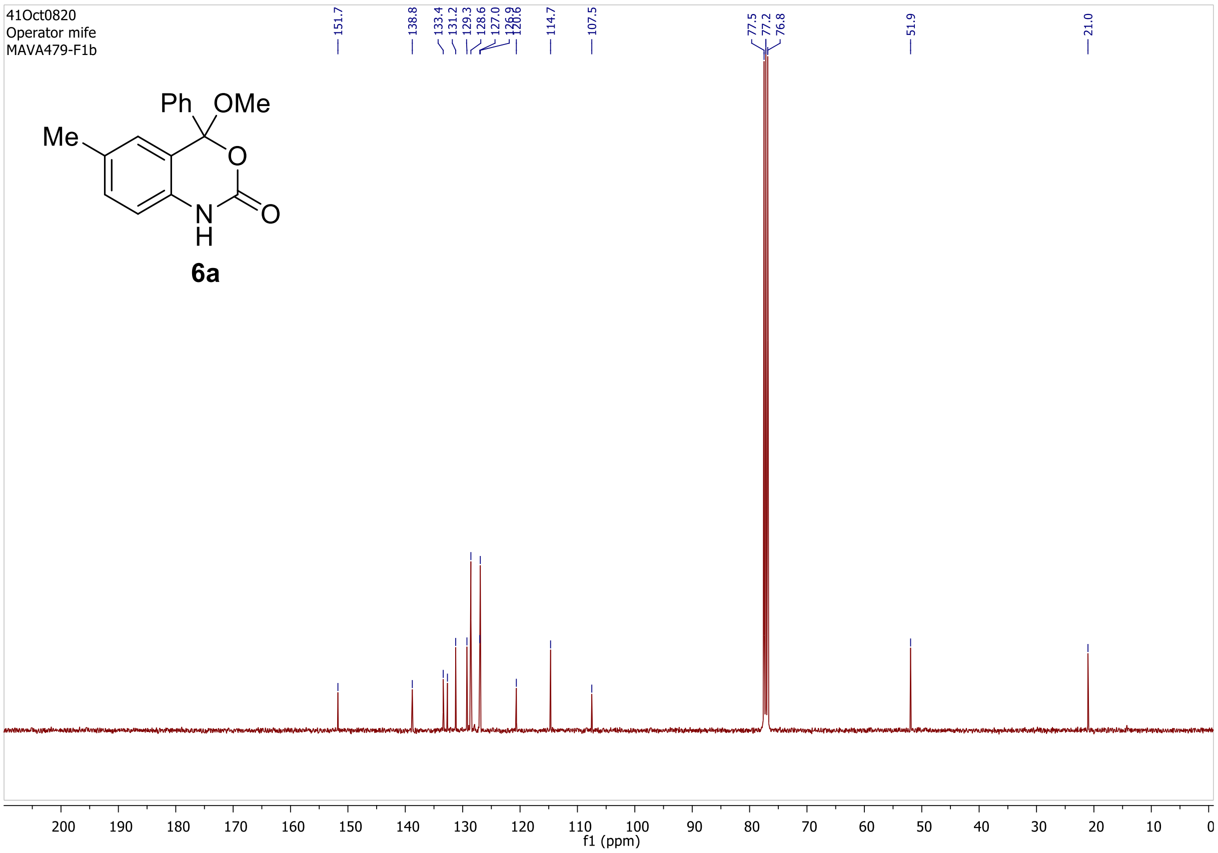


$^{13}C\{^{1}H\}$ NMR in $CDCl_3$ at 100 MHz

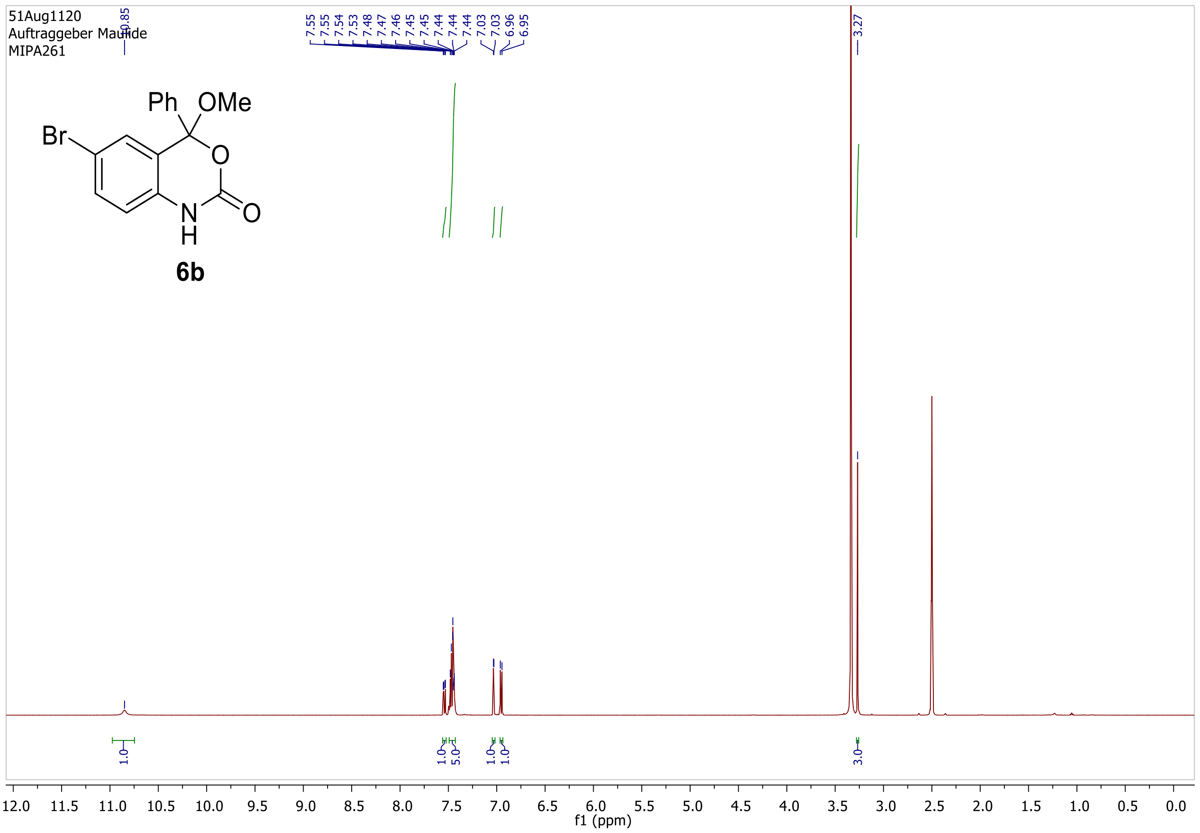


$^{1}H$ NMR in $CDCl_3$ at 500 MHz

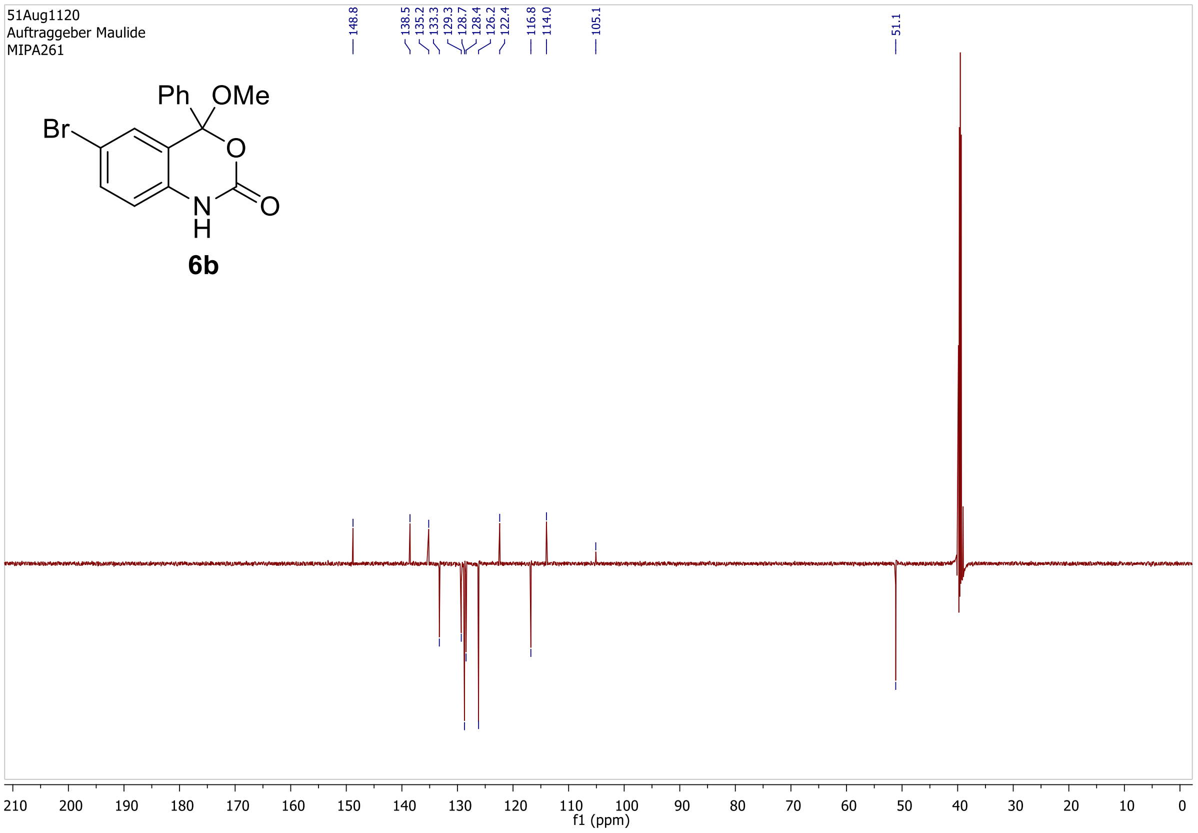


$^{13}C\{^{1}H\}$ NMR in $CDCl_3$ at 126 MHz

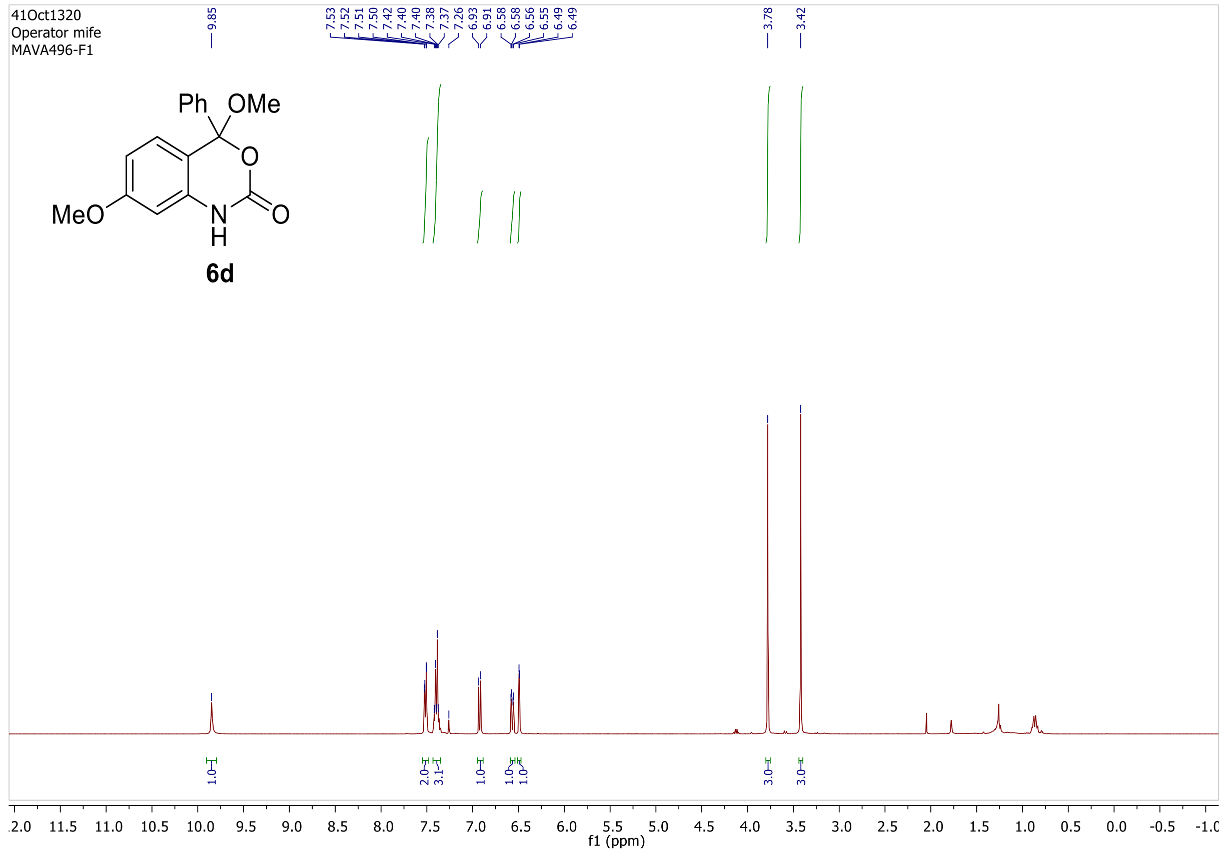


$^{1}$H NMR in $CDCl_3$ at 400 MHz

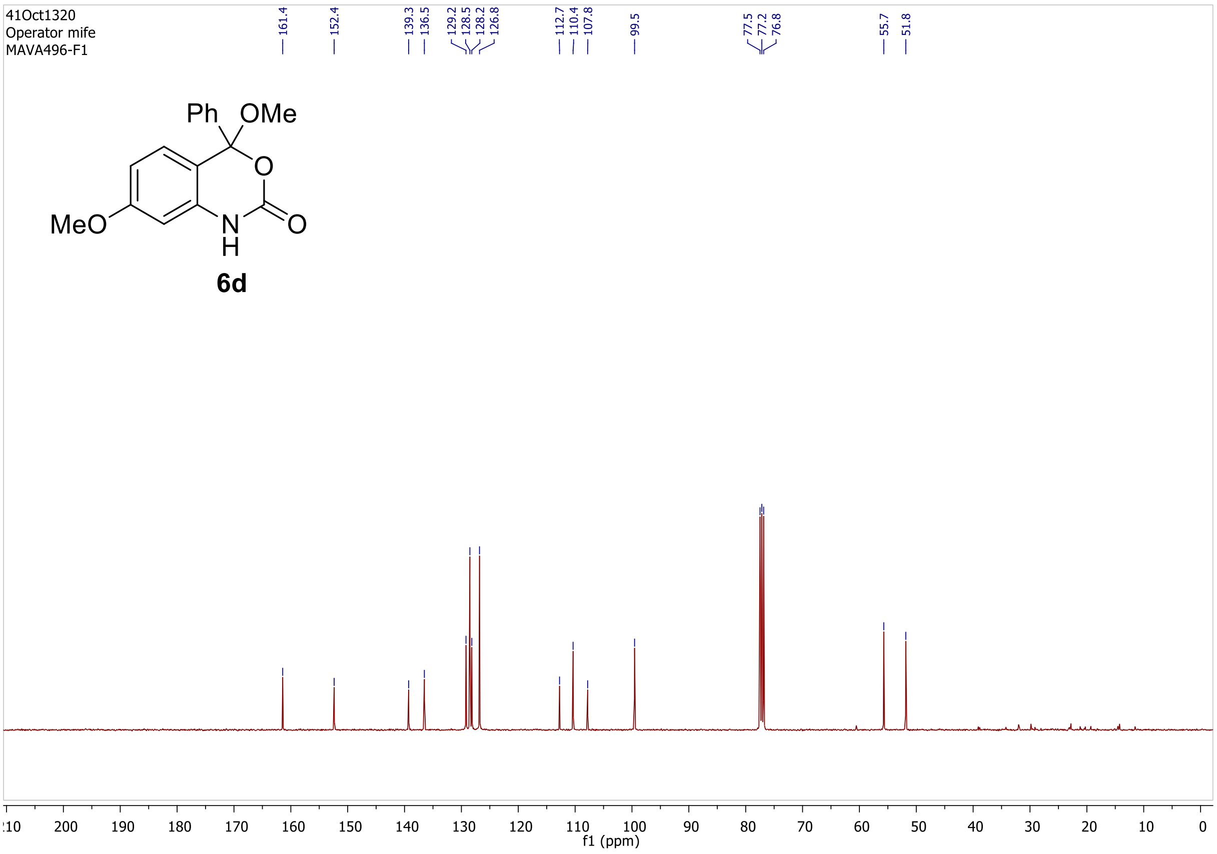


$^{13}$C{$^{1}$H} NMR in $CDCl_3$ at 100 MHz

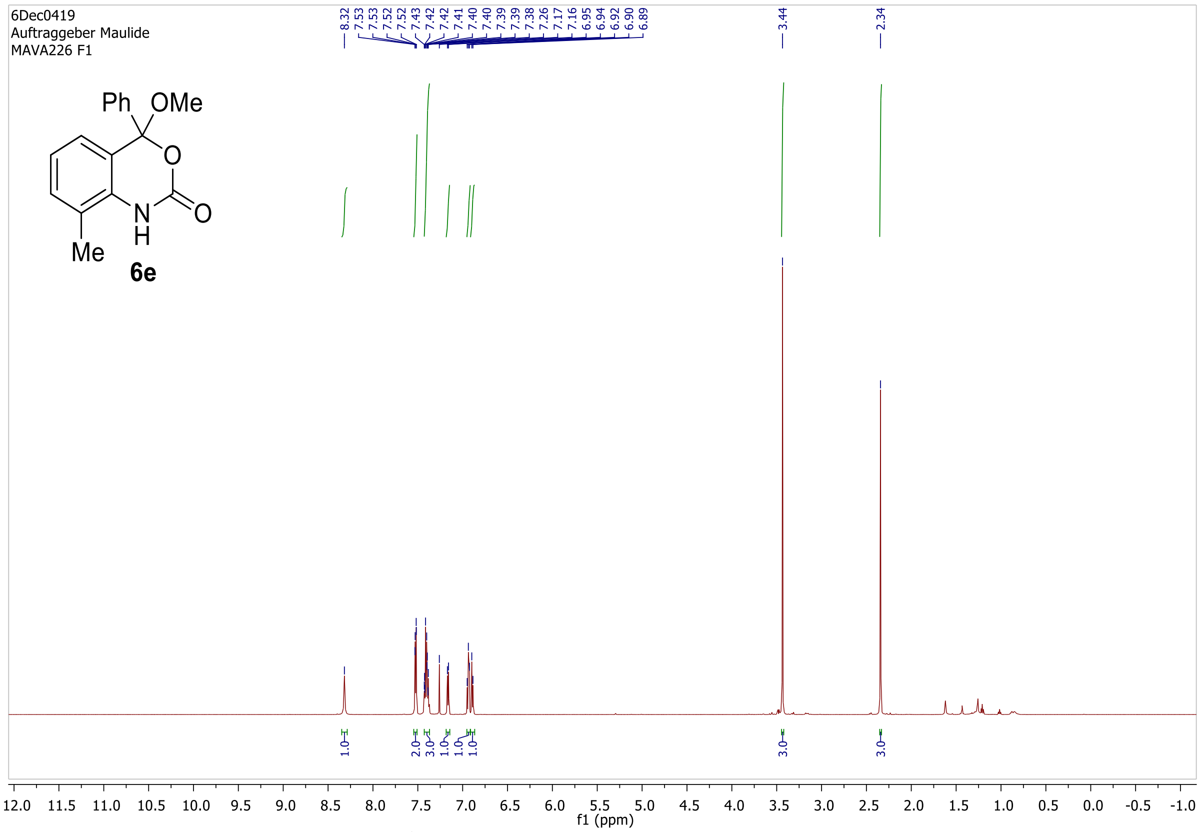


$^{1}$H NMR in $CDCl_3$ at 600 MHz

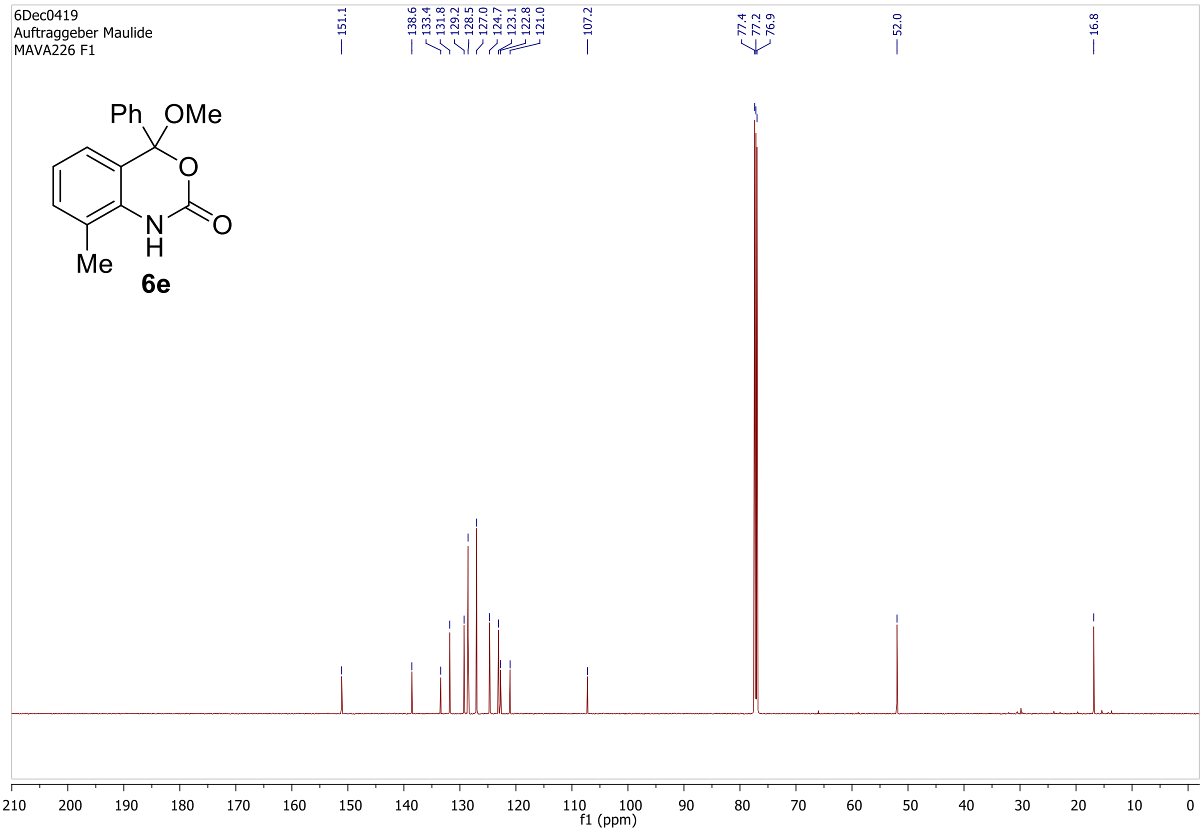


$^{13}C\{^{1}H\}$ NMR in $CDCl_3$ at 150 MHz

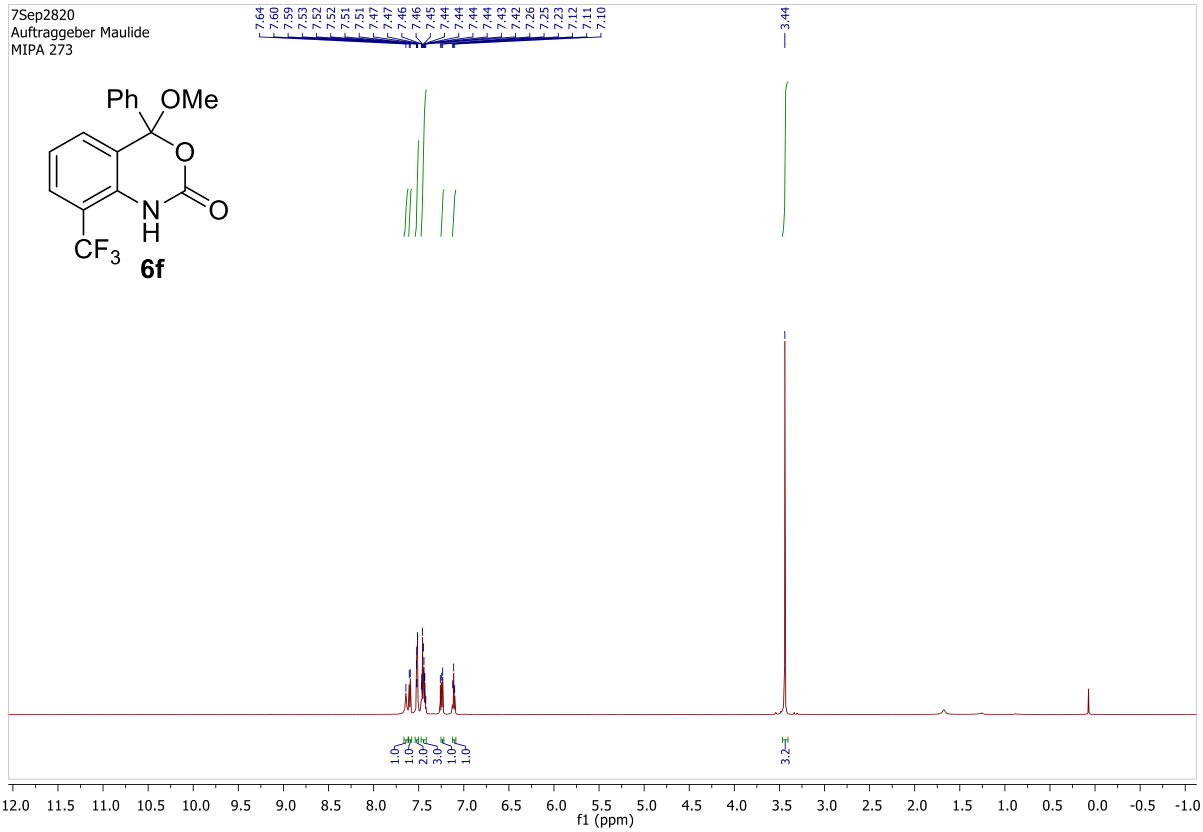


$^{1}$H NMR in $CDCl_3$ at 700 MHz

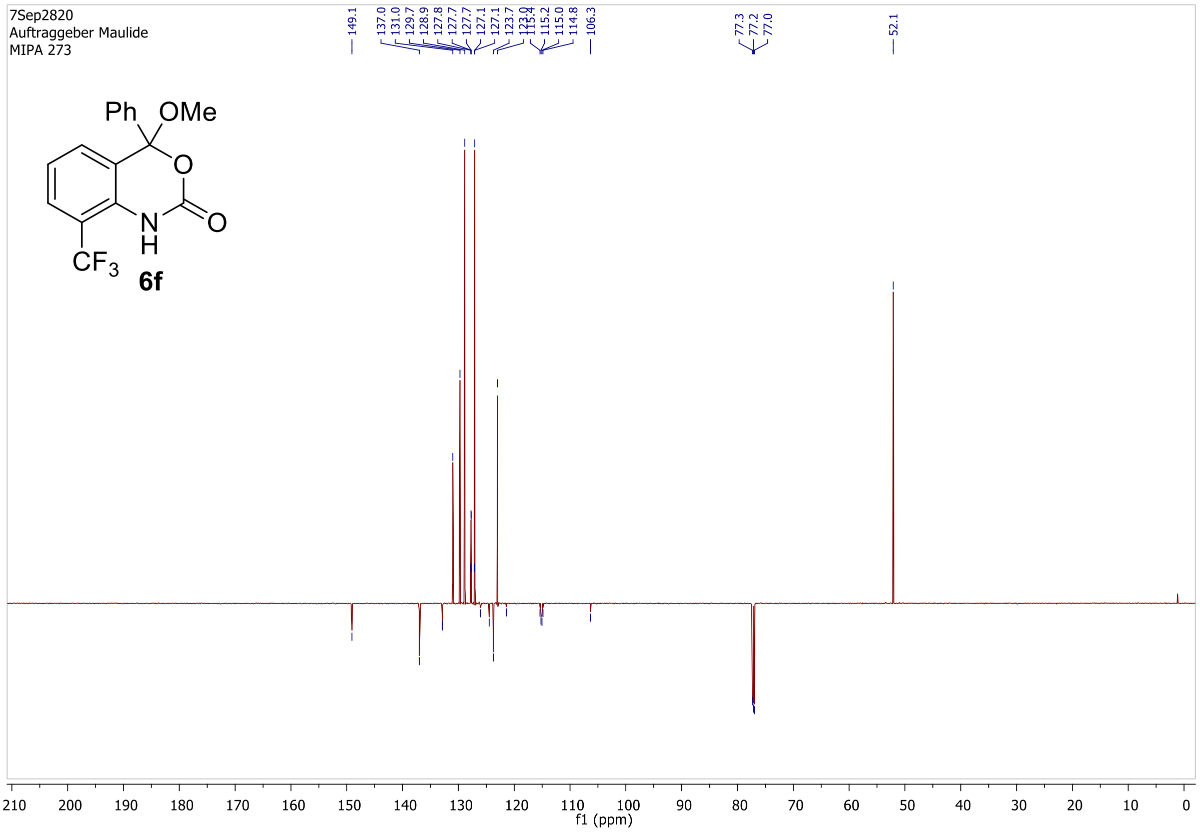


$^{13}C\{^{1}H\}$ NMR in $CDCl_3$ at 175 MHz

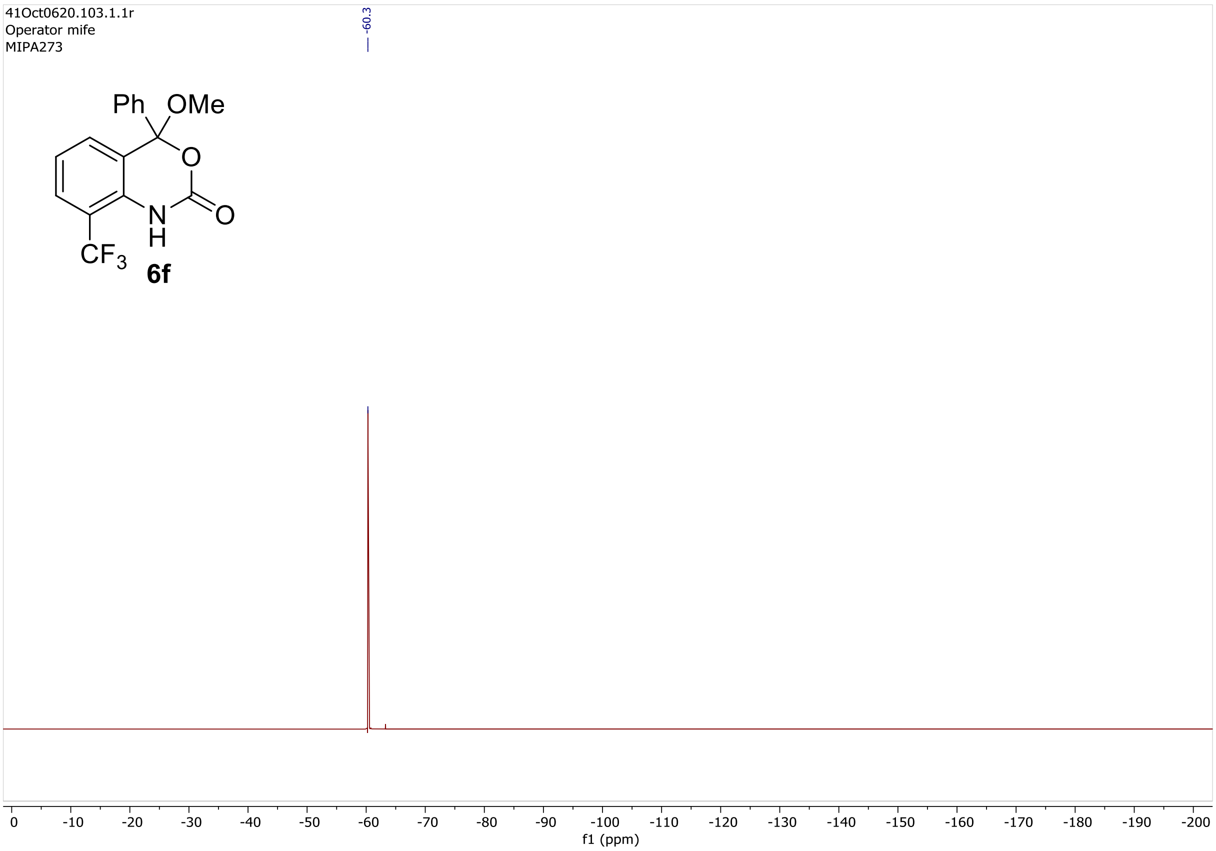


$^{19}$F NMR in DMSO-$d_6$ at 376 MHz

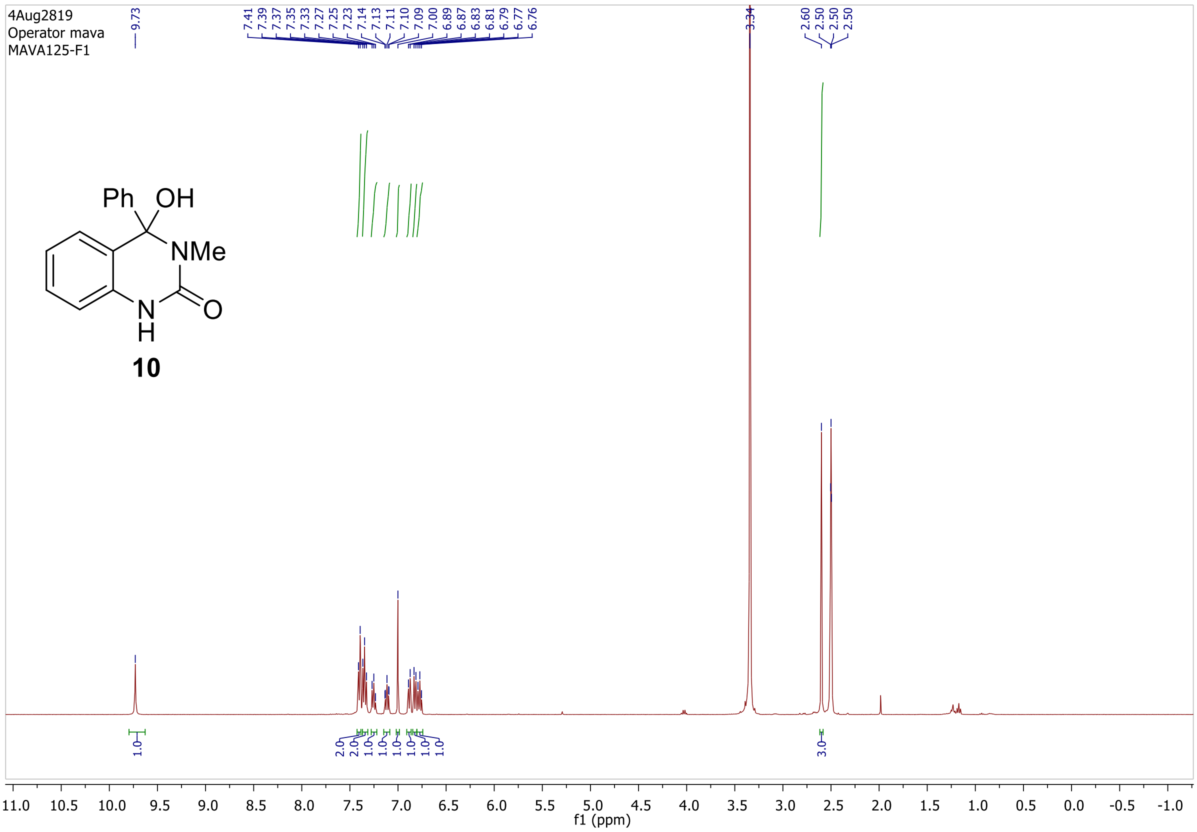


$^{1}H$ NMR in $CDCl_3$ at 400 MHz

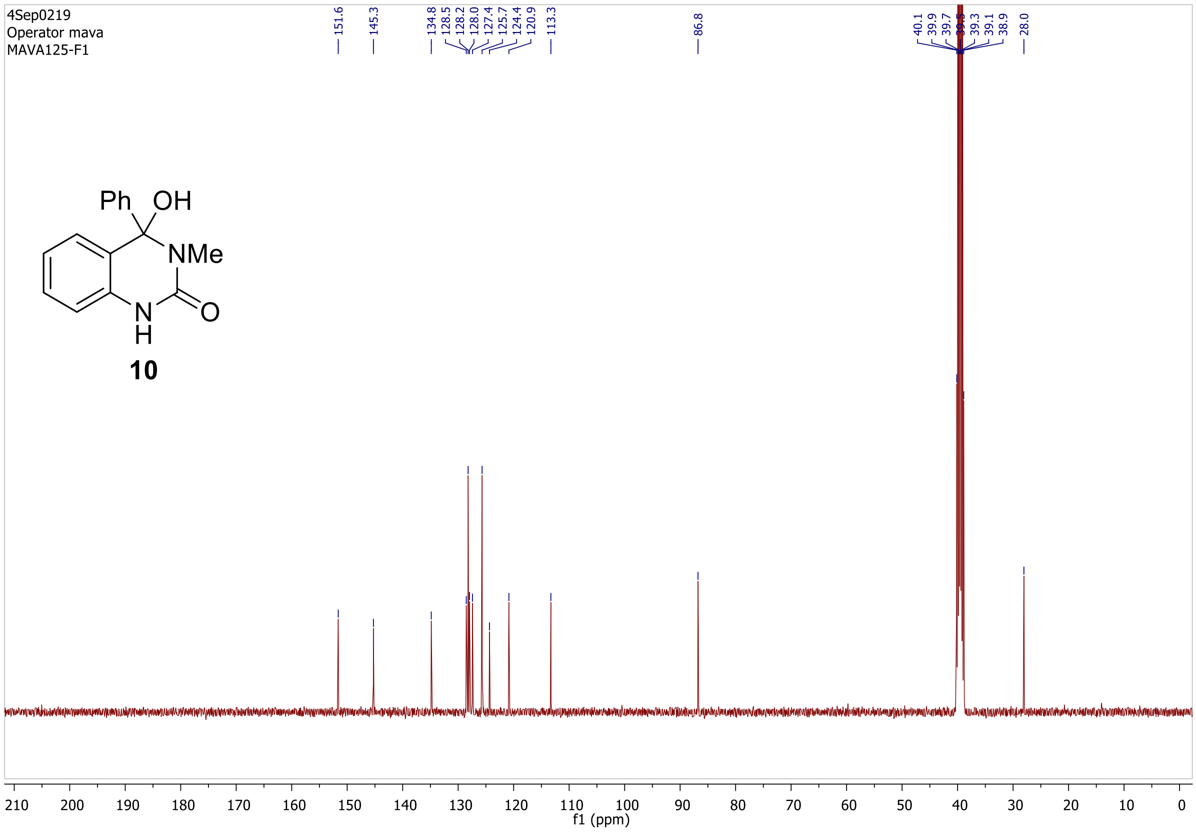


$^{13}C\{^{1}H\}$ NMR in $CDCl_3$ at 100 MHz

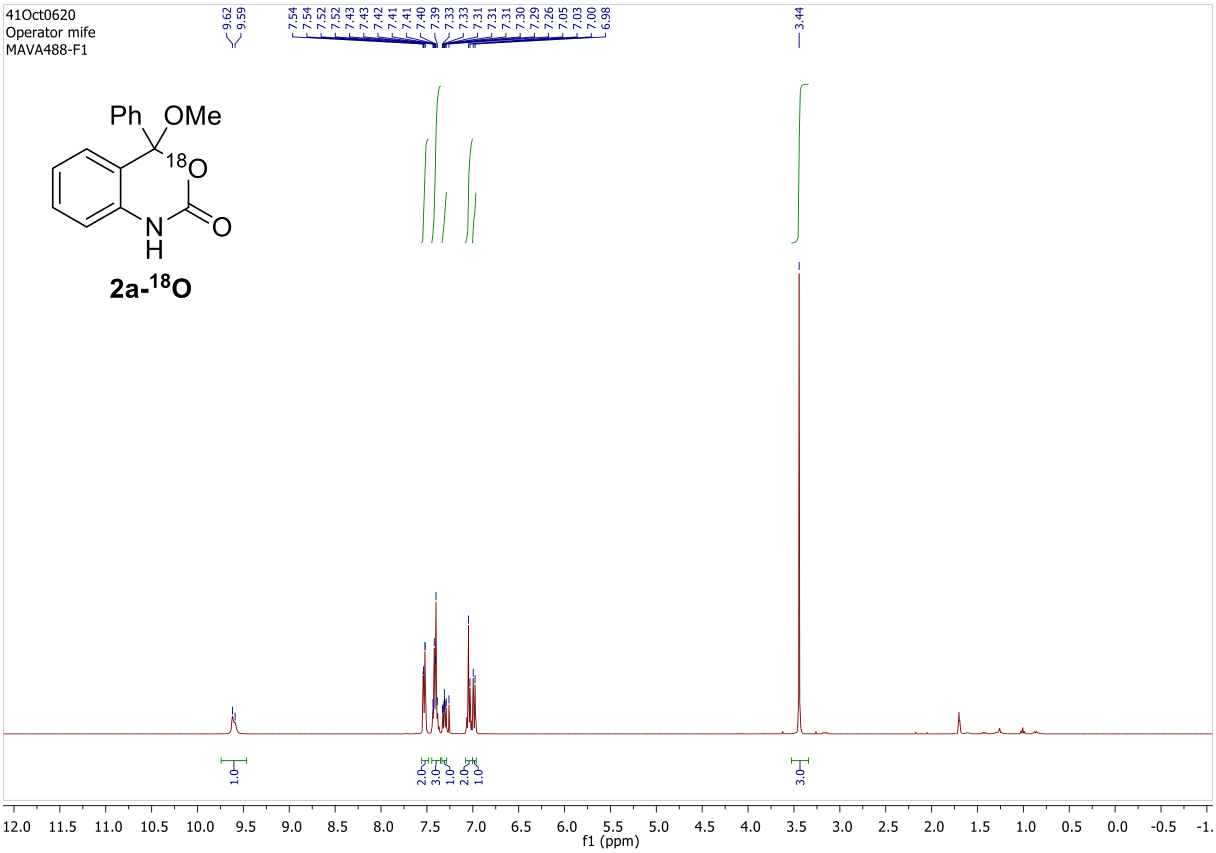


$^{1}H$ NMR in $CDCl_3$ at 400 MHz

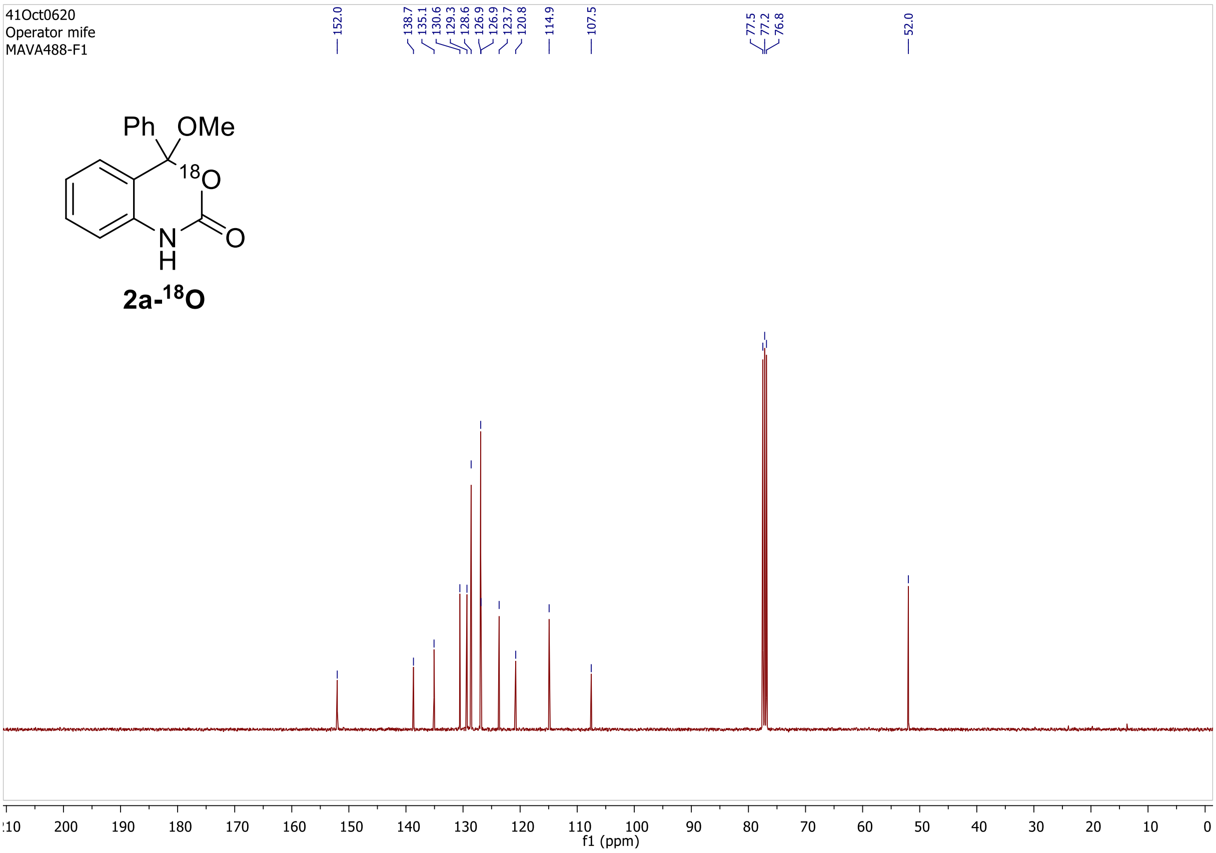


$^{13}C\{^{1}H\}$ NMR in $CDCl_3$ at 100 MHz

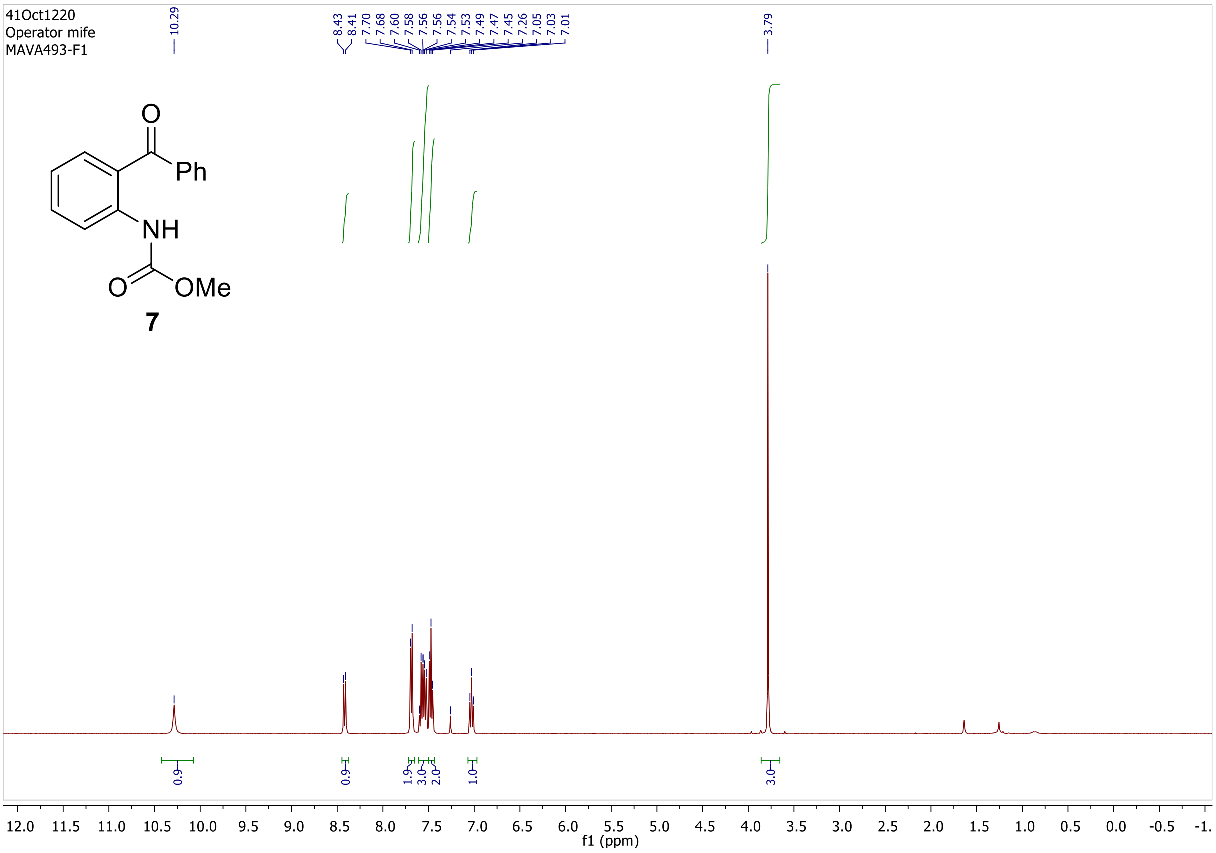

$^1$H NMR in $CDCl_3$ at 400 MHz

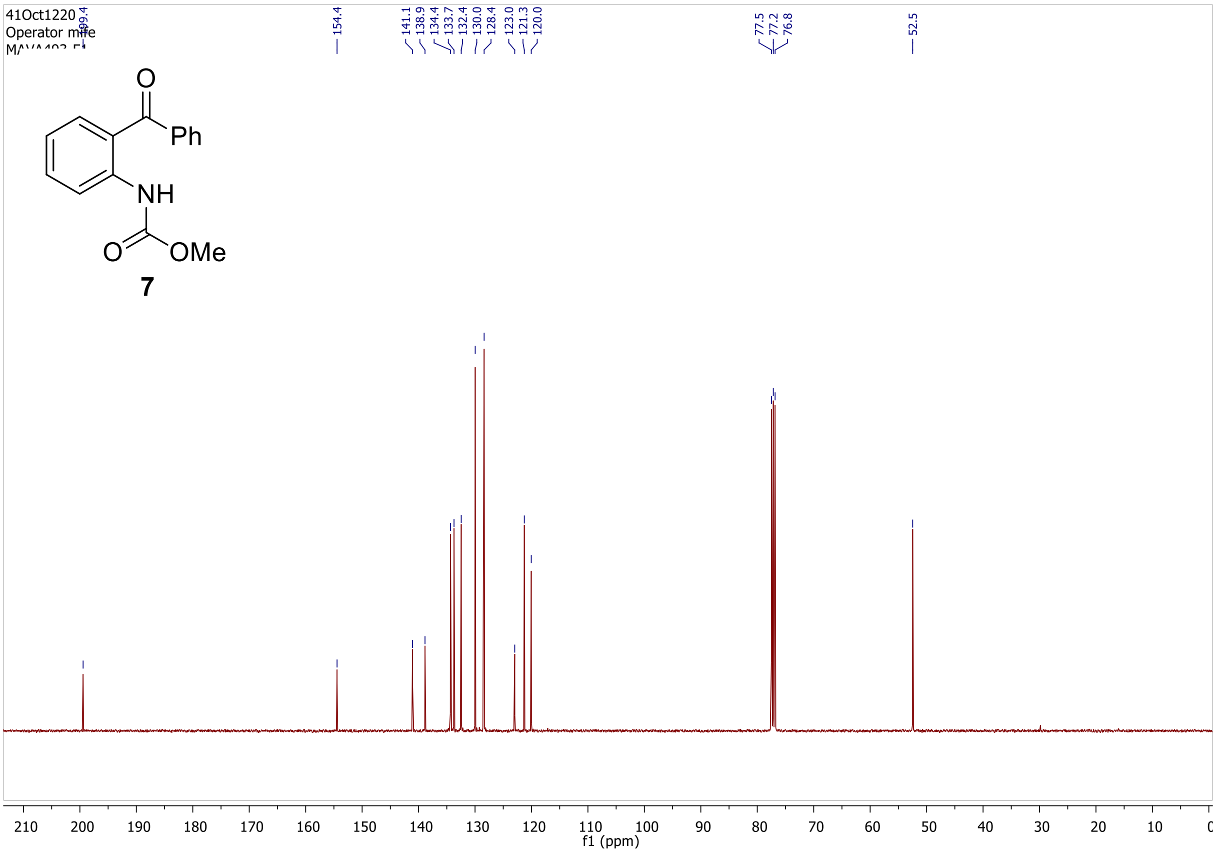

$^{13}C\{^1H\}$ NMR in $CDCl_3$ at 100 MHz

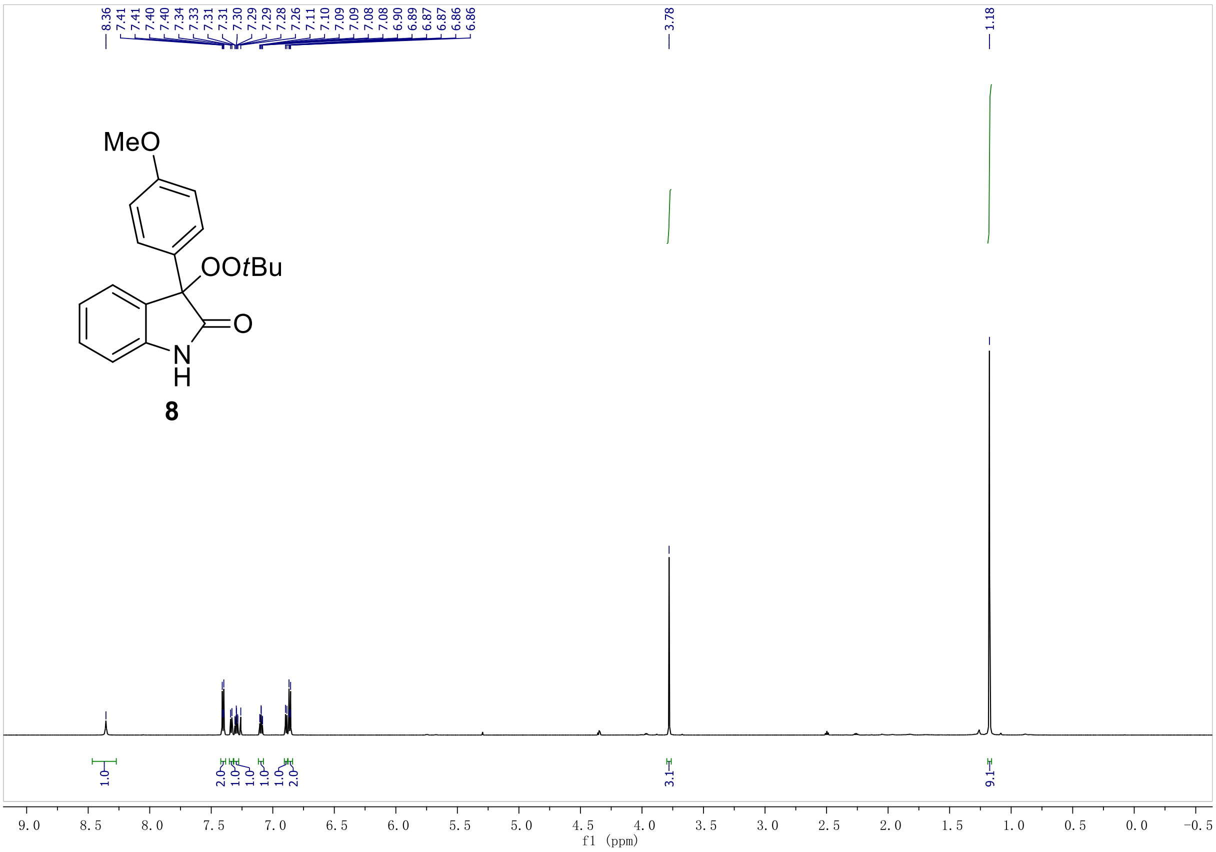


$^{1}$H NMR in $CDCl_3$ at 700 MHz

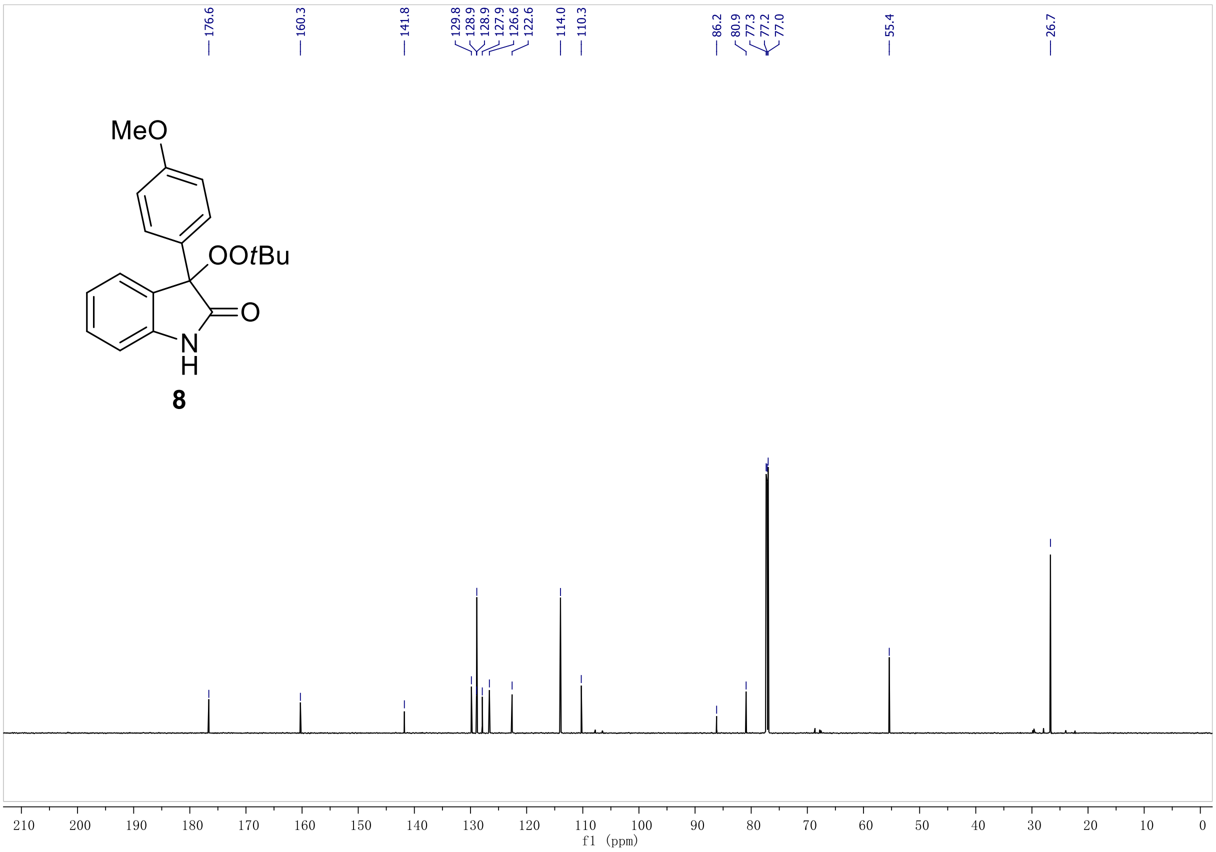




$^{13}$C NMR in $CDCl_3$ at 176 MHz

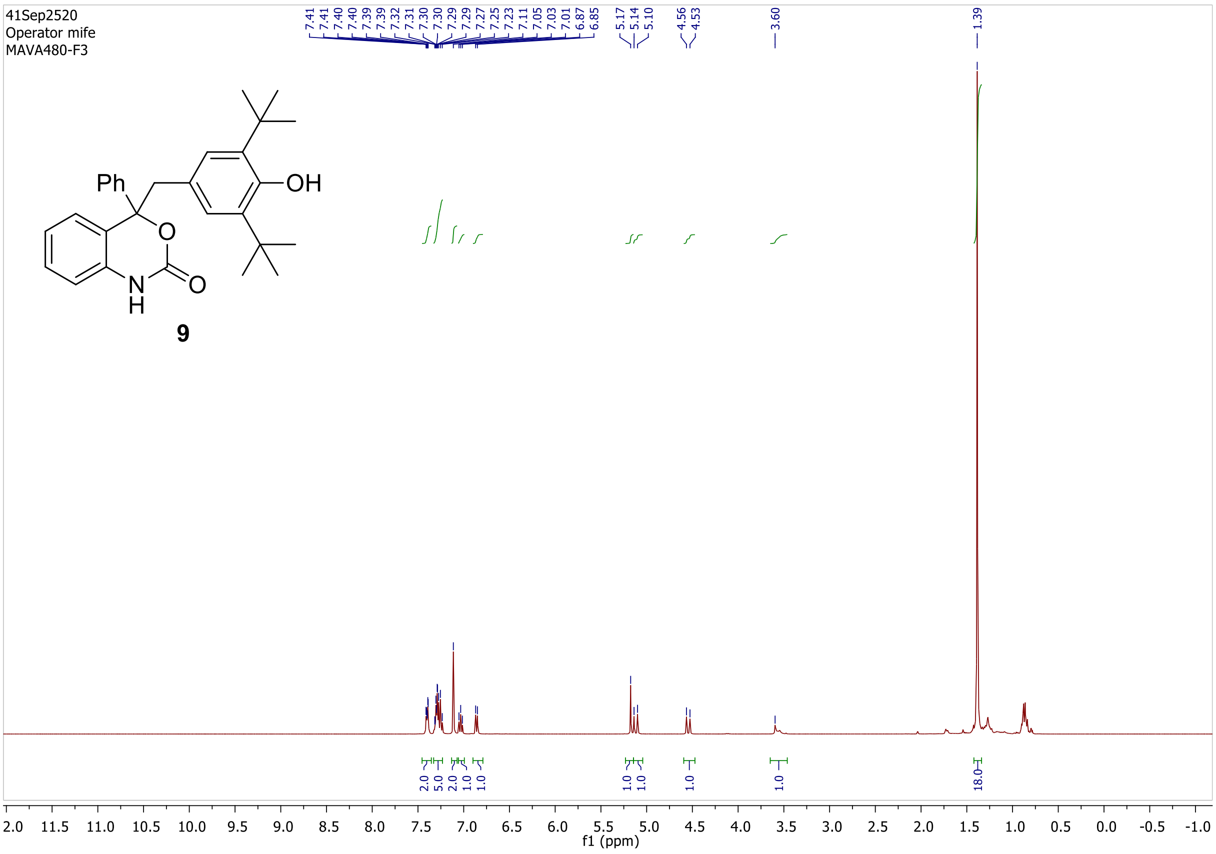


$^1$H NMR in CDCl$_3$ at 400 MHz

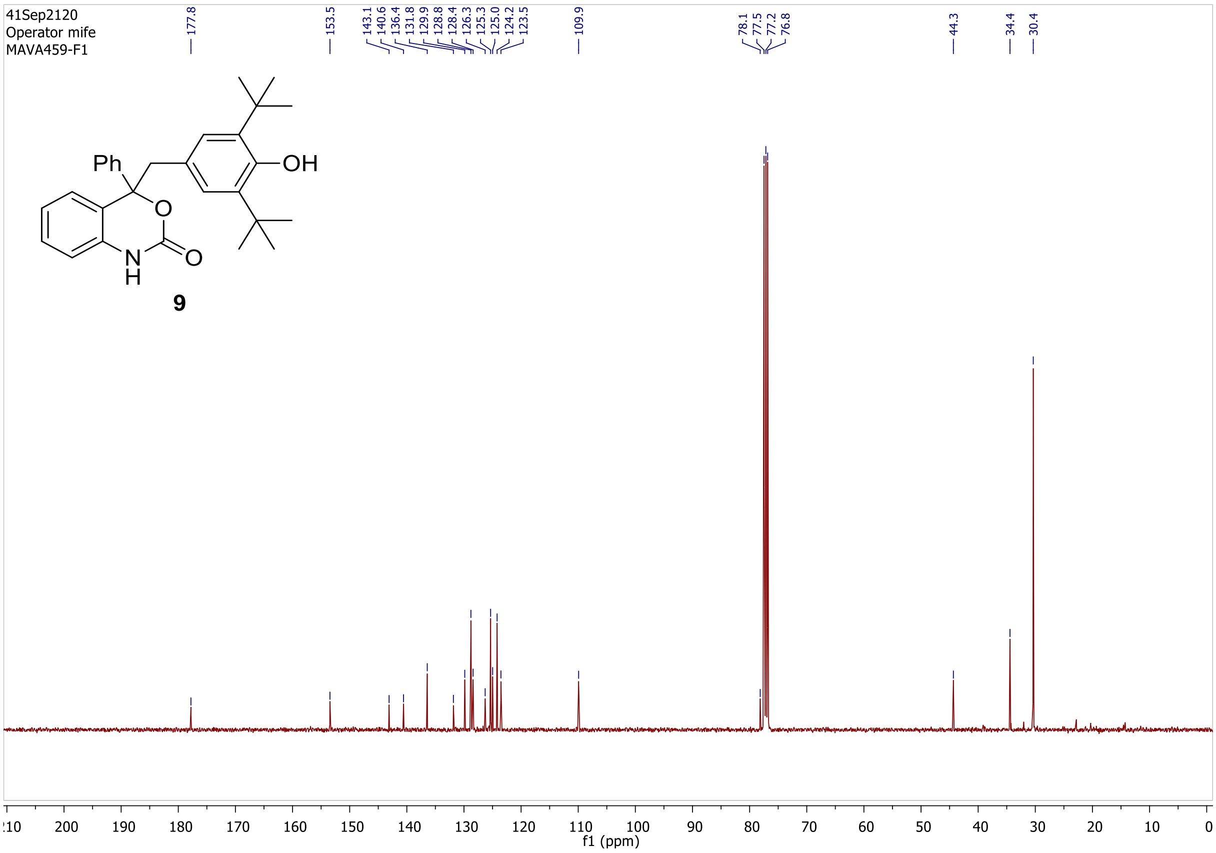


$^{13}$C{$^1$H} NMR in CDCl$_3$ at 100 MHz

# 8 References


[1] Ma, S.; Han, X.; Krishnan, S.; Virgil, S. C.; Stoltz, B. M. Catalytic Enantioselective Stereoablative Alkylation of 3-Halooxindoles: Facile Access to Oxindoles with C3 All-Carbon Quaternary Stereocenters. *Angew. Chem. Int. Ed.* **2009,** *48*, 8037–8041.

[2] Toullec, P. Y.; Jagt, R. B. C.; de Vries, J. G.; Feringa, B. L.; Minnaard, A. J. Rhodium-Catalyzed Addition of Arylboronic Acids to Isatins: An Entry to Diversity in 3-Aryl-3-Hydroxyoxindoles. *Org. Lett.* **2006,** *8*, 2715–2718.

[3] Gade, A. B.; Bagle, P. N.; Shinde, P. S.; Bhardwaj, V.; Banerjee, S.; Chande, A.; Patil, N. T. Catalytic Enantioselective 1,3-Alkyl Shift in Alkyl Aryl Ethers: Efficient Synthesis of Optically Active 3,3′-Diaryloxindoles. *Angew. Chem. Int. Ed.* **2018**, *57*, 5735-5739.

[4] Xiao, Z. -K.; Yin, H. -Y.; Shao, L. -X. N-Heterocyclic Carbene-Palladium(II)-1-Methylimidazole Complex Catalyzed α-Arylation of Oxindoles with Aryl Chlorides and Aerobic Oxidation of the Products in a One-Pot Procedure. *Org. Lett.* **2013**, *15*, 1254–1257.

[5] Ghosh, S.; Kinthada, L. K.; Bhunia, S.; Bisai, A. Lewis acid-catalyzed Friedel–Crafts alkylations of 3-hydroxy-2-oxindole: an efficient approach to the core structure of azonazine. *Chem. Commun.* **2012,** *48*, 10132–10134.

[6] Chaudhari, M. B.; Sutar, Y.; Malpathak, S.; Hazra, A.; Gnanaprakasam, B. Transition-Metal-Free C–H Hydroxylation of Carbonyl Compounds. *Org. Lett.* **2017,** *19*, 3628–3631.

[7] Yin, L.; Kanai, M.; Shibasaki, M. A Facile Pathway to Enantiomerically Enriched 3-Hydroxy-2-Oxindoles: Asymmetric Intramolecular Arylation of α-Keto Amides Catalyzed by a Palladium–DifluorPhos Complex. *Angew. Chem. Int. Ed*. **2011,** 50, 7620–7623.

[8] Verschueren, K.; Cobbaut, M.; Demaerel, J.; Saadah, L.; Voet, A. R. D.; Van Lint, J.; De Borggraeve, W. M. Discovery of a potent protein kinase D inhibitor: insights in the binding mode of pyrazolo[3,4-d]pyrimidine analogues. *Med. Chem. Commun.* **2017**, *8*, 640-646.

[9] Parrick, J.; Yahya, A.; Ijaz, A. S.; Yizun, J. Convenient preparation of 3,3-dibromo-1,3-dihydroindol-2-ones and indole-2,3-diones (isatins) from indoles. *J. Chem. Soc., Perkin Trans. 1* **1989**, 2009-2015.

[10] Klare, H. F. T.; Goldberg, A. F. G.; Duquette, D. C.; Stoltz, B. M. Oxidative Fragmentations and Skeletal Rearrangements of Oxindole Derivatives. *Org. Lett.* **2017**, *19*, 988-991.

[11] Bruker SAINT v8.38B Copyright © 2005-2019 Bruker AXS.

[12] Sheldrick, G. M. (1996). *SADABS.* University of Göttingen, Germany.

[13] Dolomanov, O. V.; Bourhis, L. J.; Gildea, R. J.; Howard, J. A. K.; Puschmann, H. OLEX2, *J. Appl. Cryst.* **2009**, *42*, 339-341.

[14] Huebschle, C. B.; Sheldrick, G. M.; Dittrich, B. ShelXle: a Qt graphical user interface for SHELXL, J. Appl. Cryst. **2011**, *44*, 1281-1284.

[15] Sheldrick, G. M. (2015). *SHELXS v 2016/4* University of Göttingen, Germany.

[16] Spek, A. L. Structure validation in chemical crystallography. *Acta Cryst.* **2009**, D65, 148-155.
